# Chemical Synthesis of *Pseudomonas aeruginosa*, *Staphylococcus aureus*, and *Acinetobacter baumannii* Capsular Polysaccharide Fragments as Leads for Cross‐Protection

**DOI:** 10.1002/anie.202524231

**Published:** 2025-12-21

**Authors:** Amar Kumar Mishra, Emelie E. Reuber, Diksha Rai, Leif E. Sander, Julia Duerr, Simon Y. Graeber, Marcus A. Mall, Bettina C. Fries, Peter H. Seeberger, Suvarn S. Kulkarni

**Affiliations:** ^1^ Department of Chemistry Indian Institution of Technology Bombay Powai Mumbai India; ^2^ Institute of Chemistry and Biochemistry Freie Universität Berlin Berlin Germany; ^3^ Institution Max Planck Institute of Colloids and Interfaces Potsdam Germany; ^4^ Department of Infectious Diseases and Critical Care Medicine Campus Virchow‐Klinikum and Campus Charité Mitte Charité – Universitätsmedizin Berlin Corporate Member of Freie Universität and Humboldt‐Universität zu Berlin Berlin Germany; ^5^ German Center for Lung Research (DZL) Associated Partner Site Berlin Berlin Germany; ^6^ Berlin Institute of Health at Charité – Universitätsmedizin Berlin Berlin Germany; ^7^ Department of Pediatric Respiratory Medicine Immunology and Critical Care Medicine Charité Universitätsmedizin Berlin Berlin Germany; ^8^ German Center for Child and Adolescent Health (DZKJ) Partner Site Berlin Berlin Germany; ^9^ Cluster of Excellence ImmunoPreCept Charité‐Universitätsmedizin Berlin Berlin Germany; ^10^ Department of Microbiology and Immunology Renaissance School of Medicine Stony Brook University Stony Brook New York 11794 USA; ^11^ Division of Infectious Diseases Department of Medicine Stony Brook University Stony Brook New York 11794 USA; ^12^ Veterans Administration Medical Center Northport NY 11768 USA

**Keywords:** ESKAPE pathogens, Glycan immunology, Microarray studies, Rare deoxy amino sugars, Total synthesis

## Abstract

*Pseudomonas aeruginosa* and *Staphylococcus aureus* are listed by the World Health Organization as high‐priority multidrug‐resistant (MDR) pathogens, whereas *Acinetobacter baumannii* is classified as the critical‐priority group. These bacteria cause life‐threatening infections such as severe bloodstream, nosocomial, urinary tract, and soft‐tissue infections. Their cell surfaces display complex and structurally distinct glycans absent in host cells, making them targets for glycoconjugate vaccine and diagnostic research. In this study, we report the chemical synthesis of mono‐ and oligosaccharide fragments derived from three ESKAPE pathogens, *P. aeruginosa* O11, *S. aureus* (CP5, CP8, and strain M), and *A. baumannii* (S34 and O5), as well as *Plesiomonas shigelloides* O1. Glycan microarray screening revealed three epitopes exhibiting strong cross‐reactive immunogenicity against *P. aeruginosa*, *S. aureus*, and *A. baumannii*, demonstrating that a trisaccharide represents the minimal epitope required to elicit cross‐protective immune responses. The key features of *P. aeruginosa* O11 trisaccharide synthesis involve efficient assembly of a 1,2‐*cis*‐linked l‐FucNAc–linker motif, followed by regioselective glycosylation at O3 and subsequently at O2 of the d‐Glc–l‐FucNAc–linker disaccharide. The same strategy was applied for assembling its tetrasaccharide fragment. Additionally, *β*‐mannosylation and 1,2‐*cis*‐d‐FucNAc linkage formations were optimized for the *S. aureus* CP8 fragment, establishing a versatile route toward bacterial glycans relevant for vaccine and diagnostic development.

## Graphical abstract

A total of 16 chemically synthesized capsular polysaccharide (CPS) fragments related to *Pseudomonas aeruginosa*, *Staphylococcus aureus*, and *Acinetobacter baumannii* were analyzed by glycan microarray. Comparative screening revealed three conserved epitopes that act as cross‐protective vaccine lead candidates against multidrug‐resistant (MDR) bacterial pathogens.

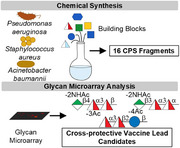

## Introduction

Multidrug‐resistant bacteria are one of the greatest threats to human health today, highlighted by the bacterial‐priority pathogens list issued by the World Health Organization (WHO).^[^ ^1^, ^2^, ^3^ ^]^ The Gram‐negative bacterium *Acinetobacter baumannii* is resistant to last‐generation antibiotics and has been categorized as a “critical‐priority” pathogen.^[^ ^4^ ^]^ Similarly, due to their resistance to last‐generation antibiotics, high transmissibility, and mortality rates, especially in healthcare facilities, *Pseudomonas aeruginosa* and *Staphylococcus aureus* are classified as “high‐priority” pathogens.^[^ ^2^, ^5^ ^]^ Improved diagnostics, prevention, and treatments are needed to control these bacteria.

*P. aeruginosa*, *S. aureus*, and *A. baumannii* are involved in a variety of polymicrobial infections and modulate each other's growth and resistance.^[^ ^6^, ^7^, ^8^, ^9^, ^10^, ^11^ ^]^ These three bacteria belong to a common group of pathogens called ESKAPE pathogens, composed of *Enterococcus faecium*, *S. aureus*, *Klebsiella pneumoniae*, *A. baumannii*, *P. aeruginosa*, and *Enterobacter spp*. They were initially identified as critical multidrug‐resistant (MDR) bacteria with an urgent need for effective treatments. These opportunistic bacteria share traits that allow them to thrive in healthcare environments, including a range of resistance mechanisms rendering them a leading cause of drug‐resistant infections.^[^ ^12^ ^]^

Glycoconjugate vaccines are a good option to prevent infections with pathogens.^[^ ^13^, ^14^, ^15^, ^16^ ^]^ A detailed understanding of the postinfection immune response is critical for vaccine development, particularly for the identification of glycan epitopes that may be involved in mediating protective or immunomodulatory effects. Glycan microarrays containing well‐defined, pure synthetic glycans provide a high‐throughput platform for the rapid analysis of serum antiglycan antibodies and the discovery of new cross‐species specific biomarkers.^[^ ^17^ ^]^ Synthetic access to pure, structurally‐defined bacterial glycotopes containing rare amino‐sugars, equipped with suitable linkers for protein conjugation is essential to enable biomarker discovery. Synthetic epitopes free of biological contaminants are valuable tools for immunological studies. Significant progress has been made in the chemical synthesis of bacterial glycoconjugates containing rare deoxy‐amino sugars.^[^ ^18^, ^19^ ^]^ A range of glycoconjugates, including the *K. pneumoniae* O1 and O2c antigens,^[^ ^20^ ^]^ *Streptococcus pneumoniae* capsular polysaccharides (CPS),^[^ ^21^ ^]^ and *Francisella tularensis* strain 15^[^ ^22^ ^]^ were explored using glycan microarrays.^[^ ^23^ ^]^

*P. aeruginosa* can cause severe and life‐threatening infections such as pneumonia and bloodstream infections. As an opportunistic pathogen, it poses a serious risk to hospitalized patients.^[^ ^24^, ^25^ ^]^ This Gram‐negative bacterium is especially dangerous for immunocompromised individuals and exhibits resistance to most antibiotics, including carbapenems, the few remaining treatment options for multidrug‐resistant infections. Additionally, *P. aeruginosa* plays a key role in the development of chronic lung infections in patients with cystic fibrosis,^[^ ^26^ ^]^ non‐cystic fibrosis bronchiectasis (NCFB) and chronic obstructive pulmonary disease (COPD).^[^ ^27^, ^28^ ^]^ For decades, vaccines and monoclonal antibodies have been explored for both active and passive immunization against *P. aeruginosa*. Following some promise in preclinical studies, only one vaccine candidate progressed to clinical trials, and none received approval. Currently, no effective vaccine or therapeutic is available for *P. aeruginosa*.^[^ ^12^ ^]^

Another “high‐priority” ESKAPE pathogen, *S. aureus* is a Gram‐positive bacterium that causes a range of infections, including skin and soft tissue infections as well as device‐related infections.^[^ ^29^ ^]^ It threatens vulnerable populations such as newborns, surgical patients, and immunocompromised individuals.^[^ ^30^ ^]^ Methicillin‐resistant *S. aureus* (MRSA) and vancomycin‐resistant *S. aureus* (VRSA) present a major public health threat.^[^ ^31^ ^]^ MRSA is resistant to most *β*‐lactams, including anti‐staphylococcal penicillins and cephalosporins,^[^ ^32^ ^]^ and cause approximately 10,600 deaths annually in the USA alone.^[^ ^33^ ^]^ Among the 12 known *S. aureus* CPS serotypes, serotypes 5 and 8 account for 85% of clinical isolates, making them attractive targets for vaccine development.^[^ ^34^ ^]^ A bivalent conjugate vaccine, StaphVAX was developed using CPS types 5 and 8 conjugated to a nontoxic recombinant form of *P. aeruginosa* exotoxin A and demonstrated protection against *S. aureus* bacteremia for up to ten months in a phase III clinical trial.^[^ ^31^, ^35^ ^]^ Despite the initial success, the vaccine failed to significantly reduce the incidence of invasive *S. aureus* infections.^[^ ^36^ ^]^ Vaccine efficacy needs to be improved, potentially by modulating the antigenic construct.^[^ ^37^ ^]^

**Figure 1:**
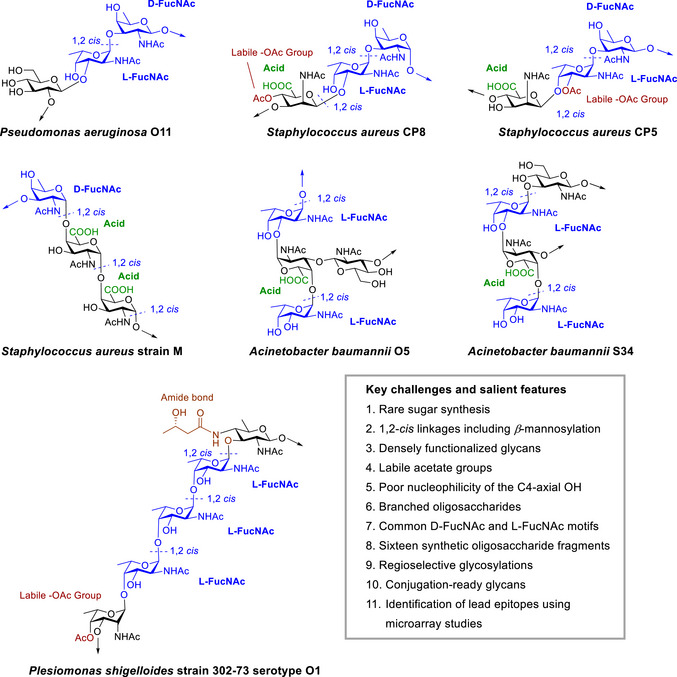
Structures of bacterial glycans of different strains of ESKAPE pathogens containing rare d‐FucNAc and l‐FucNAc sugars.

*A. baumannii*, a Gram‐negative coccobacillus^[^ ^38^, ^39^ ^]^ can be present on environmental surfaces, is resistant to many disinfectants and spreads within healthcare units.^[^ ^38^ ^]^ Like other ESKAPE pathogens, it is resistant toward a wide‐range of antibiotics. The CPS of *A. baumannii* covers the outer membrane and is composed of oligosaccharide units (K units) that serve as key virulence factors protecting the bacteria and helping it evade the host immune response.^[^ ^39^ ^]^ *A. baumannii* biofilms significantly reduce the effectiveness of antibiotics and combination therapies.^[^ ^40^, ^41^ ^]^ Vaccination offers a strategy to combat multidrug‐resistant strains. Developing a prophylactic vaccine targeting surface polysaccharides is well‐established,^[^ ^42^ ^]^ but despite extensive research, no effective vaccine has yet been developed for *P. aeruginosa*, *S. aureus*, or *A. baumannii*. These pathogens contain common rare‐amino sugar units, d‐ and l‐*N*‐acetyl‐fucosamine (FucNAc) (Figure 1). *P. aeruginosa* O11 and *S. aureus* (serotypes 5 and 8) exhibit striking structural similarities, sharing a common disaccharide unit that differs only in regio‐ and stereochemistry of the extended linkages. The trisaccharide repeating unit (RU) of *S. aureus* strain M also contains a d‐FucNAc unit. Likewise, the *A. baumannii* O5 and S34 RU contain the l‐FucNAc motif. The lipopolysaccharide of *Plesiomonas shigelloides* strain 302‐73 serotype O1 is comprised of three l‐FucNAc units linked together. These commonalities inspired our exploration into the chemical synthesis of glycan fragments and their analysis using glycan microarrays. Considering the pathogenicity and drug resistance of these bacteria, vaccine development targeting their glycan structures remains a promising strategy, and synthetic glycan fragments offer a powerful tool for probing immune responses to varied epitopes.

**Figure 2:**
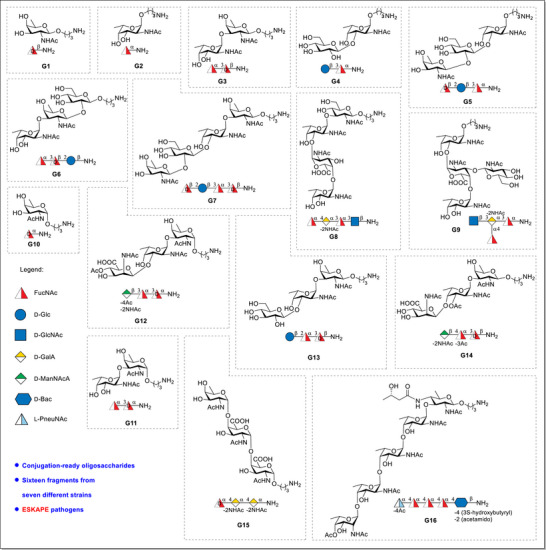
Structures of target molecules for microarray investigation.

Here, we report the synthesis of a series of glycans (**G1–G7** and **G10–G12**) related to the ESKAPE pathogens: *P. aeruginosa* O11, *S. aureus* strains CP5, CP8, and strain M, and *A. baumannii* S34 and O5 as well as *P. shigelloides* strain 302‐73 (serotype O1) followed by comparative glycan microarray analyses of **G1**–**G16** (Figure 2 and Table 1). The total syntheses of oligosaccharide RUs from *A. baumannii* S34 and O5, *P. aeruginosa* O11, *S. aureus* CP5, CP8 and strain M, and *P. shigelloides* serotype O1 including **G8**, **G9**, and **G13‐** **G16** were accomplished previously,^[^ ^43^, ^44^, ^45^, ^46^, ^47^, ^48^, ^49^, ^50^, ^51^, ^52^, ^53^ ^]^ but a systematic microarray‐based comparison of these glycotopes was not performed. Our syntheses focused on the glycan fragments: mono‐(**G1, G2, G10**), di‐(**G3, G4, G11**), tri‐(**G5, G6, G12**), and tetrasaccharide (**G7**) derivatives. Glycans **G8**, **G9,** and **G13–G16** were synthesized earlier.^[^ ^43^, ^44^, ^45^, ^46^, ^47^ ^]^

**Table 1:** Glycan fragments found in different pathogens.

Pathogens	Glycan fragments
*P. aeruginosa* O11	G1–G7 and G13
*S. aureus* type 8	G10–G12
*S. aureus* type 5	G1, G3, and G14
*S. aureus* strain M	G10 and G15
*A. baumannii* S34	G2 and G8
*A. baumannii* O5	G2 and G9
*P. shigelloides* serotype O1	G2 and G16

The trisaccharide RU of *P. aeruginosa* O11, comprised of →2)‐*β*‐d‐Glc‐(1→3)‐*α*‐l‐FucNAc‐(1→3)‐*β*‐d‐FucNAc‐(1→, a potential vaccine candidate^[^ ^54^, ^55^ ^]^ and the RU of *S. aureus* CP8 →3)‐*β*‐d‐ManNAcA(4OAc)‐(1→3)‐*α*‐l‐FucNAc‐(1→3)‐*α*‐d‐FucNAc‐(1→^[^ ^56^ ^]^ share a common disaccharide core, differing only in the stereochemistry at their reducing‐end linkages. The syntheses of these glycans pose challenges including rare sugars, 1,2 *cis*‐linkages including *β*‐mannosylation and stereoselective linker installation. Additional complexities were encountered in preserving the *O*‐acetyl substituent at the C4 position of d‐ManNAcA and the incorporation of carboxylates and acetamides in the *S. aureus* CP8 glycans. The glycan syntheses established a robust platform for accessing diverse glycotopes and enable high‐throughput glycan microarray analyses.

## Results and Discussion

**Scheme 1:**
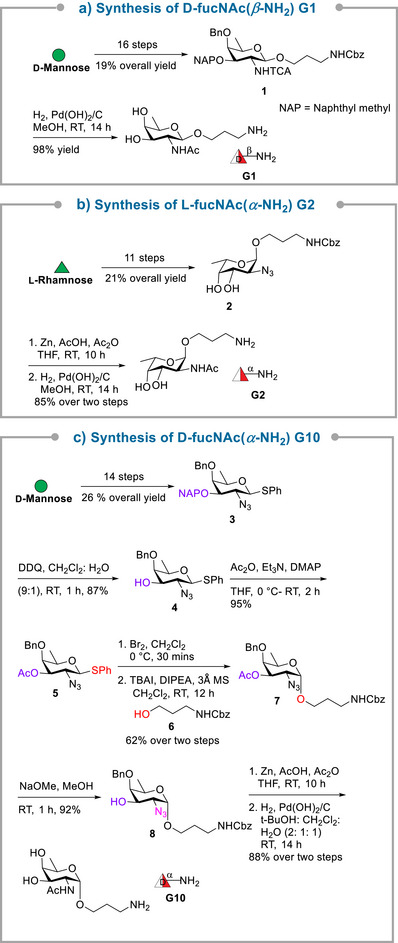
Synthesis of linker appended monosaccharide glycans **G1** (a), **G2** (b), and **G10** (c).

The synthesis of glycan epitopes **G1–G7** and **G10–G12** began with readily available monosaccharides, including d‐mannose, l‐rhamnose, d‐glucose, and *α*‐methoxy d‐glucose, which were elaborated into FucNAc, Glc, and ManNAcA derivatives. The assembly strategy was designed to enable precise installation of *α*‐ and *β*‐glycosidic linkages while maintaining functional groups for subsequent conjugation. The synthesis of three monosaccharide sugar units (**G1**, **G2**, and **G10**) is outlined in Scheme 1. The preparation of intermediate **1** from d‐mannose involved a 16‐step reaction sequence to obtain the desired product in 19% overall yield.^[^ ^43^ ^]^ Subsequent hydrogenolysis of the protected d‐fucosamine derivative **1** facilitated the global deprotection and simultaneous conversion of NHTCA to acetamide (NHAc) groups, affording target epitope **G1** in 98% yield. Further, l‐fucosamine derivative **2** was accessed from l‐rhamnose through an established 11‐step protocol^[^ ^43^, ^45^ ^]^ affording the desired product in 21% overall yield. The C2‐azide was then converted to an acetamide group via zinc‐mediated reduction and acetylation in same pot, followed by hydrogenolysis, providing target epitope **G2** in 85% yield over two steps. The synthesis of *α*‐linked d‐fucosamine precursor **3** was accomplished via an established protocol involving a 14‐step reaction sequence, starting from d‐mannose with an overall 26% yield.^[^ ^43^ ^]^ Oxidative cleavage of 2‐naphthylmethyl (NAP) group in **3** using DDQ afforded **4** in 87% yield which upon subsequent acetylation using acetic anhydride furnished 95% yield of **5**. Attempts at the direct glycosylation of highly reactive linker acceptor **6** using donors **3** and **5** and NIS/TfOH or NIS/TMSOTf activation in diethylether as a participating solvent furnished *α*/*β* mixtures of d‐fucosamine glycoside. Failure to obtain exclusive *α*‐selectivity prompted attempts at S*_N_* 2 glycosylations employing glycosyl halide‐mediated in‐situ anomerization. To that end, the NAP ether protecting group was replaced with an electron‐withdrawing acetyl ester to ensure the stability of the bromo intermediate required for stereoselective coupling with 3‐aminopropyl linker **6**. This stereoselective glycosyl‐halide mediated S*_N_* 2 reaction^[^ ^57^ ^]^ afforded d‐fucosamine derivative **7** in 62% yield over two steps. Here, TBAI generates a *β *‐glycosyl iodide intermediate via in situ halide exchange, forcing the nucleophile to attack exclusively from the bottom face and thereby delivering exclusively *α*−glycoside.^[^ ^58^, ^59^ ^]^ De‐esterification of **7** and azide to acetamide conversion followed by hydrogenolysis using a solvent mixture (tBuOH:CH_2_Cl_2_:H_2_O in 2:1:1 ratio), furnished **G10** in 88% yield.

**Scheme 2:**
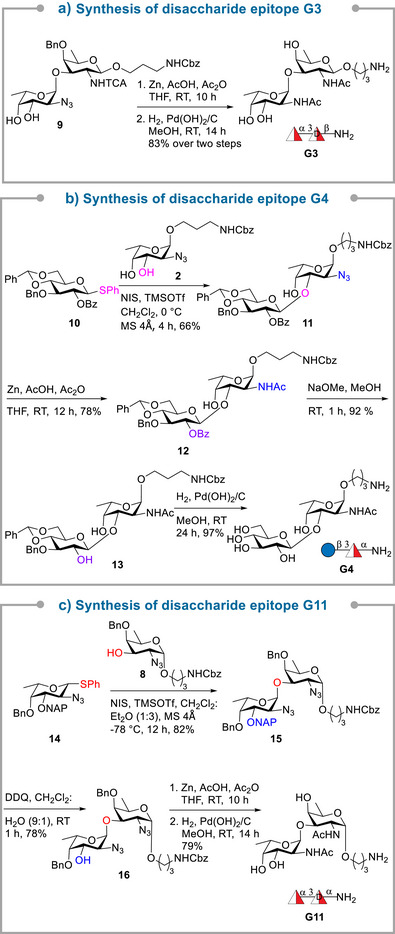
Synthesis of the disaccharide fragments from *P. aeruginosa* O11 and *S. aureus* CP5 and CP8. Synthesis of **G3** (a), **G4** (b) and **G11** (c).

The syntheses of disaccharide epitopes **G3**, **G4,** and **G11** started with disaccharide diol **9**, glucose **10** and l‐fucosamine **14**, respectively.^[^ ^43^ ^]^ For the synthesis of **G3**, the azide and NHTCA groups were first converted to acetamide using zinc, acetic acid, and acetic anhydride, followed by hydrogenolysis, to furnish the desired disaccharide epitope in 83% yield. The synthesis of **G4**, relied on the regioselective union of glucose donor **10** and l‐fucosamine diol acceptor **2** ^[^ ^44^ ^]^ in the presence of NIS/TMSOTf at 0 °C to afford O3‐linked disaccharide **11** in 66% yield, as a single isomer (Scheme 2). The regioselectivity of disaccharide **11** was confirmed by acetylation of the free C4‐hydroxy group to afford compound **S1** (see Supporting Information). A doublet of H4 at *δ* 5.23 ppm with *J* = 2.9 Hz in ^1^H NMR spectrum correlating with C4 at *δ* 68.6 ppm in ^1^H‐^13^C HSQC NMR spectrum, and correlation of ‐CH_3_ proton with H5, and consecutively H5 with H4, and H4 with H3 in the ^1^H − ^1^H COSY spectrum along with HMBC correlations confirmed the presence of the acetyl group at the C‐4 position and eventually the glycosidic bond at C‐3 of **S1**. The transformation of the azide into an acetamido group using zinc‐mediated reduction followed by acetylation furnished **12** in 78% yield. Finally, debenzoylation of **12** using NaOMe in MeOH gave diol **13** in 92% yield, before hydrogenolysis furnished disaccharide epitope **G4** in 97% yield. Disaccharide **G11** was prepared by coupling l‐fucosamine donor **14** ^[^ ^44^ ^]^ with d‐fucosamine acceptor **8** and NIS/TMSOTf using solvent participation, (CH_2_Cl_2_:Et_2_O; 1:3) at −78 °C furnished 1,2‐*cis* linked disaccharide **15** as a sole isomer in 82% yield. Oxidative cleavage of the NAP ether afforded disaccharide **16** in 78% yield. Azide to acetamide conversion followed by hydrogenolysis afforded disaccharide epitope **G11** in 79% yield.

**Scheme 3:**
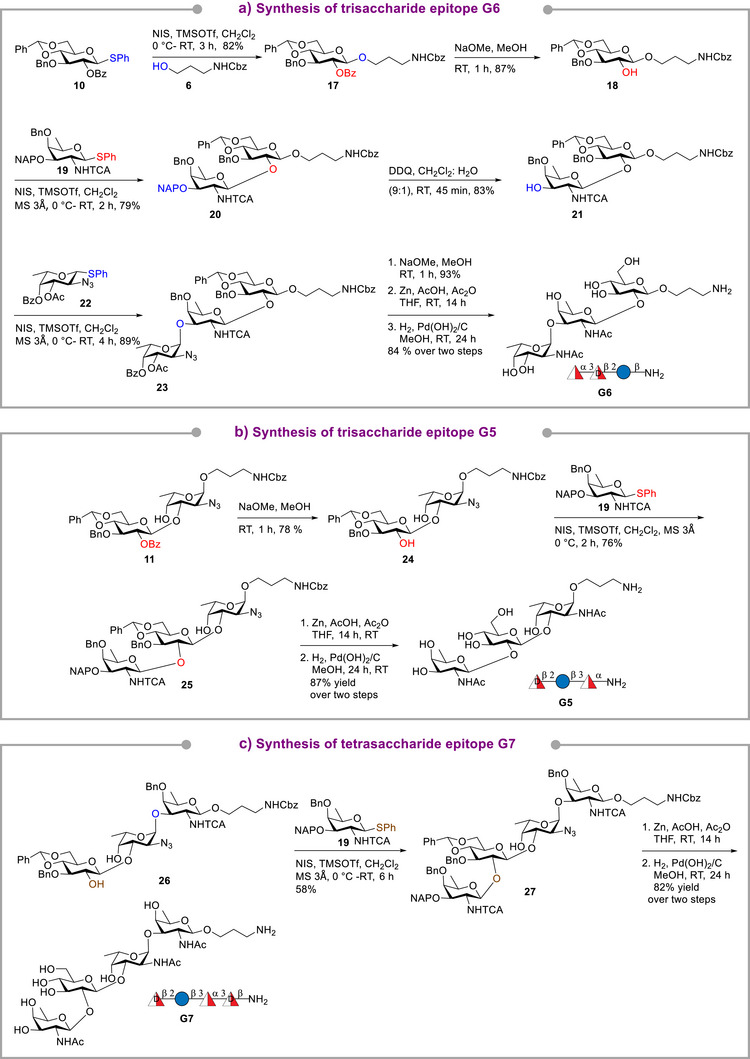
Synthesis of trisaccharides **G6** (a), **G5** (b) and tetrasaccharide **G7** (c) of *P. aeruginosa* O11.

The synthesis of tri‐ (**G5** and **G6**) and tetrasaccharide (**G7**) epitopes of *P. aeruginosa* O11 (Scheme 3) started with coupling of the aminopropyl linker with glucose **10** followed by debenzoylation to furnish 87% yield of acceptor **18**. Utilizing NHTCA neighboring group participation in d‐fucosamine**19** ^[^ ^43^ ^]^ ensured selective glycosylation of **18** to give *β*‐linked disaccharide **20** in 79% yield. Oxidative cleavage of the 2‐naphthylmethyl group in **20** using DDQ in CH_2_Cl_2_:H_2_O (9:1) afforded disaccharide acceptor **21** in 83% yield. Utilizing long‐range participation via C4‐OBz in donor **22**, *α*‐stereoselective glycosylation of disaccharide acceptor **21** yielded exclusively fully functionalized trisaccharide **23** in 89% yield. De‐esterification of trisaccharide **23**, afforded 93% of the corresponding diol, followed by conversion of the azide and NHTCA groups to acetamido and hydrogenolysis using Pd(OH)_2_/C in methanol yielded trisaccharide glycotope **G6** in 84% yield. (Scheme 3a). For the synthesis of **G5**, debenzoylation of **11** gave disaccharide acceptor **24** in 78% yield. Regioselective coupling of the equatorial C2‐OH in **24** with donor **19** in the presence of NIS/TMSOTf afforded the 1,2‐*trans* O2 coupled trisaccharide **25** in 76% yield. Azide and NHTCA were converted to NHAc, followed by hydrogenolysis to furnish trisaccharide epitope **G5** in 87% yield (Scheme 3b). The synthesis of tetrasaccharide **G7** started with a regioselective 1,2‐*trans* coupling of trisaccharide diol **26** with donor **19** at 0 °C to afford tetrasaccharide **27** in 58% yield. Treatment of the protected tetrasaccharide with Zn, AcOH, Ac_2_O and hydrogenolysis delivered desired epitope **G7** in 82% yield (Scheme 3c). The position of glycosidic bonds in oligosaccharides such as **G5** and **G7** were ascertained by extensive 2D NMR experiments (see Supporting Information).

**Scheme 4:**
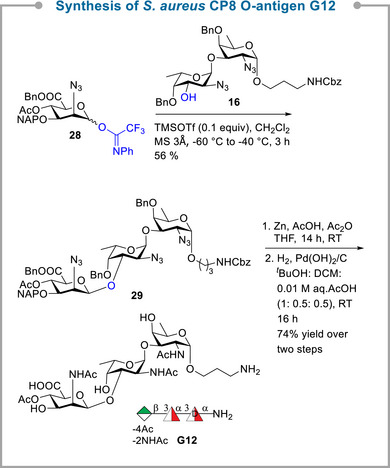
Synthesis of *S. aureus* CP8 trisaccharide RU **G12**.

The synthesis of the *S. aureus* type 8 trisaccharide epitope **G12** commenced with the coupling of trifluoroacetimidate donor **28** ^[^ ^44^ ^]^ and disaccharide acceptor **16**. Earlier, we reported the synthesis of a trisaccharide RU of CP8 bearing a PMP group at the reducing end, wherein the installation of a similar *β*‐mannosidic linkage was achieved using excess glycosyl donor at low temperature. Since a linker is necessary for microarray studies, glycosylation of the linker‐appended disaccharide acceptor **16** (Scheme 2c) was attempted at −60 °C using TfOH (0.5 equiv) as a promoter. The reaction proceeded in low yield with a lots of unreacted acceptor **16** remaining. Conducting the reaction −60 °C using TMSOTf (0.1 equiv) while slowly increasing the temperature to −40 °C over 3 h, gave 56% of trisaccharide **29** (Scheme 4). This reaction is believed to proceed via a half‐chair oxocarbenium intermediate in ^3^ *H*_4_ confirmation with the benzylic ester participating to enforce *β*‐selectivity.^[^ ^60^, ^61^, ^62^ ^]^ *β*‐Stereoselectivity was confirmed by *J*_CH_ coupling experiments (*J*_C1−H1_ = 162.2 Hz) (see Supporting Information). Using limited amounts of promoter (0.1 equiv) and excess donor (four equivalents) proved crucial for achieving the desired *β*‐mannosidic linkage, as excess promoter led to TMS‐protection of the hydroxyl groups. Subsequent reduction of the azides in fully protected trisaccharide **29** to amines followed by acetylation furnished the corresponding NHAc intermediate. Surprisingly, the acetate group at C‐4 was found to be highly labile, migrating and/or cleaving under certain conditions (PhSH/Et_3_N or hydrogenolysis in methanol). We believe that the free amino group in the linker may promote acetate migration or partial deacetylation, particularly during prolonged hydrogenation in methanol. Hydrogenolysis in a solvent mixture of tBuOH:CH_2_Cl_2_:0.01 M aq. AcOH (1:0.5:0.5) and Pearlman's catalyst for 16 h afforded the final *S. aureus* CP8 epitope **G12** in 74% yield over two steps.

**Figure 3:**
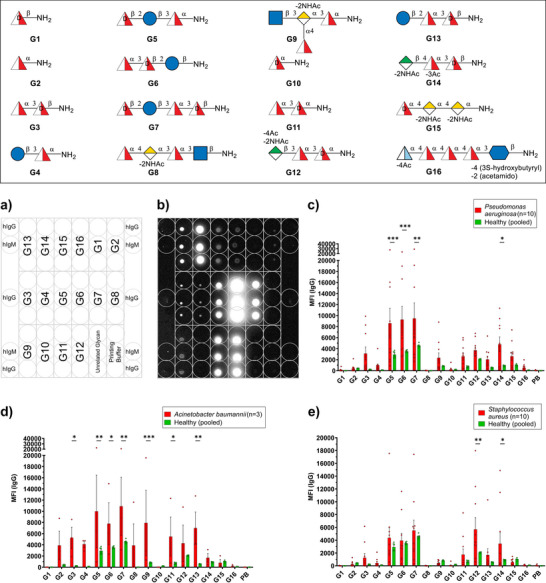
Glycan microarray analysis of sera derived from patients infected with *P. aeruginosa*, *A. baumannii* or *S. aureus*. Glycan microarray printing pattern (a) and exemplary binding pattern of one human serum to immobilized synthetic glycans (b). Mean fluorescence intensity (MFI) of IgG antibody binding to synthetic glycans in patients infected with *P. aeruginosa* (c), *A. baumannii* (d), or *S. aureus* (e). A serum dilution of 1:100 was used. Values represent mean ± SEM. Differences were tested for significance to pooled sera of healthy individuals using multiple Two‐Way ANOVA test with (***) *p* < 0.0002, (**) *p* < 0.002 and (*) *p* < 0.033.

The synthetic glycans were immobilized in triplicates on *N*‐hydroxy succinimide (NHS)‐activated glass slides to detect binding of IgG antibodies in sera of patients infected with *P. aeruginosa*, *A. baumannii*, or *S. aureus* (Figure 3). Patients infected with *P. aeruginosa* showed a higher IgG antibody level against the *P. aeruginosa* strain O11‐related **G5**, **G6,** and **G7** compared to healthy individuals. IgG antibodies against the *S. aureus* type 5 related **G14** were detected in addition. IgG antibodies directed toward *A. baumannii* strain O5‐related glycan **G9** were present in the sera of patients infected with *A. baumannii*. Furthermore, *P. aeruginosa* strain O11‐related **G3**, **G5**, **G6**, **G7,** and **G13** and *S. aureus* CP8‐related **G11** were epitopes for IgG antibody binding. All these structures contain both l‐FucNAc and d‐FucNAc monosaccharides. *S. aureus* infected patients showed IgG antibodies against the *S. aureus* CP8‐related **G12** and *S. aureus* type 5‐related **G14** and no binding toward **G11**, leading to the conclusions that the trisaccharide is the minimal epitope and the d‐FucNAc glycosidic linkage with linker is of no significance. Since current diagnostic tools do not differentiate between strains of bacterial infections, not all patients showed antibodies toward the bacteria‐related glycans. IgG antibodies directed against **G5**, **G6**, **G7,** and **G12** were detected in healthy patients, potentially a result of previous infections with related bacterial species. The microarray results show that although the trisaccharide is the minimal epitope, the sequence and stereochemistry of the d‐Glc, l‐FucNAc, and d‐FucNAc residues have an influence on antibody binding. Glycans **G5**, **G6,** and **G13** contain the same monosaccharides but differ in connectivity, resulting in different immunogenicity. These subtle topological differences explain why **G6**, and not the closely related **G13**, emerged as the lead structure together with **G12** and **G14**.

## Conclusion

We synthesized glycans that structurally mimic *O*‐antigens and CPS belonging to three pathogens from the ESKAPE family, *P. aeruginosa* O11, *S. aureus* (CP5, CP8, and strain M), *A. baumannii* (S34 and O5), as well as *P. shigelloides* O1. These glycans share rare deoxy amino sugars, notably d‐fucosamine and l‐fucosamine. Our synthetic route to the target glycans relied on regio‐ and stereoselective glycosylations to avoid extensive protection/deprotection sequences. The formation of the requisite 1,2‐*cis* glycosidic linkages was controlled by leveraging both solvent and long‐range participation effects. The terminal aminopropyl linker was installed onto the d‐FucNAc moiety, via in situ anomerization protocol involving a bromo intermediate, to furnish exclusively the desired α‐linked product. A direct *β*‐mannosylation employing a *N*‐phenyl‐2,2,2‐trifluoroacetimidoyl chloride donor with a rare disaccharide acceptor facilitated expedient access to a conjugation‐ready trisaccharide repeating unit of *S. aureus* CP8, a promising vaccine candidate.

A total of sixteen glycans were evaluated in order to get insights into the antibody production after the infection with *P. aeruginosa*, *S. aureus* or *A. baumannii* to define key immunogenic epitopes. Glycan microarray analysis of sera derived from patients after infection with *P. aeruginosa*, *S. aureus* or *A. baumannii* showed significantly higher antibody levels against the trisaccharides **G14**, **G6,** and **G12** compared to the level in healthy humans. Similar glycan composition across different strains of bacteria renders synthetic trisaccharides **G14**, **G6,** and **G12** a promising option for cross‐protective immunization against all three bacteria.

Future investigations will focus on detailed antigen–antibody binding studies to uncover molecular recognition patterns, along with animal immunization experiments to assess protective efficacy. This synthetic platform broadens access to rare‐sugar‐containing bacterial glycans for next‐generation carbohydrate‐based vaccines and diagnostics tools.

## Conflict of Interests

The authors declare no conflict of interest.

## Supporting information



Supporting Information

## Data Availability

The data that support the findings of this study are available in the Supporting Information of this article.
