# Supplementary material for: Chemical Synthesis of Pseudomonas aeruginosa, Staphylococcus aureus, and Acinetobacter baumannii Capsular Polysaccharide Fragments as Leads for Cross‐Protection
Source: Angew Chem Int Ed Engl. 2025 Dec 21;65(6):e24231. doi: 10.1002/anie.202524231 (PMC12865246; doi:10.1002/anie.202524231)
Supplement: Supplementary file 1 — Supporting Information [file ANIE-65-e24231-s001.pdf]

Supporting Information

Chemical Synthesis of *Pseudomonas aeruginosa*, *Staphylococcus aureus* and *Acinetobacter baumannii* Capsular Polysaccharide Fragments as Leads for Cross-Protection

Amar Kumar Mishra,[1] Emelie E. Reuber,[2,3] Diksha Rai,[1] Leif E. Sander,[4,5,6] Julia Duerr[5,7,8], Simon Y. Graeber[5,7,8], Marcus A. Mall[5,7,8,9], Bettina C. Fries,[10,11,12] Peter H. Seeberger,[2,3*] Suvarn S. Kulkarni[1*]

[1] Department of Chemistry, Indian Institute of Technology Bombay, Mumbai 400076, India.

[2] Institute of Chemistry and Biochemistry, Freie Universität Berlin; Berlin, Germany.

[3] Max Planck Institute of Colloids and Interfaces; Potsdam, Germany.

[4] Department of Infectious Diseases and Critical Care Medicine, Campus Virchow-Klinikum and Campus Charité Mitte, Charité - Universitätsmedizin Berlin, Corporate Member of Freie Universität and Humboldt-Universität zu Berlin; Berlin, Germany.

[5] German Center for Lung Research (DZL), Associated Partner Site Berlin, Berlin, Germany.

[6] Berlin Institute of Health at Charité - Universitätsmedizin Berlin; Berlin, Germany.

[7]Department of Pediatric Respiratory Medicine, Immunology and Critical Care Medicine, Charité-Universitätsmedizin Berlin, Berlin, Germany

[8]German Center for Child and Adolescent Health (DZKJ), Partner Site Berlin, Berlin, Germany

[9]Cluster of Excellence ImmunoPreCept, Charité - Universitätsmedizin Berlin, Berlin, Germany

[10] Department of Microbiology and Immunology, Renaissance School of Medicine, Stony Brook University, Stony Brook, NY 11794 USA.

[11] Division of Infectious Diseases, Department of Medicine, Stony Brook University, Stony Brook, NY 11794 USA.

[12] Veterans Administration Medical Center, Northport, NY 11768 USA.

*Corresponding authors: Suvarn S. Kulkarni and Peter H. Seeberger

Email: suvarn@chem.iitb.ac.in

peter.seeberger@mpikg.mpg.de

Table of Contents

I. Experimental Procedure

General Information S4

Synthesis of compound **G1** S5

Synthesis of compound **G2** S5

Synthesis of compound **4** S6

Synthesis of compound **5** S7

Synthesis of compound **7** S8

Synthesis of compound **8** S9

Synthesis of compound **G10** S9

Synthesis of compound **G3** S10

Synthesis of compound **11** S11

Synthesis of compound **S1** S12

Synthesis of compound **12** S13

Synthesis of compound **13** S14

Synthesis of compound **G4** S15

Synthesis of compound **15** S16

Synthesis of compound **16** S17

Synthesis of compound **G11** S18

Synthesis of compound **17** S18

Synthesis of compound **18** S19

Synthesis of compound **20** S20

Synthesis of compound **21** ………………………………………… S21

Synthesis of compound **23** ………………………………………… S22

Synthesis of compound **G6** ……………………………………….. S23

Synthesis of compound **24** ……………………………………….. S24

Synthesis of compound **25** ……………………………………….. S25

Synthesis of compound **G5** ……………………………………….. S26

Synthesis of compound **27** ……………………………………….. S27

Synthesis of compound **G7** ……………………………………….. S28

Synthesis of compound **29** ……………………………………….. S29

Synthesis of compound **G12** ……………………………………….. S30

Glycan Microarray ……………………………………….. S31

Study participant details ……………………………………….. S32

Data Analysis ……………………………………….. S32

II. References S33

III. Spectra S34

EXPERIMENTAL SECTION:

General Methods. All reactions were conducted under the dry nitrogen atmosphere. Solvents (CH_2Cl_2 >99%, THF 99.5%, acetonitrile 99.8%, DMF 99.5%) were purchased in capped bottles and dried under sodium or CaH_2. All other solvents and reagents were used without further purification. All glassware used was oven dried before use. TLC was performed on pre-coated Aluminium plates of Silica Gel 60 F254 (0.25 mm, E. Merck). Developed TLC plates were visualized under a short-wave UV lamp and by heating plates that were dipped in ammonium molybdate/cerium (IV) sulfate solution. Silica gel column chromatography was performed using Silica Gel (100-200 mesh) and employed a solvent polarity correlated with TLC mobility. We have used 3 Angstrom powdered molecular sieves in our study. The powdered MS was weighed in a dried pear-shaped flask and activated by periodic heating using a flame for over 15 minutes. Reactions under heating conditions were performed using a paraffin oil bath.

NMR experiments were conducted on 600, 500, and 400 MHz instruments using $CDCl_3$ (D, 99.8%) or D_2O (D, 99.9%) as solvents. Chemical shifts are relative to the deuterated solvent peaks and are in parts per million (ppm). ^1H-^1H COSY, ^1H-^{13}C HSQC, ^1H-^{13}C HMBC and 2D TOCSY were used to confirm proton assignments. Mass spectra were acquired in the ESI mode. Melting points were determined by capillary apparatus. Specific rotation experiments were measured at 589 nm (Na) and 25 °C. IR spectra were recorded on an FT-IR spectrometer.

Compound G1:

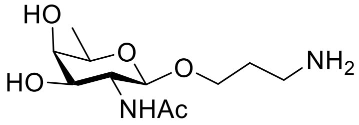

G1

To a solution of compound **1** (26 mg, 0.026 mmol) in methanol (3 mL), $Pd(OH)_2/C$ (80 mg) at room temperature was added. The reaction mixture was then stirred under a hydrogen atmosphere (1 atm) for 14 h. After the reaction was completed, the crude mixture was filtered through Celite with methanol as eluent, concentrated, and dried under a high vacuum to obtain **G1** (9.1 mg, 98%) as a colourless sticky solid.

$[\alpha]_D^{25}$ = -1.310 (*c* = 0.170, MeOH).

IR (cm^{-1}, $CHCl_3$) ***ν*** = 3694, 3358, 3109, 1716, 1575, 1356, 1122, 998, 847.

^{1}H NMR (400 MHz, D_2O) ***δ*** 4.28 (d, *J* = 8.4 Hz, 1H, H-1), 3.87- 3.85 (m, 1H), 3.75- 3.58 (m, 5H), 2.96 (t, *J* = 6.6 Hz, 2H, linker -NCH_2), 1.92 (s, 3H, -NHAc), 1.83- 1.76 (m, 2H, linker -CH_2), 1.14 (d, *J* = 6.20 Hz, 3H, -CH_3).

^{13}C{^{1}H} NMR (100 MHz, D_2O) ***δ*** 174.9, 101.6, 70.90, 70.85, 70.4, 68.0, 52.1, 37.8, 26.6, 22.2, 15.4

HRMS (ESI-TOF) (m/z) calculated for $C_{11}H_{22}N_2O_5Na$ $[M+Na]^+$: 285.1421, found 285.1416.

Compound G2:

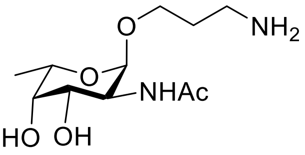

G2

Activated zinc dust (100 mg), acetic anhydride (0.08 mL) and acetic acid (0.1 mL) were added sequentially to the stirring solution compound **2** (38 mg, 0.099 mmol) in THF (2 mL). The mixture was stirred overnight at room temperature. After completion of reaction (indicated by TLC), the reaction mixture was diluted with ethyl acetate and zinc was filtered out through celite pad, then the liquor was directly concentrated under reduced pressure. Toluene azeotrope

was performed to remove the remaining acetic acid, purified by column chromatography on silica gel (1:9 methanol: ethyl acetate) to obtain NHAc compound as a colourless foam and kept under high vacuum for the next step. The obtained amide compound was dissolved in methanol (4 mL). To this solution, $Pd(OH)_2/C$ (80 mg) was added and stirred under hydrogen atmosphere (1 atm, balloon) for 14 h. The reaction mixture was then filtered through Celite, concentrated, dried under high vacuum to obtain **G2** (22 mg, 85%) as a colourless sticky solid.

$[\alpha]_D^{25}$ = -26.426 (*c* = 0.170, MeOH).

IR (cm^{-1}, $CHCl_3$) *ν* = 3428, 1642, 1130, 1016, 846, 601.

^{1}H NMR (400 MHz, D_2O) *δ* 4.72 (1H, H-1), 3.99-3.93 (m, 2H), 3.80-3.77 (m, 1H), 3.67- 3.57 (brs, m, 2H), 3.40-3.21 (m, 2H), 2.94 (t, *J* = 7.3 Hz, 1H), 1.90 (s, 3H), 1.82- 1.70 (m, 2H), 1.10 (d, *J* = 5.24 Hz, 3H, $-CH_3$).

$^{13}C\{^1H\}$ NMR (100 MHz, D_2O) *δ* 174.5, 97.0, 71.0, 67.6, 66.6, 65.1, 49.6, 37.2, 27.1, 21.9, 15.4.

HRMS (ESI-TOF) (m/z) calculated for $C_{11}H_{23}N_2O_5$ $[M+H]^+$: 263.1601, found 263.1598.

Compound 4:

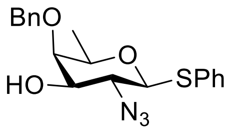

4

DDQ (0.59 g, 2.59 mmol) was added to a stirred solution of **3** (0.44 g, 0.86 mmol) in CH_2Cl_2 (10 mL) and H_2O (1 mL) at rt, and the reaction mixture was allowed to stir for 1 h. After completion of the reaction, the reaction mixture was quenched with Et_3N, the reaction mixture was diluted with CH_2Cl_2 (2×30 mL), and washed with saturated sodium bicarbonate solution. The separated organic layer was dried over anhydrous Na_2SO_4 and concentrated in vacuo. The crude product was purified by column chromatography on silica gel 2:8 ethyl acetate: pet ether) to obtain **4** as a pale-yellow viscous liquid (0.32 g, 87%).

$[\alpha]_D^{25}$ = +1.781 (*c* = 0.585, $CHCl_3$).

IR (cm^{-1}, $CHCl_3$) *ν* = 2924, 1658, 1466, 1045, 765.

^{1}H NMR (400 MHz, $CDCl_3$) ***δ*** 7.63- 7.60 (m, 2H), 7.39- 7.25 (m, 8H), 4.75 (q, *J* = 13.9 Hz, 2H), 4.40 (d, *J* = 9.7 Hz, 1H, H-1), 3.61- 3.51 (m, 4H, H-5, H-4, H3, H-2), 2.42 (brs, 1H, -OH), 1.37 (d, *J* = 6.5 Hz, 3H, $-CH_3$).

^{13}C{^{1}H} NMR (100 MHz, $CDCl_3$) ***δ*** 138.0, 132.8, 132.1, 128.9, 128.6, 128.0, 127.9, 127.7, 86.3, 78.7, 75.8, 75.0, 74.6, 63.2, 17.4.

HRMS (ESI-TOF) (m/z) calculated for $C_{19}H_{25}N_4O_3S$ $[M+NH_4]^+$: 389.1642, found: 389.1642.

Compound 5:

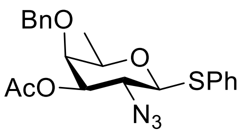

5

Compound **4** (0.30 g, 0.82 mmol) was dissolved in THF (5 mL) and Et_3N (0.22 mL), to this stirred solution, Ac_2O (0.11 mL) was added in a dropwise manner at 0 °C and the reaction mixture was kept stirring for 30 min. After completion of the reaction (confirmed by TLC) organic layer was washed with 1N HCl (15 mL) and brine solution, dried over anhydrous Na_2SO_4, organic layer was concentrated, and purified by column chromatography on silica gel (1:9 ethyl acetate: pet ether) to obtain **5** as a sticky solid (0.32 g, 95%).

$[\alpha]_D^{25}$ = +2.00 (*c* = 0.605, $CHCl_3$).

IR (cm^{-1}, $CHCl_3$) ***ν*** = 3019, 2926, 2855, 2114, 1745, 1463, 1215, 1047, 757, 669.

^{1}H NMR (400 MHz, $CDCl_3$) ***δ*** 7.63- 7.61 (m, 2H), 7.39- 7.24 (m, 8H), 4.80 (dd, *J* = 10.3, 2.9 Hz, 1H, H-3), 4.63 (q, *J* = 14.6 Hz, 2H, $PhCH_2$), 4.46 (d, *J* = 10.0 Hz, H-1), 3.85 (t, *J* = 10.1 Hz, 1H, H-2), 3.71 (d, *J* = 2.7 Hz, 1H, H-4), 3.64 (q, *J* = 6.4 Hz, 1H, H-5), 2.05 (s, 3H), 1.30 (d, *J* = 6.4 Hz, 3H, $-CH_3$).

^{13}C{^{1}H} NMR (100 MHz, $CDCl_3$) ***δ*** 170.3, 137.9, 133.0, 131.8, 128.9, 128.4, 128.0, 127.8, 127.7, 86.3, 76.3, 76.2, 75.4, 74.7, 59.5, 20.9, 17.1.

HRMS (ESI-TOF) (m/z) calculated for $C_{21}H_{27}N_4O_4S$ $[M+NH_4]^+$: 431.1748, found 431.1750.

Compound 7:

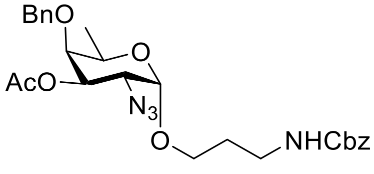

7

D-fucosamine donor **5** (0.326 g, 0.79 mmol) was dissolved in CH_2Cl_2 (4 mL) and Br_2 (0.12 mL, 2.36 mmol) was added under nitrogen atmosphere. After half an hour, TLC was checked, which showed the complete conversion of thiol donor into bromo intermediate. After that, toluene (2 mL) was added and the azeotropic mixture was evaporated under reduced pressure and kept under high vacuum for 15 mins. This crude bromo compound was further dissolved into CH_2Cl_2 (6 mL) and it was added into stirred solution containing benzyl 3- hydroxypropyl carbamate linker **6** (0.197 g, 0.94 mmol), tetrabutylammonium iodide (0.475 g, 2.36 mmol), DIPEA (0.13 mL, 0.78 mmol) and molecular sieves 3Å (0.2 g) at rt. Reaction was stirred for 12 h, and then monitored by TLC. After completion of the reaction, the mixture was filtered off using a celite bed, the filtrate was concentrated and purified by column chromatography over silica gel (20% ethyl acetate: pet ether) to afford the desired linker appended product **7** as a pale-yellow liquid (0.251 g, 62%). The challenges 1,2 cis-linkage in D-fucosamine with highly reactive primary -OH of the linker was achieved via glycosyl bromides intermediates that proceed through an S_N2 pathway.[1]

$[\alpha]_D^{25}$ = +5.762 (c = 0.585, $CHCl_3$).

IR (cm^{-1}, $CHCl_3$) ν = 2921, 1734, 763.

^{1}H NMR (400 MHz, $CDCl_3$) δ 7.35- 7.28 (m, 10H), 5.23 (dd, J = 11.9, 2.9 Hz, 1H, H-3), 5.09 (s, 2H, -NHCbz), 4.88 (d, J = 3.5 Hz, 1H, H-1), 4.67 (d, J = 11.5 Hz, 1H, PhCH), 4.57 (d, J = 11.5 Hz, 1H, PhCH), 3.97 (q, J = 6.3 Hz, 1H, H-5), 3.85 (dd, J = 11.0, 3.4 Hz, 1H, H-2), 3.81- 3.75 (m, 2H, H-4, OCH-Linker), 3.52- 3.46 (m, 1H, -OCH-Linker), 3.38- 3.28 (m, 2H, -NCH_2-Linker), 2.05 (s, 3H, -OAc), 1.84- 1.79 (m, 2H, -CH_2-Linker), 1.17 (d, J = 6.5 Hz, 3H, -CH_3).

$^{13}C\{^1H\}$ NMR (100 MHz, $CDCl_3$) δ 170.4, 156.5, 137.7, 128.49, 128.47, 128.2, 128.1, 128.0, 98.0, 77.2, 75.7, 72.1, 66.64, 66.57, 66.4, 57.9, 39.0, 29.2, 20.9, 16.4.

HRMS (ESI-TOF) (m/z) calculated for $C_{26}H_{33}N_4O_7$ $[M+H]^+$: 513.2344, found 513.2347.

Compound 8:

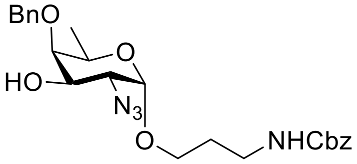

8

0.2M NaOMe (43 mg) was added to a stirred solution of **7** (0.226 g, 0.44 mmol) in MeOH (4 mL). After 1 h, the reaction mixture was neutralized with Amberlite IR 120 (H^+) (0.25 g) resin. The reaction mixture was filtered and concentrated under reduced pressure, purified by column chromatography on silica gel (20% ethyl acetate: pet ether) to afford the desired compound **8** as a colourless liquid (0.19 g, 92%).

[α]$_D^{25}$ = +1.073 (c = 0.550, $CHCl_3$).

IR (cm^{-1}, $CHCl_3$) ν = 2924, 2851, 1733, 1465, 1378, 762.

^{1}H NMR (500 MHz, $CDCl_3$) δ 7.40- 7.34 (m, 10H), 5.35 (brs, 1H, -NH), 5.15- 5.08 (m, 2H), 4.86 (d, J = 3.2 Hz, 1H, H-1), 4.80 (d, J = 11.5 Hz, 1H), 4.72 (d, J = 11.5 Hz, 1H), 4.06 (d, J = 10.1 Hz, 1H), 3.94 (q, J = 6.4 Hz, 1H), 3.80- 3.76 (m, 1H), 3.63 (brs, 1H), 3.54- 3.48 (m, 2H), 3.34- 3.30 (m, 2H), 2.42 (brs, 1H, -OH), 1.86- 1.80 (m, 2H), 1.24 (d, J = 6.4 Hz, 3H, $-CH_3$).

^{13}C{^{1}H} NMR (125 MHz, $CDCl_3$) δ 156.6, 137.9, 136.7, 128.7, 128.5, 128.2, 128.11, 128.08, 98.1, 80.0, 76.1, 69.2, 66.7, 66.6, 61.1, 39.1, 29.3,16.8.

HRMS (ESI-TOF) (m/z) calculated for $C_{24}H_{31}N_4O_6$ $[M+H]^+$: 471.2238, found 471.2241.

Compound G10:

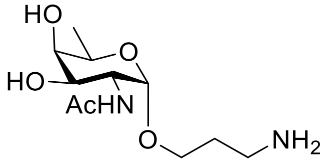

G10

Activated zinc dust (100 mg), acetic anhydride (0.05 mL), and acetic acid (0.1 mL) were added sequentially to the stirring solution of compound **8** (26 mg, 0.055 mmol) in THF (3 mL). The mixture was stirred overnight at room temperature. After completion of the reaction (indicated

by TLC), the reaction mixture was diluted with ethyl acetate, and zinc was filtered out through a celite pad, then the solvent was directly concentrated under reduced pressure. Toluene azeotrope was performed to remove the remaining acetic acid and kept under a high vacuum for the next step. The obtained amide compound was dissolved in *t*BuOH/CH_2Cl_2/H_2O (2/1/1, v/v/v, 2 mL: 1 mL: 1 mL, 4 mL). To this solution, $Pd(OH)_2/C$ (65 mg) was added and stirred under a hydrogen atmosphere (1 atm, balloon) for 24 h. The reaction mixture was then filtered through celite, concentrated, and dried over a vacuum to obtain compound **G10** (12 mg, 85% over two steps) as a sticky colourless liquid.

$[\alpha]_D^{25}$ = +78.595 (c = 0.083, MeOH).

IR (cm^{-1}, $CHCl_3$) ν = 3298, 2134, 1644, 1118, 1016, 751.

^1H NMR (500 MHz, D_2O) δ 4.79 (1H, H-1), 4.04 (dd, J = 11.1, 3.7 Hz, 1H), 3.99 (q, J = 6.5 Hz, 1H), 3.84 (dd, J = 11.1, 3.2 Hz, 1H), 3.74- 3.72 (m, 1H), 3.71- 3.68 (m, 1H), 3.47- 3.43 (m, 1H), 3.03 (t, J = 7.5 Hz, 2H), 1.96 (s, 3H), 1.92- 1.88 (m, 2H), 1.15 (d, J = 6.7 Hz, 3H, -CH_3).

$^{13}C\{^1H\}$ NMR (100 MHz, D_2O) δ 174.5, 97.0, 71.0, 67.6, 66.7, 65.0, 49.6, 37.1, 26.7, 21.9, 15.4.

HRMS (ESI-TOF) (m/z) calculated for $C_{11}H_{23}N_2O_5$ $[M+H]^+$: 263.1601, found 263.1603.

Compound G3:

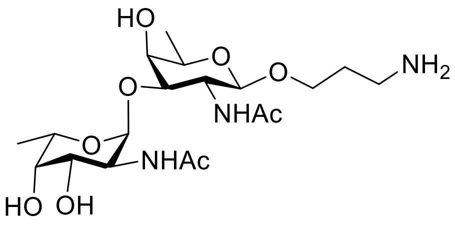

G3

Activated zinc dust (0.077 g), AcOH (0.07 mL), and Ac_2O (0.013 mL, 0.1416 mmol) were added in a stirring solution of compound **9** (0.018 g, 0.0236 mmol) in THF (2.5 mL) and stirred at room temperature for 10 h. The reaction was completed as monitored by TLC, Zinc was filtered out using Celite bed and EtOAc as an organic solvent, concentrated under reduced pressure, and purified by silica gel column chromatography (5% methanol: ethyl acetate) to obtain di-NHAc compound as a colourless foam. To this solution in MeOH (2 mL), $Pd(OH)_2/C$

(0.012 g) was added and the reaction was stirred under a hydrogen atmosphere for 14 h. The reaction mixture was filtered through a Celite bed using methanol as an eluent. Filtrate was concentrated under reduced pressure and dried under a high vacuum to obtain **G3** (6.7 mg, 83% over two steps) as a sticky solid.

$[\alpha]_D^{25}$ = -2.302 (*c* = 0.056, MeOH).

IR (cm^{-1}, $CHCl_3$) *ν* = 3040, 1725, 1547, 1261, 1065, 533.

^1H NMR (600 MHz, D_2O) *δ* 4.92 (d, *J* = 4.0 Hz, 1H), 4.31 (d, *J* = 8.6 Hz, 1H), 4.03 (t, *J* = 6.7 Hz, 2H), 3.90- 3.87 (m, 3H), 3.76 (brs, 1H), 3.71- 3.67 (m, 2H), 3.59 (m, 2H), 3.04 (m, 1H), 2.99 (m, 1H), 1.96- 1.93 (m, 8H), 1.19 (d, *J* = 6.4 Hz, 3H, -CH3), 1.15 (d, *J* = 6.6 Hz, 3H).

$^{13}C\{^1H\}$ NMR (100 MHz, D_2O) *δ* 174.3, 174.2, 101.5, 98.9, 76.5, 70.9, 70.7, 70.3, 67.9, 67.5, 67.1, 51.3, 49.5, 37.6, 26.6, 22.1, 15.4, 15.3.

HRMS (ESI-TOF) (m/z) calculated for $C_{19}H_{36}N_3O_9$ $[M+H]^+$: 450.2446, found 450.2441.

Compound 11:

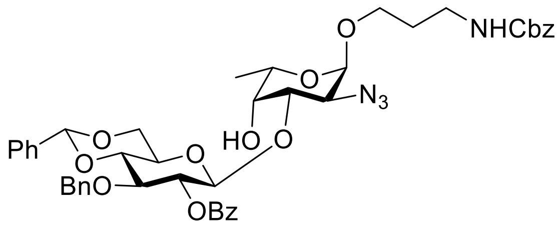

11

Anhydrous CH_2Cl_2 (4 mL) was added to the mixture of fully protected D-glucose donor **10** (96 mg, 0.17 mmol), 3-OH-L-fucosamine acceptor **2** (55 mg, 0.14 mmol), and molecular sieves (40 mg) and this solution was stirred at room temperature for 0.5 h. Then NIS (78 mg, 0.3470 mmol) and TMSOTf (6.29 µL, 0.007 mmol) were added to the stirring solution at 0 ºC and it was allowed to stir for 4 h. After completion of the reaction (monitored by TLC), Et_3N was added and 4Å molecular sieves were removed by filtering through a celite bed and the reaction mixture was diluted with CH_2Cl_2 (10 mL) and washed with saturated $Na_2S_2O_3$ solution. The separated organic layer was dried over anhydrous Na_2SO_4, concentrated, and purified by silica gel column chromatography (30% ethyl acetate: pet ether) to give regioselectively C3 coupled disaccharide compound **11** (78 mg, 66%) as a colourless viscous liquid.

[α]$_D^{25}$ = +1.514 (c = 0.525, $CHCl_3$).

IR (cm^{-1}, $CHCl_3$) ν = 2956, 2922, 2851, 1740, 1653, 1465, 1028, 761, 667.

^{1}H NMR (400 MHz, $CDCl_3$) δ 7.98 (d, J = 7.3 Hz, 2H), 7.61 (t, J =7.4 Hz,1H), 7.53- 7.51 (m, 2H), 7.46 (t, J = 7.7 Hz, 2H), 7.41- 7.38 (m, 2H), 7.34- 7.33 (m, 4H), 7.31-7.25 (m, 2H), 7.17- 7.10 (m, 5H), 5.60 (s, 1H, PhCH), 5.30 (t, J = 8.0 Hz, 1H, H-2'), 5.17- 5.04 (m, 3H), 4.86- 4.83 (m, 2H, H-1, PhCH), 4.74- 4.69 (m, 2H, H-1', PhCH), 4.38 (dd, J = 10.7, 4.9 Hz, 1H, H-6), 4.01 (dd, J = 10.5, 2.9 Hz, 1H, H-3), 3.92- 3.70 (m, 6H, H-3', H-5, H-6', H-4', H-4), 3.51- 3.47 (m, 3H, H-2, H-5'), 3.32 (q, J = 6.1 Hz, 2H), 1.84- 1.73 (m, 2H, CH_2-Linker), 1.10 (d, J =6.5 Hz, 3H, -CH_3).

^{13}C{^{1}H} NMR (100 MHz, $CDCl_3$) δ 165.6, 156.5, 137.7, 137.2, 133.6, 129.9, 129.8, 129.6, 129.3, 129.1, 128.9, 128.6, 128.32, 128.26, 128.16, 128.08, 127.7, 127.6, 126.0, 101.3, 98.4, 81.3, 77.63, 77.57, 74.1, 74.0, 69.7, 68.5, 66.6, 66.4, 65.6, 57.8, 39.0, 29.3, 16.0.

HRMS (ESI-TOF) (m/z) calculated for $C_{44}H_{49}N_4O_{12}$ $[M+H]^+$: 825.3341 found 825.3338.

Compound S1:

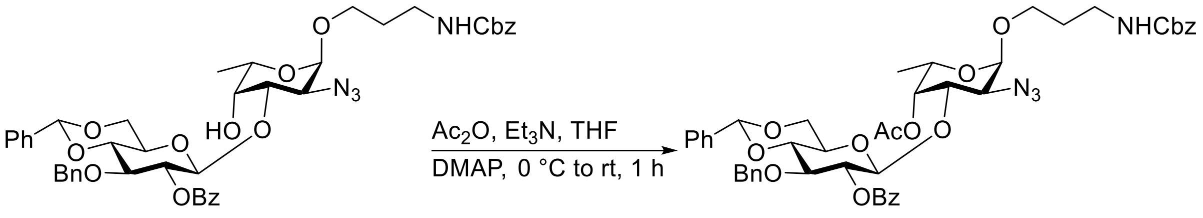

11 **S1**

Ac_2O (0.01 ml, 0.12 mmol), triethyl amine (0.017 mL, 0.12 mmol), and DMAP (3.7 mg, 0.03 mmol) were added to a solution of compound **11** (51 mg, 0.06 mmol) in THF (3 ml) at 0 °C. The reaction was quenched by adding ice water after one hour of stirring at room temperature, and the mixture was then extracted with EtOAc (12 mL x 2). The combined organic layer was washed with 2N HCl, aq. $NaHCO_3$, and brine solution, dried over anhydrous Na_2SO_4 and concentrated under vacuum. The crude product was purified by column chromatography on silica gel (20% ethyl acetate: petroleum ether) to give **S1** as a colourless sticky solid (44 mg, 83%).

[α]$_D^{25}$ = -42.089 (c = 0.32, $CHCl_3$).

IR (cm^{-1}, $CHCl_3$) ν = 2925, 2115, 1724, 1263, 1098, 996, 796, 755.

^{1}H NMR (500 MHz, CDCl$_3$) δ 8.06 (d, J = 7.3 Hz, 2H), 7.63 (t, J = 7.4 Hz, 1H), 7.53- 7.51 (m, 3H), 7.45- 7.48 (m, 1H), 7.45- 7.40 (m, 3H), 7.39 (d, J = 4.4 Hz, 4H), 7.35- 7.32 (m, 2H), 7.12- 7.08 (m, 4H), 5.56 (s, 1H, PhCH), 5.32- 5.29 (m, 2H, H2', -NH), 5.23 (d, J = 2.9 Hz, 1H, H-4), 5.14 (t, J = 13.7 Hz, 2H, -NHCbzCH$_2$), 4.86 (d, J = 3.4 Hz, 1H, H-1), 4.81 (d, J = 11.9 Hz, 1H, PhCH), 4.73 (d, J = 7.6 Hz, 1H, H-1'), 4.69 (d, J = 11.9 Hz, 1H, PhCH), 4.39- 4.34 (m, 2H, H-3, H-6), 3.99- 3.97 (m, 1H, H-5), 3.87- 3.83 (m, 2H, H-3', -OCH Linker), 3.81- 3.74 (m, 2H, H-6'), 3.56- 3.50 (m, 2H, H-2, -OCH Linker), 3.47- 3.43 (m, 1H, H-4'), 3.38 (d, J = 6.0 Hz, 2H, -NCH$_2$ Linker), 1.89- 1.82 (m, 2H, CH$_2$-Linker), 1.48 (s, 3H), 1.04 (d, J = 6.5 Hz, 3H, CH$_3$).

^{13}C{^{1}H} NMR (100 MHz, CDCl$_3$) δ 170.2, 164.8, 156.5, 137.7, 137.3, 136.7, 133.2, 130.2, 129.6, 129.0, 128.6, 128.3, 128.23, 128.16, 128.10, 127.6, 126.0, 101.2, 98.4, 98.0, 81.5, 77.9, 74.0, 73.2, 71.5, 68.8, 68.6, 66.9, 66.7, 66.2, 65.1, 57.7, 39.2, 29.2, 19.8, 16.1.

HRMS (ESI-TOF) (m/z) calculated for $C_{46}H_{50}N_4O_{13}Na$ $[M+Na]^+$: 889.3267 found 889.3256

Compound 12:

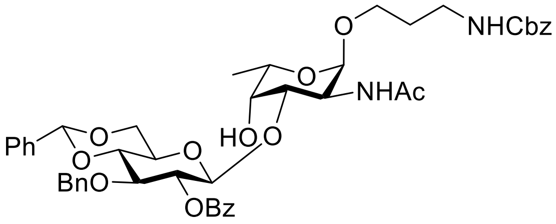

12

Activated zinc dust (0.160 g), AcOH (0.04 mL) and Ac$_2$O (0.02 mL) were added in a stirring solution of compound **11** (36 mg, 0.043 mmol) in THF (2 mL) and stirred at room temperature for 12 h. Reaction was completed as monitored by TLC, Zinc was filtered out using Celite bed and EtOAc as an organic solvent, concentrated under reduced pressure and purified by silica gel column chromatography (70% ethyl acetate: pet ether) to obtain acetamide compound **12** (28 mg, 78%) as a colourless foam.

$[\alpha]_D^{25}$ = -0.0362 (c = 0.525, CHCl$_3$).

IR (cm^{-1}, CHCl$_3$) ν = 2956, 2922, 2850, 1737, 1465, 1378, 1215, 1028, 761, 668.

^{1}H NMR (400 MHz, CDCl$_3$) δ 7.97 (d, J = 7.4 Hz, 2H), 7.60 (t, J = 7.3 Hz, 1H), 7.51- 7.50 (m, 2H), 7.45 (t, J = 7.7 Hz, 2H), 7.41- 7.36 (m, 3H), 7.32- 7.28 (m, 5H), 7.18- 7.09 (m, 5H),

6.09 (d, *J* = 6.8 Hz, 1H, -NH), 5.58 (s, 1H, PhCH), 5.26 (t, *J* = 8.0 Hz, 1H, H-2'), 5.11- 4.97 (m, 4H), 4.83 (d, *J* = 11.9 Hz, 1H), 4.70 (d, *J* = 11.9 Hz, 1H), 4.64 (d, *J* = 7.7 Hz, 1H), 4.40 (dd, *J* = 9.9, 4.6 Hz, 1H), 4.26 (m, 1H), 3.87- 3.82 (m, 2H), 3.80- 3.72 (m, 2H), 3.70- 3.66 (m, 1H), 3.48 (brs, 2H), 3.44- 3.38 (m, 1H), 3.34 (t, *J* = 6.6 Hz, 1H), 3.27 (t, *J* = 6.4 Hz, 1H), 1.89 (s, 3H), 1.78- 1.73 (m, 2H), 1.11 (d, *J* = 6.4 Hz, 3H).

$^{13}C\{^1H\}$ NMR (100 MHz, $CDCl_3$) *δ* 170.6, 165.6, 156.5, 137.7, 137.0, 136.6, 133.6, 129.8, 129.3, 129.1, 128.6, 128.5, 128.3, 128.2, 128.1, 128.0, 127.7, 127.5, 126.0, 101.4, 101.3, 97.3, 81.3, 78.6, 77.6, 77.2, 74.0, 73.7, 69.9, 68.5, 66.6, 66.3, 65.1, 48.1, 38.3, 29.5, 23.2, 16.2.

HRMS (ESI-TOF) (m/z) calculated for $C_{46}H_{53}N_2O_{13}$ $[M+H]^+$: 841.3542 found 841.3546.

Compound 13:

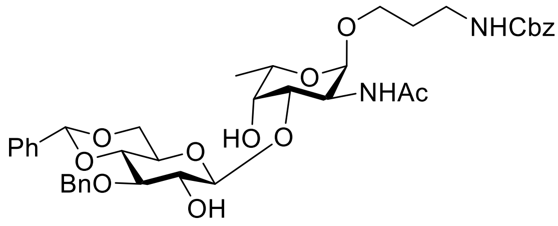

13

NaOMe (21.6 mg) was added to a stirred solution of **12** (20 mg, 0.023 mmol) in MeOH (2 mL). After 30 minutes reaction mixture was neutralized with amberlite IR 120 (H^+) (50 mg) resin. The reaction mixture was filtered and concentrated under reduced pressure purified by column chromatography on silica gel (5% methanol: ethyl acetate) to afford the desired compound **13** (15.5 mg, 92%) as a viscous liquid.

$[\alpha]_D^{25}$ = +1.60 (*c* = 0.525, $CHCl_3$).

IR (cm^{-1}, $CHCl_3$) *ν* = 2922, 1729, 1464, 761.

^1H NMR (500 MHz, $CDCl_3$) *δ* 7.40- 7.34 (m, 12H), 7.23- 7.22 (m, 3H), 6.46 (d, *J* = 9.5 Hz, 1H), 6.0 (brs, 1H), 5.30 (s, 1H), 5.11 (q, *J* = 10.2 Hz, 2H), 4.93-4.87 (m, 3H), 4.85- 4.84 (m, 1H), 4.48 (t, *J* = 10.0 Hz, 1H), 4.30 (d, *J* = 7.5 Hz, 1H), 4.17 (dd, *J* = 10.0, 4.7 Hz, 1H), 3.89- 3.88 (m, 2H), 3.81 (t, *J* = 9.3 Hz, 1H), 3.75- 3.73 (m, 1H), 3.68 (d, *J* = 10.4 Hz, 1H), 3.64 (t, *J* = 9.1 Hz, 1H), 3.56- 3.52 (m, 1H), 3.50- 3.43 (m, 2H), 3.31- 3.29 (m, 2H), 2.07 (s, 3H), 1.78 (m, 2H), 1.33 (d, *J* = 6.4 Hz, 3H, $-CH_3$).

$^{13}C\{^1H\}$ NMR (125 MHz, $CDCl_3$) δ 172.6, 156.6, 138.9, 137.5, 136.5, 128.7, 128.6, 128.3, 128.2, 128.1, 128.0, 127.6, 126.0, 103.9, 100.7, 97.2, 81.1, 80.8, 79.5, 75.2, 73.8, 69.5, 68.3, 66.8, 66.4, 63.8, 47.9, 37.6, 29.4, 23.1, 16.5.

HRMS (ESI-TOF) (m/z) calculated for $C_{39}H_{49}N_2O_{12}$ $[M+H]^+$: 737.3280 found 737.3284.

Compound G4:

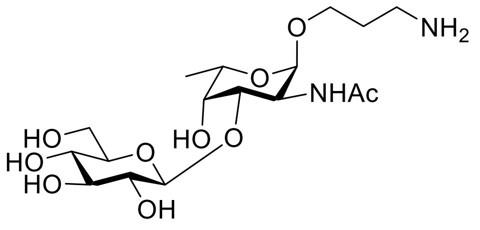

G4

The compound **13** (16 mg, 0.021 mmol) was dissolved in MeOH (2 mL). To this solution $Pd(OH)_2/C$ (0.08 g, 20%Pd content) was added. The reaction was stirred under hydrogen atmosphere for 8 h. The reaction mixture was filtered through a Celite bed with methanol as eluent. Filtrate was concentrated under reduced pressure and dried under high vacuum to obtain **G4** (8.9 mg, 97%) as a sticky solid.

$[\alpha]_D^{25}$ = -45.732 (c = 0.083, MeOH).

IR (cm^{-1}, $CHCl_3$) ν = 3357, 2136, 1645, 1016, 699.

^1H NMR (600 MHz, D_2O) δ 4.85 (1H), 4.47 (d, J = 7.9 Hz, 1H), 4.15- 4.13 (m, 1H), 4.07- 4.05 (m, 1H), 4.00- 3.95 (m, 2H), 3.90 (d, J = 10.7 Hz, 1H), 3.74- 3.69 (m, 1H), 3.68- 3.63 (m, 1H), 3.49- 3.46 (m, 1H), 3.43- 3.37 (m, 1H), 3.29 (t, J = 9.4 Hz, 1H), 3.23 (t, J = 8.6 Hz, 1H), 3.07- 3.02 (m, 2H), 1.97 (s, 3H), 1.93- 1.89 (m, 2H), 1.19 (d, J = 6.5 Hz, 3H).

$^{13}C\{^1H\}$ NMR (125 MHz, D_2O) δ 174.5, 100.3, 96.9, 76.1, 75.4, 75.1, 72.8, 69.7, 68.5, 66.5, 65.0, 61.0, 48.0, 37.1, 26.7, 22.0, 15.4.

HRMS (ESI-TOF) (m/z) calculated for $C_{17}H_{33}N_2O_{10}$ $[M+H]^+$: 425.2130 found 425.2138.

Compound 15:

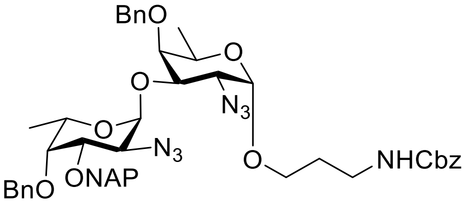

15

Anhydrous CH_2Cl_2: Et_2O (1: 3, 6mL) was added to the mixture of L-fucosamine donor **14** (0.343 g, 0.6715 mmol), D-fucosamine acceptor **8** (0.158 g, 0.3357 mmol) and molecular sieves (350 mg) and this solution was stirred at room temperature for 0.5 h. Then NIS (0.302 g, 1.343 mmol) and TMSOTf (24 µL, 0.1343 mmol) were added to the stirring solution at -78 ºC and it was allowed to stir for 12 h. After completion of the reaction (consumption of both donor and acceptor were monitored by TLC), Et_3N was added and 4Å molecular sieves were removed by filtering through a celite bed and the reaction mixture was diluted with CH_2Cl_2 (20 mL) and washed with saturated $Na_2S_2O_3$ solution. The separated organic layer was dried over anhydrous Na_2SO_4, concentrated, and purified by silica gel column chromatography (20% Ethyl acetate: pet ether) to give a sole-isomer of disaccharide **15** (0.24 g, 82%) as a colourless viscous liquid

$[\alpha]_D^{25}$ =+3.48 (*c* = 0.590, $CHCl_3$).

IR (cm^{-1}, $CHCl_3$) ***ν*** = 2922, 1736, 1465, 761.

^{1}H NMR (500 MHz, $CDCl_3$) ***δ*** 7.83- 7.81 (m, 2H), 7.78 (brs, 1H), 7.73- 7.71 (m, 2H), 7.52 (dd, *J* = 8.4, 1.5 Hz, 1H), 7.49- 7.43 (m, 2H), 7.37- 7.27 (m, 15H), 5.41 (brs, 1H), 5.16 (brs, 1H), 5.14-5.06 (m, 2H), 4.97 (d, *J* = 11.4 Hz, 1H), 4.92- 4.90 (m, 1H), 4.87 (brs, 1H), 4.83- 4.80 (m, 1H), 4.78- 4.74 (m, 1H), 4.62 (dd, *J* = 11.5, 3.1 Hz, 2H), 4.04- 4.00 (m, 1H), 3.95- 3.85 (m, 5H), 3.82- 3.77 (m, 1H), 3.57 (brs, 1H), 3.53 (brs, 1H), 3.52- 3.48 (m, 1H), 3.42- 3.37 (m, 1H), 3.34- 3.28 (m, 1H), 1.84- 1.80 (m, 2H), 1.20 (d, *J* = 6.5 Hz, 3H, -CH3), 1.13 (d, *J* = 6.5 Hz, 3H, $-CH_3$).

^{13}C{^{1}H} NMR (125 MHz, $CDCl_3$) ***δ*** 156.6, 138.3, 138.2, 136.7, 135.1, 133.3, 133.1, 128.7, 128.54, 128.45, 128.41, 128.35, 128.28, 128.18, 128.10, 128.05, 128.0, 127.8, 127.7, 126.7, 126.2, 126.1, 125.8, 99.8, 98.1, 79.9, 77,4, 76.6, 76.4, 75.4, 75.0, 72.6, 67.7, 67.0, 66.6, 60.8, 59.8, 39.4, 29.2, 16.82, 16.79.

HRMS (ESI-TOF) (m/z) calculated for $C_{48}H_{53}N_7O_9Na$ $[M+Na]^+$: 894.3797, found 894.3797.

Compound 16:

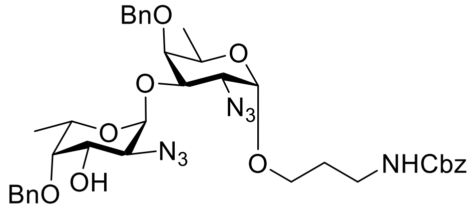

16

DDQ (137 mg, 0.6055 mmol) was added to a stirring solution of **15** (176 mg, 0.2018 mmol) in CH_2Cl_2 (3 mL) and H_2O (0.3 mL) at rt, and the reaction mixture was allowed to stir for 1 h. After completion of the reaction, the reaction mixture was quenched with Et_3N, the reaction mixture was diluted with CH_2Cl_2 (2×10 mL) and washed with saturated sodium bicarbonate solution. Separated organic layer was dried over anhydrous Na_2SO_4 and concentrated in vacuo. The crude product was purified by column chromatography on silica gel (30% ethyl acetate: pet ether) to obtain **16** (115 mg, 78%) as a pale-yellow viscous liquid.

$[\alpha]_D^{25}$ = +2.064 (*c* = 0.590, $CHCl_3$).

IR (cm^{-1}, $CHCl_3$) *ν* = 2956, 2924, 2853, 1737, 1464, 1378, 1215, 1025, 762.

^1H NMR (500 MHz, $CDCl_3$) *δ* 7.41- 7.33 (m, 15H), 5.45 (brs, 1H), 5.17 (d, *J* = 3.2 Hz, 1H), 5.12 (q, *J* = 10.8 Hz, 2H), 4.95 (d, *J* = 3.2 Hz, 1H), 4.84- 4.70 (m, 4H), 4.04- 3.93 (m, 5H), 3.85- 3.81 (m, 1H), 3.58- 3.56 (m, 2H), 3.55- 3.51 (m, 1H), 3.44- 3.35 (m, 2H), 3.34- 3.30 (m, 1H), 1.86- 1.84 (m, 2H), 1.26 (dd, *J* = 6.3, 3.1 Hz, 6H).

$^{13}C\{^1H\}$ NMR (125 MHz, $CDCl_3$) *δ* 156.6, 138.2, 137.8, 136.7, 128.7, 128.54, 128.45, 128.23, 128.19, 128.1, 128.0, 127.9, 127.8, 99.9, 98.1, 80.1, 79.8, 76.3, 76.1, 75.5, 68.6, 67.4, 67.13, 67.10, 66.6, 60.9, 60.8, 39.4, 29.2, 16.8.

HRMS (ESI-TOF) (m/z) calculated for $C_{37}H_{46}N_7O_9$ $[M+H]^+$: 732.3352 found 732.3348.

Compound G11:

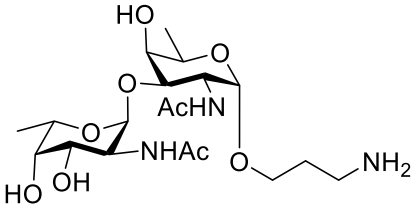

G11

Activated zinc dust (0.307 g, 4.7006 mmol), AcOH (0.03 mL) and Ac_2O (0.022 mL, 0.2348 mmol) were added in a stirring solution of compound **16** (43 mg, 0.0587 mmol) in THF (3 mL) and stirred at room temperature for 10 h. Reaction was completed as monitored by TLC, zinc was filtered out using Celite bed and EtOAc as an organic solvent. Filtrate was concentrated under reduced pressure and purified by silica gel column chromatography (5% methanol: ethyl acetate) to obtain di-acetamido compound as a sticky liquid. To the stirring solution of acetamido compound in MeOH (4 mL), 20 wt% $Pd(OH)_2/C$ (45 mg) was added and reaction was stirred under hydrogen atmosphere for 14 h. The reaction mixture was filtered through a Celite bed using methanol as an eluent. Filtrate was concentrated under reduced pressure and dried under high vacuum to obtain **G11** (20 mg, 79%) as a sticky liquid

$[\alpha]_D^{25}$ = -1.690 (c = 0.233, MeOH).

IR (cm^{-1}, $CHCl_3$) ν = 3582, 2948, 2835, 1656, 1023, 845.

^{1}H NMR (500 MHz, D_2O) δ 4.93 (d, J = 4.0 Hz, 1H), 4.76 (1H), 4.23 (dd, J = 11.1, 3.8 Hz, 1H), 4.05- 4.01 (m, 3H), 3.89- 3.85 (m, 3H), 3.76- 3.68 (m, 4H), 3.47- 3.43 (m, 1H), 3.04 (t, J = 7.3 Hz, 2H), 1.96 (s, 3H), 1.92 (s, 3H), 1.93- 1.87 (m, 2H), 1.15 (dd, J = 6.5, 3.6 Hz, 6H).

^{13}C{^{1}H} NMR (125 MHz, D_2O) δ 174.2, 173.8, 98.7, 97.2, 73.6, 71.1, 70.9, 67.6, 67.1, 66.6, 65.0, 49.5, 48.6, 37.1, 26.8, 22.2, 21.9, 15.4, 15.2.

HRMS (ESI-TOF) (m/z) calculated for $C_{19}H_{36}N_3O_9$ $[M+H]^+$: 450.2446 found 450.2449.

Compound 17:

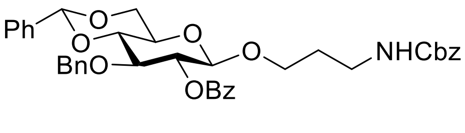

17

Donor **10** (156 mg, 0.28 mmol), acceptor **6** (70 mg, 0.33 mmol), and molecular sieves (3Å, 100 mg) were dissolved in anhydrous CH_2Cl_2 and kept for stirring at room temperature for 0.5 h. Then, NIS (126 mg, 0.5624 mmol) and TMSOTf (10.2 µL, 0.05624 mmol) were added dropwise to the solution at 0 ºC and stirred for over 3 h. After completion of the reaction, the mixture was quenched using Et_3N, and molecular sieves were filtered out through Celite. Then crude was concentrated and purified by flash column chromatography (20% ethyl acetate: petroleum ether) to afford linker appended glucose derivative **17** (150 mg, 82%) as an amorphous white solid.

$[\alpha]_D^{25}$ = +1.488 (c = 0.475, $CHCl_3$).

IR (cm^{-1}, $CHCl_3$) ν = 2954, 2922, 2851, 2255, 1724, 1464, 1378, 761.

^{1}H NMR (400 MHz, $CDCl_3$) δ 7.98 (d, J = 7.5 Hz, 2H), 7.70 (d, J = 7.2 Hz, 1H), 7.59- 7.46 (m, 5H), 7.44- 7.38 (m, 5H), 7.29- 7.24 (m, 2H), 7.16- 7.07 (m, 5H), 5.6 (s, 1H, PhCH), 5.29 (t, J = 8.1 Hz, 1H, H-2), 5.08- 4.98 (m, 3H), 4.84 (d, J = 12.1 Hz, 1H), 4.70 (d, J = 12.1 Hz, 1H), 4.59 (d, J = 7.9 Hz, 1H), 4.37 (dd, J = 10.5, 4.9 Hz, 1H), 3.90- 3.80 (m, 3H), 3.56- 3.50 (m, 2H), 3.31- 3.21 (m, 1H), 3.19- 3.08 (m, 1H), 1.71- 1.70 (m, 2H).

^{13}C{^{1}H} NMR (100 MHz, $CDCl_3$) δ 165.2, 156.4, 137.8, 137.2, 133.3, 132.3, 131.2, 130.4, 129.8, 129.6, 129.2, 129.1, 128.9, 128.53, 128.49, 128.46, 128.3, 128.2, 128.1, 128.0, 127.6, 126.0, 125.2, 124.5, 101.7, 101.3, 81.7, 77.7, 74.0, 73.4, 68.7, 67.7, 66.4, 66.3, 38.1, 29.4.

HRMS (ESI-TOF) (m/z) calculated for $C_{38}H_{40}NO_9$ $[M+H]^+$: 654.2698 found 654.2698.

Compound 18:

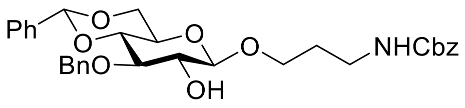

18

0.2M NaOMe (43 mg) was added to a stirred solution of **17** (156 mg, 0.2388 mmol) in MeOH (4 mL). After 1 h, reaction mixture was neutralized with Amberlite IR 120 (H^+) (250 mg) resin. The reaction mixture was filtered and concentrated under reduced pressure, purified by column chromatography on silica gel (40% ethyl acetate: pet ether) to afford the desired compound **18** (114 mg, 87%) as a colourless liquid.

$[\alpha]_D^{25}$ = -0.568 (c = 0.555, $CHCl_3$).

IR (cm^{-1}, $CHCl_3$) ν = 2927, 1716, 1462, 1375, 1087, 759.7.

^{1}H NMR (400 MHz, $CDCl_3$) δ 7.51- 7.49 (m, 2H), 7.42- 7.28 (m, 13H), 5.56 (s, 1H, -PhCH), 5.26 (t, J= 5.6 Hz, 1H, -NH), 5.10 (brs, 2H, -NHCbzCH_2), 4.97 (d, J = 11.7 Hz, 1H, PhCH), 4.83 (d, J = 11.6 Hz, 1H, PhCH), 4.38- 4.31 (m, 2H, H-1, H-6), 3.98- 3.94 (m, 1H, H-3), 3.77 (t, J = 10.3 Hz, 1H, H6'), 3.68- 3.57 (m, 3H, H4, H5), 3.49- 3.40 (m, 2H), 3.30- 3.23 (m, 2H, -NCH_2 Linker), 1.83- 1.76 (m, 2H, -CH_2 Linker).

^{13}C{^{1}H} NMR (100 MHz, $CDCl_3$) δ 156.8, 138.5, 137.3, 136.6, 129.0, 128.6, 128.4, 128.3, 128.19, 128.16, 128.0, 127.8, 126.0, 103.4, 101.2, 81.3, 80.4, 74.6, 74.4, 68.7, 67.7, 66.7, 66.4, 38.0, 29.6.

HRMS (ESI-TOF) (m/z) calculated for $C_{31}H_{36}NO_8$ $[M+H]^+$: 550.2435 found 550.2431.

Compound 20:

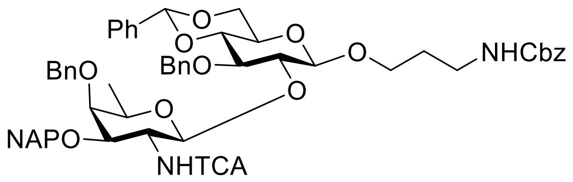

20

Thioglycoside donor **19** (112 mg, 0.18 mmol) and acceptor **18** (82 mg, 0.15 mmol) were azeotrope with toluene and dissolved in anhydrous CH_2Cl_2 (4 mL). Activated molecular sieves (3Å, 100 g) were added, and the mixture was stirred at room temperature for 30 minutes under nitrogen. NIS (80 mg, 0.3578 mmol) and TMSOTf (6.47 µL, 0.03 mmol) were added dropwise at 0 °C. After 2 h, TLC (EtOAc: Hexane, 4:6, v/v) showed the acceptor has been converted into a less polar product. The mixture was neutralized with triethyl amine, and filtered through a Celite bed using CH_2Cl_2. The organic layer was washed with aq. $Na_2S_2O_3$ solution, separated organic layer was dried over anhydrous Na_2SO_4, concentrated in vacuo, and purified by column chromatography on silica gel (30% ethyl acetate: pet ether) afforded disaccharide **20** (126 mg, 79%) as a sticky liquid.

$[\alpha]_D^{25}$ = +2.183 (c = 0.545, $CHCl_3$).

IR (cm^{-1}, $CHCl_3$) ν = 2955, 2920, 2850, 2280, 1735, 1464, 1378, 979, 761.

^{1}H NMR (500 MHz, CDCl$_3$) ***δ*** 7.90- 7.80 (m, 5H), 7.55- 7.53 (m, 2H), 7.49- 7.47 (m, 3H), 7.41- 7.38 (m, 4H), 7.36- 7.33 (m, 6H), 7.32- 7.30 (m, 4H), 7.21 (t, *J* = 7.4 Hz, 2H), 7.17- 7.15 (m, 1H), 6.77 (d, *J* = 7.5 Hz, 1H, -NH), 5.60 (t, *J* = 5.7 Hz, 1H), 5.56 (brs, 1H), 5.14 (d, *J* = 8.2 Hz, 1H), 5.09 (q, *J* = 9.9 Hz, 2H), 4.97 (d, *J* = 11.2 Hz, 2H), 4.81 (d, *J* = 11.6 Hz, 1H), 4.73- 4.68 (m, 3H), 4.50 (d, *J* = 6.8 Hz, 1H), 4.37 (dd, *J*= 10.5, 5.0 Hz, 1H), 4.18- 4.15 (m, 1H), 3.98- 3.94 (m, 2H), 3.81- 3.75 (m, 3H), 3.71- 3.66 (m, 3H), 3.52 (q, *J* = 6.2 Hz, 1H), 3.47- 3.42 (m, 1H), 3.38- 3.35 (m, 2H), 1.94- 1.91 (m, 1H), 1.86- 1.82 (m, 1H), 1.23 (d, *J* = 6.3 Hz, 3H, CH$_3$).

^{13}C{^{1}H} NMR (125 MHz, CDCl$_3$) ***δ*** 161.9, 156.7, 138.6, 138.2, 137.2, 136.8, 135.1, 133.3, 133.1, 129.0, 128.5, 128.40, 128.39, 128.30, 128.27, 128.1, 128.0, 127.9, 127.8, 126.8, 126.3, 126.2, 126.0, 125.9, 102.8, 101.2, 99.2, 92.7, 81.7, 78.2, 77.6, 74.8, 74.5, 74.4, 72.2, 70.7, 68.8, 67.9, 66.4, 65.7, 56.3, 37.7, 29.0, 17.1.

HRMS (ESI-TOF) (m/z) calculated for $C_{57}H_{59}Cl_3N_2O_{12}$ $[M+H]^+$: 1069.3194 found 1071.3192.

Compound 21:

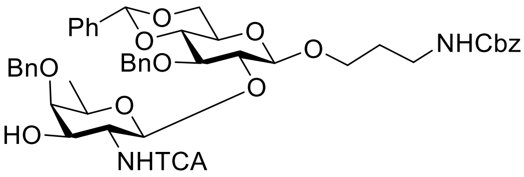

21

DDQ (54 mg, 0.2382 mmol) was added to a stirred solution of **19** (85 mg, 0.0794 mmol) in CH_2Cl_2 (4 mL) and H_2O (0.4 mL) at rt, and the reaction mixture was allowed to stir for 45 mins. After completion of the reaction, the reaction mixture was quenched with Et_3N, the reaction mixture was diluted with CH_2Cl_2 (2×15 mL) and washed with saturated sodium bicarbonate solution. The separated organic layer was dried over anhydrous Na_2SO_4 and concentrated in vacuo. The crude product was purified by column chromatography on silica gel (40% ethyl acetate: pet ether) to obtain **21** (61 mg, 83%) as a pale-yellow viscous liquid.

$[\alpha]_D^{25}$ = +1.877 (*c* = 0.580, CHCl$_3$).

IR (cm^{-1}, CHCl$_3$) ***ν*** = 3370, 2926, 2395, 2171, 1706, 1261, 1096, 804, 582.

^{1}H NMR (400 MHz, CDCl$_3$) ***δ*** 7.45- 7.25 (m, 20H), 6.59 (d, *J* = 7.3 Hz, 1H), 5.66 (t, *J* = 5.7 Hz, 1H, H-2), 5.5 (s, 1H, PhCH), 5.10- 5.00 (m, 3H), 4.85 (d, *J* = 8.1 Hz, 1H), 4.77- 4.61 (m,

3H), 4.44 (d, J = 6.8 Hz, 1H), 4.35 (dd, J = 10.5, 5.0 Hz, 1H), 3.95- 3.91 (m, 1H), 3.79- 3.64 (m, 7H), 3.51- 3.33 (m, 5H), 2.44 (d, J = 9.3 Hz, 1H, -OH), 1.84- 1.79 (m, 2H), 1.23 (d, J = 6.4 Hz, 3H, $-CH_3$).

$^{13}C\{^1H\}$ NMR (100 MHz, $CDCl_3$) δ 162.8, 156.8, 138.6, 137.7, 137.1, 136.8, 129.1, 128.6, 128.54, 128.47, 128.34, 128.31, 128.2, 128.1, 128.0, 127.9, 127.4, 126.0, 102.3, 101.2, 99.9, 92.6, 82.0, 81.5, 78.5, 77.9, 75.8, 74.2, 72.0, 71.0, 68.7, 67.9, 66.4, 65.7, 57.1, 37.5, 28.8, 16.8.

HRMS (ESI-TOF) (m/z) calculated for $C_{46}H_{52}Cl_3N_2O_{12}$ $[M+H]^+$: 931.2564 found 931.2559.

Compound 23:

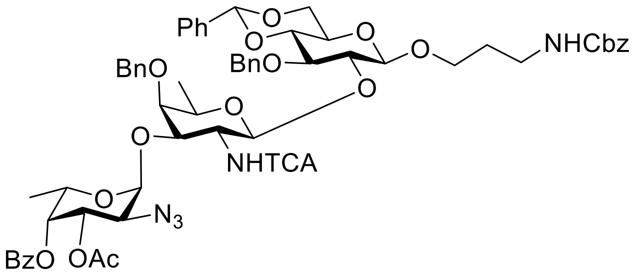

23

Anhydrous CH_2Cl_2 (3 mL) was added to the mixture of fully protected thioglycoside donor **22** (31 mg, 0.07 mmol), disaccharide acceptor **21** (46 mg, 0.05 mmol), and molecular sieves (40 mg), and this solution was stirred at room temperature for 0.5 h. Then NIS (33 mg, 0.1483 mmol) and TMSOTf (2.68 µL, 0.0148 mmol) were added to the stirring solution at 0 ºC and it was allowed to stir for 4 h. After completion of the reaction (monitored by TLC), Et_3N was added and 4Å molecular sieves were removed by filtering through a celite bed and the reaction mixture was diluted with CH_2Cl_2 (10 mL) and washed with saturated $Na_2S_2O_3$ solution. The separated organic layer was dried over anhydrous Na_2SO_4, concentrated, and purified by silica gel column chromatography (30% ethyl acetate: pet ether) to give trisaccharide compound **23** (54 mg, 89%) as a colourless viscous liquid.

$[\alpha]_D^{25}$ = +2.450 (c = 0.640, $CHCl_3$).

IR (cm^{-1}, $CHCl_3$) ν = 3420, 2921, 2401, 1732, 1465, 1255, 983, 764, 599.

^1H NMR (500 MHz, $CDCl_3$) δ 8.09 (d, J = 7.3 Hz, 2H), 7.64 (t, J = 7.4 Hz, 1H), 7.53- 7.47 (m, 5H), 7.44- 7.43 (m, 4H), 7.41- 7.33 (m, 13H), 7.16 (d, J = 7.0 Hz, 1H, -NH), 5.5 (s, 1H, PhCH), 5.43 (t, J = 5.6 Hz, 1H), 5.36- 5.35 (m, 2H), 5.22 (d, J = 8.1 Hz, 1H), 5.10- 5.06 (m,

3H), 4.98 (d, J = 11.2 Hz, 1H), 4.82 (q, J = 13.3 Hz, 2H), 4.74 (d, J = 11.2 Hz, 1H), 4.51 (d, J = 7.3 Hz, 1H), 4.40- 4.34 (m, 2H), 3.96- 3.88 (m, 3H), 3.84- 3.76 (m, 3H), 3.75- 3.72 (m, 1H), 3.67 (t, J = 9.2 Hz, 2H), 3.62- 3.59 (m, 2H), 3.45- 3.41 (m, 1H), 3.37- 3.30 (m, 2H), 2.04 (s, 3H), 1.91- 1.87 (m, 1H), 1.85- 1.83 (m, 1H), 1.31 (d, J = 6.3 Hz, 3H), 1.05 (d, J = 6.4 Hz, 3H).

$^{13}C\{^1H\}$ NMR (125 MHz, $CDCl_3$) δ 169.7, 165.9, 162.2, 156.6, 138.7, 138.1, 137.1, 136.8, 133.6, 129.8, 129.3, 129.0, 128.65, 128.57, 128.49, 128.47, 128.3, 128.1, 128.0, 127.9, 127.5, 126.0, 102.8, 101.1, 99.2, 98.9, 92.4, 81.8, 81.5, 78.6, 78.4, 77.8, 75.1, 74.8, 70.9, 70.8, 69.4, 68.7, 67.8, 66.5, 65.8, 58.2, 56.6, 37.8, 29.2, 20.7, 17.4, 16.1.

HRMS (ESI-TOF) (m/z) calculated for $C_{61}H_{67}Cl_3N_5O_{17}$ $[M+H]^+$: 1248.3583 found 1248.3576.

Compound G6:

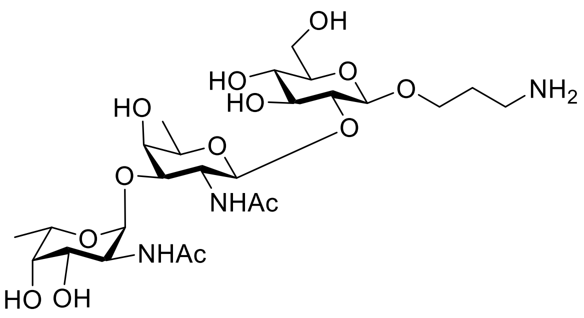

G6

0.2M NaOMe (43 mg) was added to a stirred solution of **22** (38 mg, 0.0304 mmol) in MeOH (4 mL). After 1 hour, the reaction mixture was neutralized with amberlite IR 120 (H+) resin. The reaction mixture was filtered and concentrated under reduced pressure purified by column chromatography on silica gel (50% ethyl acetate: pet ether) to afford the desired diol derivative (31 mg, 93%) as viscous liquid.

Activated zinc dust (159 mg, 2.447 mmol) and AcOH (0.08 mL) were added in trisaccharide diol compound (31 mg, 0.0305 mmol) in THF (4 mL) and in the same pot Ac_2O (0.05 mL) were added and stirred at room temperature for 10 h. Reaction was completed as monitored by TLC, Zinc was filtered out using Celite bed and EtOAc as an organic solvent, concentrated under reduced pressure and purified by silica gel column chromatography (10% methanol: ethyl acetate) to obtain di-NHAc compound as a colourless foam. To this solution in MeOH (3 mL), $Pd(OH)_2/C$ (27 mg) was added and reaction was stirred under hydrogen atmosphere for

36 h. The reaction mixture was filtered through a Celite bed using methanol as an eluent, concentrated and purified by using Sephadex-G25 gel (milli-Q water as an eluent) to afford target molecule **G6** (15 mg, 84% over 2 steps) as a sticky liquid.

[*α*]$_D^{25}$ = -30.056 (*c* = 0.161, MeOH).

IR (cm^{-1}, $CHCl_3$) *ν* = 3675, 2995, 2406, 2317, 1719, 1556, 1270, 844, 703, 664.

^{1}H NMR (500 MHz, D_2O) *δ* 4.89 (d, *J* = 3.8 Hz, 1H), 4.64 (d, *J* = 8.5 Hz, 1H), 4.42 (d, *J* = 7.7 Hz, 1H), 4.02- 3.97 (m, 3H), 3.87- 3.79 (m, 3H), 3.72- 3.62 (m, 5H), 3.59 (dd, *J* = 12.3, 5.8 Hz, 1H), 3.42 (t, *J* = 9.0 Hz, 1H), 3.36- 3.30 (m, 2H), 3.24 (t, *J* = 9.4 Hz, 1H), 3.10 (t, *J* = 6.0 Hz, 2H), 1.93 (s, 3H), 1.91 (s, 3H), 1.91- 1.84 (m, 2H), 1.16 (d, *J* = 6.4 Hz, 3H), 1.12 (d, *J* = 6.5 Hz, 3H).

^{13}C{^{1}H} NMR (125 MHz, D_2O) *δ* 174.5, 174.2, 101.8, 100.7, 99.0, 79.6, 76.6, 76.2, 75.6, 70.9, 70.6, 70.3, 69.9, 68.4, 67.5, 67.1, 60.5, 51.7, 49.5, 38.4, 26.1, 22.2, 22.1, 15.5, 15.3.

HRMS (ESI-TOF) (m/z) calculated for $C_{25}H_{46}N_3O_{14}$ $[M+H]^+$: 612.2974 found 612.2968.

Compound 24:

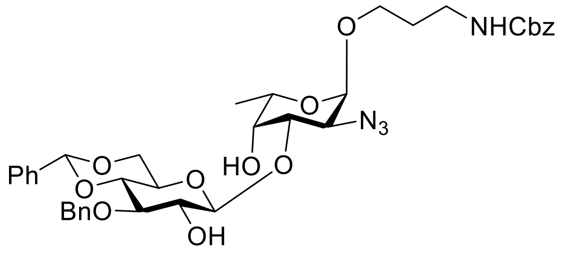

24

0.2M NaOMe (43 mg) was added to a stirred solution of **11** (78 mg, 0.0945 mmol) in MeOH (4 mL). After 1 hour, the reaction mixture was neutralized with amberlite IR 120 (H+) (0.75 mg) resin. The reaction mixture was filtered and concentrated under reduced pressure purified by column chromatography on silica gel (50% ethyl acetate: pet ether) to afford the desired compound **24** (53 mg, 78%) as viscous liquid.

[*α*]$_D^{25}$ = +1.998 (*c* = 0.60, $CHCl_3$).

IR (cm^{-1}, $CHCl_3$) *ν* = 3415, 2926, 2396, 1727, 1464, 1106, 984, 765, 591.

^{1}H NMR (500 MHz, CDCl$_3$) δ 7.52- 7.50 (m, 2H), 7.43- 7.39 (m, 3H), 7.38- 7.35 (m, 10H), 5.58 (s, 1H, PhCH), 5.21 (brs, 1H), 5.12 (q, J = 10.7 Hz, 2H), 5.02 (d, J = 11.6 Hz, 1H), 4.88 (d, J = 3.2 Hz, 1H), 4.75 (d, J = 11.6 Hz, 1H), 4.53 (d, J = 6.9 Hz, 1H), 4.35 (dd, J = 10.6, 4.8 Hz, 1H), 4.15 (dd, J = 10.4, 3.0 Hz, 1H), 3.97- 3.94 (m, 1H), 3.90 (brs, 1H), 3.82- 3.74 (m, 3H), 3.66- 3.59 (m, 3H), 3.54- 3.51 (m, 1H), 3.45- 3.41 (m, 1H), 3.39- 3.35 (m, 2H), 1.92- 1.86 (m, 1H), 1.84- 1.81 (m, 1H), 1.31 (d, J = 6.6 Hz, 3H, CH$_3$).

^{13}C{^{1}H} NMR (125 MHz, CDCl$_3$) δ 156.5, 138.1, 137.2, 136.6, 129.1, 128.63, 128.57, 128.3, 128.14, 128.07, 126.0, 101.2, 100.2, 98.1, 81.3, 80.4, 75.9, 74.5, 73.4, 68.6, 68.5, 66.7, 66.6, 66.4, 65.5, 57.6, 38.9, 29.3, 16.3.

HRMS (ESI-TOF) (m/z) calculated for $C_{37}H_{45}N_4O_{11}$ $[M+H]^+$: 721.3079 found 721.3071.

Compound 25:

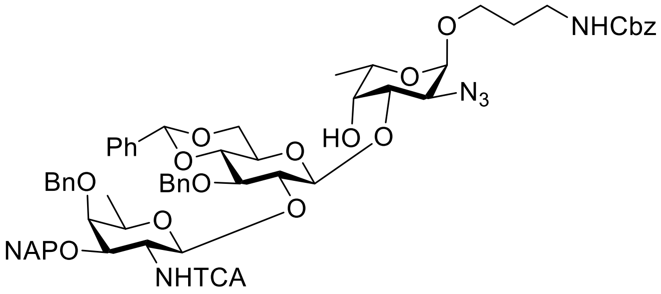

25

Anhydrous CH_2Cl_2 (3 mL) was added to the mixture of fully protected thioglycoside donor **19** (25 mg, 0.0399 mmol), disaccharide acceptor **24** (24 mg, 0.0332 mmol), and molecular sieves (30 mg) and this solution was stirred at room temperature for 0.5 h. Then NIS (17 mg, 0.0799 mmol) and TMSOTf (1.45 μL, 0.008 mmol) were added to the stirring solution at 0 ºC and it was allowed to stir for 4 h. After completion of the reaction (monitored by TLC), Et_3N was added and 4Å molecular sieves were removed by filtering through a celite bed and the reaction mixture was diluted with CH_2Cl_2 (10 mL) and washed with saturated $Na_2S_2O_3$ solution. The separated organic layer was dried over anhydrous Na_2SO_4, concentrated, and purified by silica gel column chromatography (20% ethyl acetate: pet ether) to give trisaccharide compound **25** (31 mg, 76%) as a colourless viscous liquid.

$[\alpha]_D^{25}$ = +1.810 (c = 0.665, CHCl$_3$).

IR (cm^{-1}, CHCl$_3$) ν = 2924, 1726, 1461, 1377, 1216, 762.

^{1}H NMR (400 MHz, $CDCl_3$) *δ* 7.86- 7.71 (m, 4H), 7.52- 7.50 (m, 2H), 7.41- 7.28 (m, 17H), 7.22- 7.20 (m, 2H), 7.07 (t, *J* = 7.3 Hz, 1H), 7.01 (t, *J* = 7.3 Hz, 1H), 6.42 (d, *J* = 8.4 Hz, 1H, NH), 5.49 (s, 1H), 5.13- 5.10 (m, 3H), 5.01- 4.89 (m, 4H), 4.79- 4.70 (m, 2H), 4.60 (t, *J* = 11. 6 Hz, 2H), 4.49 (d, *J* = 7.2 Hz, 1H), 4.32 (dd, *J* = 10.4, 4.9 Hz, 1H), 4.15- 4.05 (m, 1H), 4.01- 3.94 (m, 1H), 3.94 (brs, 1H), 3.88- 3.86 (m, 2H), 3.81- 3.70 (m, 6H), 3.58- 3.50 (m, 3H), 3.37- 3.32 (m, 3H), 1.87- 1.77 (m, 2H), 1.31 (d, *J* = 6.4 Hz, 3H), 1.22 (d, *J* = 6.4 Hz, 3H).

$^{13}C\{^{1}H\}$ NMR (100 MHz, $CDCl_3$) *δ* 162.0, 156.5, 138.7, 138.2, 137.1, 136.7, 134.7, 133.2, 133.1, 129.0, 128.6, 128.4, 128.28, 128.26, 128.11, 128.08, 127.9, 127.8, 127.6, 127.5, 127.0, 126.8, 126.4, 126.2, 125.9, 125.7, 102.0, 101.1, 100.9, 98.6, 92.6, 82.2, 81.6, 78.4, 78.0, 77.9, 74.8, 74.3, 71.6, 71.4, 69.0, 68.6, 66.6, 66.2, 66.0, 65.8, 57.7, 54.8, 38.8, 29.4, 16.8, 16.2.

HRMS (ESI-TOF) (m/z) calculated for $C_{63}H_{69}Cl_3N_5O_{15}$ $[M+H]^+$: 1240.3850 found 1240.3818.

Compound G5:

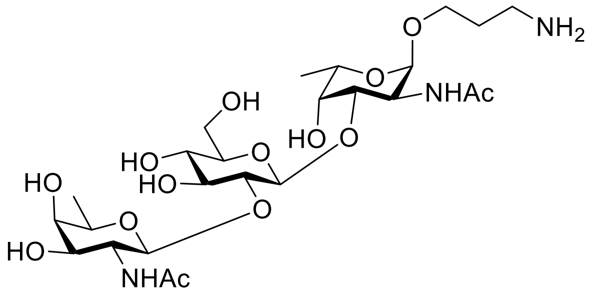

G5

Activated zinc dust (96 mg), acetic anhydride (0.1 mL) and acetic acid (0.05 mL) were added sequentially to the stirring solution of compound **23** (23 mg, 0.0185 mmol) in THF (3 mL). The mixture was stirred overnight at room temperature. After completion of reaction (indicated by TLC), the reaction mixture was diluted with ethyl acetate and zinc was filtered out through a celite pad, then the liquor was directly concentrated under reduced pressure. Toluene azeotrope was performed to remove the remaining acetic acid and kept under high vacuum for the next step. The obtained amide compound was dissolved in MeOH: Ethyl acetate (1:1, 2 mL). To this solution, $Pd(OH)_2/C$ (12 mg) was added and stirred under hydrogen atmosphere (1 atm) for 24 h. The reaction mixture was then filtered through Celite, concentrated, purified by using Sephadex-G25 gel (milli-Q water as an eluent) to afford the target molecule **SSK-M5** (9.8 mg, 87% over two steps) as a sticky colourless liquid.

[α]$_D^{25}$ = -3.523 (c = 0.083, MeOH).

IR (cm^{-1}, $CHCl_3$) ν = 3682, 3097, 1553, 851.

^{1}H NMR (400 MHz, D_2O) δ 4.72 (d, J = 3.6 Hz, 1H), 4.45 (t, J = 7.1 Hz, 2H), 4.08- 4.06 (m, 2H), 3.96 (m, 2H), 3.81- 3.72 (m, 2H), 3.63- 3.52 (m, 5H), 3.37- 3.33 (m, 2H), 3.27- 3.22 (m, 2H), 3.17 (t, J = 9.3 Hz, 1H), 3.00- 2.93 (m, 2H), 1.90- 1.83 (m, 8H), 1.18- 1.15 (m, 6H).

^{13}C{^{1}H} NMR (100 MHz, D_2O) δ 175.0, 174.4, 103.2, 98.9, 96.9, 81.4, 76.1, 75.9, 71.1, 70.9, 70.3, 70.0, 68.7, 68.2, 65.8, 64.7, 60.9, 52.4, 47.9, 37.1, 25.8, 22.3, 21.9, 15.44, 15.40.

HRMS (ESI-TOF) (m/z) calculated for $C_{25}H_{46}N_3O_{14}$ $[M+H]^+$: 612.2974 found 612.2960.

Compound 27

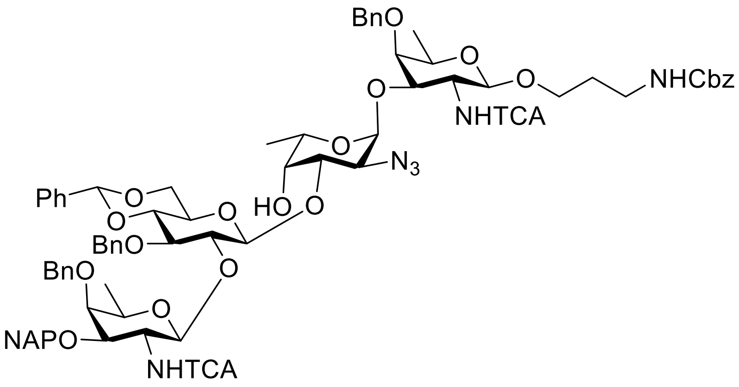

27

D-fucosamine thioglycoside donor **19** (16 mg, 0.0259 mmol) and trisaccharide acceptor **26** (26 mg, 0.0215 mmol) were dissolved in anhydrous CH_2Cl_2 (3 mL), along with the activated molecular sieves (3Å, 30 mg), and the mixture was stirred at room temperature for 30 minutes under nitrogen atmosphere. NIS (11 mg, 0.0518 mmol) was added and the reaction was kept at 0 °C, after 15 mins, TMSOTf (0.9 µL, 0.005 mmol) was added in a dropwise manner. After completion of the reaction, as monitored by TLC, the reaction mixture was neutralized with Et_3N, and filtered through a Celite bed using CH_2Cl_2. The reaction mixture was washed with aq. $Na_2S_2O_3$ solution. The separated organic layer was dried over anhydrous Na_2SO_4, concentrated in vacuo, and purified by column chromatography on silica gel (30% ethyl acetate: pet ether) to afford tetrasaccharide **27** (20 mg, 58%) as a viscous liquid.

[α]$_D^{25}$ = +4.612 (c = 0.585, $CHCl_3$).

IR (cm^{-1}, $CHCl_3$) *ν* = 3417, 2921, 2386, 2171, 1645, 1462, 1376, 1216, 763.

^{1}H NMR (500 MHz, $CDCl_3$) ***δ*** 7.89- 7.82 (m, 5H), 7.55- 7.53 (m, 2H), 7.48- 7.46 (m, 2H), 7.43- 7.36 (m, 12H), 7.35-7.30 (m, 6H), 7.24 (d, *J* = 7.3 Hz, 2H), 7.10 (t, *J* = 7.4 Hz, 2H), 7.03 (t, *J* = 7.3 Hz, 1H), 6.38 (d, *J* = 8.6 Hz, 1H, -NH), 5.56 (s, 1H, PhCH), 5.18- 5.15 (m, 1H), 5.10 (brs, 2H), 5.05 (d, *J* = 11.7 Hz, 1H), 5.02 (d, *J* = 3.6 Hz, 1H), 4.96 (d, *J* = 11.7 Hz, 2H), 4.92 (d, *J* = 3.4 Hz, 2H), 4.79 (d, *J* = 12.0 Hz, 1H), 4.72 (d, *J* = 12.0 Hz, 1H), 4.68- 4.59 (m, 3H), 4.46- 4.42 (m, 2H), 4.36- 4.32 (m, 2H), 4.17- 4.11 (m, 1H), 3.98- 3.93 (m, 2H), 3.87 (brs, 1H), 3.78- 3.76 (m, 3H), 3.70 (brs, 2H), 3.68 (d, *J* = 2.9 Hz, 2H), 3.58 (d, *J* = 1.9 Hz, 2H), 3.57- 3.55 (m, 2H), 3.46- 3.41 (m, 2H), 3.38- 3.34 (m, 1H), 3.29- 3.26 (m, 2H), 1.79- 1.77 (m, 2H), 1.35 (d, *J* = 6.3 Hz, 3H), 1.31 (d, *J* = 6.4 Hz, 3H), 1.18 (d, *J* = 6.6 Hz, 3H).

^{13}C{^{1}H} NMR (125 MHz, $CDCl_3$) ***δ*** 162.3, 162.0, 156.5, 138.8, 138.6, 138.2, 137.0, 136.8, 134.7, 133.2, 133.1, 129.1, 128.6, 128.5, 128.38, 128.35, 128.32, 128.25, 128.1, 128.0, 127.9, 127.8, 127.7, 127.6, 127.5, 127.3, 127.2, 126.9, 126.8, 126.5, 126.4, 126.3, 125.9, 125.7, 101.8, 101.2, 100.8, 100.3, 99.1, 92.6, 92.5, 82.3, 81.6, 80.5, 78.4, 78.0, 77.9, 75.8, 74.8, 74.3, 74.1, 71.6, 71.4, 70.9, 68.7, 67.4, 66.8, 66.5, 65.9, 57.9, 56.0, 54.6, 38.1, 29.7, 17.0, 16.7, 16.2.

HRMS (ESI-TOF) (m/z) calculated for $C_{78}H_{88}Cl_6N_7O_{19}$ $[M+NH_4]^+$: 1640.4236, found 1640.4247.

Compound G7:

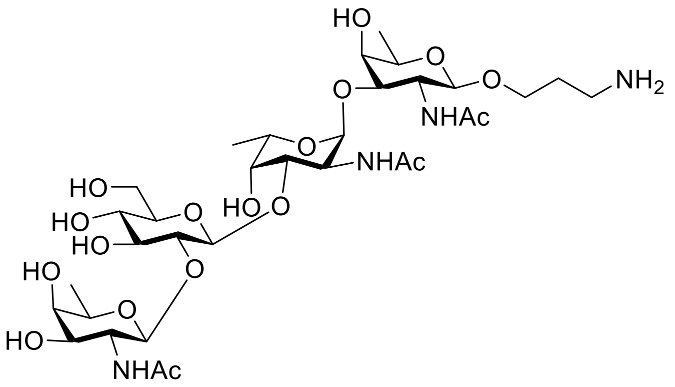

G7

Activated zinc dust (64 mg), acetic anhydride (0.1 mL), and acetic acid (0.05 mL) were added sequentially to the stirring solution of compound **26** (20 mg, 0.0123 mmol) in THF (3 mL). The mixture was stirred overnight for 10 h at room temperature. After completion of the reaction (indicated by TLC), the reaction mixture was diluted with ethyl acetate, and zinc was

filtered out through a celite pad, then the liquor was directly concentrated under reduced pressure. Toluene azeotrope was performed to remove the remaining acetic acid and kept under a high vacuum for the next step. The obtained amide compound was dissolved in MeOH (2 mL). To this solution, $Pd(OH)_2/C$ (8 mg) was added and stirred under a hydrogen atmosphere (1 atm, balloon) for 24 h. The reaction mixture was then filtered through Celite, concentrated, and purified by using Sephadex-G25 gel (milli-Q water as an eluent) to afford the target molecule **G7** (8 mg, 82% over two steps) as a sticky colourless liquid.

$[\alpha]_D^{25}$ = -51.276 (*c* = 0.085, MeOH).

IR (cm^{-1}, $CHCl_3$) *ν* = 3552, 2839, 1642, 1263, 1017, 846, 766, 602.

^{1}H NMR (600 MHz, D_2O) *δ* 4.94 (brs, 1H), 4.51 (m, 2H), 4.30 (J = 8.5 Hz, 1H), 4.11 – 4.10 (m, 2H), 4.03 (d, J = 7.1 Hz, 2H), 3.89- 3.80 (m, 2H), 3.70- 3.65 (m, 8H), 3.62 (brs, 1H), 3.32- 3.27 (m, 2H), 2.99 (t, J = 6.9 Hz, 2H), 1.96 (s, 3H), 1.94 (s, 3H), 1.92 (s, 3H), 1.86 (m, 2H), 1.24 (d, J = 6.9 Hz, 3H), 1.21- 1.18 (m, 6H).

^{13}C{^{1}H} NMR (100 MHz, D_2O) *δ* 175.1, 174.4, 174.2, 103.3, 101.6, 99.0, 98.8, 81.5, 76.6, 76.1, 75.9, 75.5, 71.2, 71.0, 70.7, 70.4, 70.0, 68.2, 67.9, 66.3, 60.9, 52.5, 51.4, 48.9, 47.9, 37.7, 26.7, 22.4, 22.2, 15.5, 15.3.

HRMS (ESI-TOF) m/z: calculated for $C_{33}H_{59}N_4O_{18}$ $[M + H]^+$: 799.3819; found 799.3802.

Compound 29:

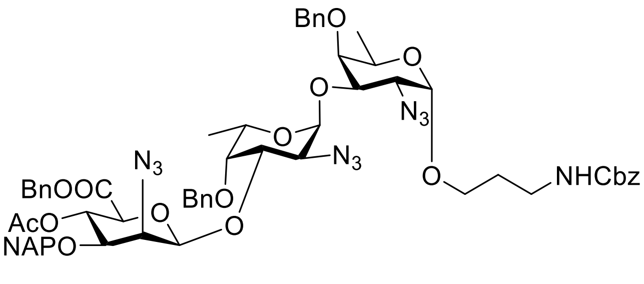

29

N-(phenyl) trifluoroacetimidate donor **28** (73 mg, 0.11 mmol) and disaccharide acceptor **16** (0.20 mg, 0.027 mmol) were dissolved in anhydrous CH_2Cl_2 (4 mL). Activated molecular sieves (3Å, 100 mg) were added, and the mixture was stirred at room temperature for 30 minutes under nitrogen. The mixture was cooled to -60 °C and TMSOTf (2 µL, 0.011 mmol) was added dropwise. After 1 h, the reaction mixture was warmed to -40 °C, and was kept for

stirring at the same temperature for the next 2 h. The entire disaccharide acceptor got transformed into a less polar product (as determined by TLC results). Triethylamine was used to neutralize the mixture, and then CH_2Cl_2 was used to filter it through a Celite bed. Filtrate was dried over anhydrous Na_2SO_4, concentrated, and purified by column chromatography on silica gel (30% ethyl acetate: pet ether) afforded trisaccharide **29** (18 mg, 56%) as white foam.

[α]$_D^{25}$ = +4.776 (c = 0.600, $CHCl_3$).

IR (cm^{-1}, $CHCl_3$) ν = 3421, 2925, 1725, 1462, 1377, 1215, 763.

^{1}H NMR (400 MHz, $CDCl_3$) δ 7.82- 7.73 (m, 5H), 7.48- 7.43 (m, 5H), 7.32- 7.25 (m, 17H), 5.44 (t, J = 9.1 Hz, 1H), 5.32 (brs, 1H), 5.13- 5.06 (m, 3H), 5.02 (d, J = 2.9 Hz, 2H), 4.90 (d, J = 3.2 Hz, 1H), 4.78 (t, J = 10.7 Hz, 3H), 4.66- 4.62 (m, 2H), 4.57- 4.53 (m, 2H), 4.22 (dd, J = 10.6, 2.6 Hz, 1H), 4.00- 3.97 (m, 1H), 3.92- 3.89 (m, 2H), 3.82 (d, J = 8.9 Hz, 2H), 3.72 (dd, J = 10.3, 3.2 , Hz, 1H), 3.64 (brs, 1H), 3.59- 3.56 (m, 1H), 3.53- 3.47 (m, 3H), 3.37- 3.28 (m, 2H), 1.83 (s, 3H, -OAc), 1.83- 1.73 (m, 2H), 1.20 (d, J = 6.5 Hz, 3H), 1.14 (d, J = 6.4 Hz, 3H).

^{13}C{^{1}H} NMR (100 MHz, $CDCl_3$) δ 169.2, 166.4, 156.5, 138.4, 138.0, 136.7, 135.0, 134.5, 133.12, 133.10, 128.7, 128.53, 128.49, 128.43, 128.38, 128.13, 128.06, 127.9, 127.82, 127.78, 127.70, 127.66, 126.8, 126.4, 126.3, 125.6, 99.7, 98.1, 97.4, 79.5, 75.6, 75.4, 75.1, 73.6, 72.3, 67.6, 67.4, 67.0, 66.9, 66.6, 61.3, 60.6, 58.7, 39.3, 29.2, 20.6, 16.8, 16.7.

HRMS (ESI-TOF) (m/z) calculated for $C_{63}H_{69}N_{10}O_{15}$ $[M+H]^+$: 1205.4938 found 1205.4932.

Compound G12:

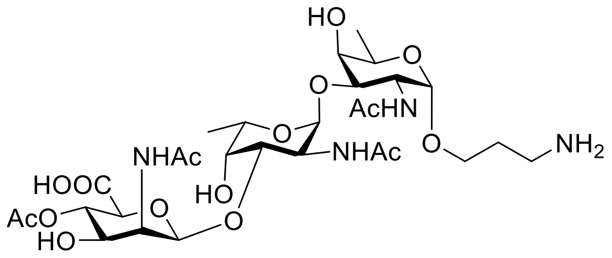

G12

Activated zinc dust (73 mg), acetic anhydride (0.2 mL), and acetic acid (0.1 mL) were added sequentially to the stirring solution of fully protected trisaccharide **28** (17 mg, 0.0141 mmol) in THF (3 mL). The mixture was stirred overnight at room temperature. After completion of the reaction (indicated by TLC), the reaction mixture was diluted with ethyl acetate, and zinc

was filtered out through a celite pad, then the liquor was directly concentrated under reduced pressure. Toluene azeotrope was performed to remove the remaining acetic acid and kept under high vacuum for the next step. The obtained amide compound (80 mg, 0.127 mmol) was dissolved in tBuOH/CH_2Cl_2/0.1M AcOH (2/1/1, v/v/v, 4 mL). To this solution, $Pd(OH)_2$/C (24 mg) was added and stirred under hydrogen atmosphere (1 atm, balloon) for 24 h. The reaction mixture was then filtered through Celite, concentrated, purified by using Sephadex-G25 gel (milli-Q water as an eluent) and lyophilized to obtain the target molecule G**12** (7 mg, 74% over two steps) as a sticky colorless liquid.

$[\alpha]_D^{25}$ = -5.034 (c = 0.233, MeOH).

IR (cm^{-1}, $CHCl_3$) ν = 3672, 3152, 2993, 2561, 1714, 1577, 1265, 1126, 845, 567.

^1H NMR (500 MHz, D_2O) δ 4.95 (t, J = 10.0 Hz, 1H), 4.92 (brs, 1H), 4.88 (brs, 1H), 4.73 (1H), 4.45 (d, J = 3.7 Hz, 1H), 4.19 (dd, J = 11.0, 3.7 Hz, 1H), 4.09 (brs, 2H), 4.01- 3.94 (m, 4H), 3.89 (d, J = 10.0 Hz, 1H), 3.84 (dd, J = 11.1, 2.9 Hz, 1H), 3.72 (d, J = 2.6 Hz, 1H), 3.70- 3.65 (m, 1H), 3.45- 3.42 (m, 1H), 3.01 (t, J = 7.1 Hz, 2H), 2.04 (s, 3H), 1.97 (s, 6H), 1.93- 1.88 (m, 5H), 1.15- 1.13 (m, 6H).

$^{13}C\{^1H\}$ NMR (125 MHz, D_2O) δ 175.6, 173.95, 173.85, 173.2, 173.0, 99.0, 97.2, 95.2, 74.1, 73.3, 73.1, 71.2,70.0, 69.5, 67.6, 66.9, 66.6, 65.0, 53.0, 48.5, 47.7, 37.2, 26.8, 22.3, 21.98, 21.96, 20.3, 15.5, 15.3.

HRMS (ESI-TOF) (m/z) calculated for $C_{29}H_{49}N_4O_{16}$ $[M+H]^+$: 709.3138 found 709.3137.

Compounds **G8**, **G9**, **G13**, **G14**, **G15**, and **G16** had been synthesized in our lab in earlier studies [2-5] and were incorporated here for glycan microarray evaluations.

Glycan Microarray

A non-contact sciFLEXARRAYER S12 microarray spotter (Scienion, Berlin, Germany) was used to immobilize the glycans (0.1 mM in 50 mM sodium phosphate buffer, pH 8.5) in 56 identical fields on *N*-hydroxysuccinimide (NHS) ester-activated hydrogel glass slides (NEXTERION® Slide H, Schott). The remaining NHS groups of the slides were quenched with ethanolamine after incubation overnight in a humidified box. The slides were blocked with 1% (w/v) bovine serum albumin (BSA) in phosphate buffered saline (PBS) and a 64 well incubation gasket (FlexWell Grid, Grace Bio Labs) was attached. The slides were incubated with the sera of patients infected with *P. aeruginosa, A. baumanii* or *S. aureus* or pooled sera

from healthy humans (Sigma-Aldrich, Cat. H4522) diluted 1:100 in 1% BSA-PBS for 1 h at 37° C. After three washes with PBS containing 0.1% (v/v) Tween-20 (PBS-T) the slides were incubated with goat anti-human IgG Fc-AF647 (SouthernBiotech, Cat. 2048-31) diluted 1:400 for 1 h at 37°C. The slides were rinsed twice with PBS-T. After the gasket was removed, the slides were washed once with PBS and once with water. Once dried, the slides were scanned using an InnoScan 1100 Fluorescence Scanner (Innopsys). Intensity measurements were analyzed using Mapix 9.1.0 (Innopsys).

Study participant details

Patients infected with *A. baumannii* consented under institutional review board (IRB)- and SBU Human Subjects Committee-approved protocols (IRB no. 896845 and 851803). Serum samples of 10 patients with cystic fibrosis chronically infected with *Pseudomonas aeruginosa* and 10 patients chronically infected with *Staphylococcus aureus* were obtained as part of an observational cohort study conducted at Charité – Universitätsmedizin Berlin, Germany. The study was approved by the Ethics Committee of Charité – Universitätsmedizin Berlin (EA2/016/18) and conducted in accordance with the Declaration of Helsinki and guidelines of Good Clinical Practice. Written informed consent was obtained from all participants.

Data Analysis

Results were analysed using GraphPad Prism 10.4.0 (GraphPad Software, Inc.). Statistical significance was determined as described in the figure legends. $P < 0.033$ was considered statistically significant. All graphs depict mean ± SEM.

Reference

1. S. S. Kulkarni, J. Gervay-Hague, *In Handbook of Chemical Glycosylation: Advances in Stereoselectivity and Therapeutic Relevance*; Wiley-VCH:, **2008**; pp 59– 93.

2. D. Rai, S. S. Kulkarni, Total Synthesis of Conjugation-Ready Tetrasaccharide Repeating Units of a Multidrug-Resistant Pathogen *Acinetobacter baumannii* Strain 34 and O5. *Org. Lett.* **2023**, *25*, 8332–8337.

3. A. Behera, D. Rai, S. S. Kulkarni, Total Syntheses of Conjugation-Ready Trisaccharide Repeating Units of *Pseudomonas aeruginosa* O11 and *Staphylococcus aureus* Type 5 Capsular Polysaccharide for Vaccine Development. *J. Am. Chem. Soc.* **2020**, 142, 456– 467.

4. A. A. Shirsat, D. Rai, B. K. Ghotekar, S. S. Kulkarni, Total Synthesis of Trisaccharide Repeating Unit of *Staphylococcus aureus* Strain M. *Org. Lett.* **2023**, *25*, 2913– 2917.

5. S. Biswas, B. K. Ghotekar, S. S. Kulkarni, Total Synthesis of the All-Rare Sugar-Containing Pentasaccharide Repeating Unit of the O-Polysaccharide of Plesiomonas shigelloides Strain 302-73 (Serotype O1). *Org. Lett.* **2021**, *23*, 6137– 6142.

SSK-34-AKM-395-1H

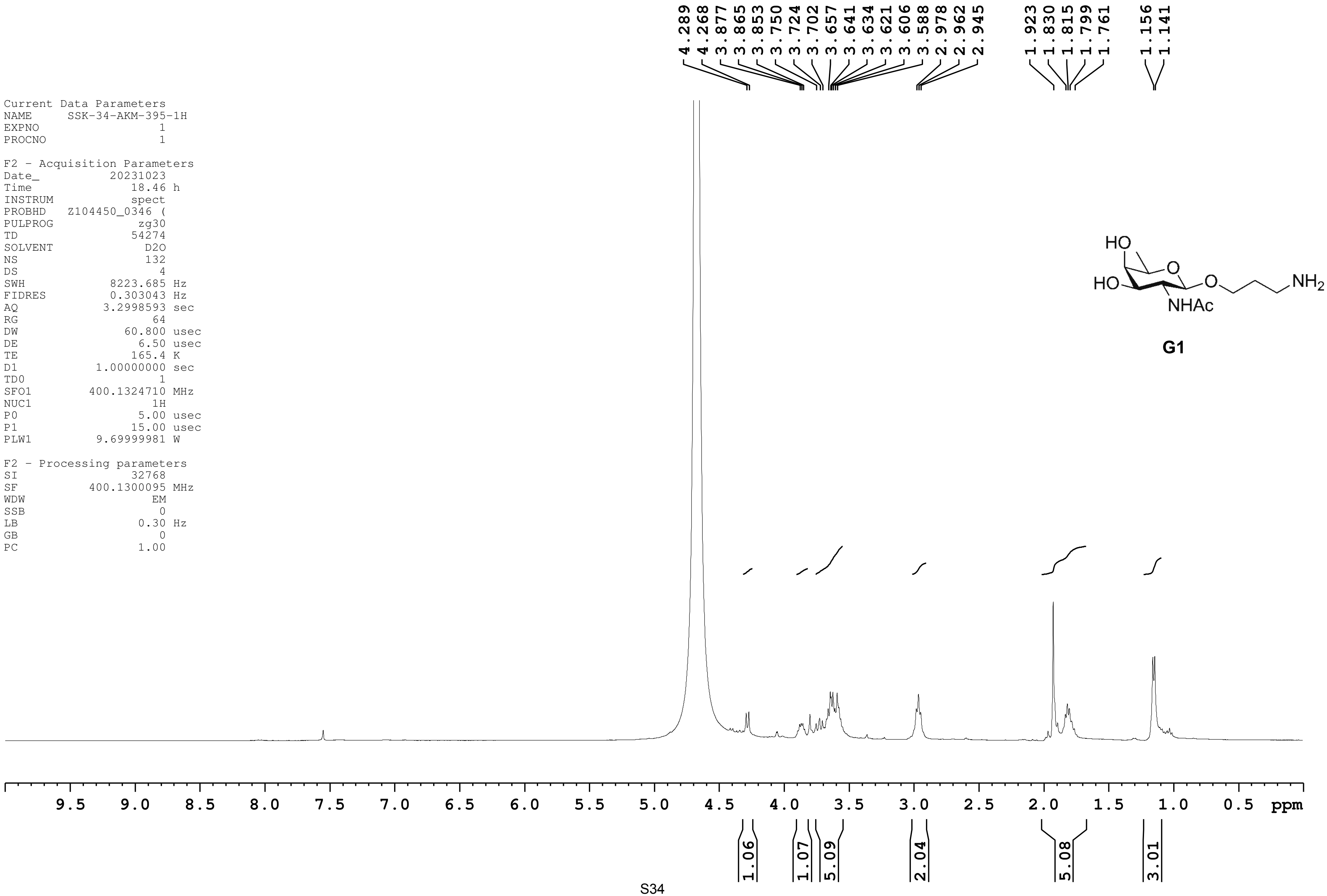


```
Current Data Parameters
NAME      SSK-34-AKM-395-1H
EXPNO                    1
PROCNO                   1

F2 - Acquisition Parameters
Date_             20231023
Time                 18.46 h
INSTRUM              spect
PROBHD   Z104450_0346 (
PULPROG               zg30
TD                   54274
SOLVENT                D2O
NS                     132
DS                       4
SWH               8223.685 Hz
FIDRES            0.303043 Hz
AQ               3.2998593 sec
RG                      64
DW                  60.800 usec
DE                    6.50 usec
TE                   165.4 K
D1              1.00000000 sec
TD0                      1
SFO1           400.1324710 MHz
NUC1                    1H
P0                    5.00 usec
P1                   15.00 usec
PLW1            9.69999981 W

F2 - Processing parameters
SI                   32768
SF             400.1300095 MHz
WDW                     EM
SSB                      0
LB                    0.30 Hz
GB                       0
PC                    1.00
```


G1

SSK-34-AKM-395-13C

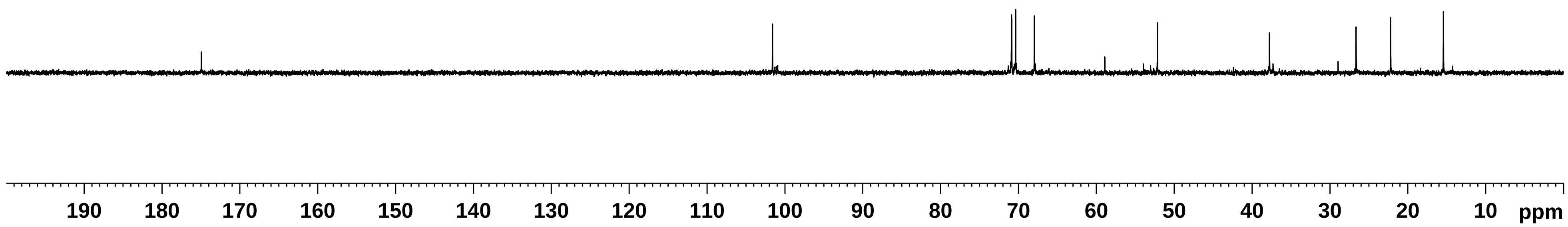


```
Current Data Parameters
NAME     SSK-34-AKM-395-13C
EXPNO                   2
PROCNO                  1

F2 - Acquisition Parameters
Date_          20231024
Time               5.04 h
INSTRUM           spect
PROBHD   Z104450_0346 (
PULPROG         zgpg30
TD               65536
SOLVENT            D2O
NS                7000
DS                   0
SWH          26041.666 Hz
FIDRES        0.794729 Hz
AQ           1.2582912 sec
RG                1030
DW              19.200 usec
DE                6.50 usec
TE               166.5 K
D1          1.00000000 sec
D11         0.03000000 sec
TD0                  1
SFO1       100.6238364 MHz
NUC1               13C
P0                3.33 usec
P1               10.00 usec
PLW1       47.00000000 W
SFO2       400.1316005 MHz
NUC2                1H
CPDPRG[2        waltz16
PCPD2            90.00 usec
PLW2        9.69999981 W
PLW12       0.26944000 W
PLW13       0.13552999 W

F2 - Processing parameters
SI               32768
SF         100.6127690 MHz
WDW                 EM
SSB                  0
LB                1.00 Hz
GB                   0
PC                1.40
```

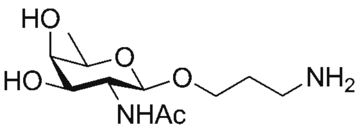

G1

SSK-34-AKM-395-DEPT

```
Current Data Parameters
NAME     SSK-34-AKM-395-DEPT
EXPNO                  2
PROCNO                 1

F2 - Acquisition Parameters
Date_          20231023
Time              20.15 h
INSTRUM           spect
PROBHD   Z104450_0346 (
PULPROG         dept135
TD                65536
SOLVENT             D2O
NS                 2377
DS                    0
SWH           27777.777 Hz
FIDRES         0.847710 Hz
AQ            1.1796480 sec
RG                  203
DW               18.000 usec
DE                 6.50 usec
TE                165.9 K
CNST2       145.0000000
D1           1.00000000 sec
D2           0.00344828 sec
D12          0.00002000 sec
TD0                   1
SFO1        100.6242389 MHz
NUC1                13C
P1                10.00 usec
P2                20.00 usec
PLW1        47.00000000 W
SFO2        400.1316005 MHz
NUC2                 1H
CPDPRG[2        waltz16
P3                15.00 usec
P4                30.00 usec
PCPD2             90.00 usec
PLW2         9.69999981 W
PLW12        0.26944000 W

F2 - Processing parameters
SI                32768
SF          100.6127690 MHz
WDW                  EM
SSB                   0
LB                 1.00 Hz
GB                    0
PC                 1.40
```

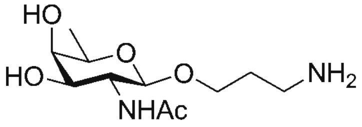

G1

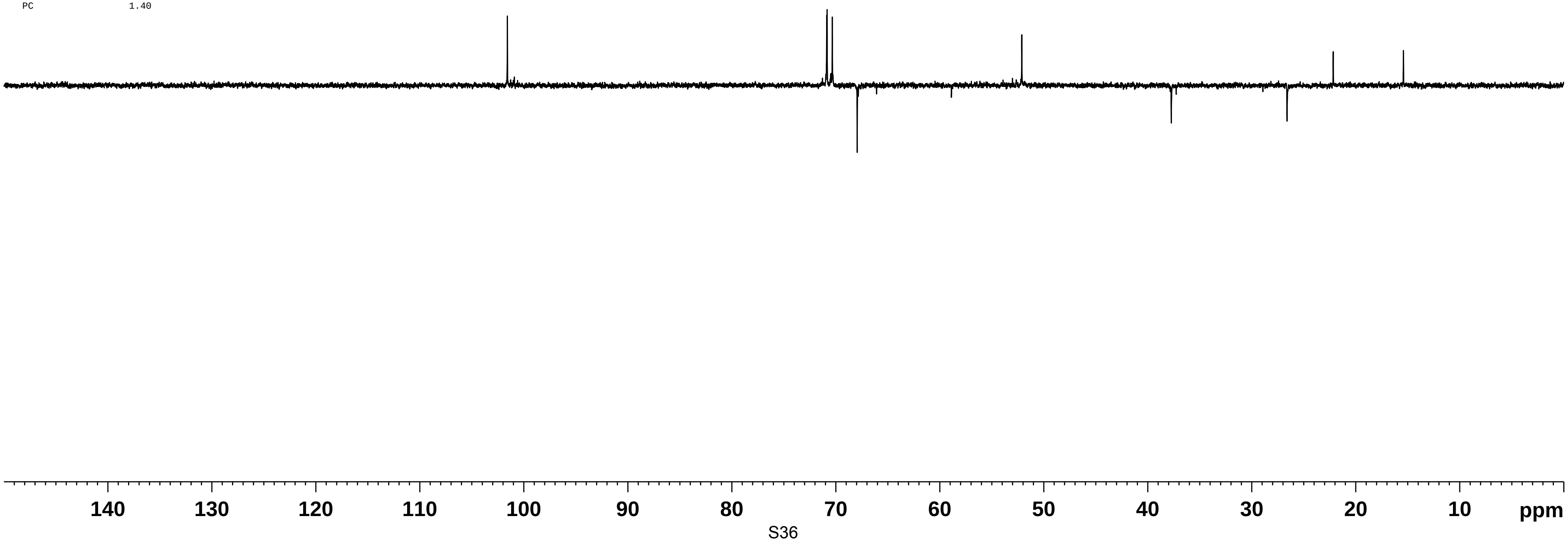

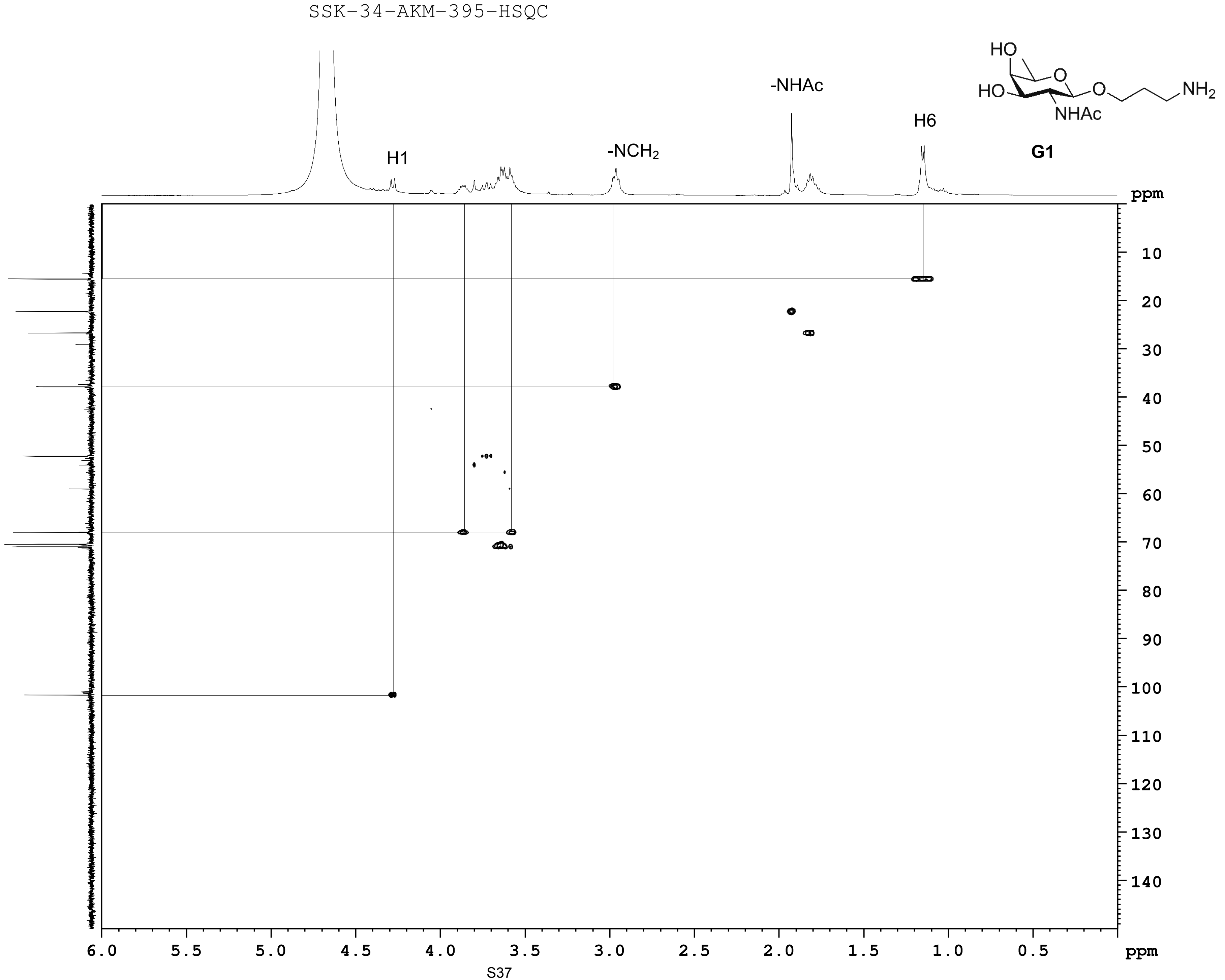


```
Current Data Parameters
NAME      SSK-34-AKM-395-HSQC
EXPNO                    3
PROCNO                   1

F2 - Acquisition Parameters
Date_             20231024
Time                6.31 h
INSTRUM              spect
PROBHD   Z104450_0346 (
PULPROG           hsqcetgp
TD                    2048
SOLVENT                D2O
NS                      16
DS                       2
SWH               4006.410 Hz
FIDRES            3.912510 Hz
AQ               0.2555904 sec
RG                    2050
DW                 124.800 usec
DE                    6.50 usec
TE                   166.5 K
CNST2          145.0000000
D0              0.00000300 sec
D1              1.00000000 sec
D4              0.00172414 sec
D11             0.03000000 sec
D16             0.00020000 sec
IN0             0.00002480 sec
TDav                     1
ZGOPTNS
SFO1           400.1319978 MHz
NUC1                    1H
P1                   15.00 usec
P2                   30.00 usec
PLW1            9.69999981 W
SFO2           100.6228412 MHz
NUC2                   13C
CPDPRG[2              garp
P3                   10.00 usec
P4                   20.00 usec
PCPD2                80.00 usec
PLW2           47.00000000 W
PLW12           0.73438001 W
GPNAM[1]          SINE.100
GPZ1                 80.00 %
GPNAM[2]          SINE.100
GPZ2                 20.10 %
P16                1000.00 usec

F1 - Acquisition parameters
TD                     256
SFO1              100.6228 MHz
FIDRES          157.510086 Hz
SW                 200.365 ppm
FnMODE       Echo-Antiecho

F2 - Processing parameters
SI                    2048
SF             400.1300257 MHz
WDW                  QSINE
SSB                      2
LB                       0 Hz
GB                       0
PC                    1.40

F1 - Processing parameters
SI                    1024
MC2           echo-antiecho
SF             100.6127655 MHz
WDW                  QSINE
SSB                      2
LB                       0 Hz
GB                       0
```

SSK-34-AKM-411-1H

Current Data Parameters
NAME SSK-34-AKM-411-1H
EXPNO 1
PROCNO 1

F2 - Acquisition Parameters
Date_ 20240316
Time 18.43 h
INSTRUM spect
PROBHD Z104450_0346 (
PULPROG zg30
TD 54274
SOLVENT D2O
NS 25
DS 0
SWH 8223.685 Hz
FIDRES 0.303043 Hz
AQ 3.2998593 sec
RG 36
DW 60.800 usec
DE 6.50 usec
TE -2946.8 K
D1 1.00000000 sec
TD0 1
SFO1 400.1324710 MHz
NUC1 1H
P0 5.00 usec
P1 15.00 usec
PLW1 9.69999981 W

F2 - Processing parameters
SI 32768
SF 400.1300095 MHz
WDW EM
SSB 0
LB 0.30 Hz
GB 0
PC 1.00

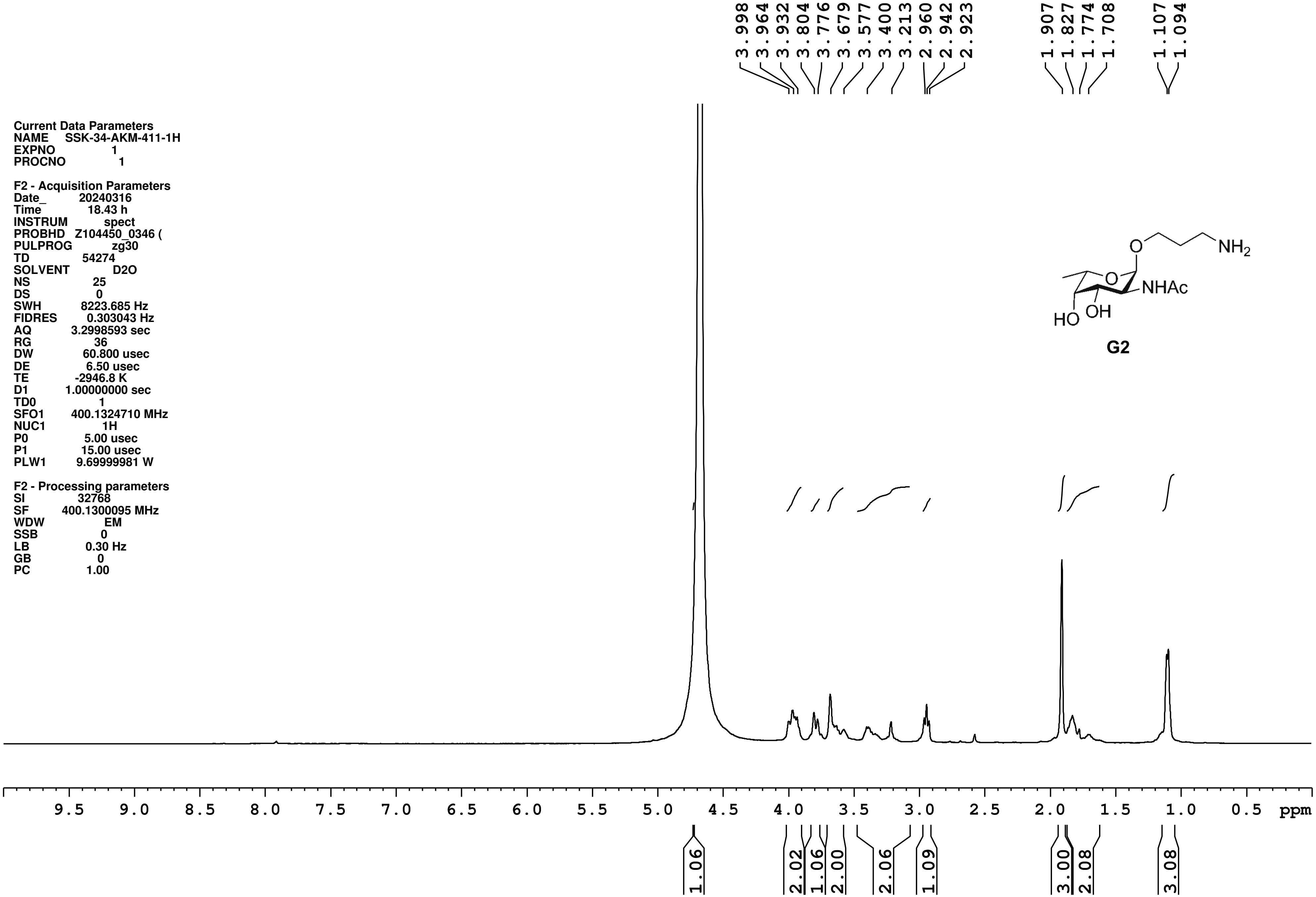

G2

SSK-34-AKM-411-13C

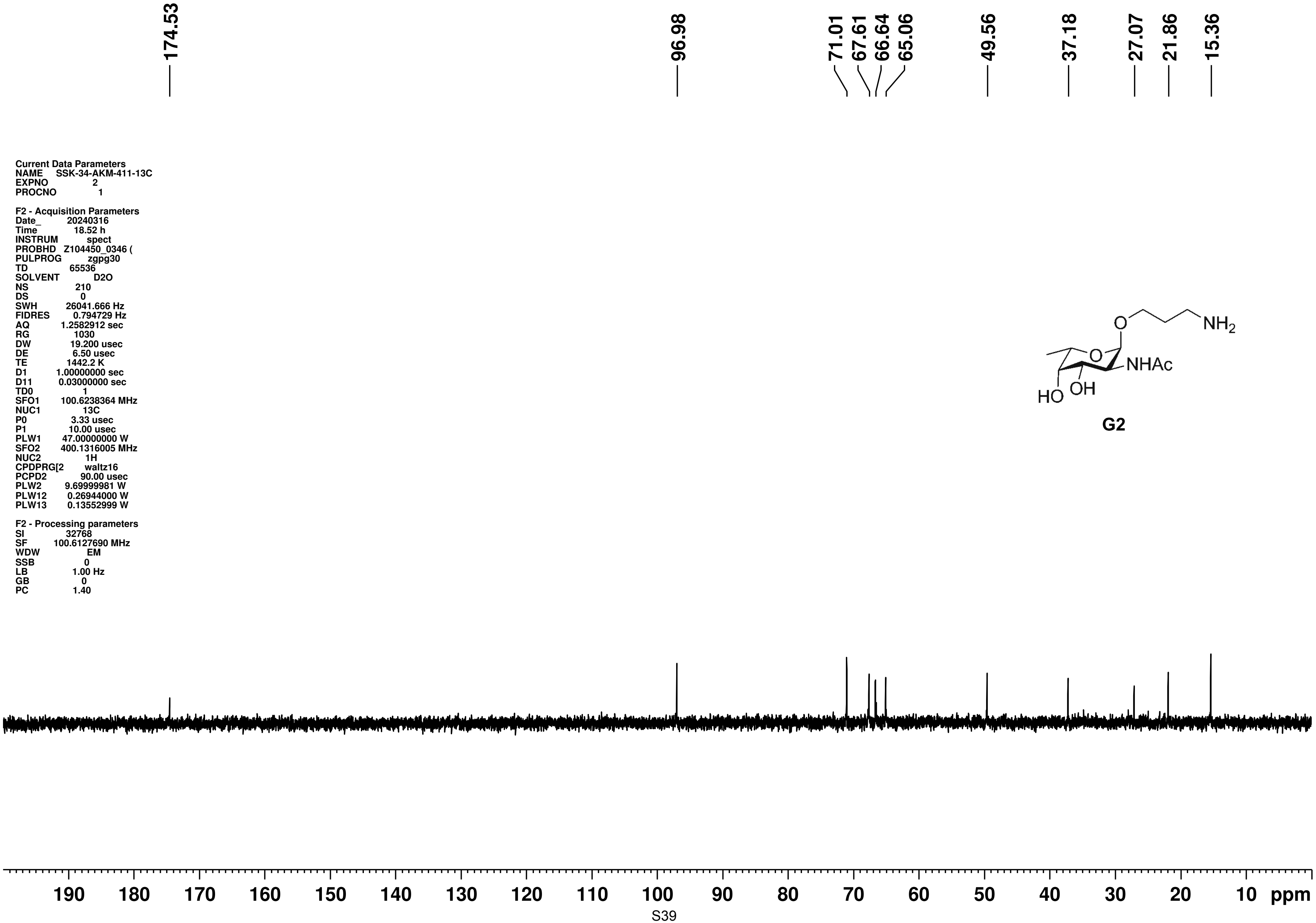

SSK-34-AKM-411-DEPT

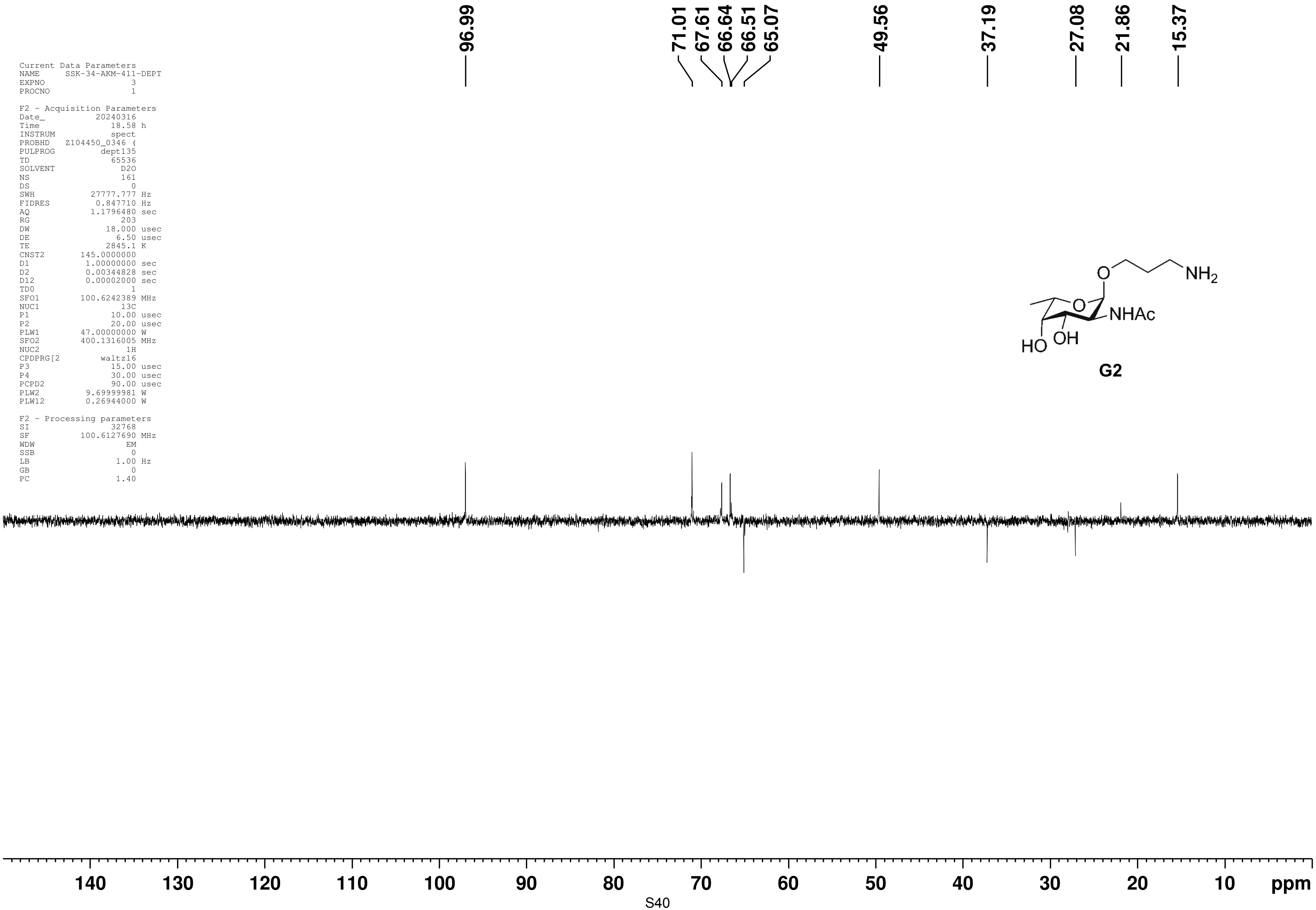

G2

SSK-34-AKM-411-HSQC

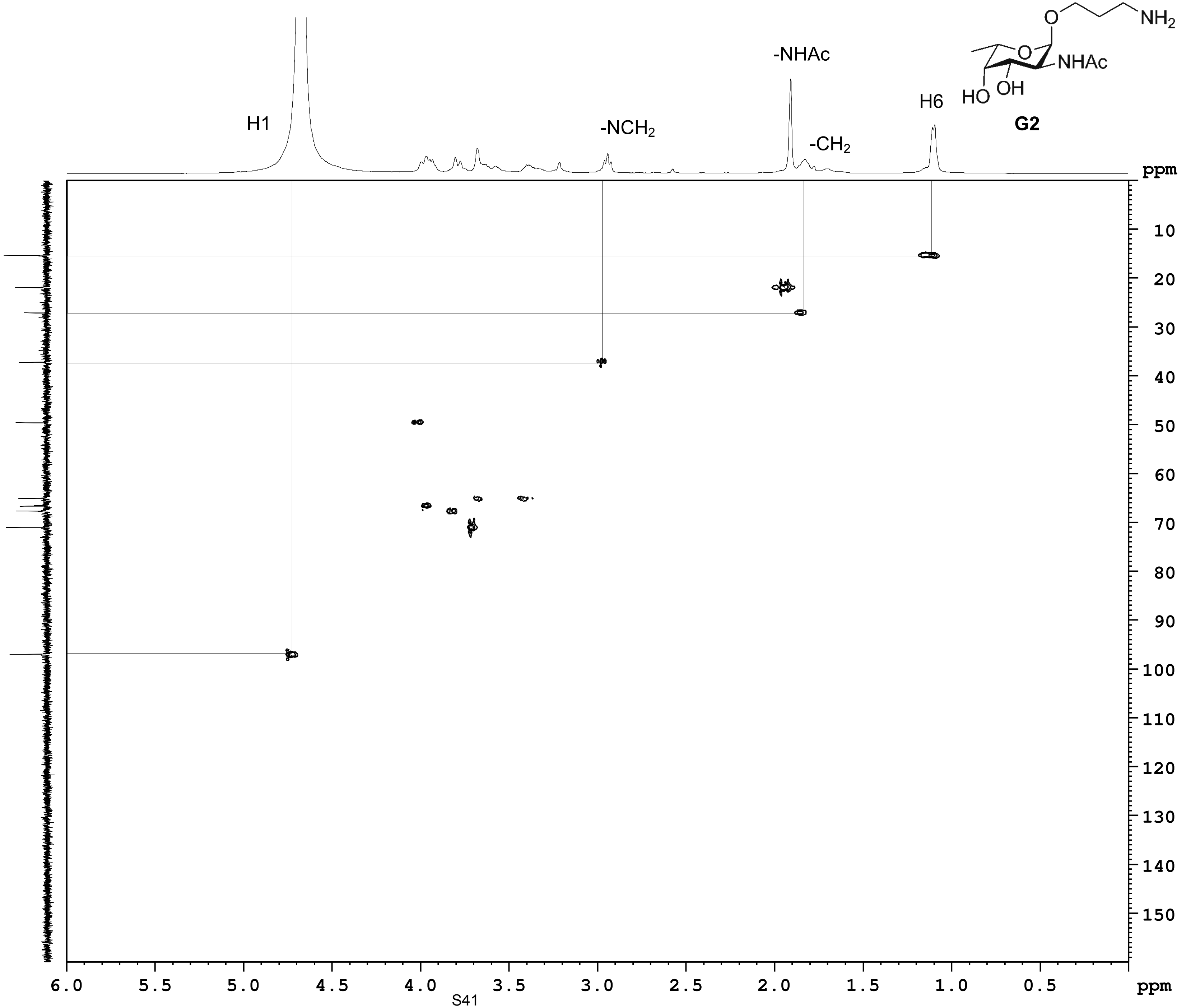


```
Current Data Parameters
NAME      SSK-34-AKM-411-HSQC
EXPNO                    5
PROCNO                   1

F2 - Acquisition Parameters
Date_             20240316
Time                 19.11 h
INSTRUM              spect
PROBHD    Z104450_0346 (
PULPROG           hsqcetgp
TD                    2048
SOLVENT                D2O
NS                       2
DS                       0
SWH               2439.024 Hz
FIDRES            2.381860 Hz
AQ               0.4198400 sec
RG                    2050
DW                 205.000 usec
DE                    6.50 usec
TE                  1174.2 K
CNST2          145.0000000
D0              0.00000300 sec
D1              1.00000000 sec
D4              0.00172414 sec
D11             0.03000000 sec
D16             0.00020000 sec
IN0             0.00002480 sec
TDav                     1
ZGOPTNS
SFO1           400.1311869 MHz
NUC1                    1H
P1                   15.00 usec
P2                   30.00 usec
PLW1            9.69999981 W
SFO2           100.6227950 MHz
NUC2                   13C
CPDPRG[2              garp
P3                   10.00 usec
P4                   20.00 usec
PCPD2                80.00 usec
PLW2           47.00000000 W
PLW12           0.73438001 W
GPNAM[1]          SINE.100
GPZ1                 80.00 %
GPNAM[2]          SINE.100
GPZ2                 20.10 %
P16                1000.00 usec

F1 - Acquisition parameters
TD                     256
SFO1              100.6228 MHz
FIDRES          157.510086 Hz
SW                 200.365 ppm
FnMODE      Echo-Antiecho

F2 - Processing parameters
SI                    2048
SF             400.1300000 MHz
WDW                  QSINE
SSB                      2
LB                       0 Hz
GB                       0
PC                    1.40

F1 - Processing parameters
SI                    1024
MC2         echo-antiecho
SF             100.6127690 MHz
WDW                  QSINE
SSB                      2
LB                       0 Hz
GB                       0
```

SSK-34-AKM-469-1H

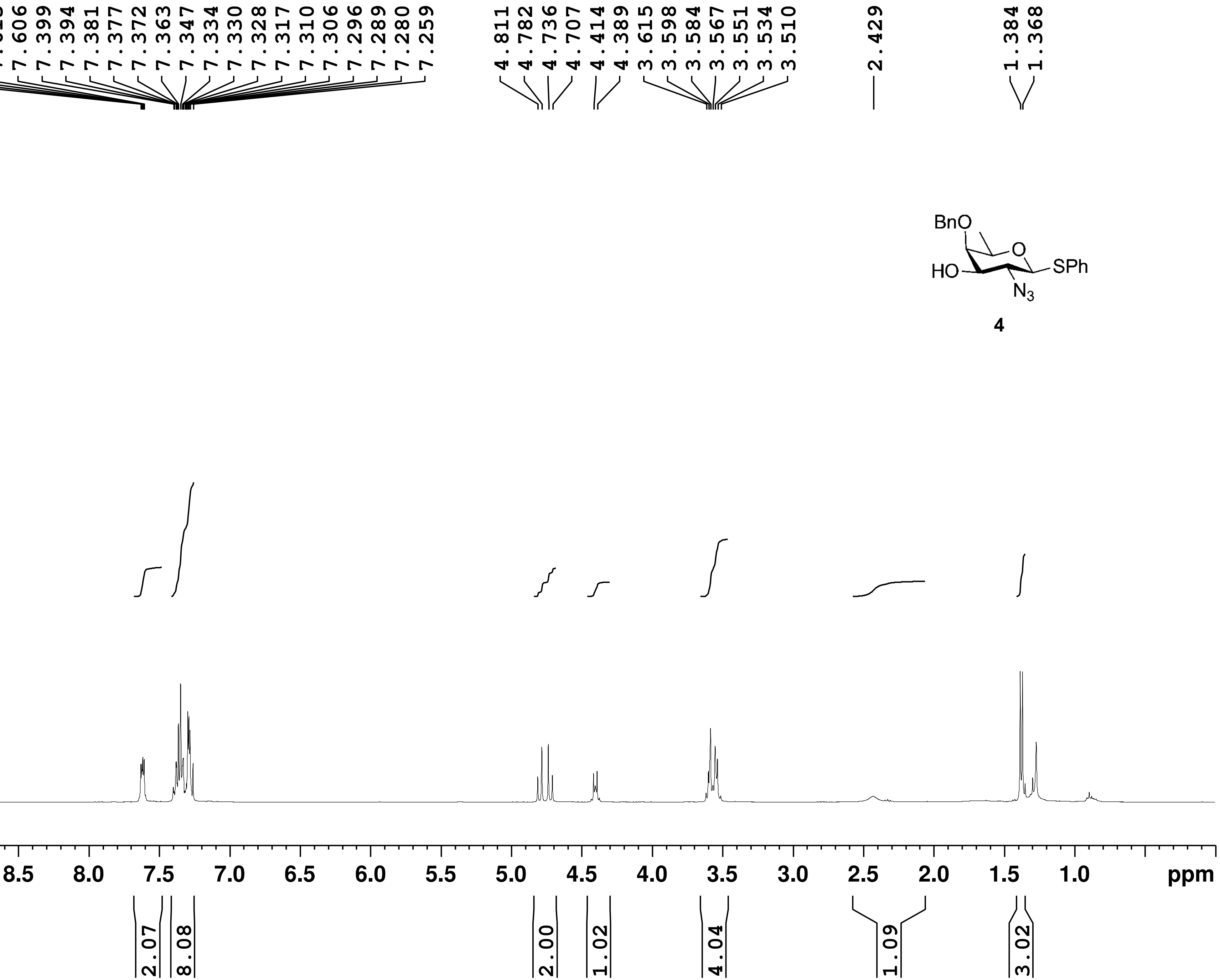

Current Data Parameters
NAME SSK-34-AKM-469-1H
EXPNO 1
PROCNO 1

F2 - Acquisition Parameters
Date_ 20240409
Time 16.54 h
INSTRUM spect
PROBHD Z104450_0346 (
PULPROG zg30
TD 54274
SOLVENT CDCl3
NS 9
DS 0
SWH 8223.685 Hz
FIDRES 0.303043 Hz
AQ 3.2998593 sec
RG 64
DW 60.800 usec
DE 6.50 usec
TE 2693.8 K
D1 1.00000000 sec
TD0 1
SFO1 400.1324710 MHz
NUC1 1H
P0 5.00 usec
P1 15.00 usec
PLW1 9.69999981 W

F2 - Processing parameters
SI 32768
SF 400.1300095 MHz
WDW EM
SSB 0
LB 0.30 Hz
GB 0
PC 1.00

4

SSK-34-AKM-469-13C

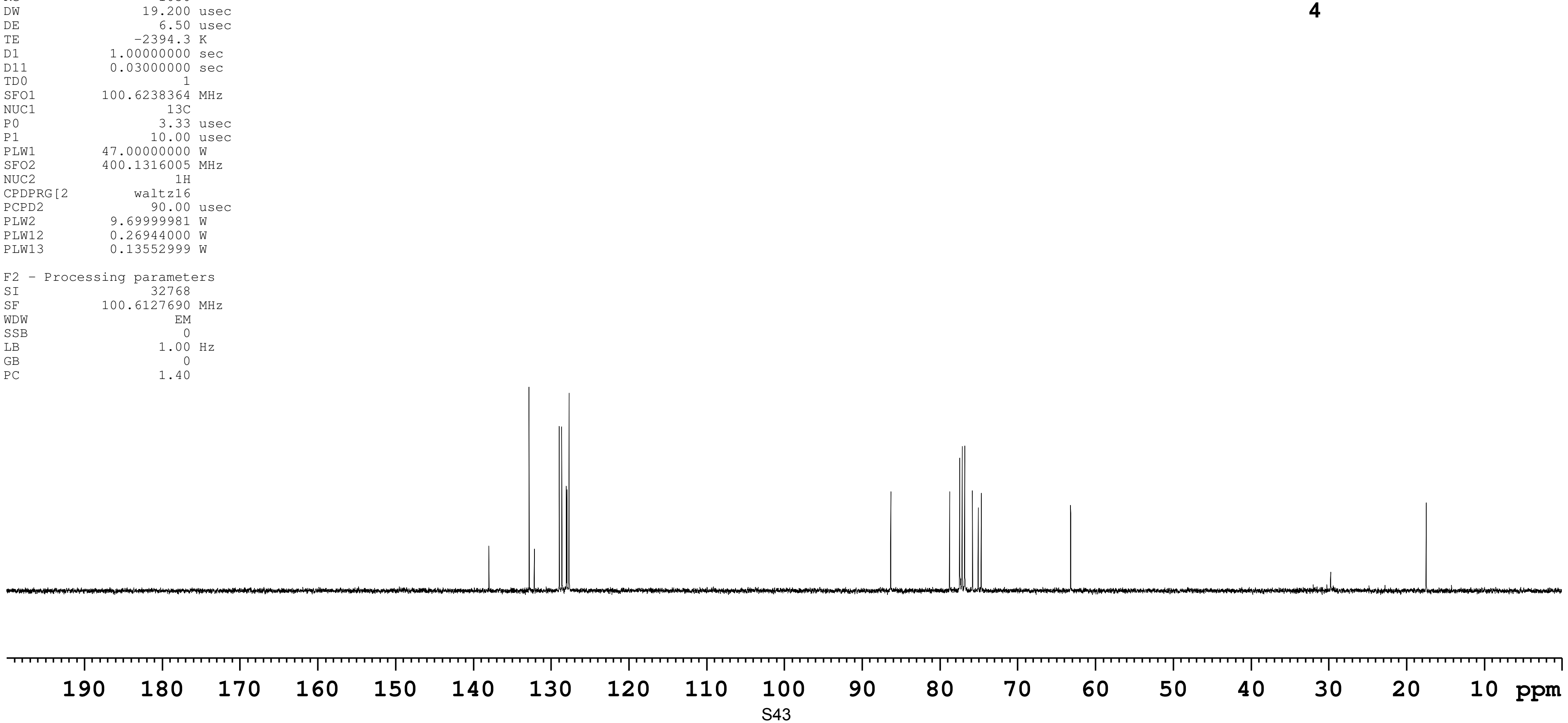

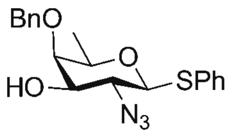

4

SSK-34-AKM-469-DEPT

Current Data Parameters
NAME SSK-34-AKM-469-DEPT
EXPNO 3
PROCNO 1

F2 - Acquisition Parameters
Date_ 20240409
Time 17.00 h
INSTRUM spect
PROBHD Z104450_0346 (
PULPROG dept135
TD 65536
SOLVENT CDCl3
NS 58
DS 0
SWH 27777.777 Hz
FIDRES 0.847710 Hz
AQ 1.1796480 sec
RG 203
DW 18.000 usec
DE 6.50 usec
TE 630.2 K
CNST2 145.0000000
D1 1.00000000 sec
D2 0.00344828 sec
D12 0.00002000 sec
TD0 1
SFO1 100.6242389 MHz
NUC1 13C
P1 10.00 usec
P2 20.00 usec
PLW1 47.00000000 W
SFO2 400.1316005 MHz
NUC2 1H
CPDPRG[2 waltz16
P3 15.00 usec
P4 30.00 usec
PCPD2 90.00 usec
PLW2 9.69999981 W
PLW12 0.26944000 W

F2 - Processing parameters
SI 32768
SF 100.6127690 MHz
WDW EM
SSB 0
LB 1.00 Hz
GB 0
PC 1.40

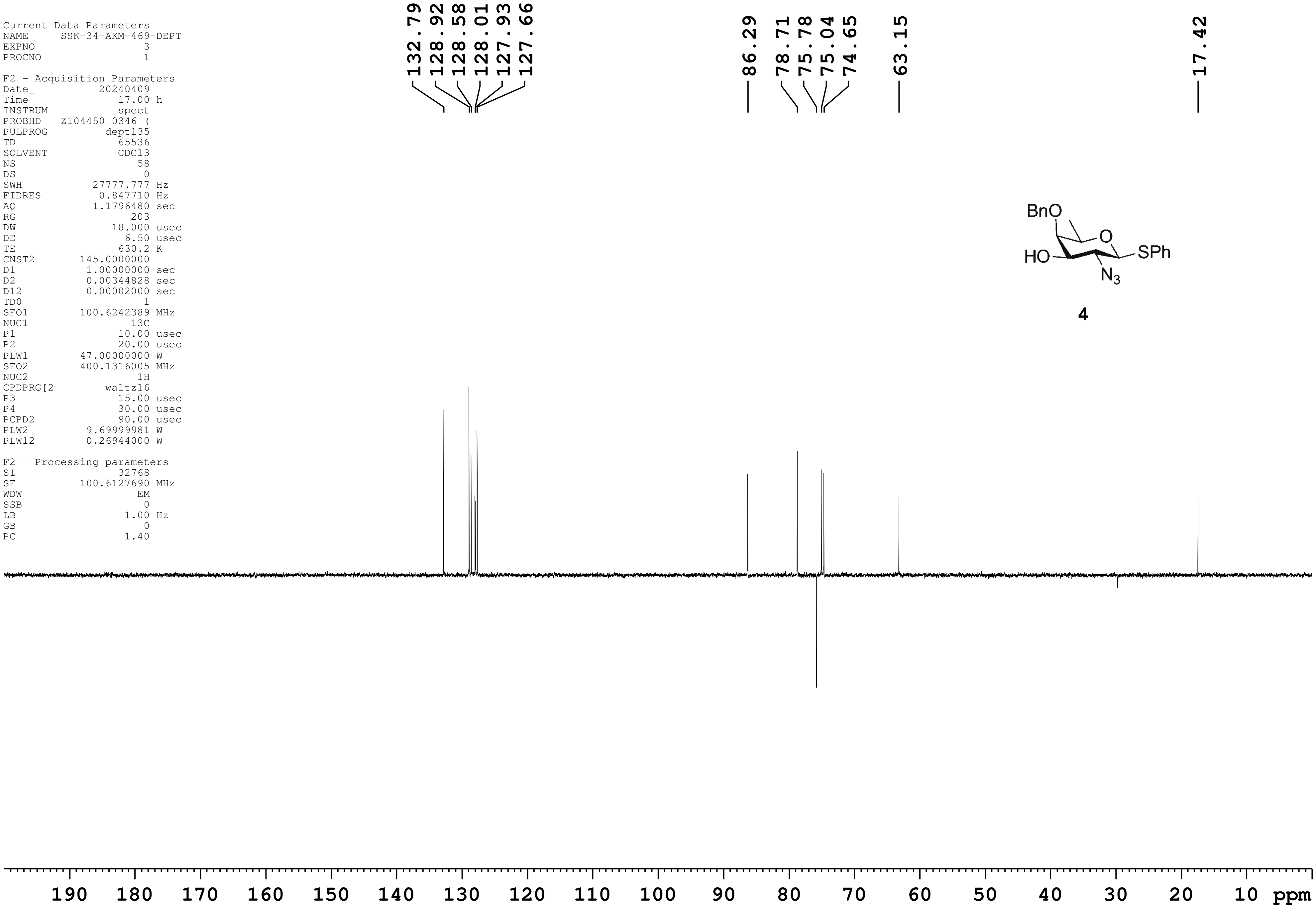

4

SSK-34-AKM-469-COSY

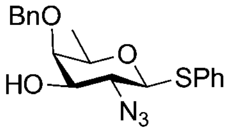

4

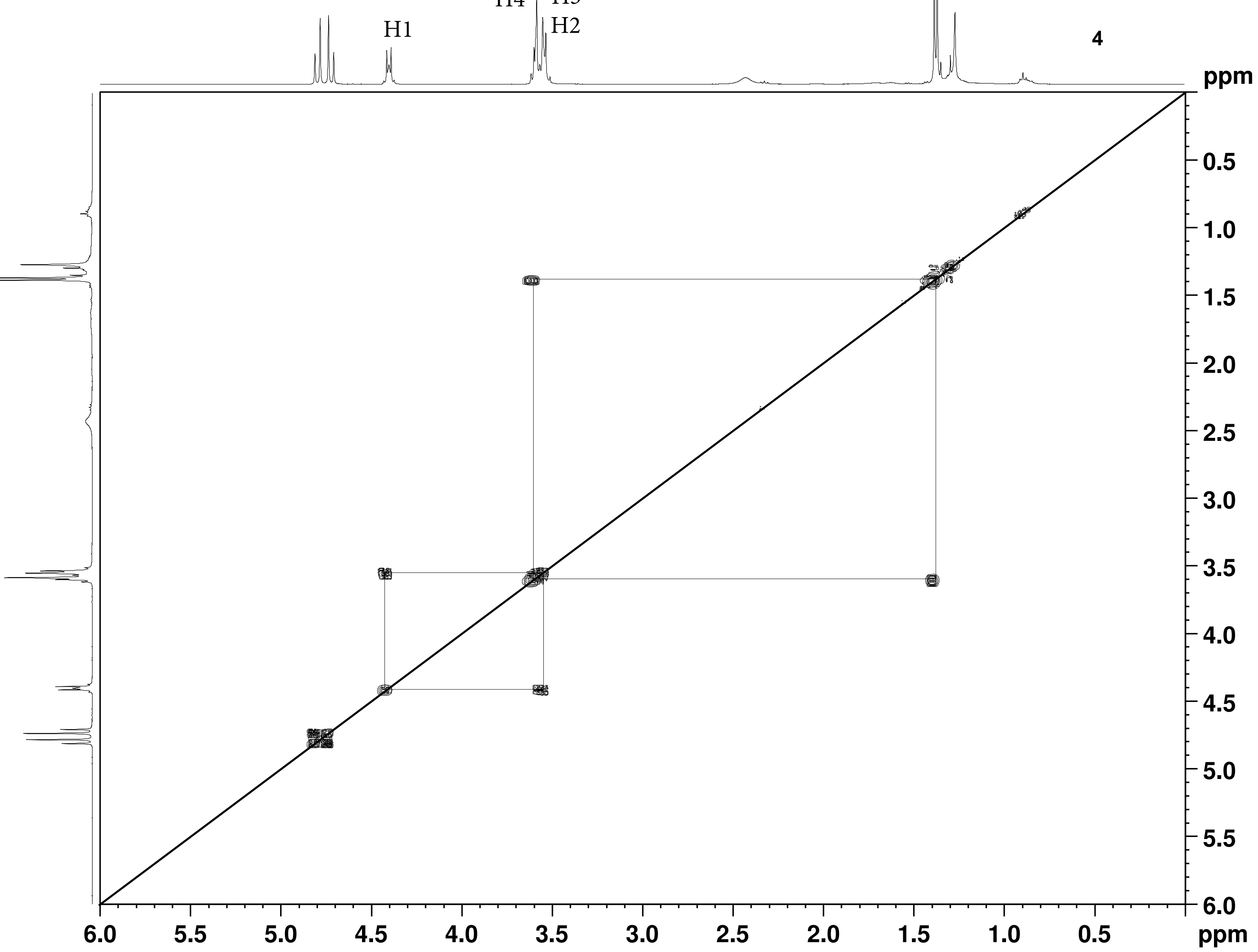


```
Current Data Parameters
NAME     SSK-34-AKM-469-COSY
EXPNO                  4
PROCNO                 1

F2 - Acquisition Parameters
Date_           20240409
Time               17.04 h
INSTRUM            spect
PROBHD   Z104450_0346 (
PULPROG         cosygpqf
TD                  2048
SOLVENT            CDCl3
NS                     2
DS                     0
SWH             2406.932 Hz
FIDRES          2.350519 Hz
AQ             0.4254379 sec
RG                    64
DW               207.733 usec
DE                  6.50 usec
TE                1903.9 K
D0            0.00000300 sec
D1            1.00000000 sec
D13           0.00000400 sec
D16           0.00020000 sec
IN0           0.00041540 sec
TDav                   1
SFO1         400.1311998 MHz
NUC1                  1H
P0                 15.00 usec
P1                 15.00 usec
PLW1          9.69999981 W
GPNAM[1]        SINE.100
GPZ1               10.00 %
P16              1000.00 usec

F1 - Acquisition parameters
TD                    80
SFO1            400.1312 MHz
FIDRES         60.182957 Hz
SW                 6.016 ppm
FnMODE                QF

F2 - Processing parameters
SI                  1024
SF           400.1300000 MHz
WDW                 SINE
SSB                    0
LB                     0 Hz
GB                     0
PC                  1.40

F1 - Processing parameters
SI                  1024
MC2                   QF
SF           400.1300000 MHz
WDW                 SINE
SSB                    0
LB                     0 Hz
GB                     0
```

SSK-34-AKM-469-HSQC

```
Current Data Parameters
NAME      SSK-34-AKM-469-HSQC
EXPNO                   5
PROCNO                  1

F2 - Acquisition Parameters
Date_           20240409
Time               17.09 h
INSTRUM            spect
PROBHD   Z104450_0346 (
PULPROG         hsqcetgp
TD                  2048
SOLVENT            CDCl3
NS                     2
DS                     0
SWH             4032.258 Hz
FIDRES          3.937752 Hz
AQ             0.2539520 sec
RG                  2050
DW               124.000 usec
DE                  6.50 usec
TE                3129.1 K
CNST2        145.0000000
D0            0.00000300 sec
D1            1.00000000 sec
D4            0.00172414 sec
D11           0.03000000 sec
D16           0.00020000 sec
IN0           0.00002480 sec
TDav                   1
ZGOPTNS
SFO1         400.1320038 MHz
NUC1                  1H
P1                 15.00 usec
P2                 30.00 usec
PLW1          9.69999981 W
SFO2         100.6228298 MHz
NUC2                 13C
CPDPRG[2            garp
P3                 10.00 usec
P4                 20.00 usec
PCPD2              80.00 usec
PLW2         47.00000000 W
PLW12         0.73438001 W
GPNAM[1]        SINE.100
GPZ1               80.00 %
GPNAM[2]        SINE.100
GPZ2               20.10 %
P16              1000.00 usec

F1 - Acquisition parameters
TD                   116
SFO1            100.6228 MHz
FIDRES        347.608459 Hz
SW               200.365 ppm
FnMODE      Echo-Antiecho

F2 - Processing parameters
SI                  2048
SF           400.1300000 MHz
WDW                QSINE
SSB                    2
LB                     0 Hz
GB                     0
PC                  1.40

F1 - Processing parameters
SI                  1024
MC2         echo-antiecho
SF           100.6127690 MHz
WDW                QSINE
SSB                    2
LB                     0 Hz
GB                     0
```

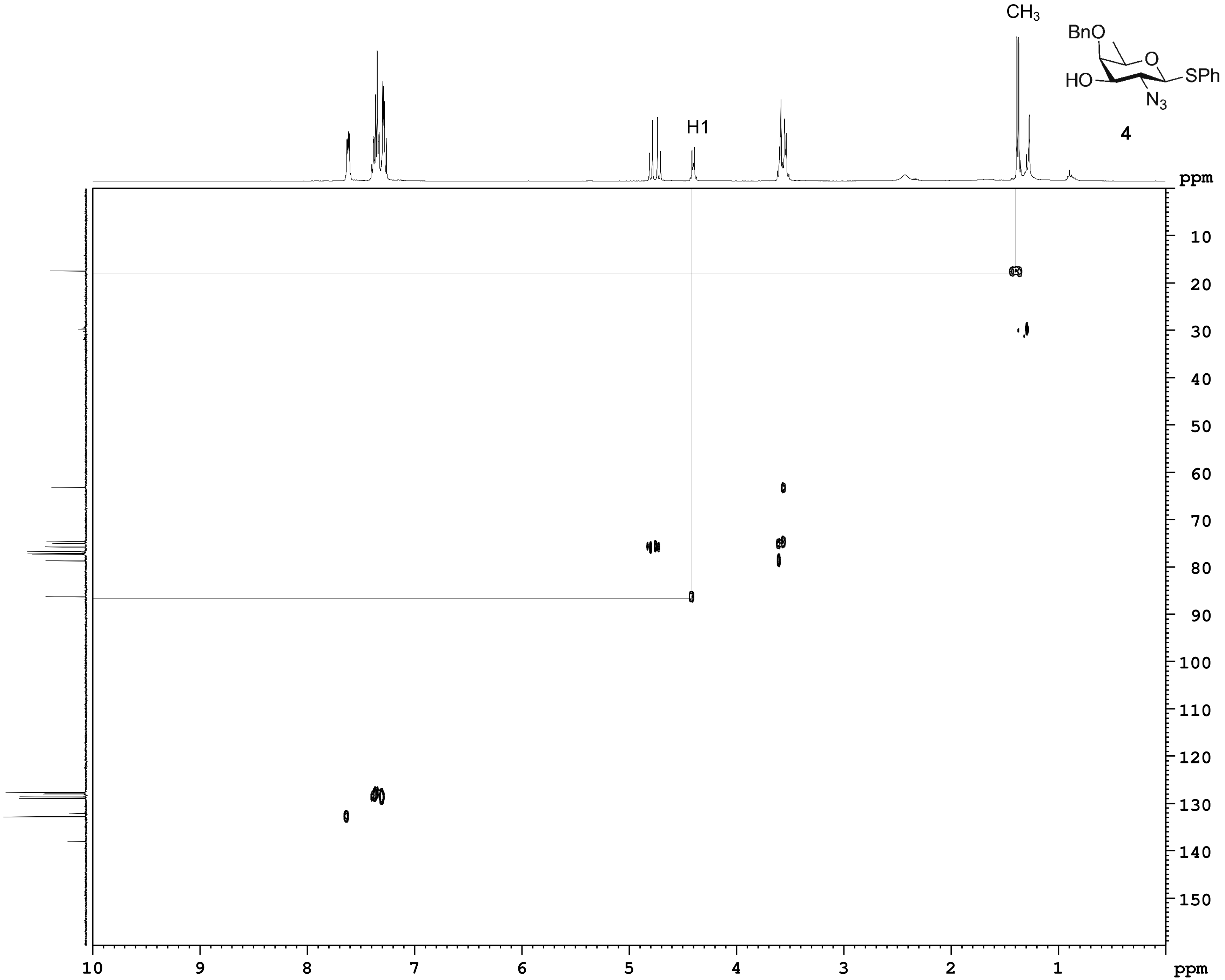

SSK-34-AKM-471-1H

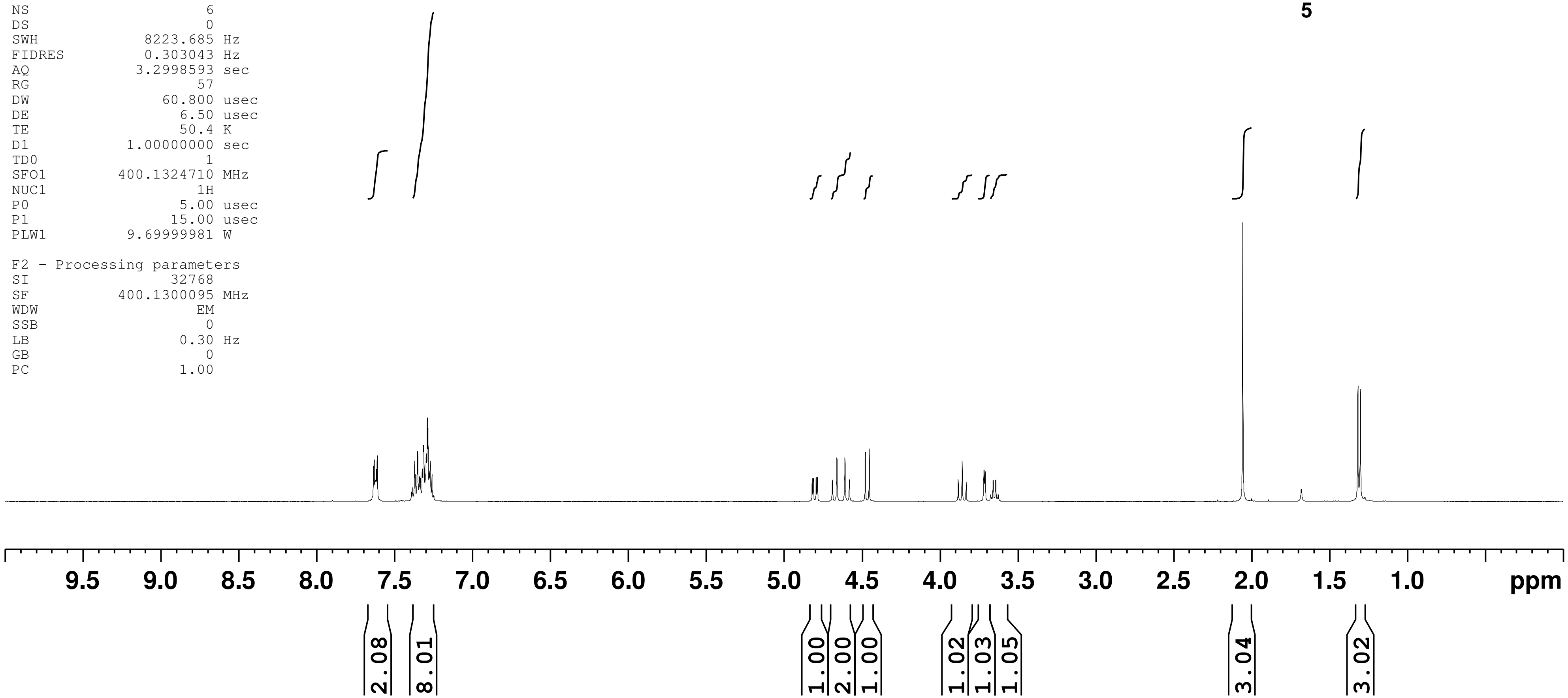

Current Data Parameters
NAME SSK-34-AKM-471-1H
EXPNO 1
PROCNO 1

F2 - Acquisition Parameters
Date_ 20240410
Time 19.21 h
INSTRUM spect
PROBHD Z104450_0346 (
PULPROG zg30
TD 54274
SOLVENT CDCl3
NS 6
DS 0
SWH 8223.685 Hz
FIDRES 0.303043 Hz
AQ 3.2998593 sec
RG 57
DW 60.800 usec
DE 6.50 usec
TE 50.4 K
D1 1.00000000 sec
TD0 1
SFO1 400.1324710 MHz
NUC1 1H
P0 5.00 usec
P1 15.00 usec
PLW1 9.69999981 W

F2 - Processing parameters
SI 32768
SF 400.1300095 MHz
WDW EM
SSB 0
LB 0.30 Hz
GB 0
PC 1.00

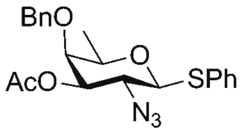

5

SSK-34-AKM-471-13C

Current Data Parameters
NAME SSK-34-AKM-471-13C
EXPNO 2
PROCNO 1

F2 - Acquisition Parameters
Date_ 20240410
Time 19.25 h
INSTRUM spect
PROBHD Z104450_0346 (
PULPROG zgpg30
TD 65536
SOLVENT CDCl3
NS 79
DS 0
SWH 26041.666 Hz
FIDRES 0.794729 Hz
AQ 1.2582912 sec
RG 1030
DW 19.200 usec
DE 6.50 usec
TE 217.4 K
D1 1.00000000 sec
D11 0.03000000 sec
TD0 1
SFO1 100.6238364 MHz
NUC1 13C
P0 3.33 usec
P1 10.00 usec
PLW1 47.00000000 W
SFO2 400.1316005 MHz
NUC2 1H
CPDPRG[2 waltz16
PCPD2 90.00 usec
PLW2 9.69999981 W
PLW12 0.26944000 W
PLW13 0.13552999 W

F2 - Processing parameters
SI 32768
SF 100.6127690 MHz
WDW EM
SSB 0
LB 1.00 Hz
GB 0
PC 1.40

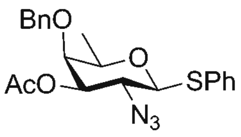

5

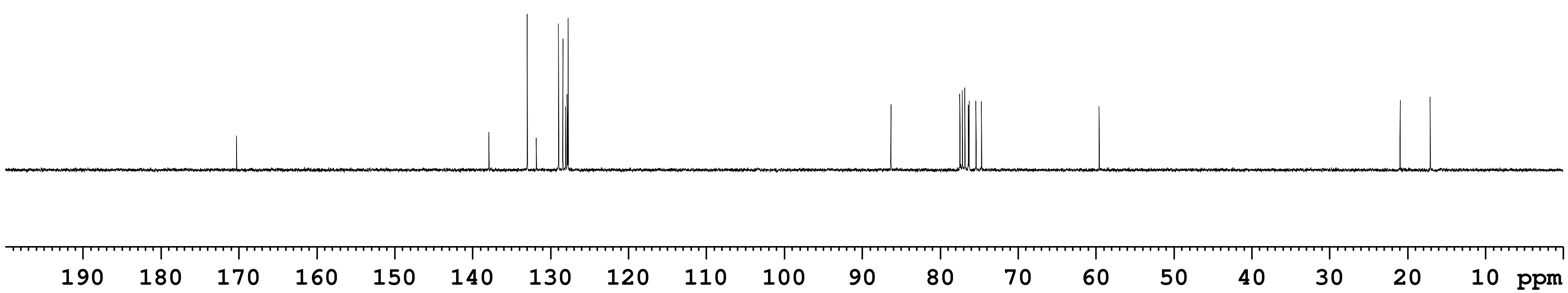

SSK-34-AKM-471-DEPT

```
Current Data Parameters
NAME      SSK-34-AKM-471-DEPT
EXPNO                   3
PROCNO                  1

F2 - Acquisition Parameters
Date_          20240410
Time              19.27 h
INSTRUM           spect
PROBHD   Z104450_0346 (
PULPROG         dept135
TD                65536
SOLVENT           CDCl3
NS                   54
DS                    0
SWH           27777.777 Hz
FIDRES         0.847710 Hz
AQ            1.1796480 sec
RG                  203
DW               18.000 usec
DE                 6.50 usec
TE                 85.9 K
CNST2       145.0000000
D1           1.00000000 sec
D2           0.00344828 sec
D12          0.00002000 sec
TD0                   1
SFO1        100.6242389 MHz
NUC1                13C
P1                10.00 usec
P2                20.00 usec
PLW1        47.00000000 W
SFO2        400.1316005 MHz
NUC2                 1H
CPDPRG[2        waltz16
P3                15.00 usec
P4                30.00 usec
PCPD2             90.00 usec
PLW2         9.69999981 W
PLW12        0.26944000 W

F2 - Processing parameters
SI                32768
SF          100.6127690 MHz
WDW                  EM
SSB                   0
LB                 1.00 Hz
GB                    0
PC                 1.40
```

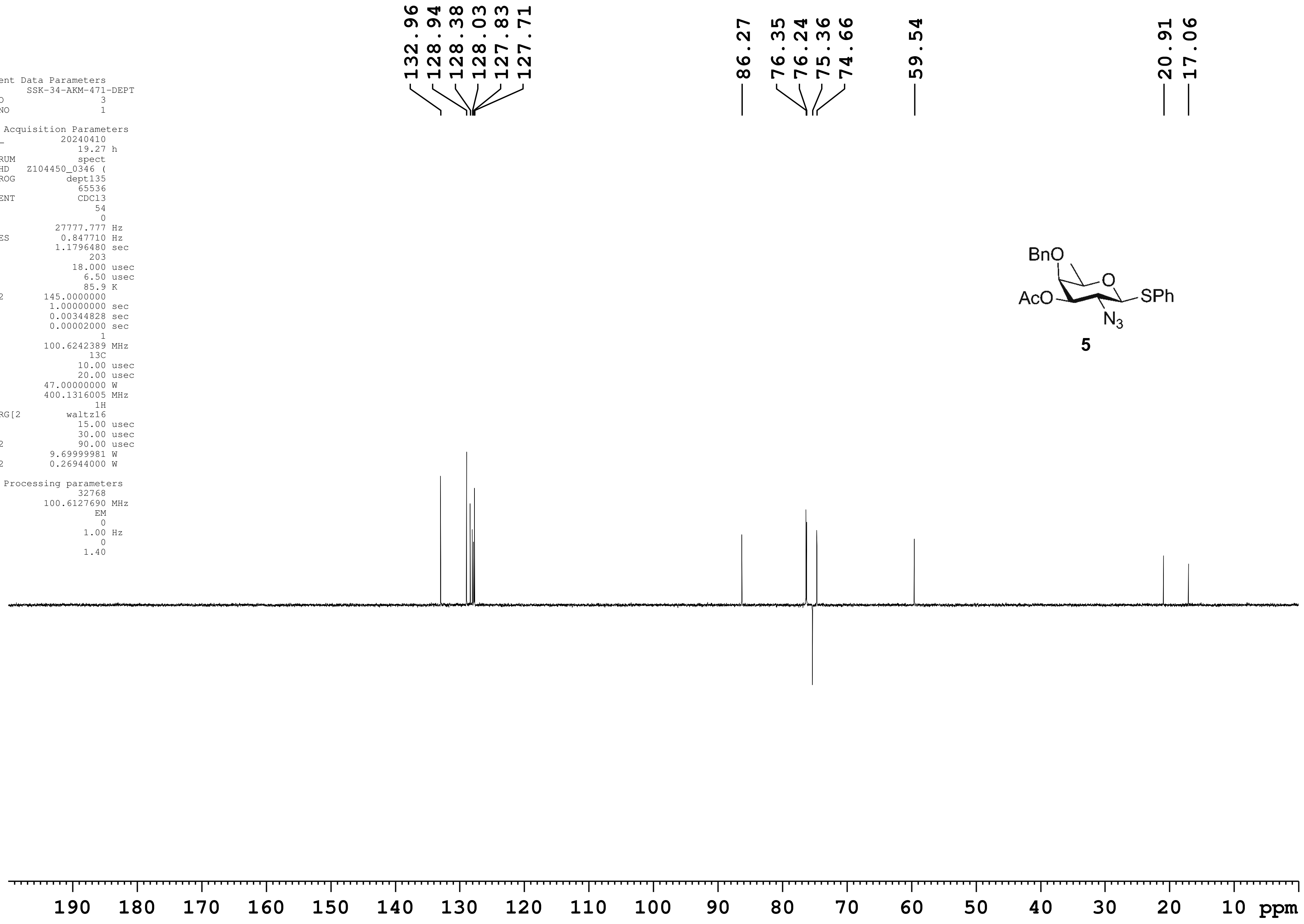

5

SSK-34-AKM-471-COSY

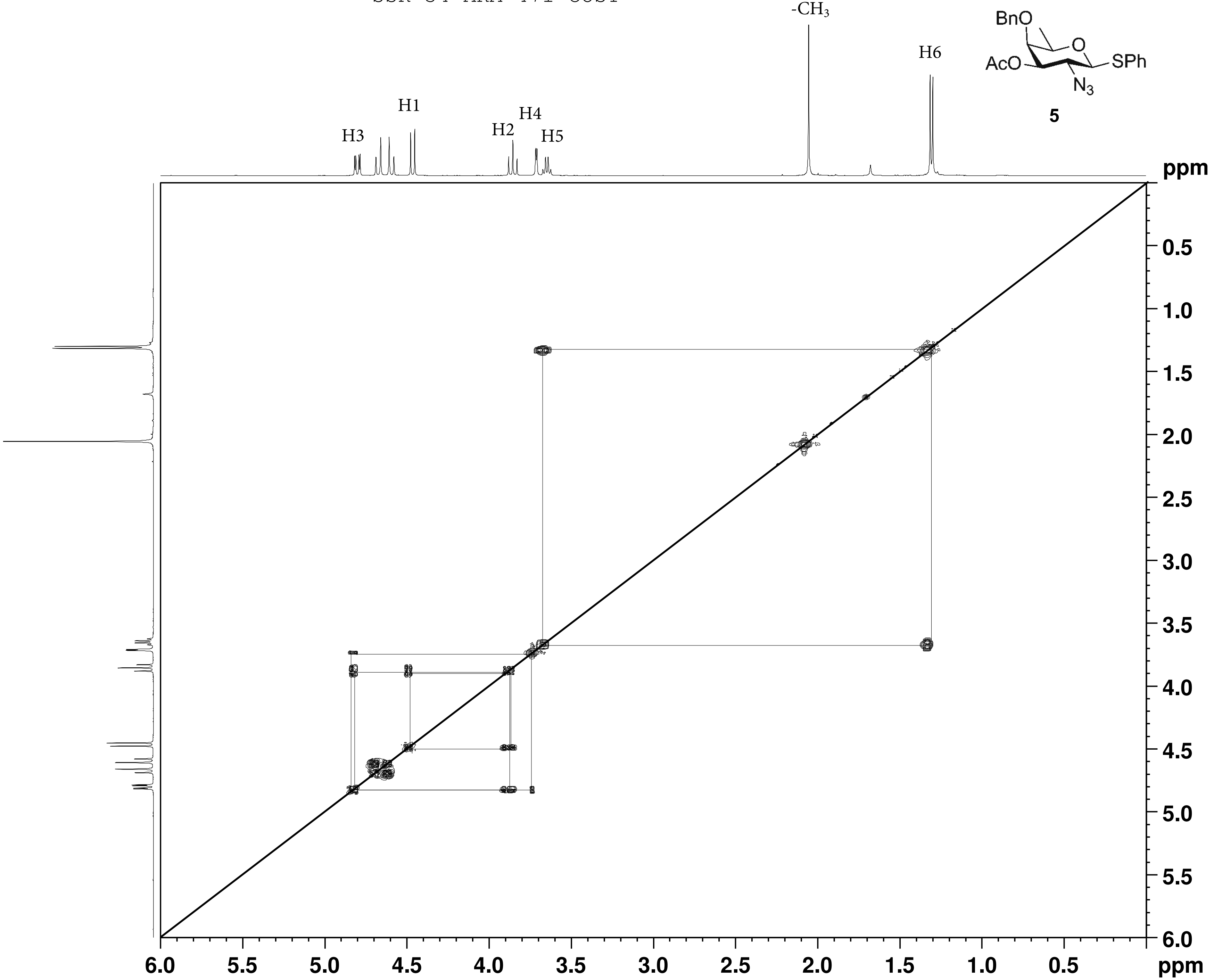

5

```
Current Data Parameters
NAME      SSK-34-AKM-471-COSY
EXPNO                   4
PROCNO                  1

F2 - Acquisition Parameters
Date_            20240410
Time                19.33 h
INSTRUM             spect
PROBHD   Z104450_0346 (
PULPROG          cosygpqf
TD                   2048
SOLVENT             CDC13
NS                      2
DS                      0
SWH              2417.795 Hz
FIDRES           2.361128 Hz
AQ              0.4235264 sec
RG                     64
DW                206.800 usec
DE                   6.50 usec
TE                   82.1 K
D0             0.00000300 sec
D1             1.00000000 sec
D13            0.00000400 sec
D16            0.00020000 sec
IN0            0.00041360 sec
TDav                    1
SFO1          400.1311987 MHz
NUC1                   1H
P0                  15.00 usec
P1                  15.00 usec
PLW1           9.69999981 W
GPNAM[1]         SINE.100
GPZ1                10.00 %
P16               1000.00 usec

F1 - Acquisition parameters
TD                    128
SFO1             400.1312 MHz
FIDRES          37.778046 Hz
SW                  6.043 ppm
FnMODE                 QF

F2 - Processing parameters
SI                   1024
SF            400.1300000 MHz
WDW                  SINE
SSB                     0
LB                      0 Hz
GB                      0
PC                   1.40

F1 - Processing parameters
SI                   1024
MC2                    QF
SF            400.1300000 MHz
WDW                  SINE
SSB                     0
LB                      0 Hz
GB                      0
```

SSK-34-AKM-471-HSQC

```
Current Data Parameters
NAME     SSK-34-AKM-471-HSQC
EXPNO                      5
PROCNO                     1

F2 - Acquisition Parameters
Date_               20240410
Time                   19.40 h
INSTRUM                spect
PROBHD    Z104450_0346 (
PULPROG             hsqcetgp
TD                      2048
SOLVENT                CDCl3
NS                         2
DS                         0
SWH                 4025.765 Hz
FIDRES              3.931411 Hz
AQ                 0.2543616 sec
RG                      2050
DW                   124.200 usec
DE                      6.50 usec
TE                   -2391.5 K
CNST2            145.0000000
D0                0.00000300 sec
D1                1.00000000 sec
D4                0.00172414 sec
D11               0.03000000 sec
D16               0.00020000 sec
IN0               0.00002480 sec
TDav                       1
ZGOPTNS
SFO1             400.1320044 MHz
NUC1                      1H
P1                     15.00 usec
P2                     30.00 usec
PLW1              9.69999981 W
SFO2             100.6228095 MHz
NUC2                     13C
CPDPRG[2                garp
P3                     10.00 usec
P4                     20.00 usec
PCPD2                  80.00 usec
PLW2             47.00000000 W
PLW12             0.73438001 W
GPNAM[1]            SINE.100
GPZ1                   80.00 %
GPNAM[2]            SINE.100
GPZ2                   20.10 %
P16                  1000.00 usec

F1 - Acquisition parameters
TD                       146
SFO1                100.6228 MHz
FIDRES            276.182068 Hz
SW                   200.365 ppm
FnMODE         Echo-Antiecho

F2 - Processing parameters
SI                      2048
SF               400.1300000 MHz
WDW                    QSINE
SSB                        2
LB                         0 Hz
GB                         0
PC                      1.40

F1 - Processing parameters
SI                      1024
MC2            echo-antiecho
SF               100.6127690 MHz
WDW                    QSINE
SSB                        2
LB                         0 Hz
GB                         0
```

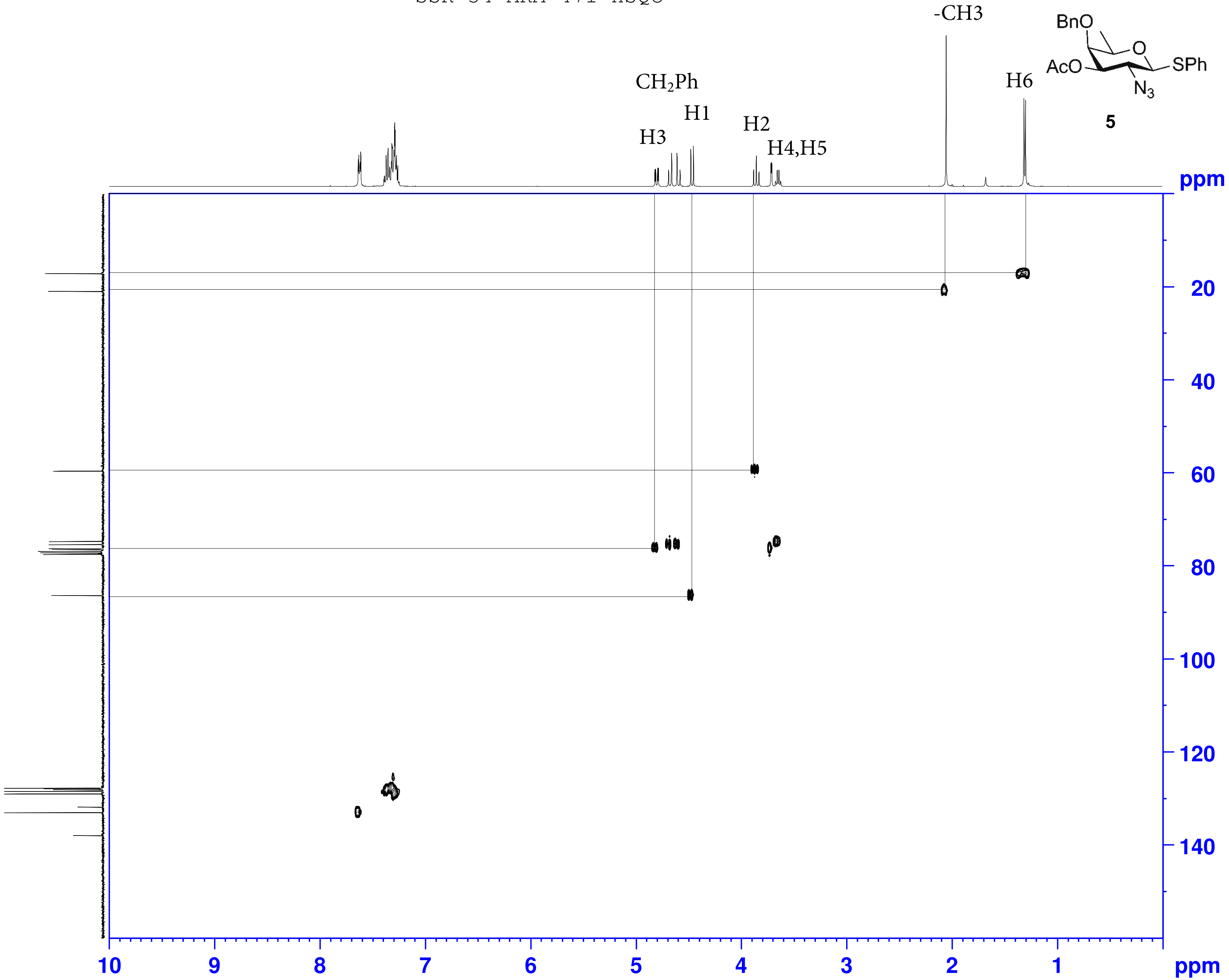

SSK-34-AKM-486-1H

Current Data Parameters
NAME SSK-34-AKM-486-1H
EXPNO 1
PROCNO 1

F2 - Acquisition Parameters
Date_ 20240509
Time 11.57 h
INSTRUM spect
PROBHD Z104450_0346 (
PULPROG zg30
TD 54274
SOLVENT CDCl3
NS 25
DS 0
SWH 8223.685 Hz
FIDRES 0.303043 Hz
AQ 3.2998593 sec
RG 161
DW 60.800 usec
DE 6.50 usec
TE 166.3 K
D1 1.00000000 sec
TD0 1
SFO1 400.1324710 MHz
NUC1 1H
P0 5.00 usec
P1 15.00 usec
PLW1 9.69999981 W

F2 - Processing parameters
SI 32768
SF 400.1300095 MHz
WDW EM
SSB 0
LB 0.30 Hz
GB 0
PC 1.00

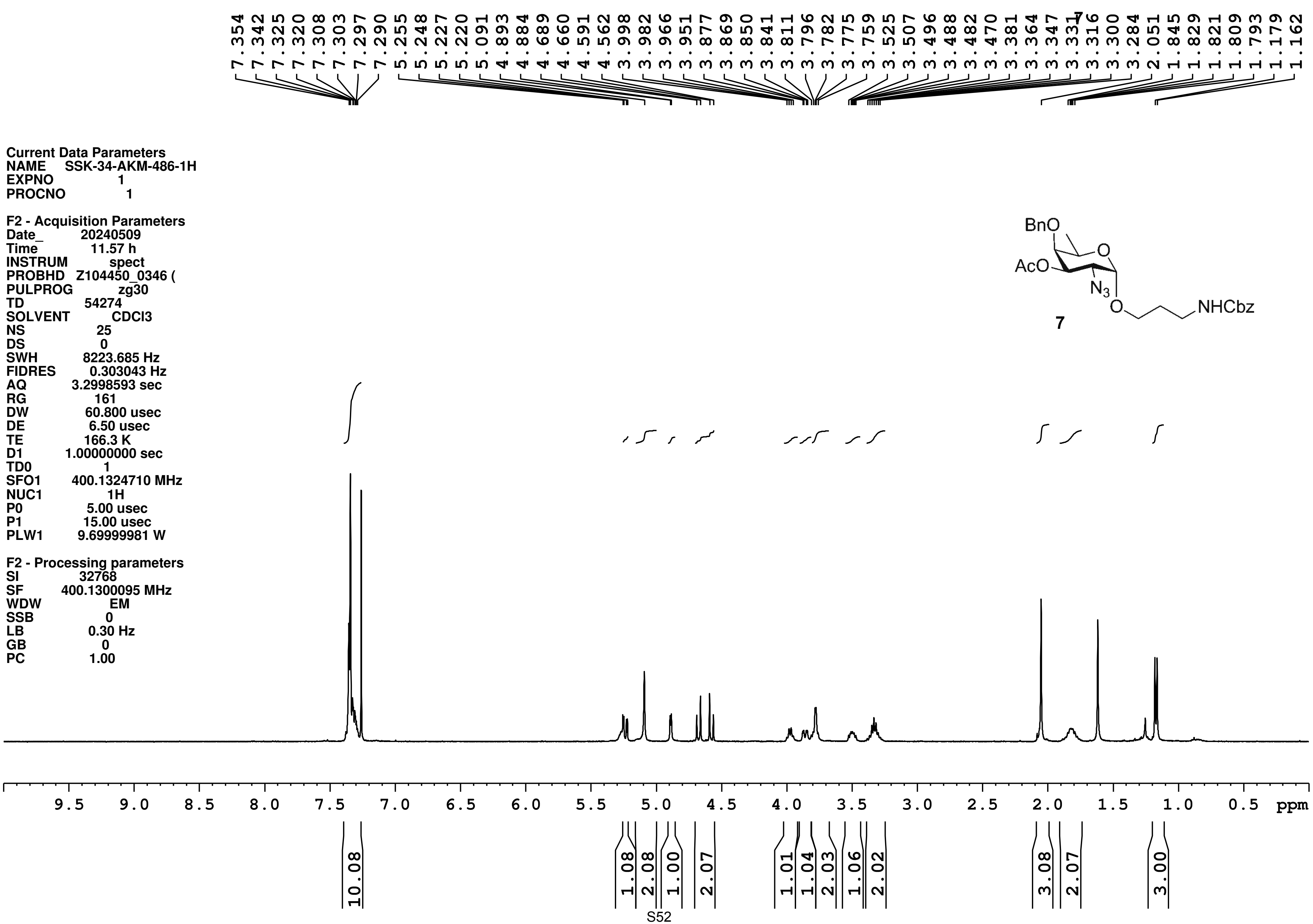

SSK-34-AKM-FUC-LIN-13C

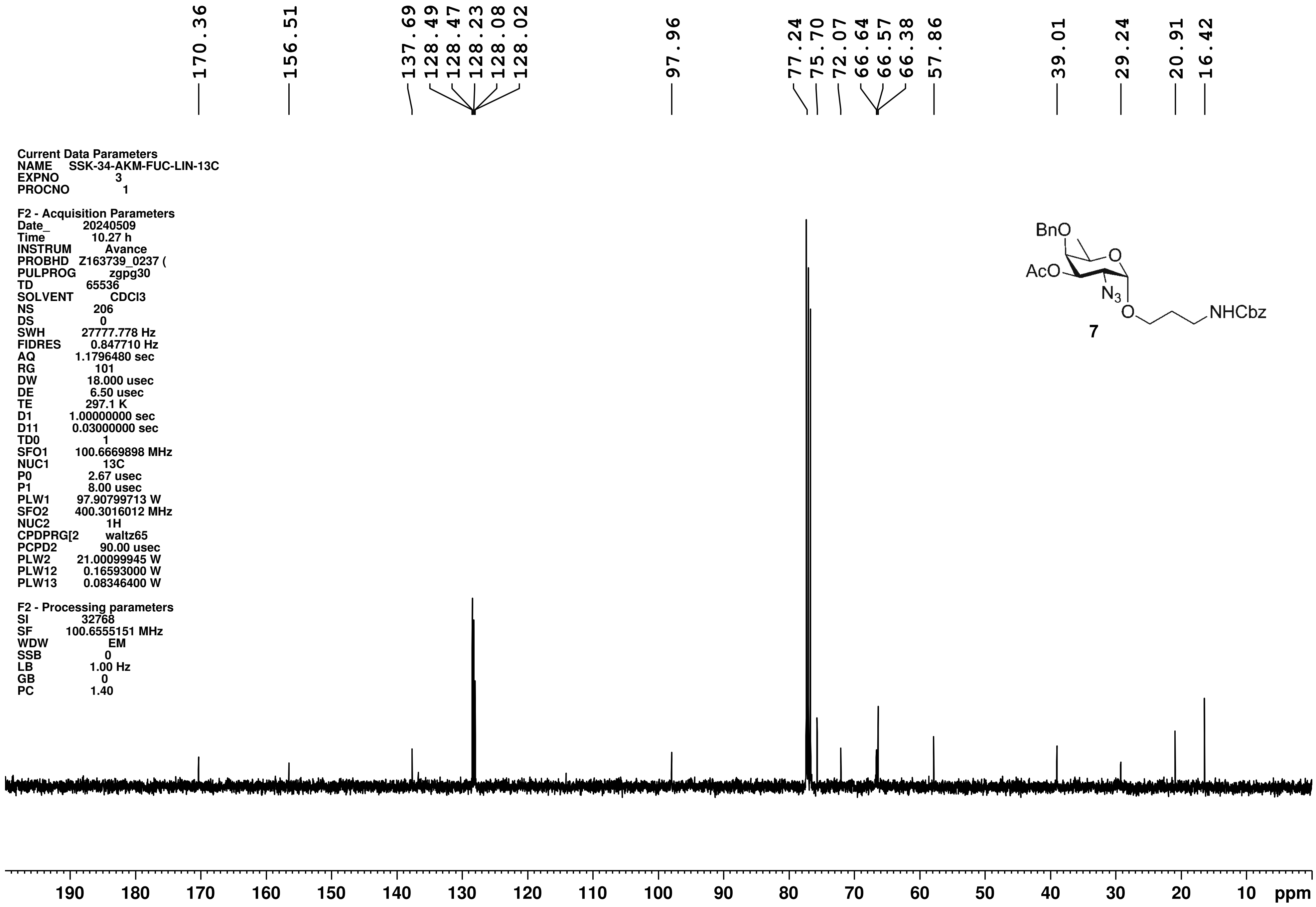

Current Data Parameters
NAME SSK-34-AKM-FUC-LIN-13C
EXPNO 3
PROCNO 1

F2 - Acquisition Parameters
Date_ 20240509
Time 10.27 h
INSTRUM Avance
PROBHD Z163739_0237 (
PULPROG zgpg30
TD 65536
SOLVENT CDCl3
NS 206
DS 0
SWH 27777.778 Hz
FIDRES 0.847710 Hz
AQ 1.1796480 sec
RG 101
DW 18.000 usec
DE 6.50 usec
TE 297.1 K
D1 1.00000000 sec
D11 0.03000000 sec
TD0 1
SFO1 100.6669898 MHz
NUC1 13C
P0 2.67 usec
P1 8.00 usec
PLW1 97.90799713 W
SFO2 400.3016012 MHz
NUC2 1H
CPDPRG[2 waltz65
PCPD2 90.00 usec
PLW2 21.00099945 W
PLW12 0.16593000 W
PLW13 0.08346400 W

F2 - Processing parameters
SI 32768
SF 100.6555151 MHz
WDW EM
SSB 0
LB 1.00 Hz
GB 0
PC 1.40

7

SSK-34-AKM-486-DEPT

Current Data Parameters
NAME SSK-34-AKM-486-DEPT
EXPNO 2
PROCNO 1

F2 - Acquisition Parameters
Date_ 20240509
Time 12.07 h
INSTRUM spect
PROBHD Z104450_0346 (
PULPROG dept135
TD 65536
SOLVENT CDCl3
NS 250
DS 0
SWH 27777.777 Hz
FIDRES 0.847710 Hz
AQ 1.1796480 sec
RG 203
DW 18.000 usec
DE 6.50 usec
TE 166.3 K
CNST2 145.0000000
D1 1.00000000 sec
D2 0.00344828 sec
D12 0.00002000 sec
TD0 1
SFO1 100.6242389 MHz
NUC1 13C
P1 10.00 usec
P2 20.00 usec
PLW1 47.00000000 W
SFO2 400.1316005 MHz
NUC2 1H
CPDPRG[2 waltz16
P3 15.00 usec
P4 30.00 usec
PCPD2 90.00 usec
PLW2 9.69999981 W
PLW12 0.26944000 W

F2 - Processing parameters
SI 32768
SF 100.6127690 MHz
WDW EM
SSB 0
LB 1.00 Hz
GB 0
PC 1.40

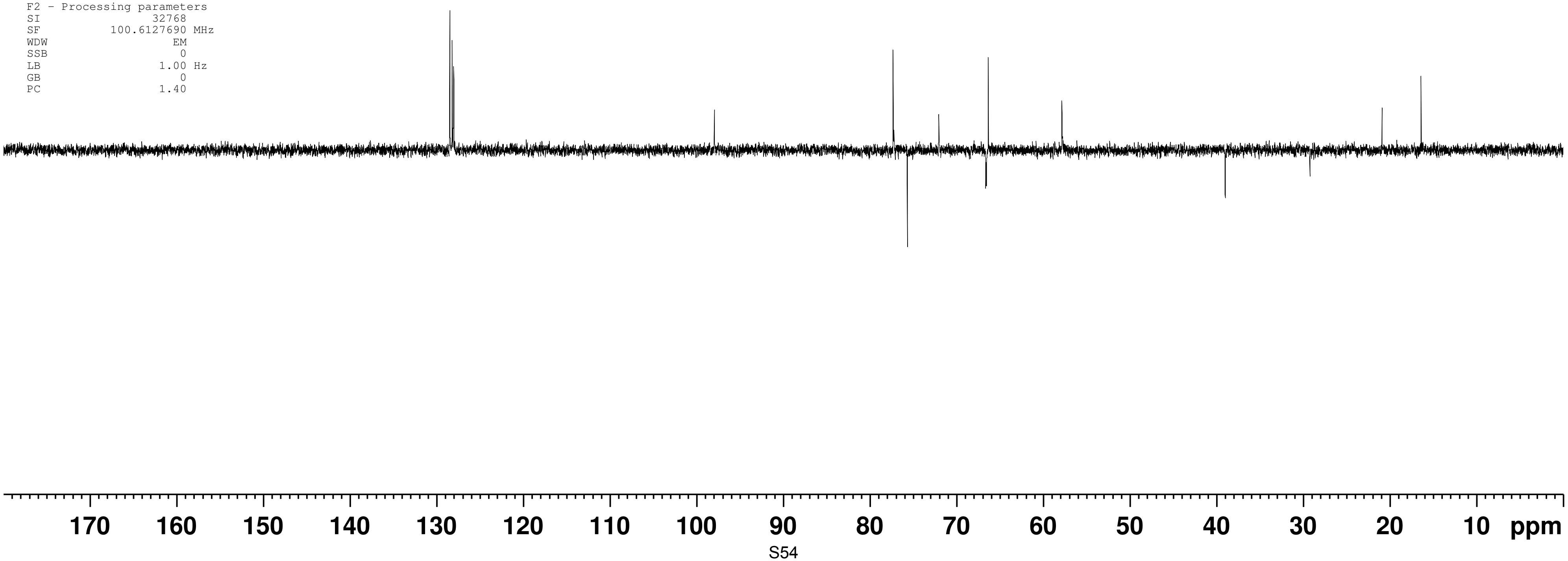

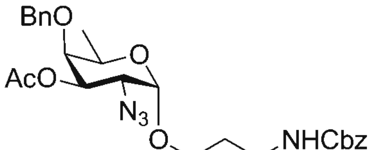

7

SSK-34-AKM-486-COSY

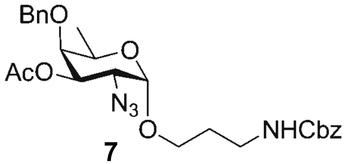

7

Current Data Parameters
NAME SSK-34-AKM-486-COSY
EXPNO 3
PROCNO 1

F2 - Acquisition Parameters
Date_ 20240509
Time 12.13 h
INSTRUM spect
PROBHD Z104450_0346 (
PULPROG cosygpqf
TD 2048
SOLVENT CDCl3
NS 2
DS 0
SWH 2413.127 Hz
FIDRES 2.356570 Hz
AQ 0.4243456 sec
RG 64
DW 207.200 usec
DE 6.50 usec
TE 166.2 K
D0 0.00000300 sec
D1 1.00000000 sec
D13 0.00000400 sec
D16 0.00020000 sec
IN0 0.00041440 sec
TDav 1
SFO1 400.1312381 MHz
NUC1 1H
P0 15.00 usec
P1 15.00 usec
PLW1 9.69999981 W
GPNAM[1] SINE.100
GPZ1 10.00 %
P16 1000.00 usec

F1 - Acquisition parameters
TD 128
SFO1 400.1312 MHz
FIDRES 37.705116 Hz
SW 6.031 ppm
FnMODE QF

F2 - Processing parameters
SI 1024
SF 400.1300000 MHz
WDW SINE
SSB 0
LB 0 Hz
GB 0
PC 1.40

F1 - Processing parameters
SI 1024
MC2 QF
SF 400.1300000 MHz
WDW SINE
SSB 0
LB 0 Hz
GB 0

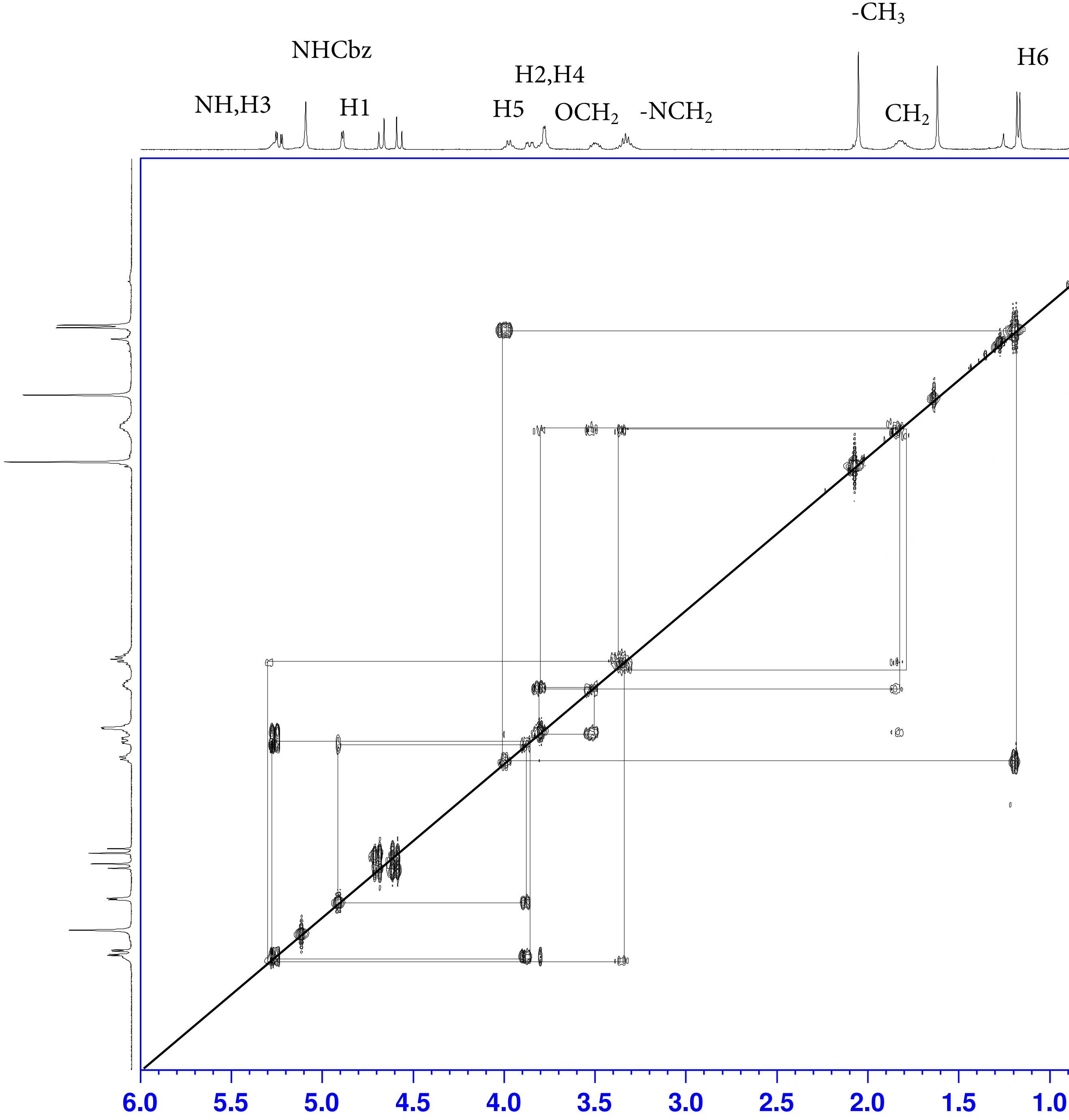

SSK-34-AKM-486-HSQC

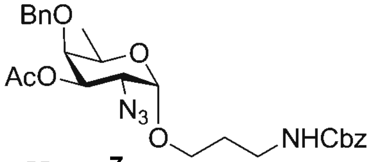

7

```
Current Data Parameters
NAME      SSK-34-AKM-486-HSQC
EXPNO                       4
PROCNO                      1

F2 - Acquisition Parameters
Date_            20240509
Time                12.21 h
INSTRUM             spect
PROBHD    Z104450_0346 (
PULPROG         hsqcetgp
TD                   2048
SOLVENT             CDCl3
NS                      2
DS                      0
SWH              4025.765 Hz
FIDRES           3.931411 Hz
AQ              0.2543616 sec
RG                   2050
DW                124.200 usec
DE                   6.50 usec
TE                  166.2 K
CNST2         145.0000000
D0             0.00000300 sec
D1             1.00000000 sec
D4             0.00172414 sec
D11            0.03000000 sec
D16            0.00020000 sec
IN0            0.00002610 sec
TDav                    1
ZGOPTNS
SFO1          400.1320040 MHz
NUC1                   1H
P1                  15.00 usec
P2                  30.00 usec
PLW1           9.69999981 W
SFO2          100.6223846 MHz
NUC2                  13C
CPDPRG[2             garp
P3                  10.00 usec
P4                  20.00 usec
PCPD2               80.00 usec
PLW2          47.00000000 W
PLW12          0.73438001 W
GPNAM[1]         SINE.100
GPZ1                80.00 %
GPNAM[2]         SINE.100
GPZ2                20.10 %
P16               1000.00 usec

F1 - Acquisition parameters
TD                    152
SFO1             100.6224 MHz
FIDRES          252.066956 Hz
SW                190.386 ppm
FnMODE      Echo-Antiecho

F2 - Processing parameters
SI                   2048
SF            400.1300000 MHz
WDW                 QSINE
SSB                     2
LB                      0 Hz
GB                      0
PC                   1.40

F1 - Processing parameters
SI                   1024
MC2         echo-antiecho
SF            100.6127690 MHz
WDW                 QSINE
SSB                     2
LB                      0 Hz
GB                      0
```

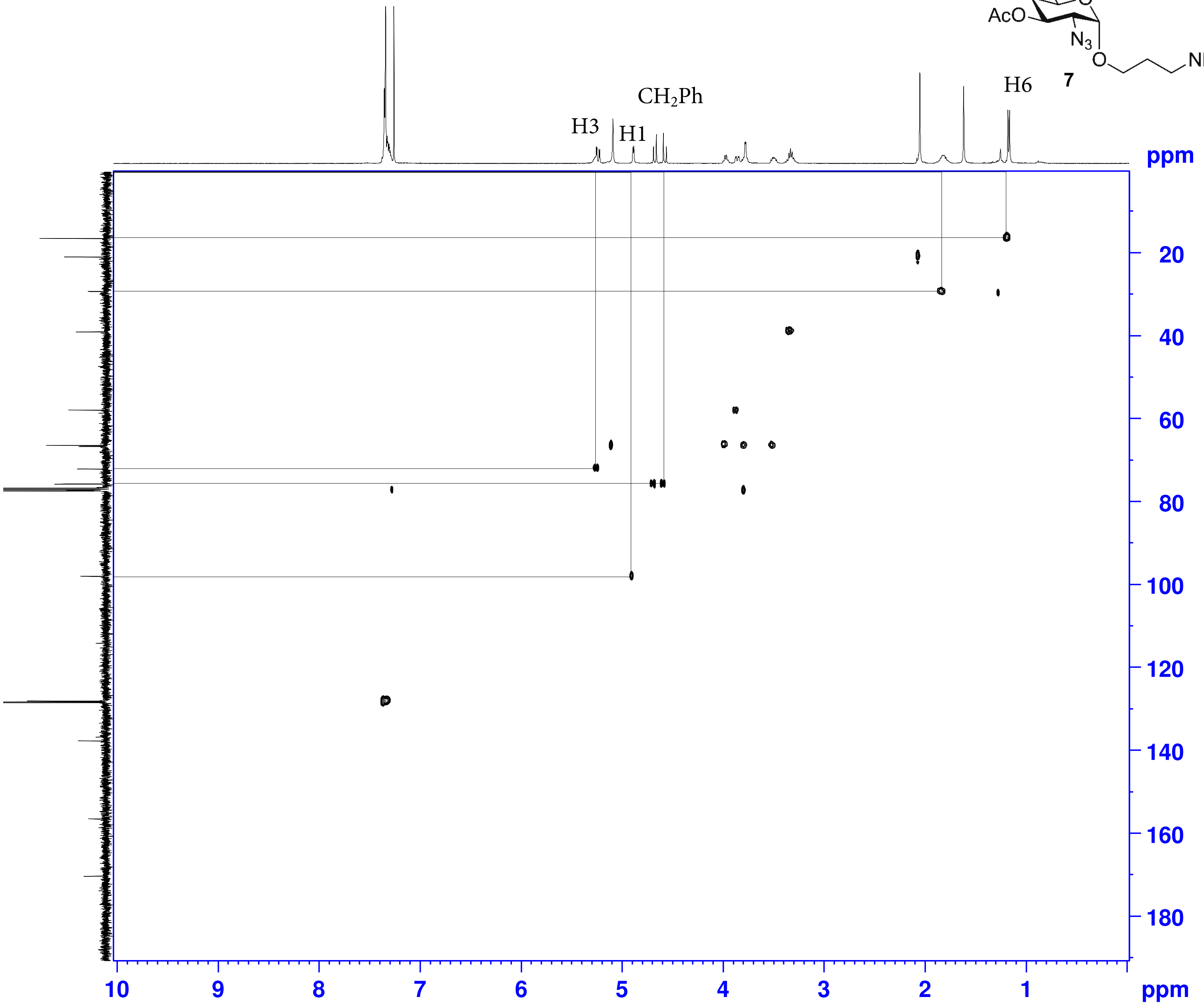

SSK-34-AKM-496-1H

```
Current Data Parameters
NAME      SSK-34-AKM-496-1H
EXPNO                    1
PROCNO                   1

F2 - Acquisition Parameters
Date_            20240531
Time                20.44 h
INSTRUM             spect
PROBHD   Z119470_0087 (
PULPROG              zg30
TD                  65536
SOLVENT             CDCl3
NS                     11
DS                      0
SWH             10000.000 Hz
FIDRES           0.305176 Hz
AQ              3.2767999 sec
RG                  30.72
DW                 50.000 usec
DE                   6.50 usec
TE                  297.3 K
D1             1.00000000 sec
TD0                     1
SFO1          500.1330885 MHz
NUC1                   1H
P0                   4.45 usec
P1                  13.35 usec
PLW1          16.00000000 W

F2 - Processing parameters
SI                  65536
SF            500.1300000 MHz
WDW                    EM
SSB                     0
LB                   0.30 Hz
GB                      0
PC                   1.00
```

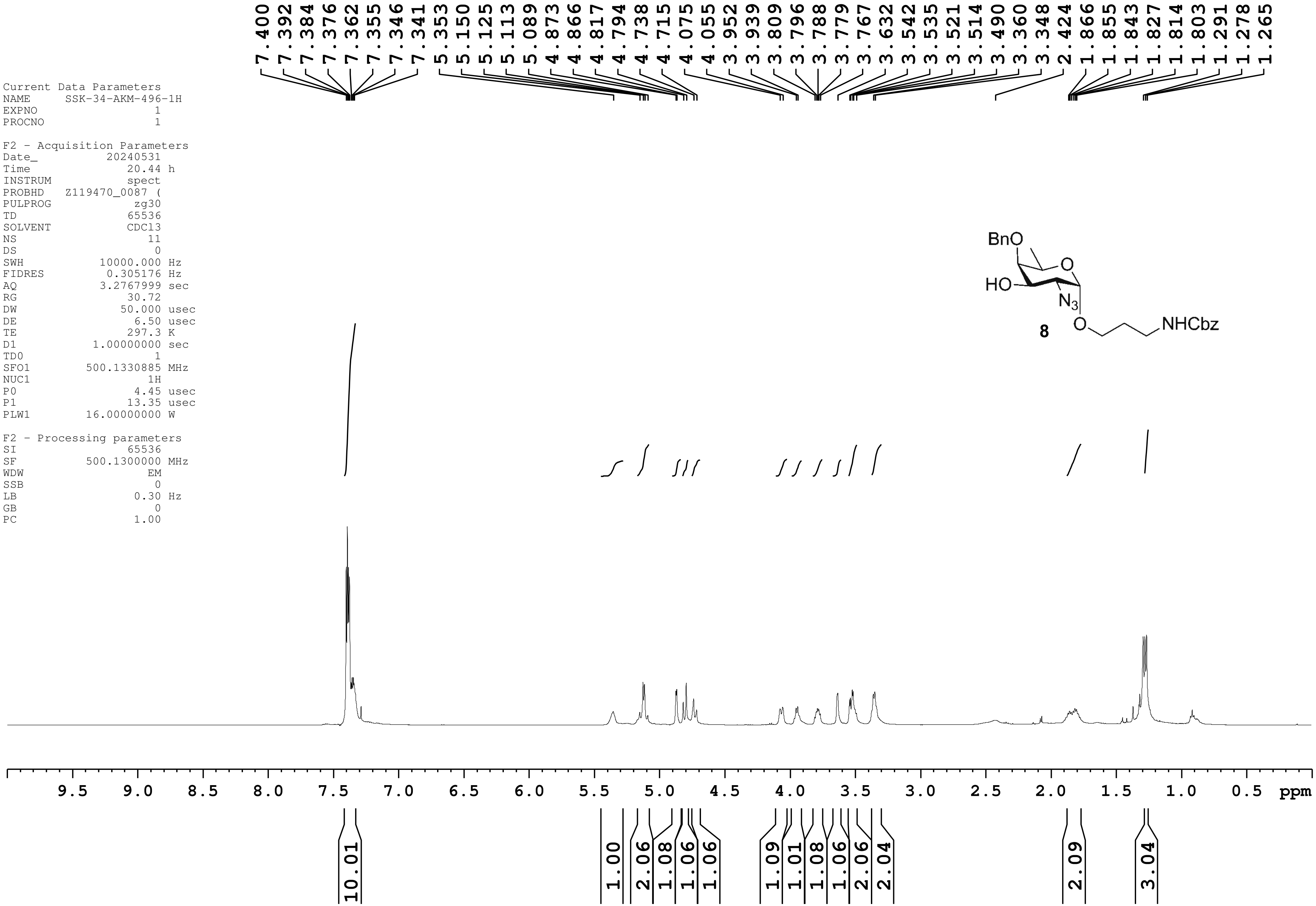

SSK-34-AKM-496-13C

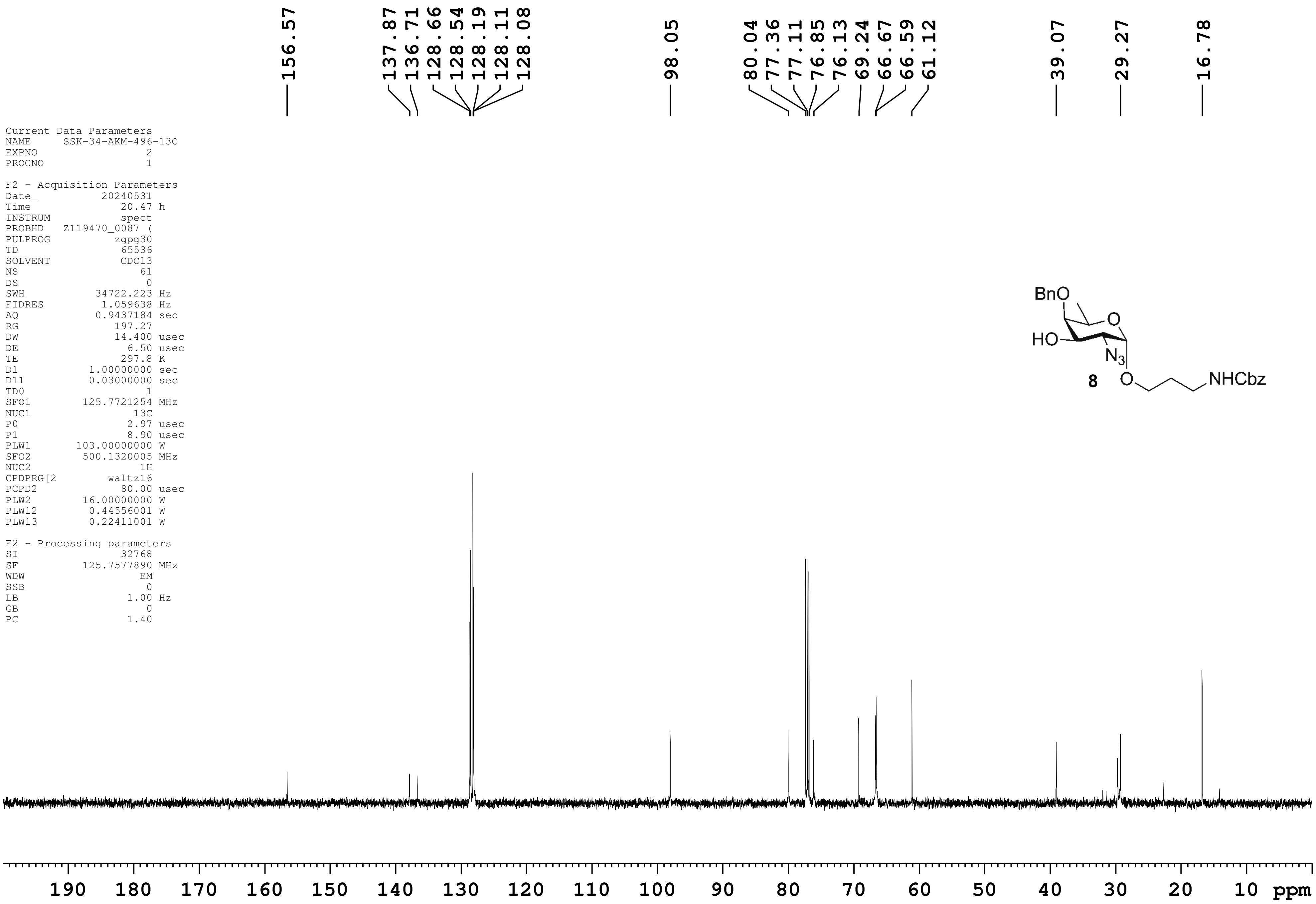

Current Data Parameters
NAME SSK-34-AKM-496-13C
EXPNO 2
PROCNO 1

F2 - Acquisition Parameters
Date_ 20240531
Time 20.47 h
INSTRUM spect
PROBHD Z119470_0087 (
PULPROG zgpg30
TD 65536
SOLVENT CDCl3
NS 61
DS 0
SWH 34722.223 Hz
FIDRES 1.059638 Hz
AQ 0.9437184 sec
RG 197.27
DW 14.400 usec
DE 6.50 usec
TE 297.8 K
D1 1.00000000 sec
D11 0.03000000 sec
TD0 1
SFO1 125.7721254 MHz
NUC1 13C
P0 2.97 usec
P1 8.90 usec
PLW1 103.00000000 W
SFO2 500.1320005 MHz
NUC2 1H
CPDPRG[2 waltz16
PCPD2 80.00 usec
PLW2 16.00000000 W
PLW12 0.44556001 W
PLW13 0.22411001 W

F2 - Processing parameters
SI 32768
SF 125.7577890 MHz
WDW EM
SSB 0
LB 1.00 Hz
GB 0
PC 1.40

8

SSK-34-AKM-496-DEPT

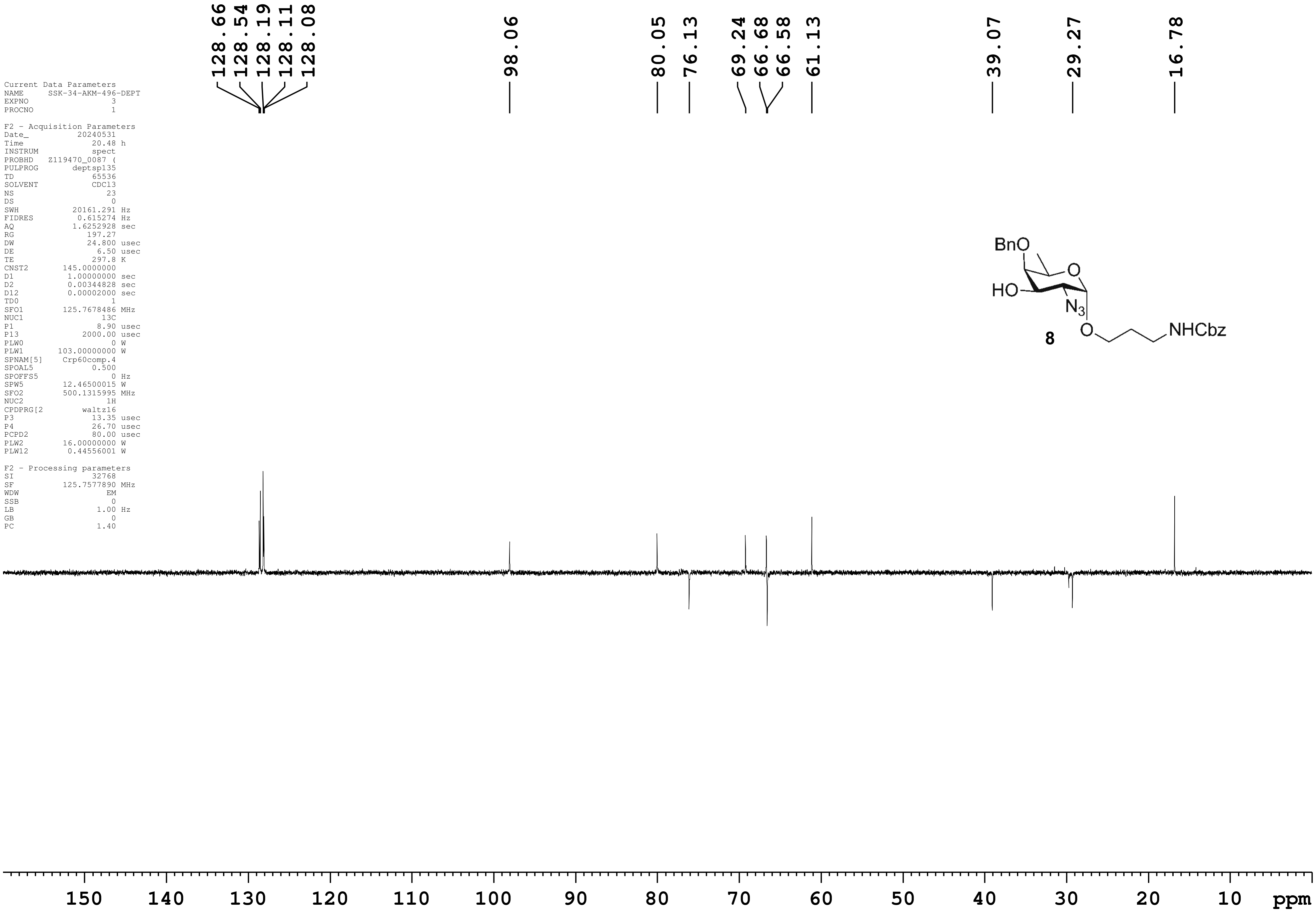

SSK-34-AKM-FINAL-MONO-1H

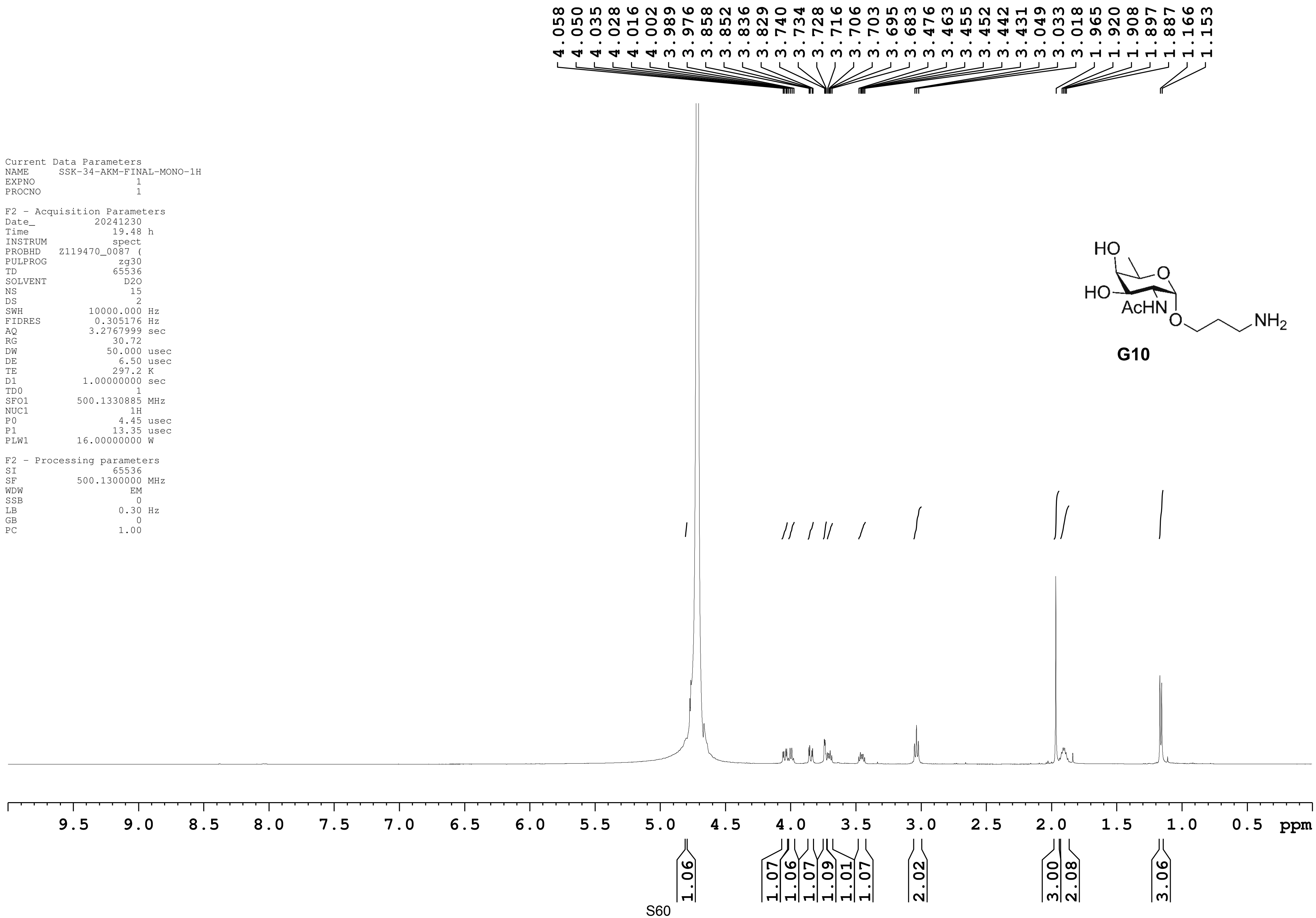

G10

SSK-34-AKM-FINAL-MONO-13C

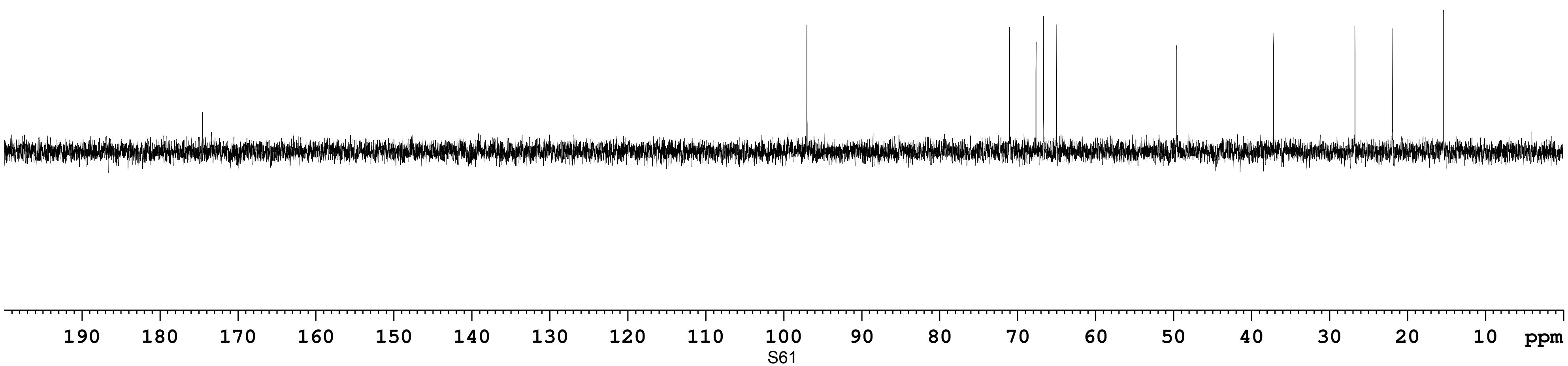

Current Data Parameters
NAME SSK-34-AKM-FINAL-MONO-13C
EXPNO 3
PROCNO 1

F2 - Acquisition Parameters
Date_ 20241231
Time 0.38 h
INSTRUM Avance Neo 400
PROBHD Z163739_0226 (
PULPROG zgpg30
TD 65536
SOLVENT D2O
NS 252
DS 0
SWH 27777.777 Hz
FIDRES 0.847710 Hz
AQ 1.1796480 sec
RG 101
DW 18.000 usec
DE 6.50 usec
TE 295.3 K
D1 1.00000000 sec
D11 0.03000000 sec
TD0 1
SFO1 100.6242384 MHz
NUC1 13C
P0 2.67 usec
P1 8.00 usec
PLW1 101.36000061 W
SFO2 400.1316005 MHz
NUC2 1H
CPDPRG[2 waltz65
PCPD2 90.00 usec
PLW2 22.88299942 W
PLW12 0.18080001 W
PLW13 0.09094300 W

F2 - Processing parameters
SI 32768
SF 100.6127685 MHz
WDW EM
SSB 0
LB 1.00 Hz
GB 0
PC 1.40

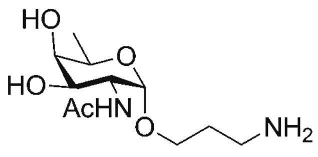

G10

SSK-34-AKM-FINAL-MONO-DEPT

```
Current Data Parameters
NAME     SSK-34-AKM-FINAL-MONO-DEPT
EXPNO                  5
PROCNO                 1

F2 - Acquisition Parameters
Date_          20241231
Time               0.41 h
INSTRUM  Avance Neo 400
PROBHD   Z163739_0226 (
PULPROG       deptsp135
TD                65536
SOLVENT             D2O
NS                   68
DS                    0
SWH           27777.777 Hz
FIDRES         0.847710 Hz
AQ            1.1796480 sec
RG                  101
DW               18.000 usec
DE                 6.50 usec
TE                295.7 K
CNST2       145.0000000
D1           1.00000000 sec
D2           0.00344828 sec
D12          0.00002000 sec
TD0                   1
SFO1        100.6242384 MHz
NUC1                13C
P1                 8.00 usec
P13             2000.00 usec
PLW0                  0 W
PLW1       101.36000061 W
SPNAM[5]    Crp60comp.4
SPOAL5            0.500
SPOFFS5               0 Hz
SPW5         9.91199970 W
SFO2        400.1316005 MHz
NUC2                 1H
CPDPRG[2        waltz65
P3                 8.00 usec
P4                16.00 usec
PCPD2             90.00 usec
PLW2        22.88299942 W
PLW12        0.18080001 W

F2 - Processing parameters
SI                32768
SF          100.6127685 MHz
WDW                  EM
SSB                   0
LB                 1.00 Hz
GB                    0
PC                 1.40
```

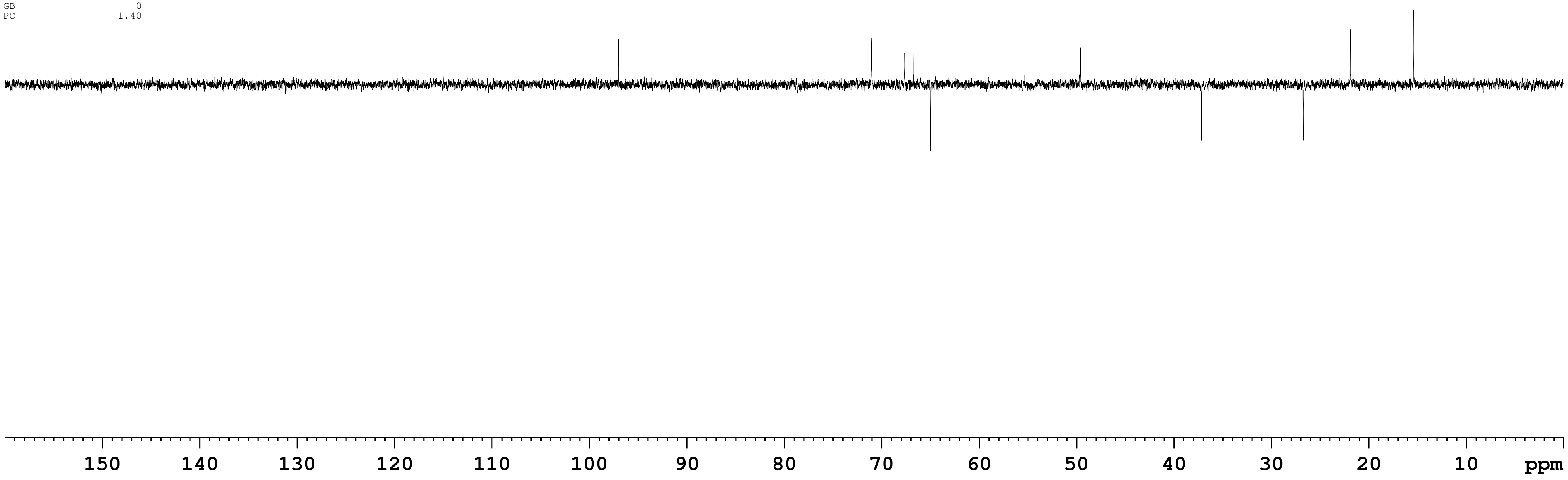

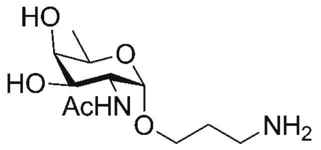

G10

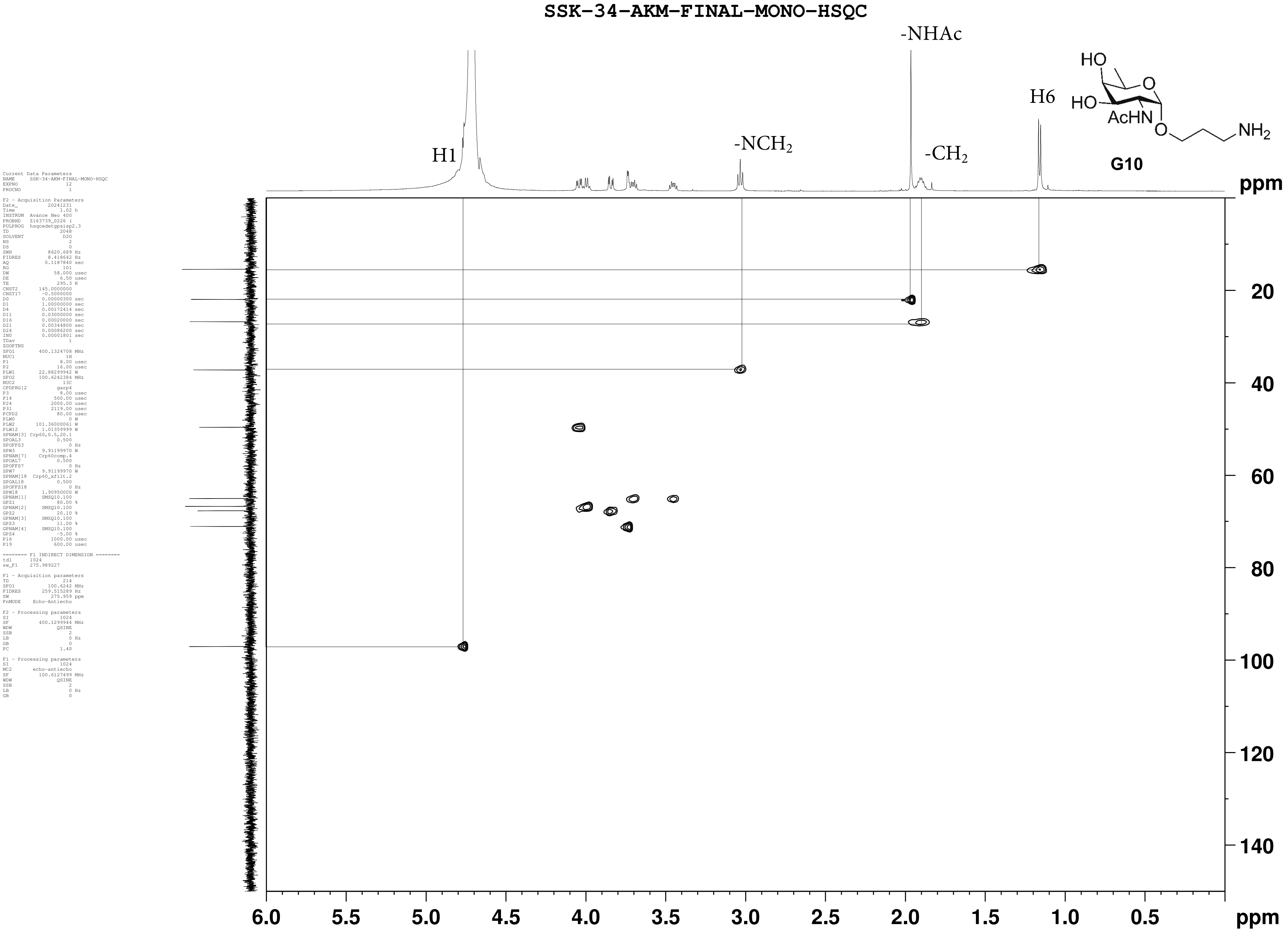
SSK-34-AKM-FINAL-MONO-HSQC
-NHAc
H6
H1
-NCH2
-CH2
HO
HO
AcHN
O
NH2
G10
ppm
20
40
60
80
100
120
140
6.0
5.5
5.0
4.5
4.0
3.5
3.0
2.5
2.0
1.5
1.0
0.5
ppm

SSK-34-AKM-G3

Current Data Parameters
NAME SSK-34-AKM-G3
EXPNO 1
PROCNO 1

F2 - Acquisition Parameters
Date_ 20251126
Time 10.
INSTRUM Avance
PROBHD Z154705_0128 (PI HR-BBO600S3-BBF/H/D-5.0
PULPROG
TD 28846
SOLVENT D2O
NS 50
DS 0
SWH 9615.385 Hz
FIDRES 0.666670 Hz
AQ 1.4999920 sec
RG 36
DW 52.000 usec
DE 6.63 usec
TE 298.6 K

F2 - Processing parameters
SI 65536
SF 600.3700000 M
WDW
SSB 0
LB 0.30 Hz
GB 0
PC 1.00

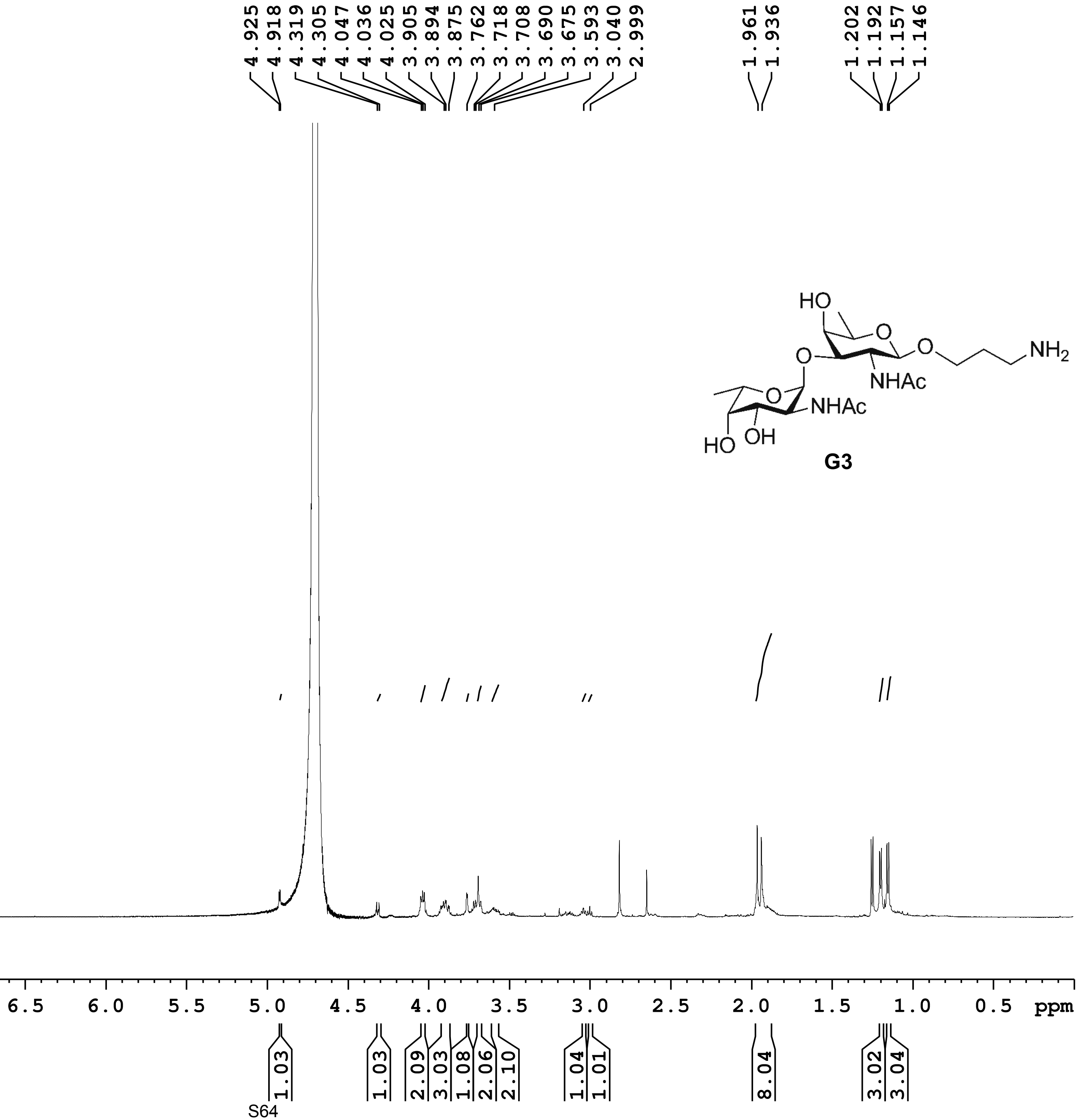

SSK-34-AKM-409-13C

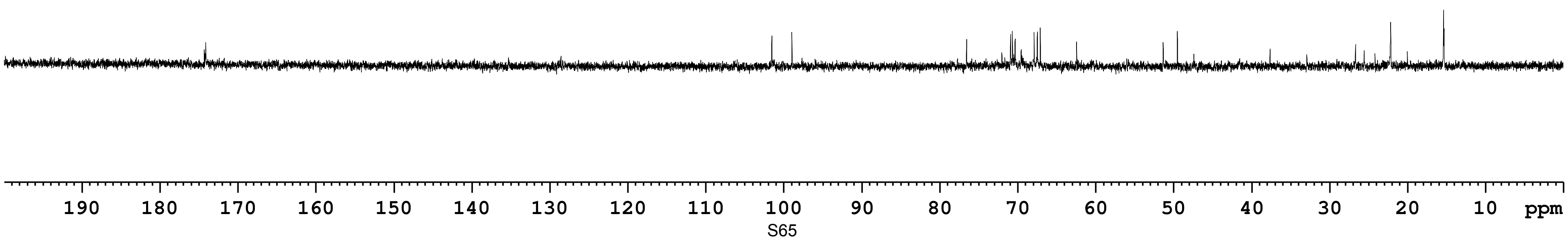

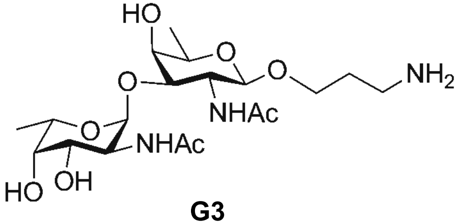

G3

SSK-34-AKM-409-DEPT

```
Current Data Parameters
NAME     SSK-34-AKM-409-DEPT
EXPNO                     2
PROCNO                    1

F2 - Acquisition Parameters
Date_          20231117
Time               1.45 h
INSTRUM           spect
PROBHD   Z104450_0346 (
PULPROG         dept135
TD                65536
SOLVENT             D2O
NS                 4954
DS                    0
SWH           27777.777 Hz
FIDRES         0.847710 Hz
AQ            1.1796480 sec
RG                 2050
DW               18.000 usec
DE                 6.50 usec
TE               3399.0 K
CNST2       145.0000000
D1           1.00000000 sec
D2           0.00344828 sec
D12          0.00002000 sec
TD0                   1
SFO1        100.6242389 MHz
NUC1                13C
P1                10.00 usec
P2                20.00 usec
PLW1        47.00000000 W
SFO2        400.1316005 MHz
NUC2                 1H
CPDPRG[2        waltz16
P3                15.00 usec
P4                30.00 usec
PCPD2             90.00 usec
PLW2         9.69999981 W
PLW12        0.26944000 W

F2 - Processing parameters
SI                32768
SF          100.6127680 MHz
WDW                  EM
SSB                   0
LB                 1.00 Hz
GB                    0
PC                 1.40
```

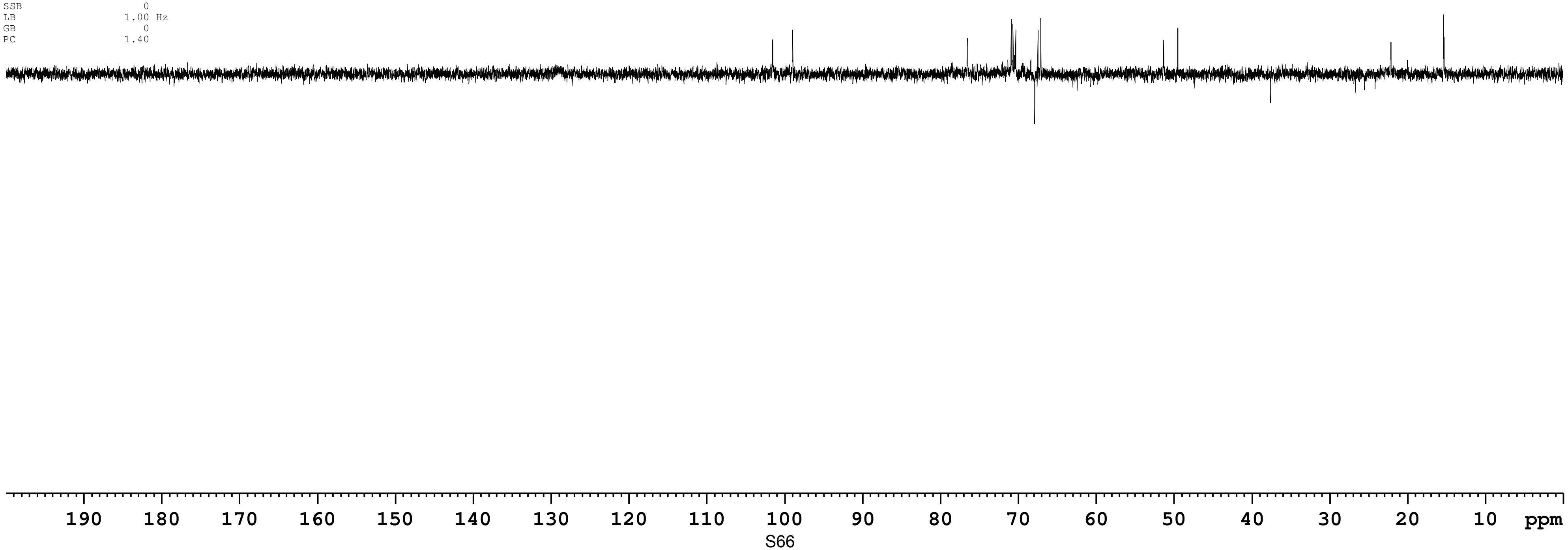

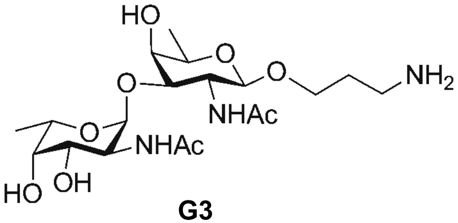

G3

SSK-34-AKM-409-HSQC

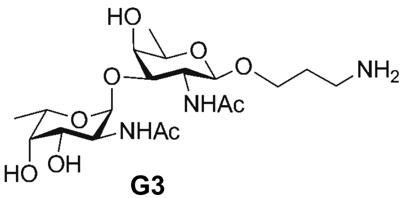

G3

```
Current Data Parameters
NAME      SSK-34-AKM-409-HSQC
EXPNO                    3
PROCNO                   1

F2 - Acquisition Parameters
Date_             20231111
Time                 23.39 h
INSTRUM              spect
PROBHD   Z104450_0346 (
PULPROG          hsqcetgp
TD                    2048
SOLVENT                D2O
NS                       8
DS                       0
SWH               3633.721 Hz
FIDRES            3.548556 Hz
AQ               0.2818048 sec
RG                    2050
DW                 137.600 usec
DE                    6.50 usec
TE                   156.5 K
CNST2          145.0000000
D0              0.00000300 sec
D1              1.00000000 sec
D4              0.00172414 sec
D11             0.03000000 sec
D16             0.00020000 sec
IN0             0.00002480 sec
TDav                     1
ZGOPTNS
SFO1           400.1318075 MHz
NUC1                    1H
P1                   15.00 usec
P2                   30.00 usec
PLW1            9.69999981 W
SFO2           100.6228162 MHz
NUC2                   13C
CPDPRG[2              garp
P3                   10.00 usec
P4                   20.00 usec
PCPD2                80.00 usec
PLW2           47.00000000 W
PLW12           0.73438001 W
GPNAM[1]          SINE.100
GPZ1                 80.00 %
GPNAM[2]          SINE.100
GPZ2                 20.10 %
P16                1000.00 usec

F1 - Acquisition parameters
TD                     140
SFO1              100.6228 MHz
FIDRES          288.018433 Hz
SW                 200.365 ppm
FnMODE        Echo-Antiecho

F2 - Processing parameters
SI                    2048
SF             400.1300000 MHz
WDW                  QSINE
SSB                      2
LB                       0 Hz
GB                       0
PC                    1.40

F1 - Processing parameters
SI                    1024
MC2          echo-antiecho
SF             100.6127690 MHz
WDW                  QSINE
SSB                      2
LB                       0 Hz
GB                       0
```

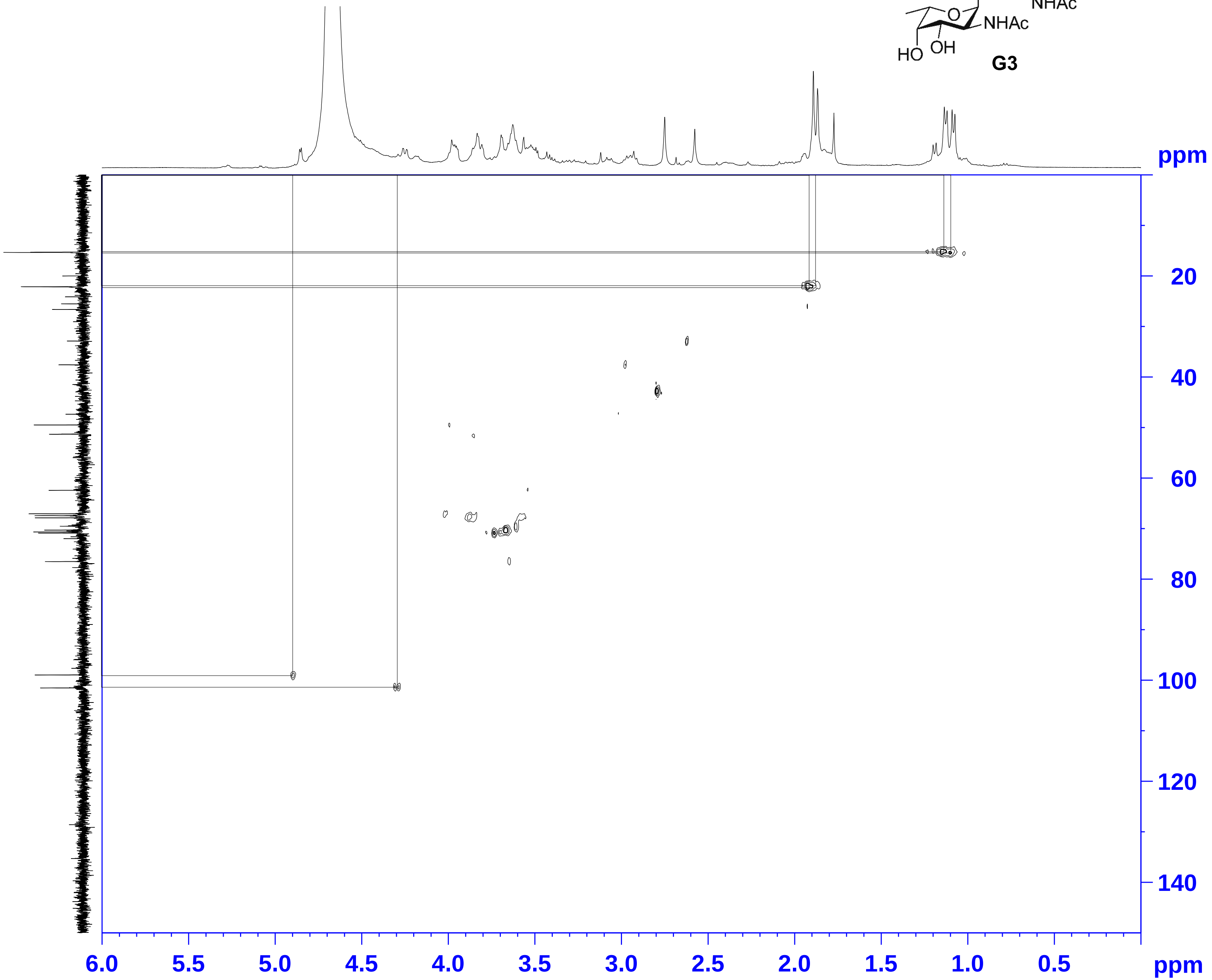

SSK-34-AKM-513-1H

7.991, 7.973, 7.617, 7.598, 7.536, 7.531, 7.516, 7.513, 7.485, 7.466, 7.446, 7.433, 7.419, 7.402, 7.390, 7.348, 7.338, 7.320, 7.310, 7.301, 7.289, 7.280, 7.259, 7.177, 7.160, 7.138, 7.122, 7.102, 5.606, 5.309, 5.097, 5.077, 4.866, 4.835, 4.743, 4.712, 4.694, 4.036, 4.029, 4.010, 4.002, 3.905, 3.888, 3.872, 3.859, 3.833, 3.767, 3.749, 3.734, 3.511, 3.498, 3.486, 3.473, 3.334, 3.318, 3.303, 1.114, 1.098

Current Data Parameters
NAME SSK-34-AKM-513-1H
EXPNO 1
PROCNO 1

F2 - Acquisition Parameters
Date_ 20240703
Time 17.30 h
INSTRUM spect
PROBHD Z104450_0346 (
PULPROG zg30
TD 54274
SOLVENT CDCl3
NS 13
DS 0
SWH 8223.685 Hz
FIDRES 0.303043 Hz
AQ 3.2998593 sec
RG 161
DW 60.800 usec
DE 6.50 usec
TE 3356.3 K
D1 1.00000000 sec
TD0 1
SFO1 400.1324710 MHz
NUC1 1H
P0 5.00 usec
P1 15.00 usec
PLW1 9.69999981 W

F2 - Processing parameters
SI 32768
SF 400.1300095 MHz
WDW EM
SSB 0
LB 0.30 Hz
GB 0
PC 1.00

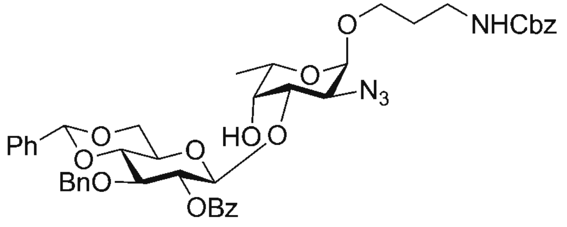

11

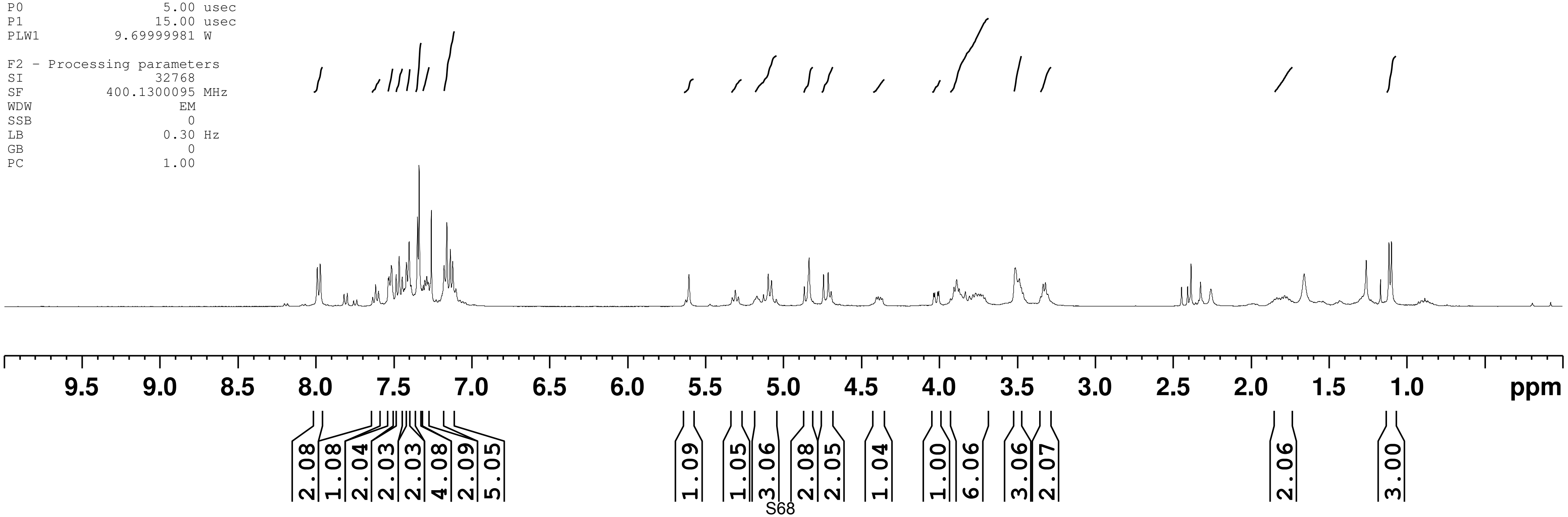

SSK-34-AKM-513-13C

```
Current Data Parameters
NAME      SSK-34-AKM-513-13C
EXPNO                  2
PROCNO                 1

F2 - Acquisition Parameters
Date_           20240703
Time               17.45 h
INSTRUM            spect
PROBHD   Z104450_0346 (
PULPROG          zgpg30
TD                 65536
SOLVENT            CDCl3
NS                   232
DS                     0
SWH            26041.666 Hz
FIDRES          0.794729 Hz
AQ             1.2582912 sec
RG                  2050
DW                19.200 usec
DE                  6.50 usec
TE                 290.9 K
D1            1.00000000 sec
D11           0.03000000 sec
TD0                    1
SFO1         100.6238364 MHz
NUC1                 13C
P0                  3.33 usec
P1                 10.00 usec
PLW1         47.00000000 W
SFO2         400.1316005 MHz
NUC2                  1H
CPDPRG[2         waltz16
PCPD2              90.00 usec
PLW2          9.69999981 W
PLW12         0.26944000 W
PLW13         0.13552999 W

F2 - Processing parameters
SI                 32768
SF           100.6127690 MHz
WDW                   EM
SSB                    0
LB                  1.00 Hz
GB                     0
PC                  1.40
```

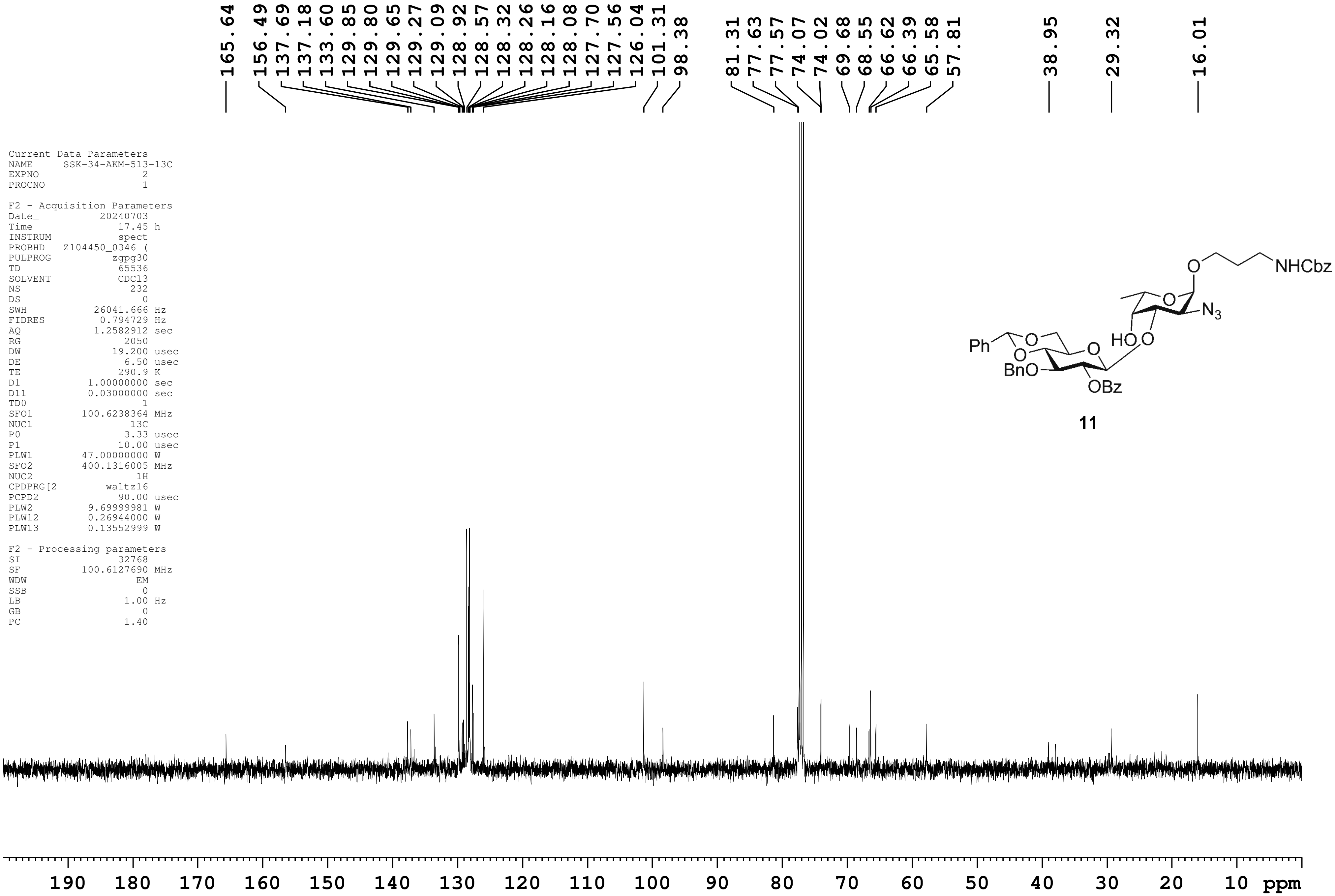

11

SSK-34-AKM-513-DEPT

```
Current Data Parameters
NAME     SSK-34-AKM-513-DEPT
EXPNO                      3
PROCNO                     1

F2 - Acquisition Parameters
Date_             20240703
Time               17.51 h
INSTRUM            spect
PROBHD   Z104450_0346 (
PULPROG          dept135
TD                 65536
SOLVENT            CDCl3
NS                   152
DS                     0
SWH          27777.777 Hz
FIDRES        0.847710 Hz
AQ           1.1796480 sec
RG                  2050
DW              18.000 usec
DE                6.50 usec
TE               595.1 K
CNST2       145.0000000
D1           1.00000000 sec
D2           0.00344828 sec
D12          0.00002000 sec
TD0                    1
SFO1       100.6242389 MHz
NUC1                 13C
P1               10.00 usec
P2               20.00 usec
PLW1       47.00000000 W
SFO2       400.1316005 MHz
NUC2                  1H
CPDPRG[2         waltz16
P3               15.00 usec
P4               30.00 usec
PCPD2            90.00 usec
PLW2        9.69999981 W
PLW12       0.26944000 W

F2 - Processing parameters
SI                 32768
SF         100.6127690 MHz
WDW                   EM
SSB                    0
LB                1.00 Hz
GB                     0
PC                  1.40
```

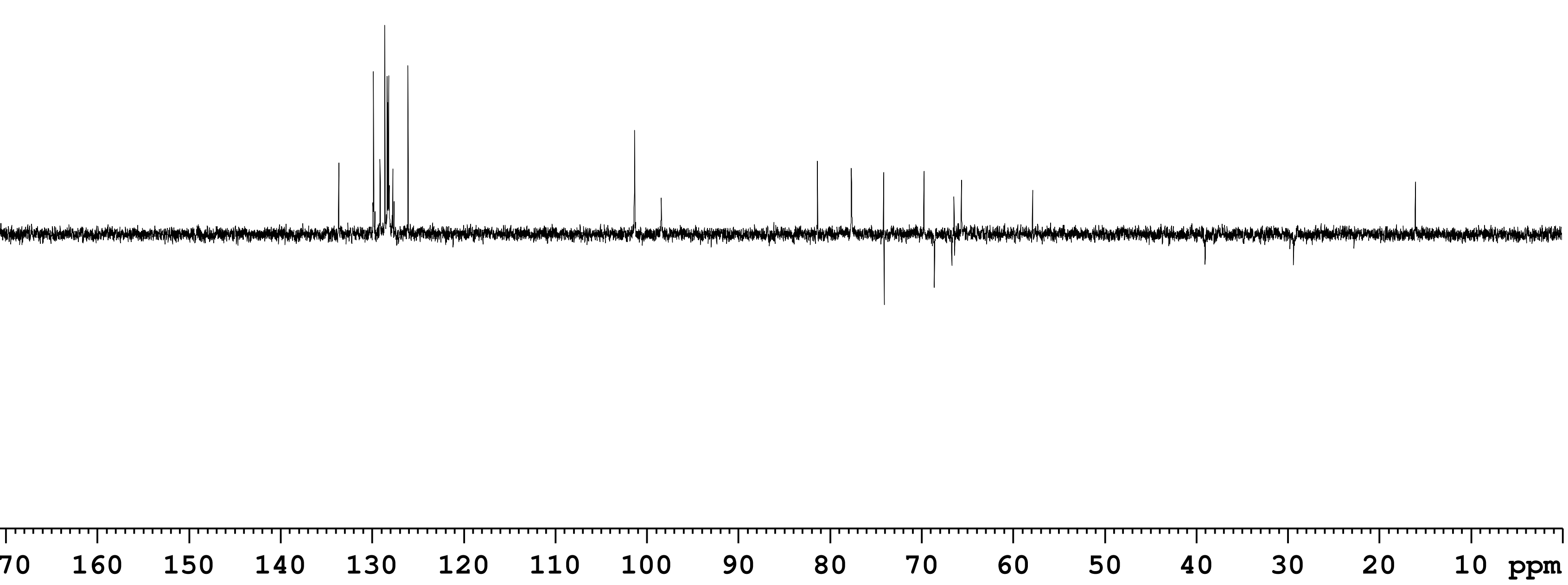

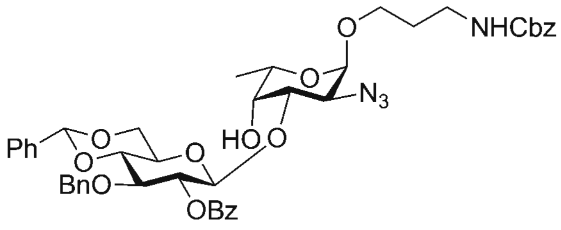

11

SSK-34-AKM-513-COSY

```
Current Data Parameters
NAME     SSK-34-AKM-513-COSY
EXPNO                    1
PROCNO                   1

F2 - Acquisition Parameters
Date_            20240703
Time                17.58 h
INSTRUM             spect
PROBHD   Z104450_0346 (
PULPROG          cosygpqf
TD                   2048
SOLVENT             CDCl3
NS                      2
DS                      0
SWH              2446.184 Hz
FIDRES           2.388851 Hz
AQ              0.4186112 sec
RG                     64
DW                204.400 usec
DE                   6.50 usec
TE                  475.9 K
D0             0.00000300 sec
D1             1.00000000 sec
D13            0.00000400 sec
D16            0.00020000 sec
IN0            0.00040880 sec
TDav                    1
SFO1          400.1312068 MHz
NUC1                   1H
P0                  15.00 usec
P1                  15.00 usec
PLW1           9.69999981 W
GPNAM[1]         SINE.100
GPZ1                10.00 %
P16               1000.00 usec

F1 - Acquisition parameters
TD                    128
SFO1             400.1312 MHz
FIDRES          38.221622 Hz
SW                  6.113 ppm
FnMODE                 QF

F2 - Processing parameters
SI                   1024
SF            400.1300000 MHz
WDW                  SINE
SSB                     0
LB                      0 Hz
GB                      0
PC                   1.40

F1 - Processing parameters
SI                   1024
MC2                    QF
SF            400.1300000 MHz
WDW                  SINE
SSB                     0
LB                      0 Hz
GB                      0
```

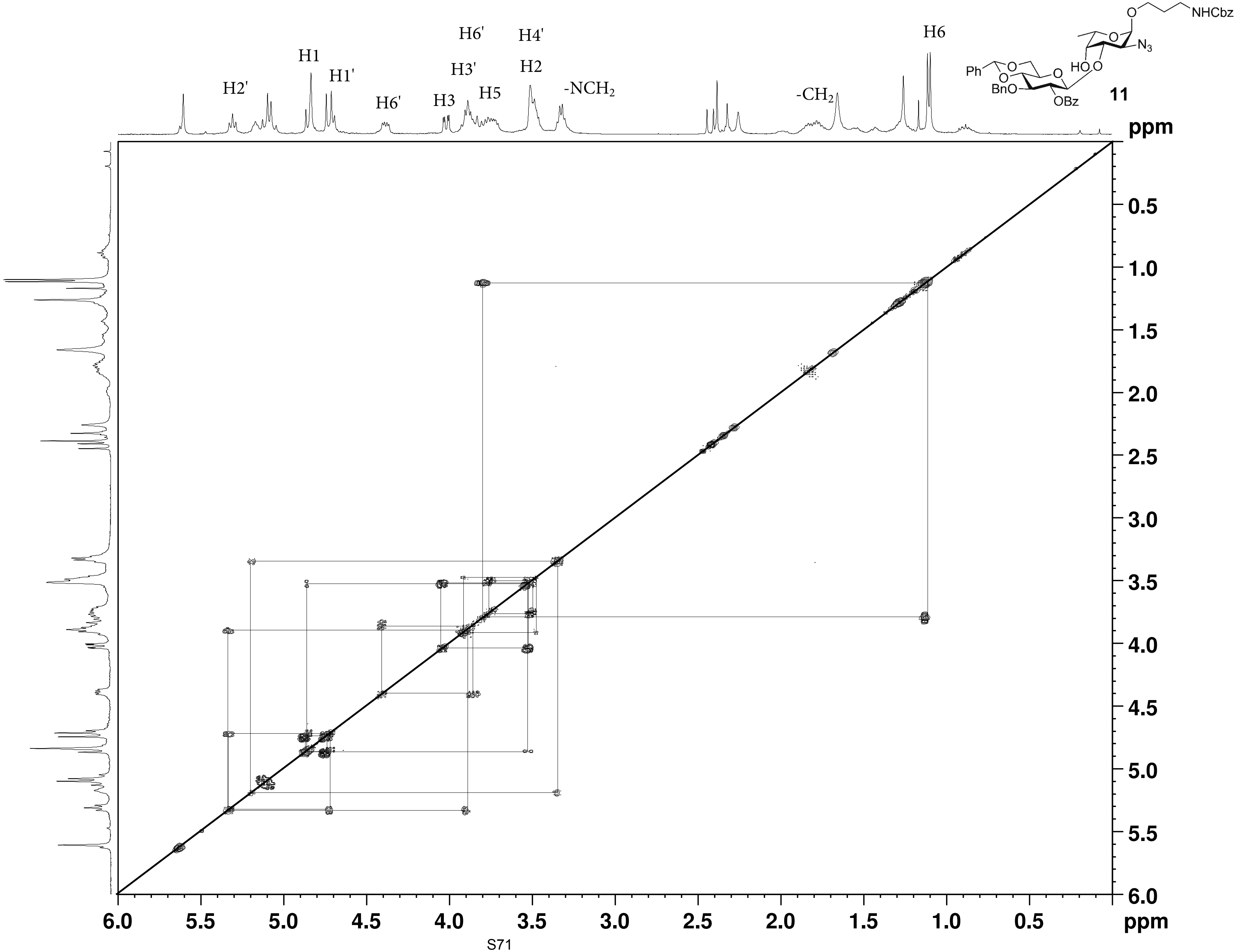

SSK-34-AKM-513-HSQC

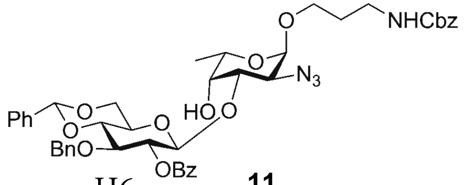

11

```
Current Data Parameters
NAME      SSK-34-AKM-513-HSQC
EXPNO                    5
PROCNO                   1

F2 - Acquisition Parameters
Date_             20240703
Time                 18.07 h
INSTRUM              spect
PROBHD   Z104450_0346 (
PULPROG          hsqcetgp
TD                    2048
SOLVENT              CDCl3
NS                       2
DS                       0
SWH               4045.307 Hz
FIDRES            3.950495 Hz
AQ               0.2531328 sec
RG                    2050
DW                 123.600 usec
DE                    6.50 usec
TE                   551.7 K
CNST2          145.0000000
D0              0.00000300 sec
D1              1.00000000 sec
D4              0.00172414 sec
D11             0.03000000 sec
D16             0.00020000 sec
IN0             0.00002500 sec
TDav                     1
ZGOPTNS
SFO1        400.1319991 MHz
NUC1                    1H
P1                   15.00 usec
P2                   30.00 usec
PLW1           9.69999981 W
SFO2        100.6227709 MHz
NUC2                   13C
CPDPRG[2              garp
P3                   10.00 usec
P4                   20.00 usec
PCPD2                80.00 usec
PLW2          47.00000000 W
PLW12          0.73438001 W
GPNAM[1]          SINE.100
GPZ1                 80.00 %
GPNAM[2]          SINE.100
GPZ2                 20.10 %
P16                1000.00 usec

F1 - Acquisition parameters
TD                     209
SFO1              100.6228 MHz
FIDRES          191.387558 Hz
SW                 198.762 ppm
FnMODE     Echo-Antiecho

F2 - Processing parameters
SI                    2048
SF             400.1300000 MHz
WDW                  QSINE
SSB                      2
LB                       0 Hz
GB                       0
PC                    1.40

F1 - Processing parameters
SI                    1024
MC2          echo-antiecho
SF             100.6127690 MHz
WDW                  QSINE
SSB                      2
LB                       0 Hz
GB                       0
```

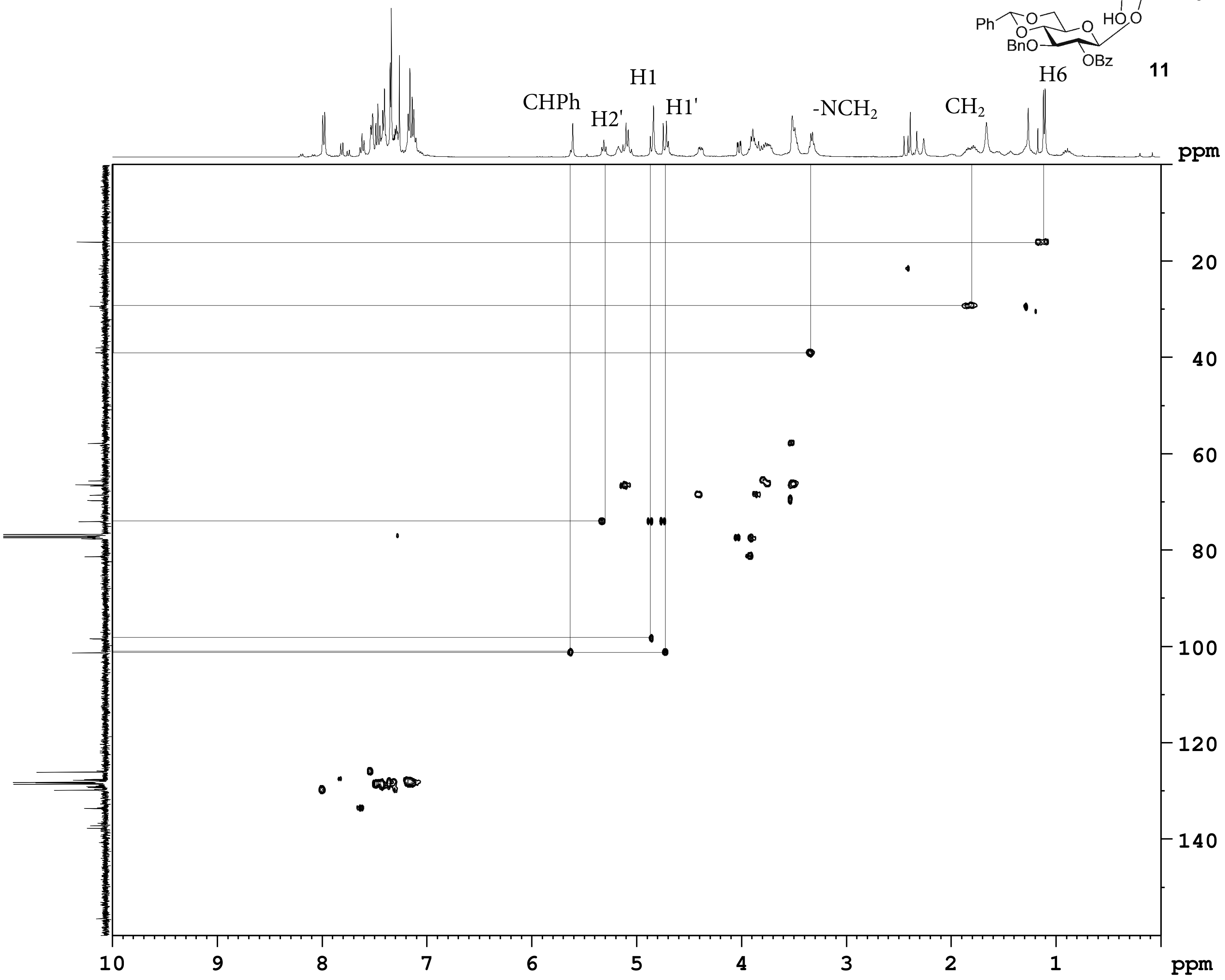

SSK-34-AKM-513-HMBC

```
Current Data Parameters
NAME      SSK-34-AKM-513-HMBC
EXPNO                     6
PROCNO                    1

F2 - Acquisition Parameters
Date_              20240703
Time                 18.16 h
INSTRUM              spect
PROBHD     Z104450_0346 (
PULPROG        hmbcgpndqf
TD                   4048
SOLVENT              CDCl3
NS                      2
DS                      0
SWH              4032.258 Hz
FIDRES           1.992222 Hz
AQ              0.5019520 sec
RG                   2050
DW                124.000 usec
DE                   6.50 usec
TE                  604.8 K
CNST13          8.0000000
D0             0.00000300 sec
D1             1.00000000 sec
D6             0.06250000 sec
D16            0.00020000 sec
IN0            0.00002600 sec
TDav                    1
SFO1          400.1319965 MHz
NUC1                   1H
P1                  15.00 usec
P2                  30.00 usec
PLW1           9.69999981 W
SFO2          100.6225451 MHz
NUC2                  13C
P3                  10.00 usec
PLW2          47.00000000 W
GPNAM[1]         SINE.100
GPZ1                50.00 %
GPNAM[2]         SINE.100
GPZ2                30.00 %
GPNAM[3]         SINE.100
GPZ3                40.10 %
P16               1000.00 usec

F1 - Acquisition parameters
TD                    179
SFO1             100.6225 MHz
FIDRES         214.868927 Hz
SW                191.118 ppm
FnMODE                 QF

F2 - Processing parameters
SI                   4096
SF            400.1300000 MHz
WDW                  SINE
SSB                     0
LB                      0 Hz
GB                      0
PC                   1.40

F1 - Processing parameters
SI                   1024
MC2                    QF
SF            100.6127690 MHz
WDW                  SINE
SSB                     0
LB                      0 Hz
GB                      0
```

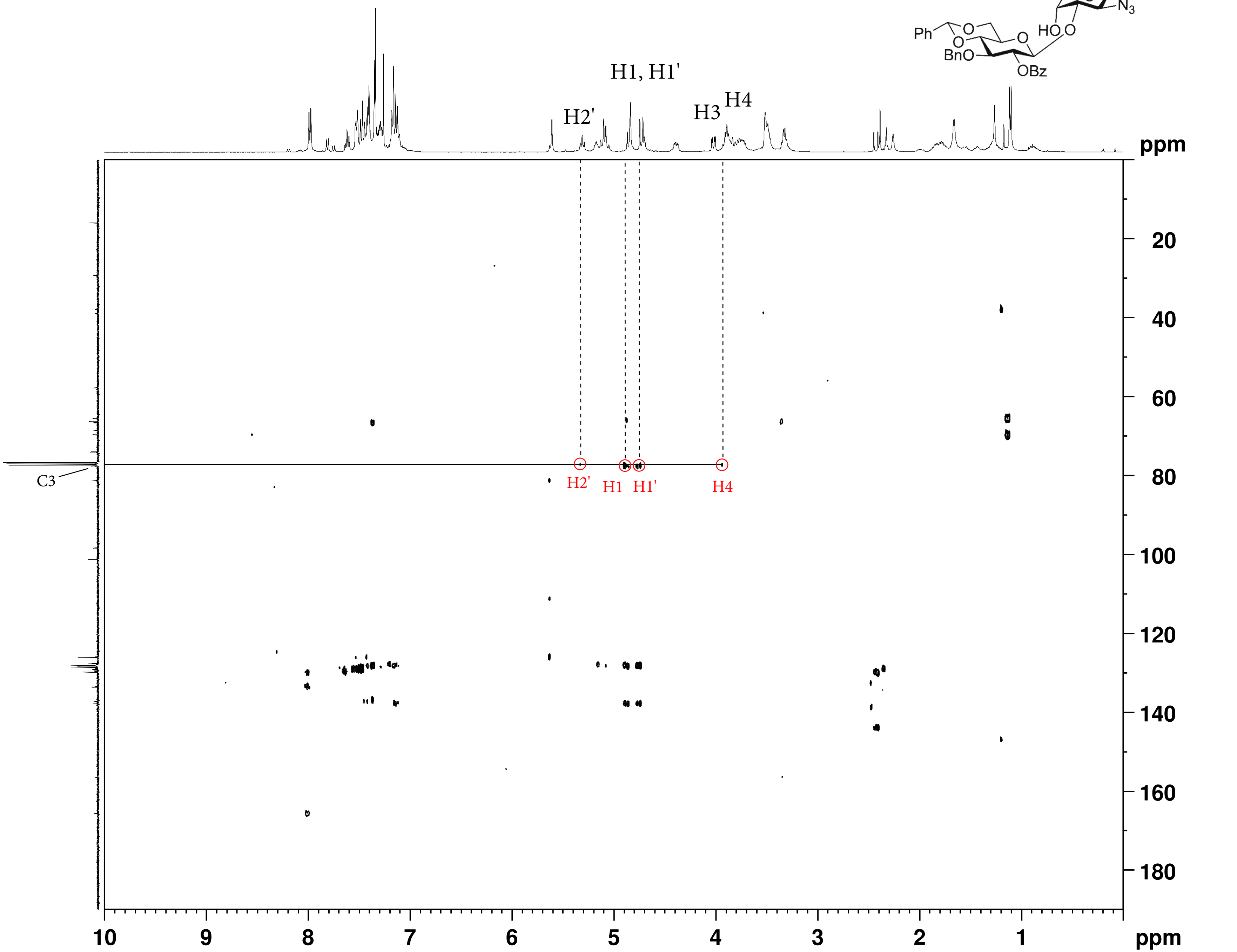

SSK-34-AKM-GL-DI-OAc-1H

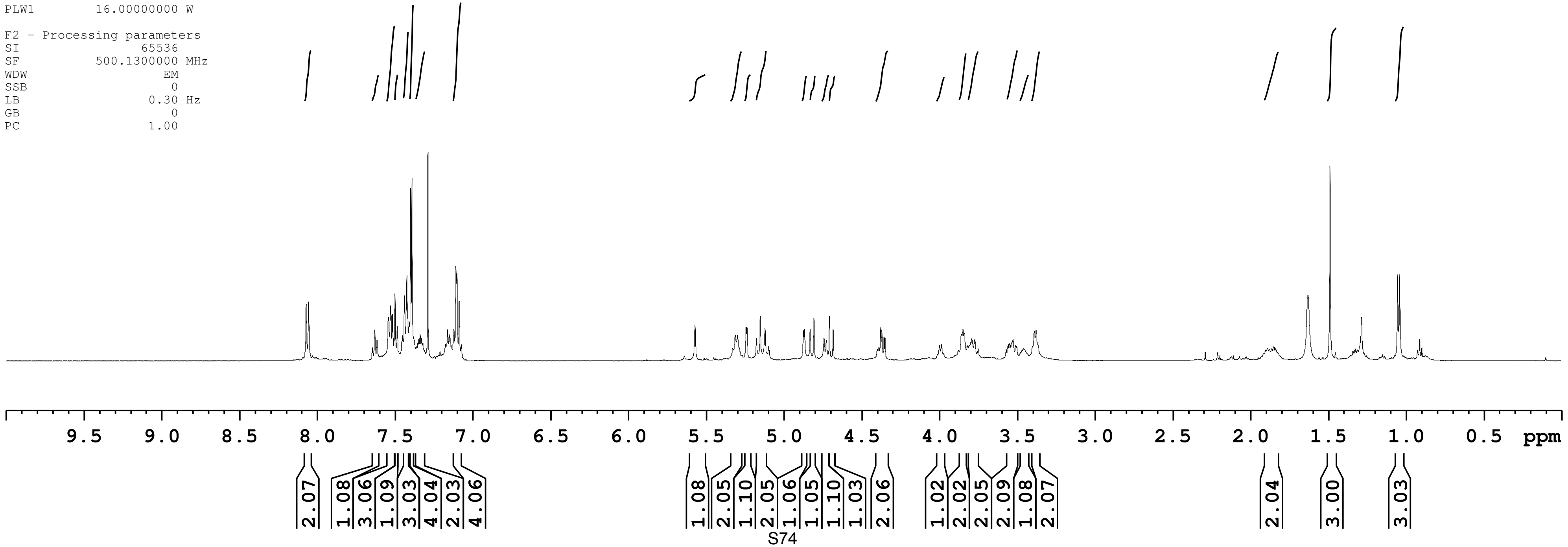

Current Data Parameters
NAME SSK-34-AKM-GL-DI-OAc-1H
EXPNO 1
PROCNO 1

F2 - Acquisition Parameters
Date_ 20250216
Time 20.46 h
INSTRUM spect
PROBHD Z119470_0087 (
PULPROG zg30
TD 65536
SOLVENT CDCl3
NS 16
DS 2
SWH 10000.000 Hz
FIDRES 0.305176 Hz
AQ 3.2767999 sec
RG 119.07
DW 50.000 usec
DE 6.50 usec
TE 297.6 K
D1 1.00000000 sec
TD0 1
SFO1 500.1330885 MHz
NUC1 1H
P0 4.45 usec
P1 13.35 usec
PLW1 16.00000000 W

F2 - Processing parameters
SI 65536
SF 500.1300000 MHz
WDW EM
SSB 0
LB 0.30 Hz
GB 0
PC 1.00

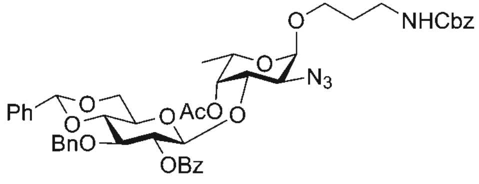

S1

SSK-34-AKM-GL-DI-OAc-13C

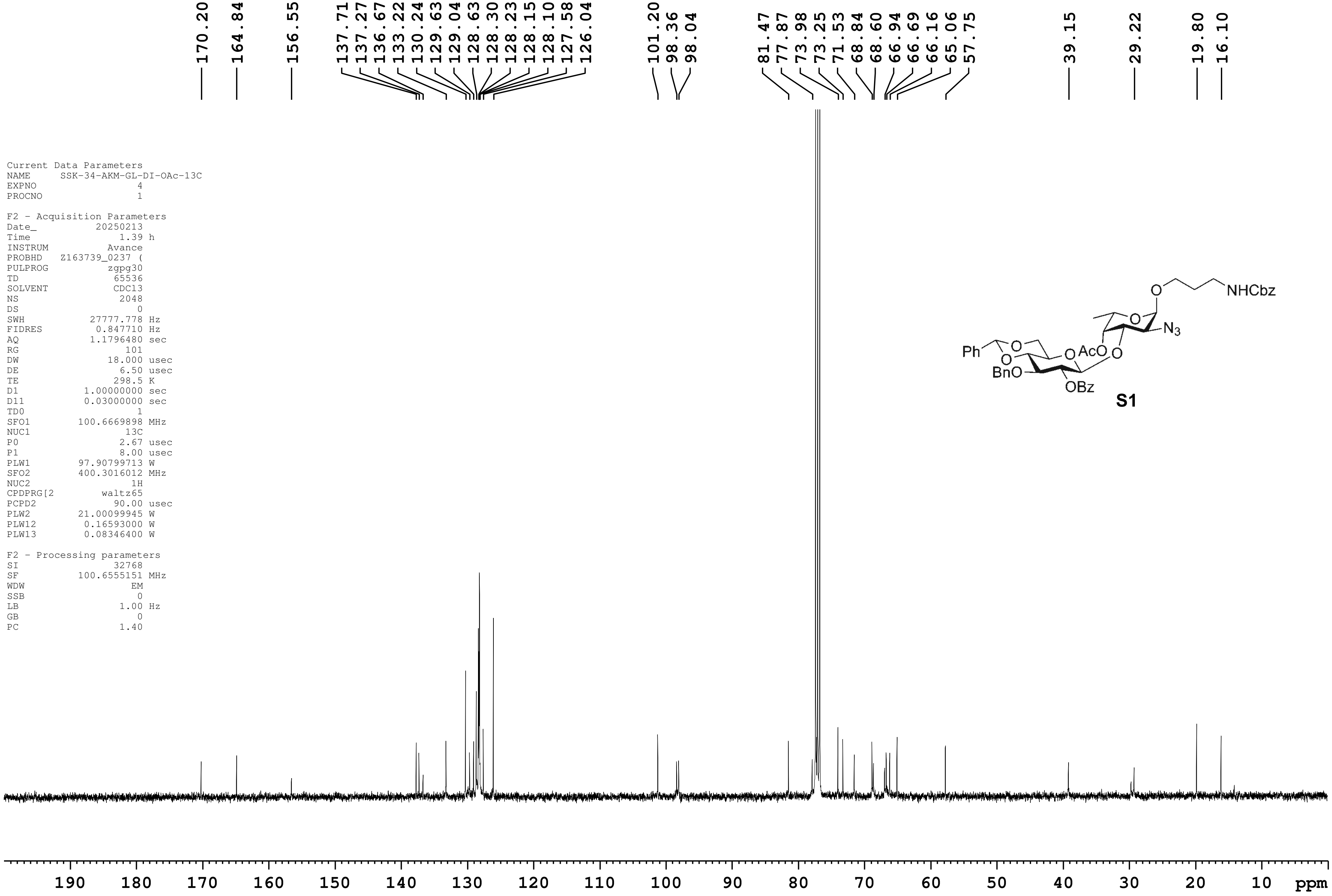

Current Data Parameters
NAME SSK-34-AKM-GL-DI-OAc-13C
EXPNO 4
PROCNO 1

F2 - Acquisition Parameters
Date_ 20250213
Time 1.39 h
INSTRUM Avance
PROBHD Z163739_0237 (
PULPROG zgpg30
TD 65536
SOLVENT CDCl3
NS 2048
DS 0
SWH 27777.778 Hz
FIDRES 0.847710 Hz
AQ 1.1796480 sec
RG 101
DW 18.000 usec
DE 6.50 usec
TE 298.5 K
D1 1.00000000 sec
D11 0.03000000 sec
TD0 1
SFO1 100.6669898 MHz
NUC1 13C
P0 2.67 usec
P1 8.00 usec
PLW1 97.90799713 W
SFO2 400.3016012 MHz
NUC2 1H
CPDPRG[2 waltz65
PCPD2 90.00 usec
PLW2 21.00099945 W
PLW12 0.16593000 W
PLW13 0.08346400 W

F2 - Processing parameters
SI 32768
SF 100.6555151 MHz
WDW EM
SSB 0
LB 1.00 Hz
GB 0
PC 1.40

S1

SSK-34-AKM-GL-DI-OAc-DEPT

```
Current Data Parameters
NAME      SSK-34-AKM-GL-DI-OAc-DEPT
EXPNO                 5
PROCNO                1

F2 - Acquisition Parameters
Date_           20250213
Time               2.17 h
INSTRUM          Avance
PROBHD   Z163739_0237 (
PULPROG        deptsp135
TD                65536
SOLVENT           CDCl3
NS                 1000
DS                    0
SWH           27777.778 Hz
FIDRES         0.847710 Hz
AQ            1.1796480 sec
RG                  101
DW               18.000 usec
DE                 6.50 usec
TE                298.6 K
CNST2       145.0000000
D1           1.00000000 sec
D2           0.00344828 sec
D12          0.00002000 sec
TD0                   1
SFO1        100.6669898 MHz
NUC1                13C
P1                 8.00 usec
P13             2000.00 usec
PLW0                  0 W
PLW1        97.90799713 W
SPNAM[5]    Crp60comp.4
SPOAL5            0.500
SPOFFS5               0 Hz
SPW5         9.57390022 W
SFO2        400.3016012 MHz
NUC2                 1H
CPDPRG[2         waltz65
P3                 8.00 usec
P4                16.00 usec
PCPD2             90.00 usec
PLW2        21.00099945 W
PLW12        0.16593000 W

F2 - Processing parameters
SI                32768
SF          100.6555151 MHz
WDW                  EM
SSB                   0
LB                 1.00 Hz
GB                    0
PC                 1.40
```

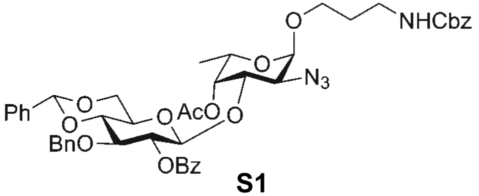

S1

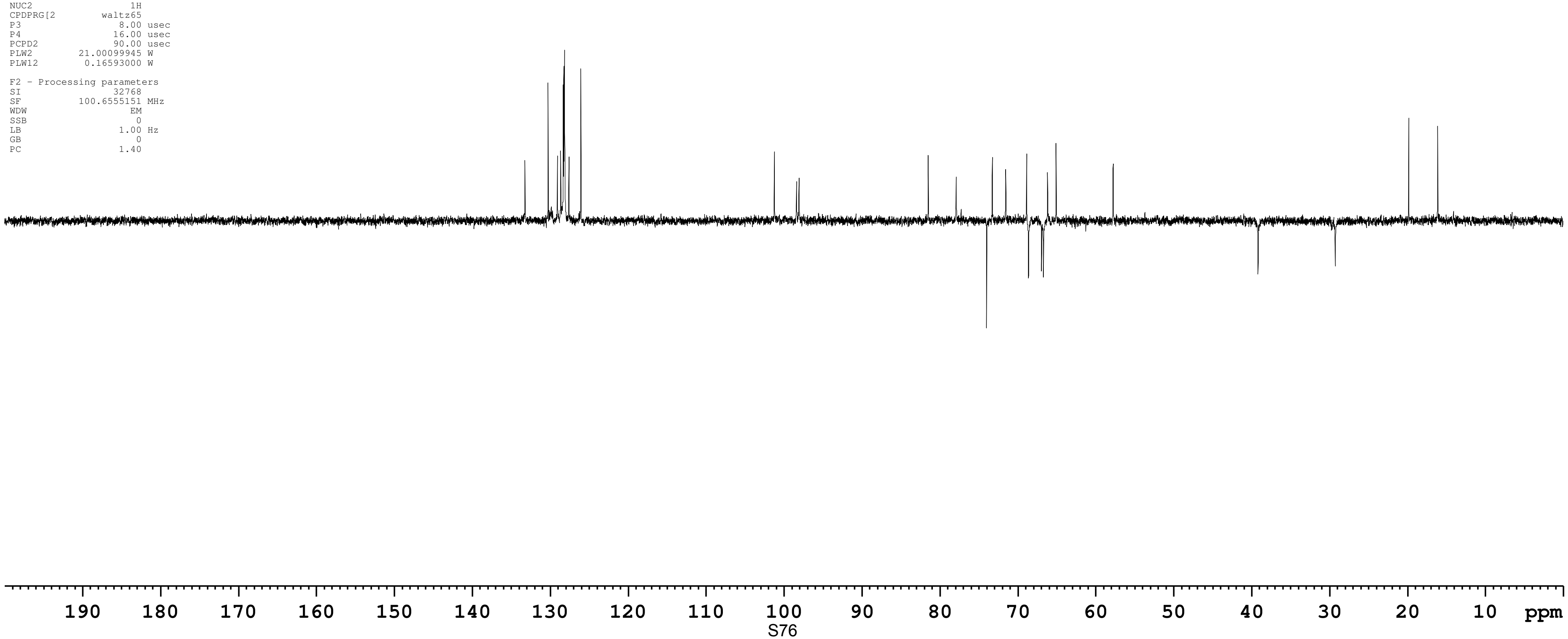

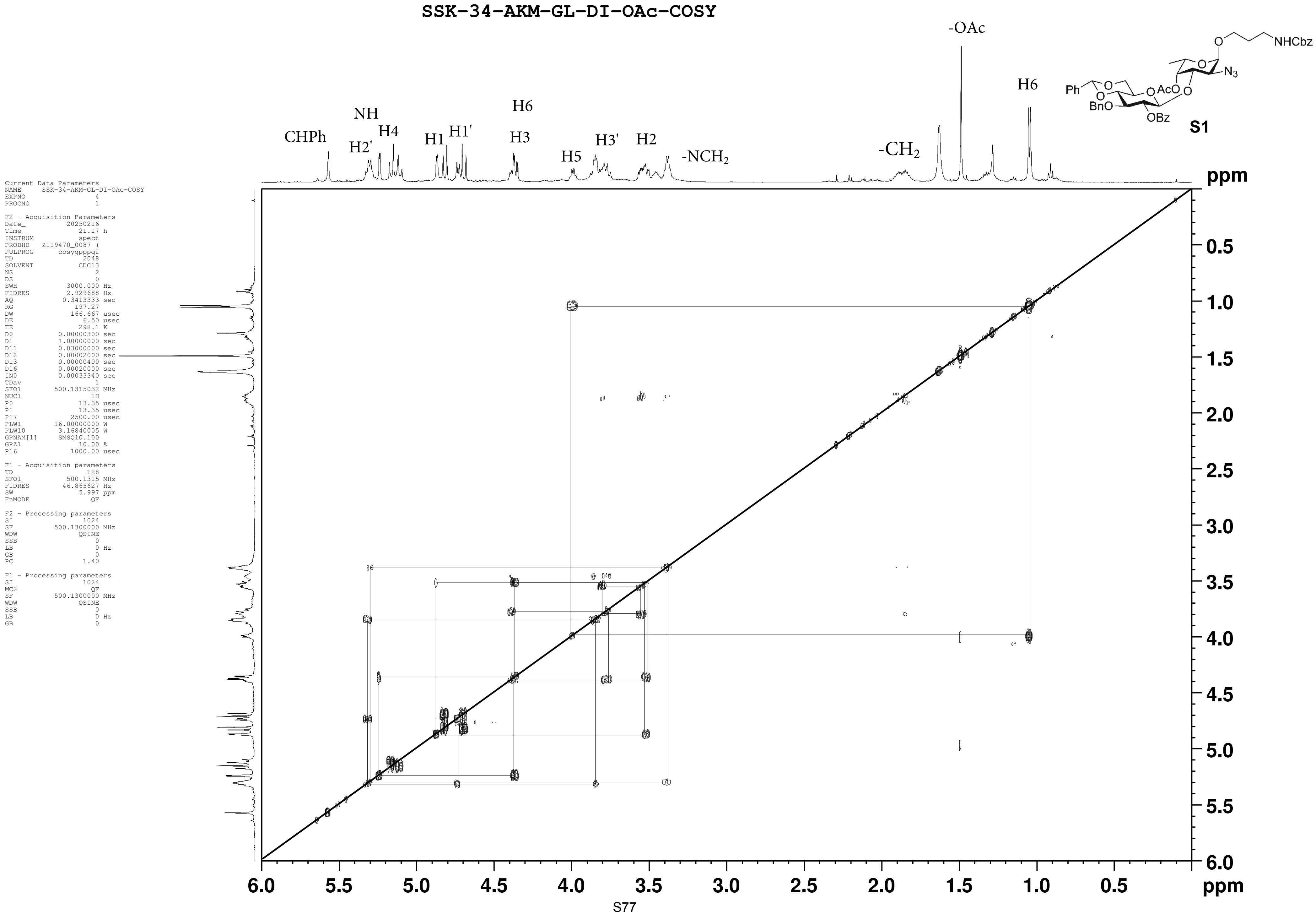
SSK-34-AKM-GL-DI-OAc-COSY
-OAc
H6
NH
H6
CHPh
H2'
H4
H1
H1'
H3
H5
H3'
H2
-NCH$_2$
-CH$_2$
NHCbz
N_3
Ph
AcO
BnO
OBz
S1
ppm
Current Data Parameters
NAME SSK-34-AKM-GL-DI-OAc-COSY
EXPNO 4
PROCNO 1
F2 - Acquisition Parameters
Date_ 20250216
Time 21.17 h
INSTRUM spect
PROBHD Z119470_0087 (
PULPROG cosygpppqf
TD 2048
SOLVENT CDCl3
NS 2
DS 0
SWH 3000.000 Hz
FIDRES 2.929688 Hz
AQ 0.3413333 sec
RG 197.27
DW 166.667 usec
DE 6.50 usec
TE 298.1 K
D0 0.00000300 sec
D1 1.00000000 sec
D11 0.03000000 sec
D12 0.00002000 sec
D13 0.00000400 sec
D16 0.00020000 sec
IN0 0.00033340 sec
TDav 1
SFO1 500.1315032 MHz
NUC1 1H
P0 13.35 usec
P1 13.35 usec
P17 2500.00 usec
PLW1 16.00000000 W
PLW10 3.16840005 W
GPNAM[1] SMSQ10.100
GPZ1 10.00 %
P16 1000.00 usec
F1 - Acquisition parameters
TD 128
SFO1 500.1315 MHz
FIDRES 46.865627 Hz
SW 5.997 ppm
FnMODE QF
F2 - Processing parameters
SI 1024
SF 500.1300000 MHz
WDW QSINE
SSB 0
LB 0 Hz
GB 0
PC 1.40
F1 - Processing parameters
SI 1024
MC2 QF
SF 500.1300000 MHz
WDW QSINE
SSB 0
LB 0 Hz
GB 0
0.5
1.0
1.5
2.0
2.5
3.0
3.5
4.0
4.5
5.0
5.5
6.0
ppm

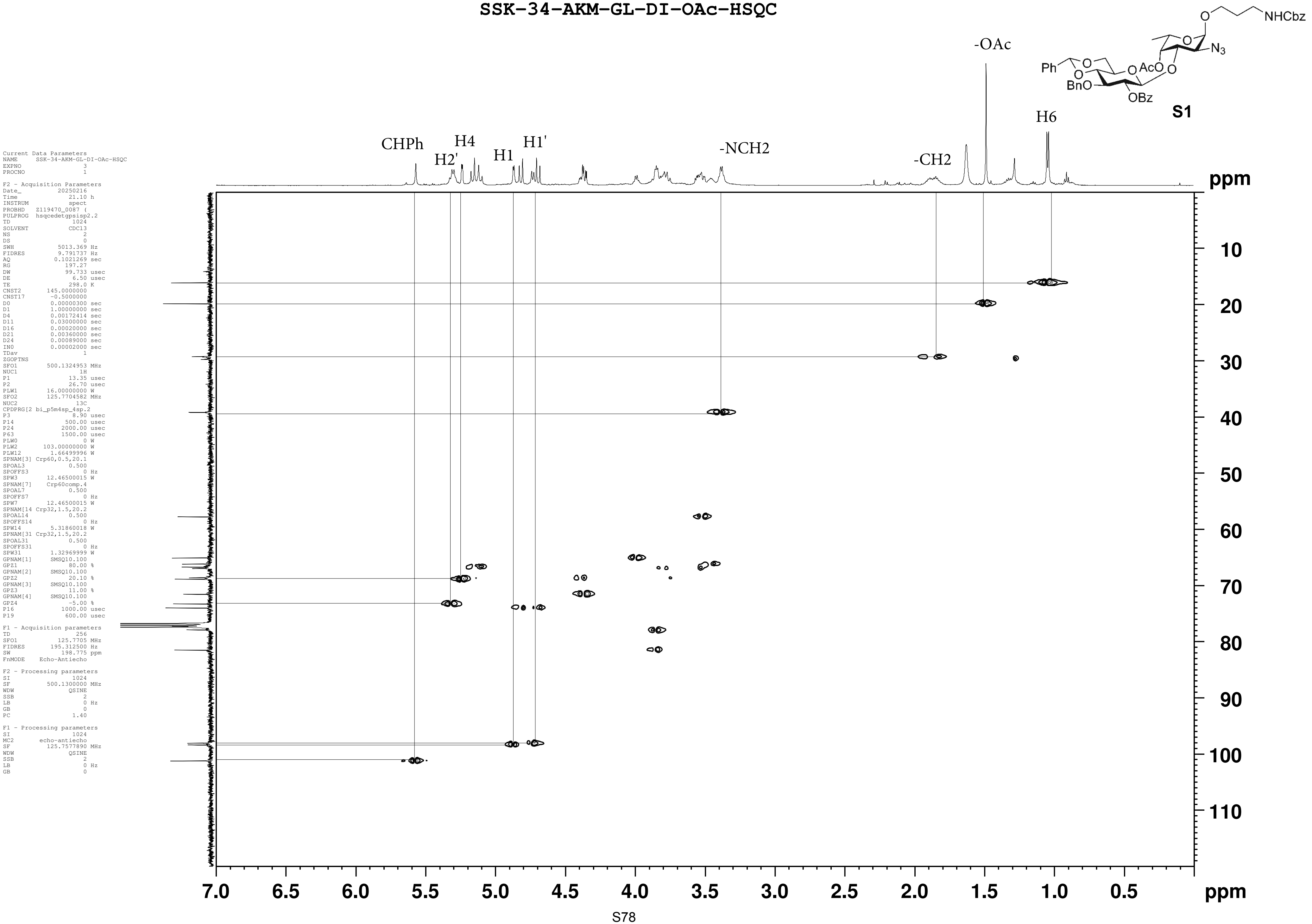
SSK-34-AKM-GL-DI-OAc-HSQC
Current Data Parameters
NAME SSK-34-AKM-GL-DI-OAc-HSQC
EXPNO 3
PROCNO 1
F2 - Acquisition Parameters
Date_ 20250216
Time 21.10 h
INSTRUM spect
PROBHD Z119470_0087 (
PULPROG hsqcedetgpsisp2.2
TD 1024
SOLVENT CDCl3
NS 2
DS 0
SWH 5013.369 Hz
FIDRES 9.791737 Hz
AQ 0.1021269 sec
RG 197.27
DW 99.733 usec
DE 6.50 usec
TE 298.0 K
CNST2 145.0000000
CNST17 -0.5000000
D0 0.00000300 sec
D1 1.00000000 sec
D4 0.00172414 sec
D11 0.03000000 sec
D16 0.00020000 sec
D21 0.00360000 sec
D24 0.00089000 sec
IN0 0.00002000 sec
TDav 1
ZGOPTNS
SFO1 500.1324953 MHz
NUC1 1H
P1 13.35 usec
P2 26.70 usec
PLW1 16.00000000 W
SFO2 125.7704582 MHz
NUC2 13C
CPDPRG[2 bi_p5m4sp_4sp.2
P3 8.90 usec
P14 500.00 usec
P24 2000.00 usec
P63 1500.00 usec
PLW0 0 W
PLW2 103.00000000 W
PLW12 1.66499996 W
SPNAM[3] Crp60,0.5,20.1
SPOAL3 0.500
SPOFFS3 0 Hz
SPW3 12.46500015 W
SPNAM[7] Crp60comp.4
SPOAL7 0.500
SPOFFS7 0 Hz
SPW7 12.46500015 W
SPNAM[14 Crp32,1.5,20.2
SPOAL14 0.500
SPOFFS14 0 Hz
SPW14 5.31860018 W
SPNAM[31 Crp32,1.5,20.2
SPOAL31 0.500
SPOFFS31 0 Hz
SPW31 1.32969999 W
GPNAM[1] SMSQ10.100
GPZ1 80.00 %
GPNAM[2] SMSQ10.100
GPZ2 20.10 %
GPNAM[3] SMSQ10.100
GPZ3 11.00 %
GPNAM[4] SMSQ10.100
GPZ4 -5.00 %
P16 1000.00 usec
P19 600.00 usec
F1 - Acquisition parameters
TD 256
SFO1 125.7705 MHz
FIDRES 195.312500 Hz
SW 198.775 ppm
FnMODE Echo-Antiecho
F2 - Processing parameters
SI 1024
SF 500.1300000 MHz
WDW QSINE
SSB 2
LB 0 Hz
GB 0
PC 1.40
F1 - Processing parameters
SI 1024
MC2 echo-antiecho
SF 125.7577890 MHz
WDW QSINE
SSB 2
LB 0 Hz
GB 0
CHPh
H2'
H4
H1
H1'
-NCH2
-CH2
-OAc
H6
Ph
BnO
OBz
AcO
N3
NHCbz
S1
ppm
10
20
30
40
50
60
70
80
90
100
110
7.0
6.5
6.0
5.5
5.0
4.5
4.0
3.5
3.0
2.5
2.0
1.5
1.0
0.5
ppm

SSK-34-AKM-DS-GL-OAc-HMBC

```
Current Data Parameters
NAME      SSK-34-AKM-DS-GL-OAc-HMBC
EXPNO                     6
PROCNO                    1

F2 - Acquisition Parameters
Date_              20251121
Time                   7.28 h
INSTRUM               spect
PROBHD   Z119470_0087 (
PULPROG    hmbcetgpl3nd
TD                     4096
SOLVENT               CDCl3
NS                       64
DS                        0
SWH                5020.080 Hz
FIDRES             2.451211 Hz
AQ                0.4079616 sec
RG                   197.27
DW                   99.600 usec
DE                     6.50 usec
TE                    297.5 K
CNST6           120.0000000
CNST7           170.0000000
CNST13            8.0000000
D0               0.00000300 sec
D1               1.00000000 sec
D6               0.06250000 sec
D16              0.00020000 sec
IN0              0.00002080 sec
TDav                      1
SFO1            500.1324963 MHz
NUC1                     1H
P1                    13.35 usec
P2                    26.70 usec
PLW1            16.00000000 W
SFO2            125.7697576 MHz
NUC2                    13C
P3                     8.90 usec
P24                 2000.00 usec
PLW2           103.00000000 W
SPNAM[7]       Crp60comp.4
SPOAL7                0.500
SPOFFS7                   0 Hz
SPW7            12.46500015 W
GPNAM[1]        SMSQ10.100
GPZ1                  80.00 %
GPNAM[3]        SMSQ10.100
GPZ3                  14.00 %
GPNAM[4]        SMSQ10.100
GPZ4                  -8.00 %
GPNAM[5]        SMSQ10.100
GPZ5                  -4.00 %
GPNAM[6]        SMSQ10.100
GPZ6                  -2.00 %
P16                 1000.00 usec

F1 - Acquisition parameters
TD                      256
SFO1               125.7698 MHz
FIDRES           187.800476 Hz
SW                  191.131 ppm
FnMODE       Echo-Antiecho

F2 - Processing parameters
SI                     2048
SF              500.1300000 MHz
WDW                    SINE
SSB                       4
LB                        0 Hz
GB                        0
PC                     1.40

F1 - Processing parameters
SI                     1024
MC2          echo-antiecho
SF              125.7577890 MHz
WDW                   QSINE
SSB                       2
LB                        0 Hz
GB                        0
```

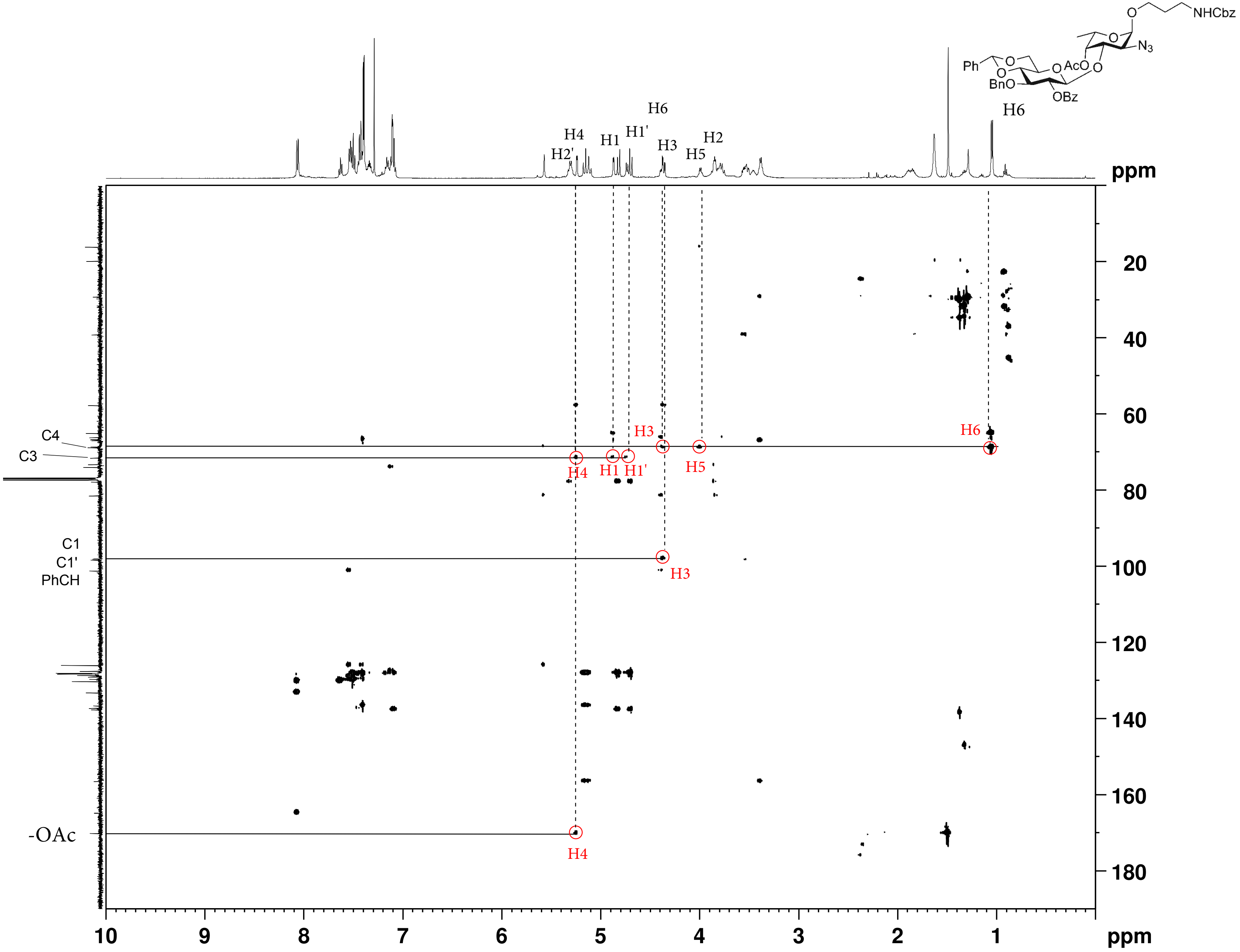

SSK-34-AKM-514-1H

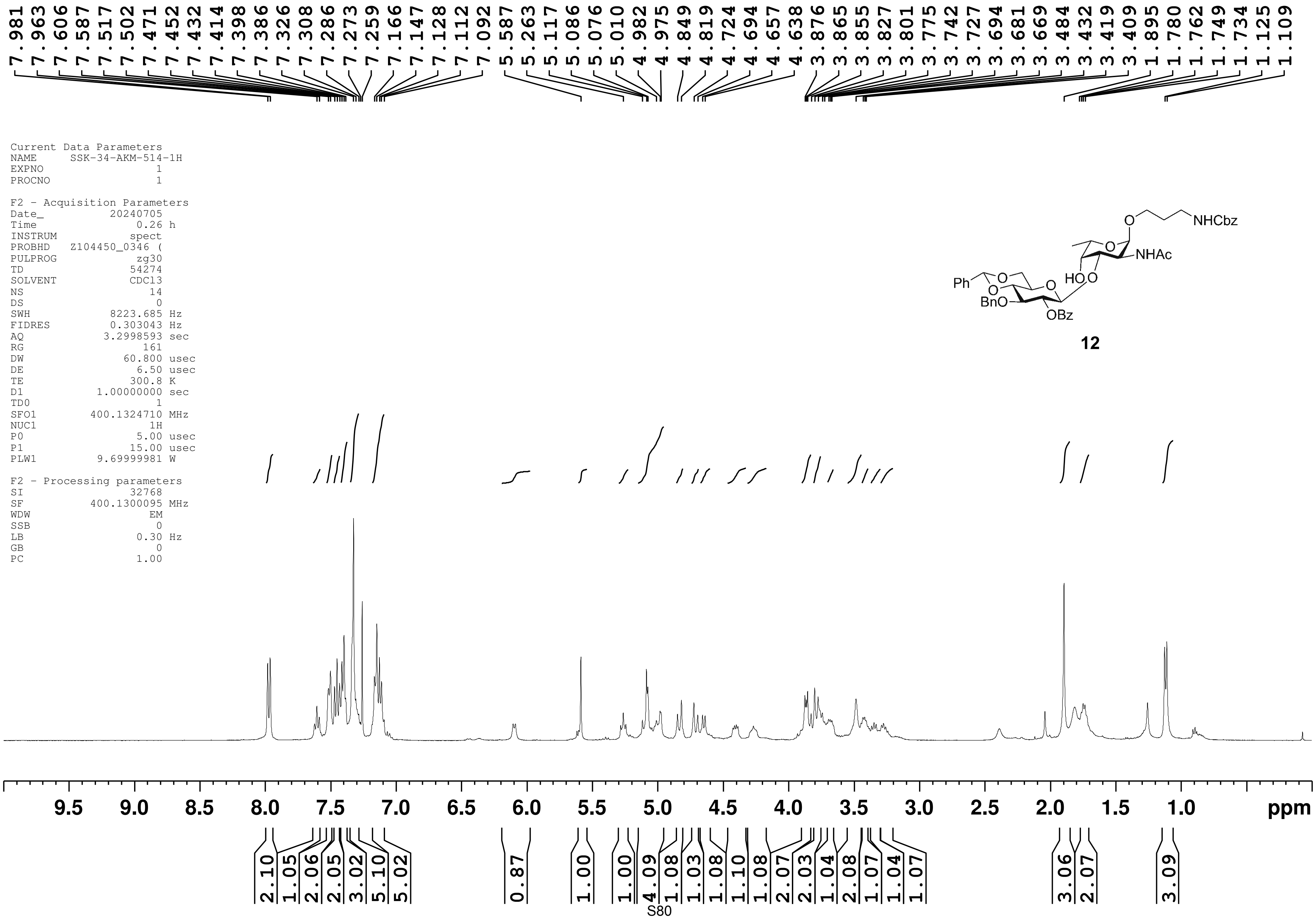

Current Data Parameters
NAME SSK-34-AKM-514-1H
EXPNO 1
PROCNO 1

F2 - Acquisition Parameters
Date_ 20240705
Time 0.26 h
INSTRUM spect
PROBHD Z104450_0346 (
PULPROG zg30
TD 54274
SOLVENT CDCl3
NS 14
DS 0
SWH 8223.685 Hz
FIDRES 0.303043 Hz
AQ 3.2998593 sec
RG 161
DW 60.800 usec
DE 6.50 usec
TE 300.8 K
D1 1.00000000 sec
TD0 1
SFO1 400.1324710 MHz
NUC1 1H
P0 5.00 usec
P1 15.00 usec
PLW1 9.69999981 W

F2 - Processing parameters
SI 32768
SF 400.1300095 MHz
WDW EM
SSB 0
LB 0.30 Hz
GB 0
PC 1.00

12

SSK-34-AKM-514-13C

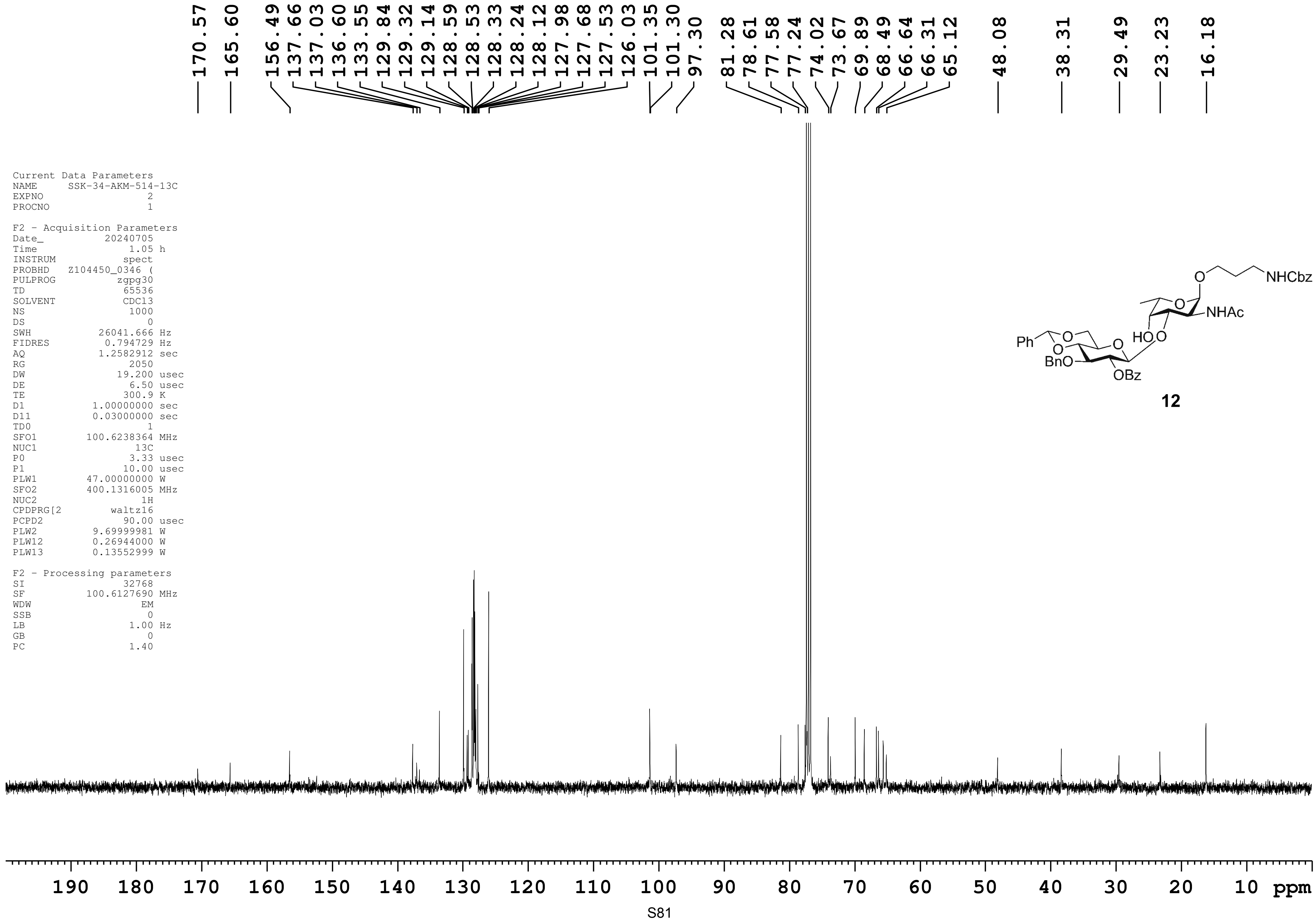

SSK-34-AKM-514-DEPT

```
Current Data Parameters
NAME     SSK-34-AKM-514-DEPT
EXPNO                      3
PROCNO                     1

F2 - Acquisition Parameters
Date_           20240705
Time                1.14 h
INSTRUM            spect
PROBHD   Z104450_0346 (
PULPROG          dept135
TD                 65536
SOLVENT            CDCl3
NS                   243
DS                     0
SWH            27777.777 Hz
FIDRES          0.847710 Hz
AQ             1.1796480 sec
RG                   203
DW                18.000 usec
DE                  6.50 usec
TE                 301.1 K
CNST2       145.0000000
D1           1.00000000 sec
D2           0.00344828 sec
D12          0.00002000 sec
TD0                    1
SFO1        100.6242389 MHz
NUC1                 13C
P1                 10.00 usec
P2                 20.00 usec
PLW1         47.00000000 W
SFO2        400.1316005 MHz
NUC2                  1H
CPDPRG[2         waltz16
P3                 15.00 usec
P4                 30.00 usec
PCPD2              90.00 usec
PLW2          9.69999981 W
PLW12         0.26944000 W

F2 - Processing parameters
SI                 32768
SF          100.6127690 MHz
WDW                   EM
SSB                    0
LB                  1.00 Hz
GB                     0
PC                  1.40
```

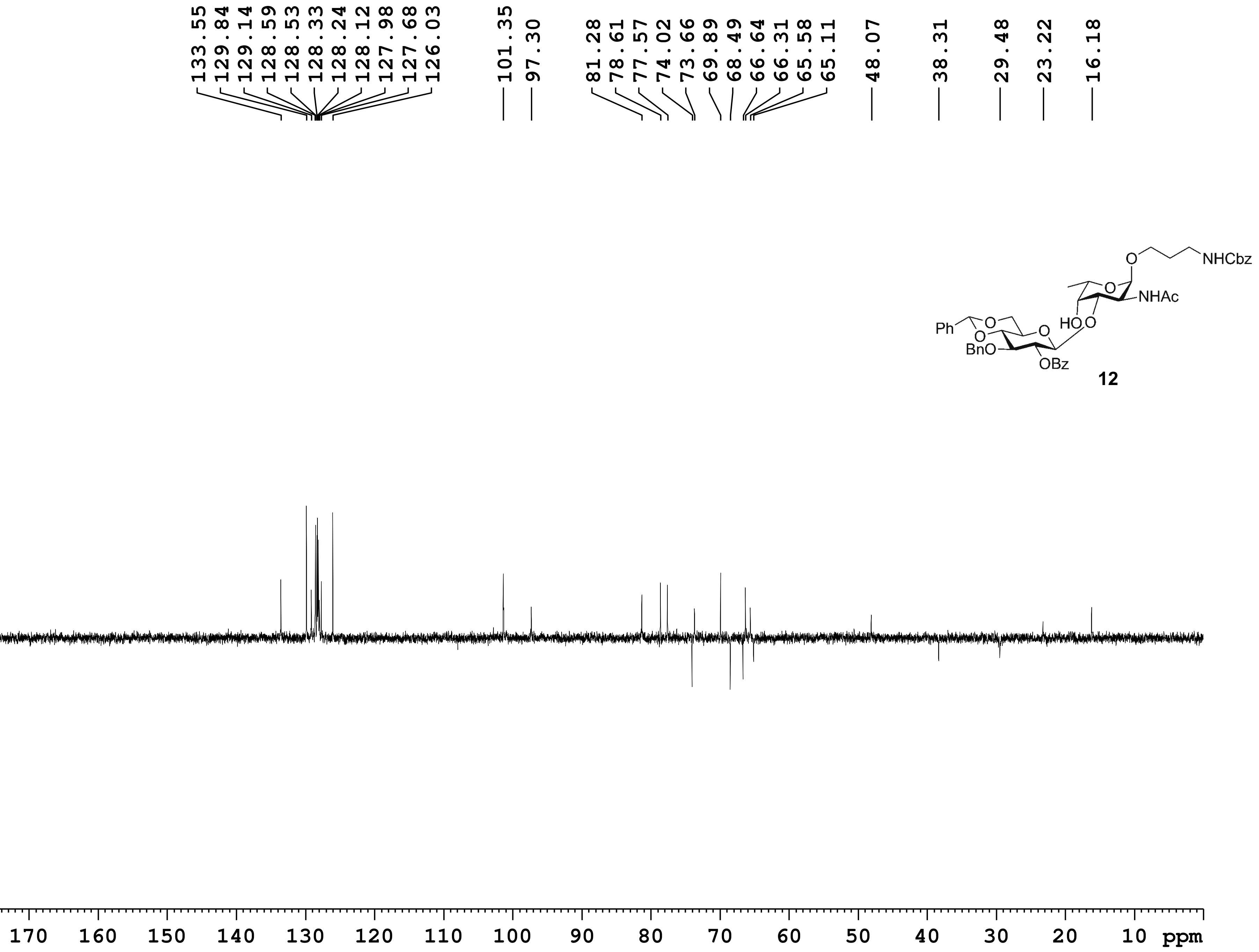

12

SSK-34-AKM-514-COSY

```
Current Data Parameters
NAME      SSK-34-AKM-514-COSY
EXPNO                       4
PROCNO                      1

F2 - Acquisition Parameters
Date_              20240705
Time                   1.27 h
INSTRUM               spect
PROBHD    Z104450_0346 (
PULPROG            cosygpqf
TD                     2048
SOLVENT               CDCl3
NS                        4
DS                        0
SWH                2503.338 Hz
FIDRES             2.444666 Hz
AQ                0.4090539 sec
RG                       64
DW                  199.733 usec
DE                     6.50 usec
TE                    300.8 K
D0               0.00000300 sec
D1               1.00000000 sec
D13              0.00000400 sec
D16              0.00020000 sec
IN0              0.00039940 sec
TDav                      1
SFO1         400.1313151 MHz
NUC1                     1H
P0                    15.00 usec
P1                    15.00 usec
PLW1             9.69999981 W
GPNAM[1]           SINE.100
GPZ1                  10.00 %
P16                 1000.00 usec

F1 - Acquisition parameters
TD                      128
SFO1            400.1313 MHz
FIDRES            39.121181 Hz
SW                    6.257 ppm
FnMODE                   QF

F2 - Processing parameters
SI                     1024
SF              400.1300000 MHz
WDW                    SINE
SSB                       0
LB                        0 Hz
GB                        0
PC                     1.40

F1 - Processing parameters
SI                     1024
MC2                      QF
SF              400.1300000 MHz
WDW                    SINE
SSB                       0
LB                        0 Hz
GB                        0
```

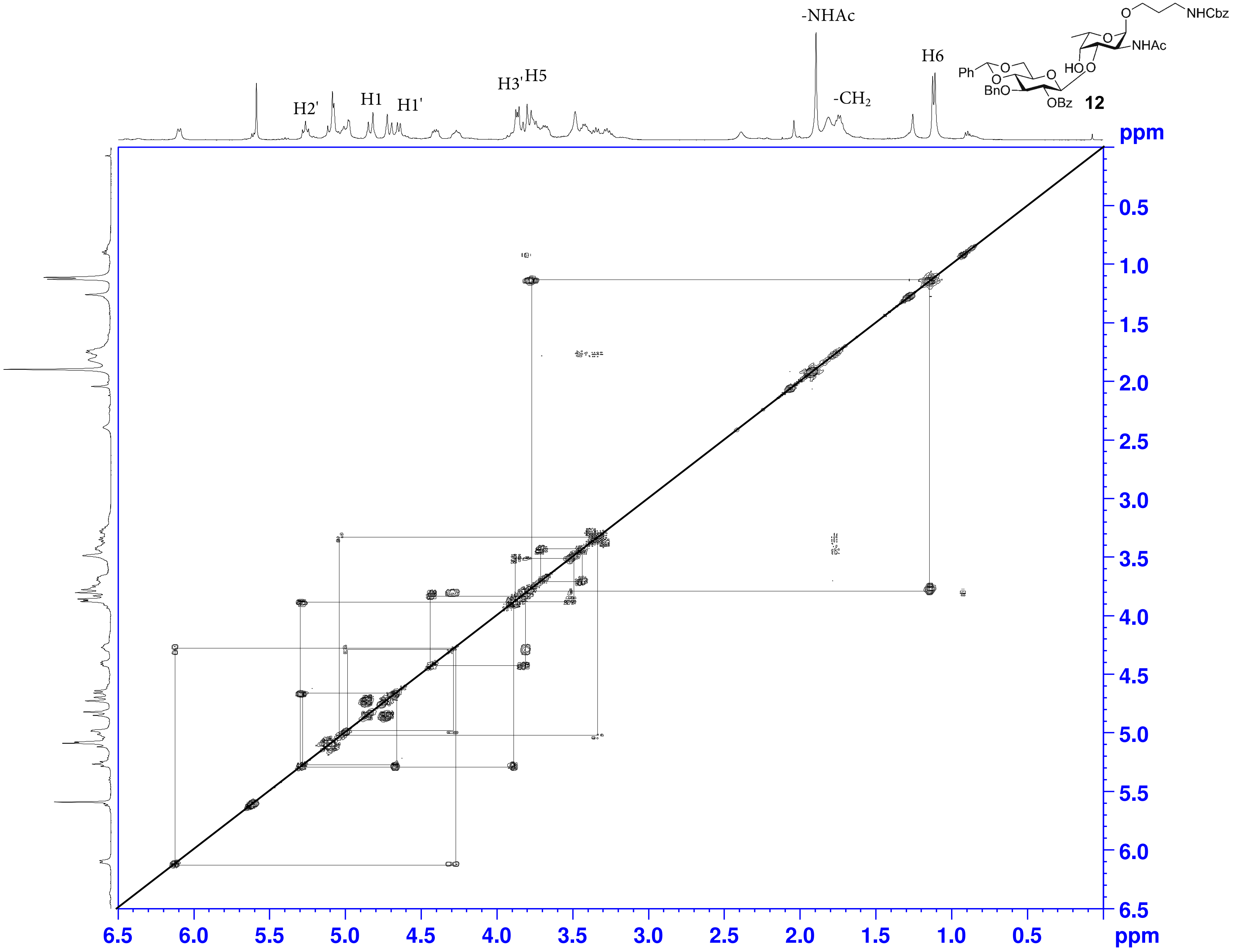

SSK-34-AKM-514-HSQC

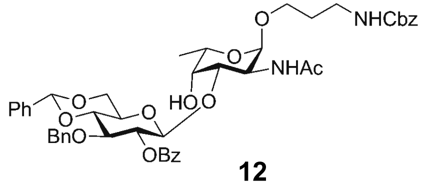

12

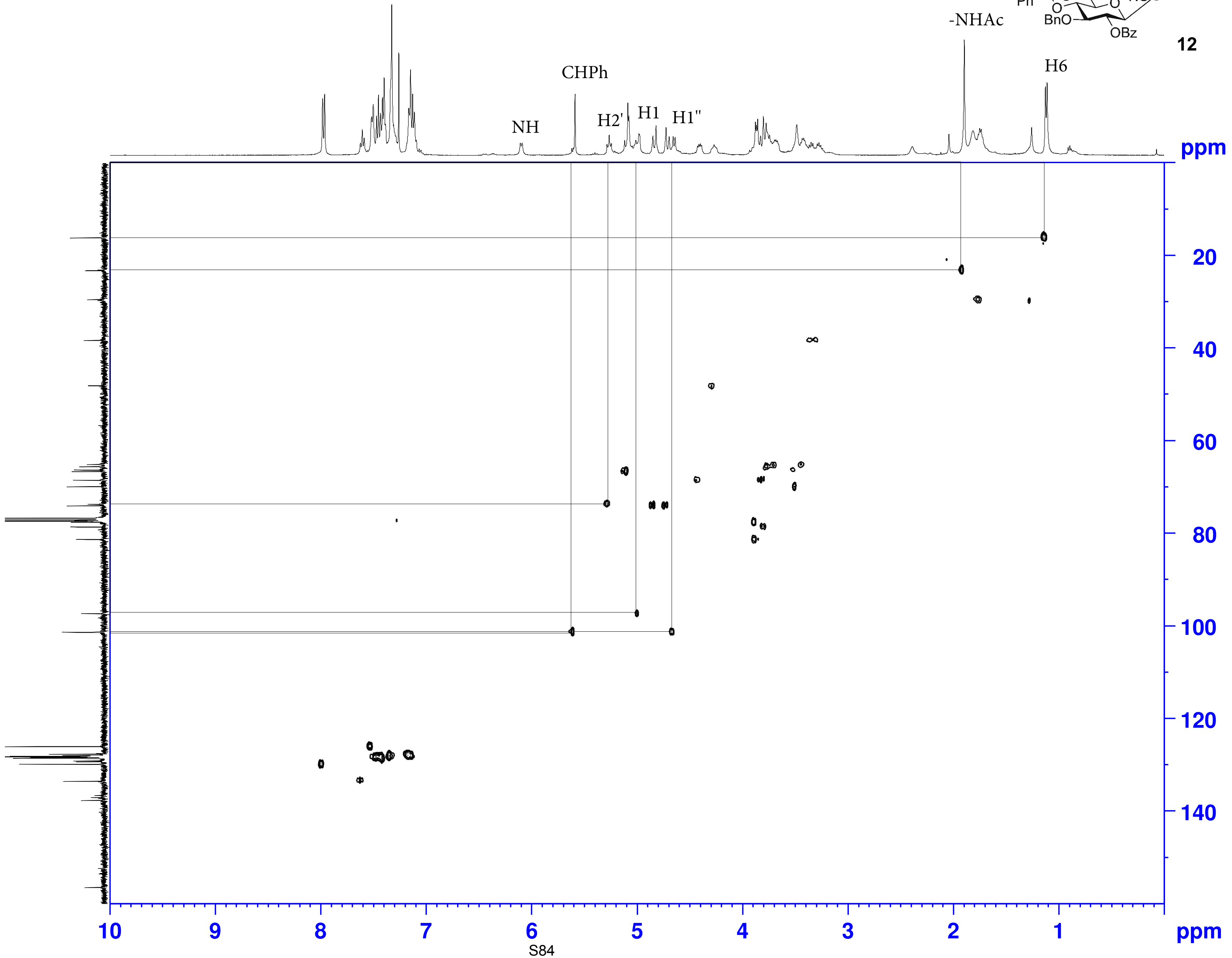


```
Current Data Parameters
NAME      SSK-34-AKM-514-HSQC
EXPNO                     5
PROCNO                    1

F2 - Acquisition Parameters
Date_              20240705
Time                  1.34 h
INSTRUM               spect
PROBHD    Z104450_0346 (
PULPROG            hsqcetgp
TD                     2048
SOLVENT               CDCl3
NS                        2
DS                        0
SWH                4032.258 Hz
FIDRES             3.937752 Hz
AQ                0.2539520 sec
RG                     2050
DW                  124.000 usec
DE                     6.50 usec
TE                    300.3 K
CNST2           145.0000000
D0               0.00000300 sec
D1               1.00000000 sec
D4               0.00172414 sec
D11              0.03000000 sec
D16              0.00020000 sec
IN0              0.00002760 sec
TDav                      1
ZGOPTNS
SFO1            400.1320070 MHz
NUC1                     1H
P1                    15.00 usec
P2                    30.00 usec
PLW1             9.69999981 W
SFO2            100.6218761 MHz
NUC2                    13C
CPDPRG[2               garp
P3                    10.00 usec
P4                    20.00 usec
PCPD2                 80.00 usec
PLW2            47.00000000 W
PLW12            0.73438001 W
GPNAM[1]           SINE.100
GPZ1                  80.00 %
GPNAM[2]           SINE.100
GPZ2                  20.10 %
P16                 1000.00 usec

F1 - Acquisition parameters
TD                      171
SFO1               100.6219 MHz
FIDRES           211.882370 Hz
SW                  180.040 ppm
FnMODE       Echo-Antiecho

F2 - Processing parameters
SI                     2048
SF              400.1300000 MHz
WDW                   QSINE
SSB                       2
LB                        0 Hz
GB                        0
PC                     1.40

F1 - Processing parameters
SI                     1024
MC2          echo-antiecho
SF              100.6127690 MHz
WDW                   QSINE
SSB                       2
LB                        0 Hz
GB                        0
```

SSK-34-AKM-516-1H

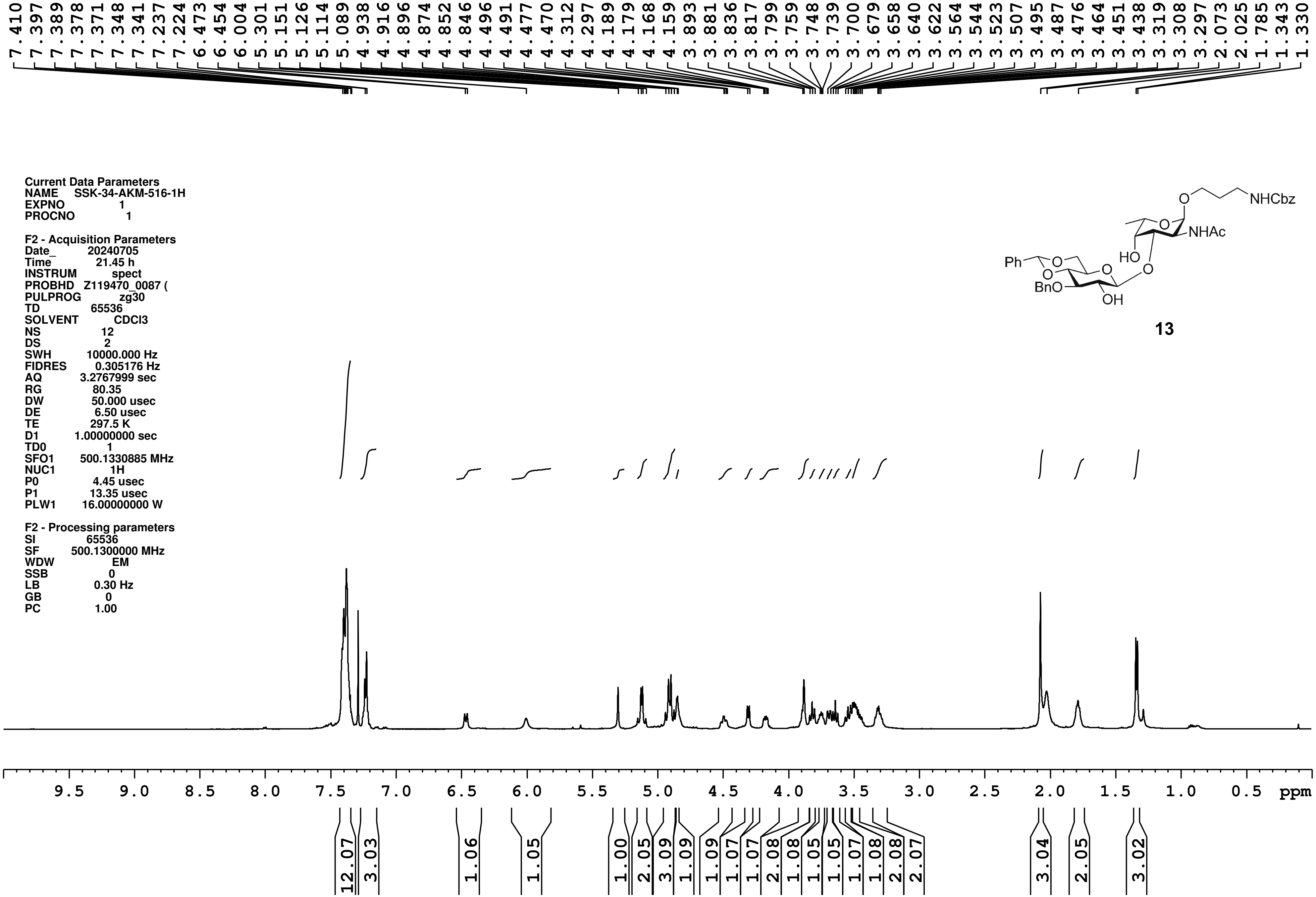

13

SSK-34-AKM-516-13C

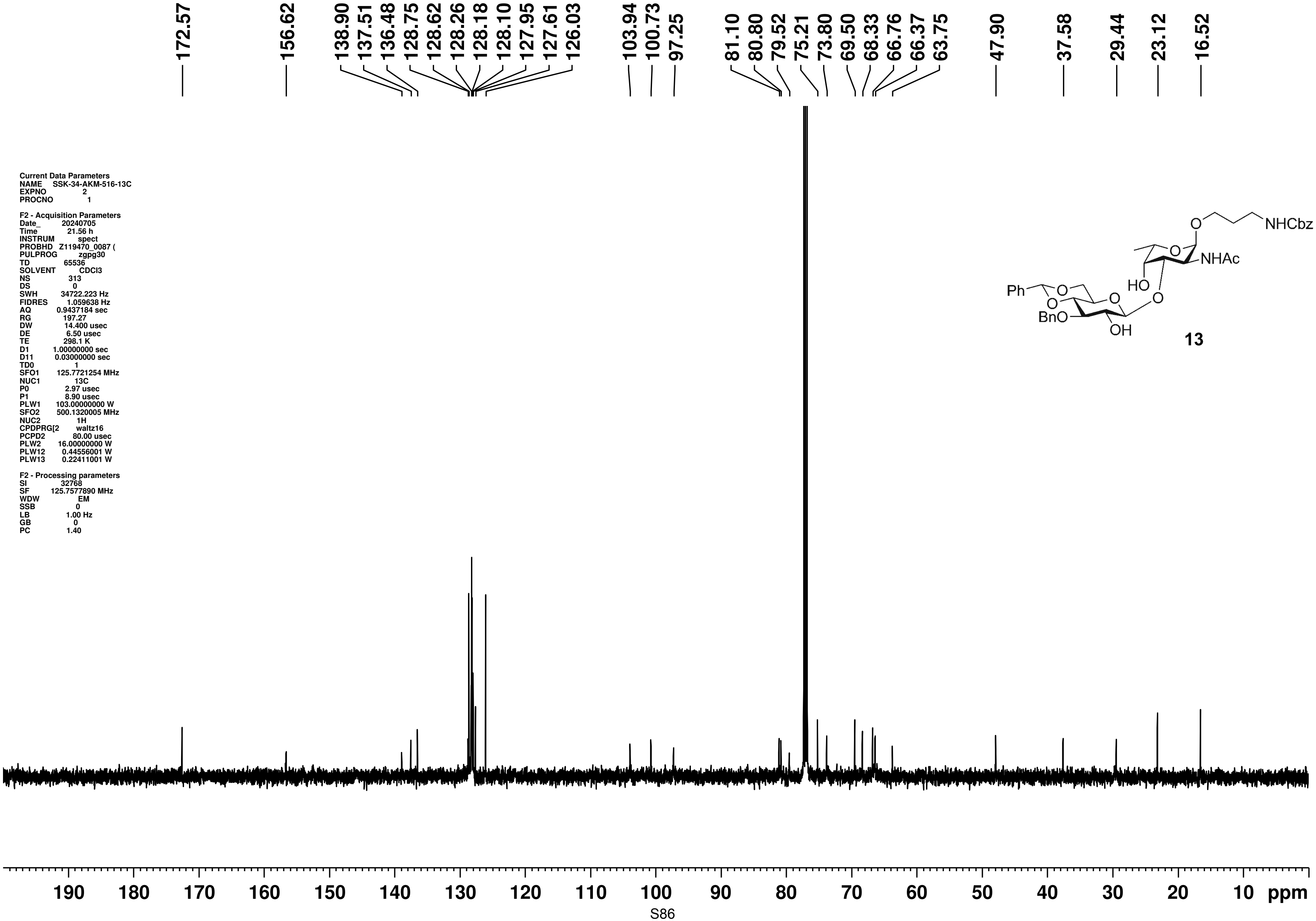

Current Data Parameters
NAME SSK-34-AKM-516-13C
EXPNO 2
PROCNO 1

F2 - Acquisition Parameters
Date_ 20240705
Time 21.56 h
INSTRUM spect
PROBHD Z119470_0087 (
PULPROG zgpg30
TD 65536
SOLVENT CDCl3
NS 313
DS 0
SWH 34722.223 Hz
FIDRES 1.059638 Hz
AQ 0.9437184 sec
RG 197.27
DW 14.400 usec
DE 6.50 usec
TE 298.1 K
D1 1.00000000 sec
D11 0.03000000 sec
TD0 1
SFO1 125.7721254 MHz
NUC1 13C
P0 2.97 usec
P1 8.90 usec
PLW1 103.00000000 W
SFO2 500.1320005 MHz
NUC2 1H
CPDPRG[2 waltz16
PCPD2 80.00 usec
PLW2 16.00000000 W
PLW12 0.44556001 W
PLW13 0.22411001 W

F2 - Processing parameters
SI 32768
SF 125.7577890 MHz
WDW EM
SSB 0
LB 1.00 Hz
GB 0
PC 1.40

SSK-34-AKM-516-DEPT

```
Current Data Parameters
NAME      SSK-34-AKM-516-DEPT
EXPNO                   1
PROCNO                  1

F2 - Acquisition Parameters
Date_            20240705
Time                22.26 h
INSTRUM             spect
PROBHD   Z104450_0346 (
PULPROG           dept135
TD                  65536
SOLVENT             CDCl3
NS                    271
DS                      0
SWH             27777.777 Hz
FIDRES           0.847710 Hz
AQ              1.1796480 sec
RG                   2050
DW                 18.000 usec
DE                   6.50 usec
TE                 3089.1 K
CNST2         145.0000000
D1             1.00000000 sec
D2             0.00344828 sec
D12            0.00002000 sec
TD0                     1
SFO1          100.6242389 MHz
NUC1                  13C
P1                  10.00 usec
P2                  20.00 usec
PLW1          47.00000000 W
SFO2          400.1316005 MHz
NUC2                   1H
CPDPRG[2          waltz16
P3                  15.00 usec
P4                  30.00 usec
PCPD2               90.00 usec
PLW2           9.69999981 W
PLW12          0.26944000 W

F2 - Processing parameters
SI                  32768
SF            100.6127690 MHz
WDW                    EM
SSB                     0
LB                   1.00 Hz
GB                      0
PC                   1.40
```

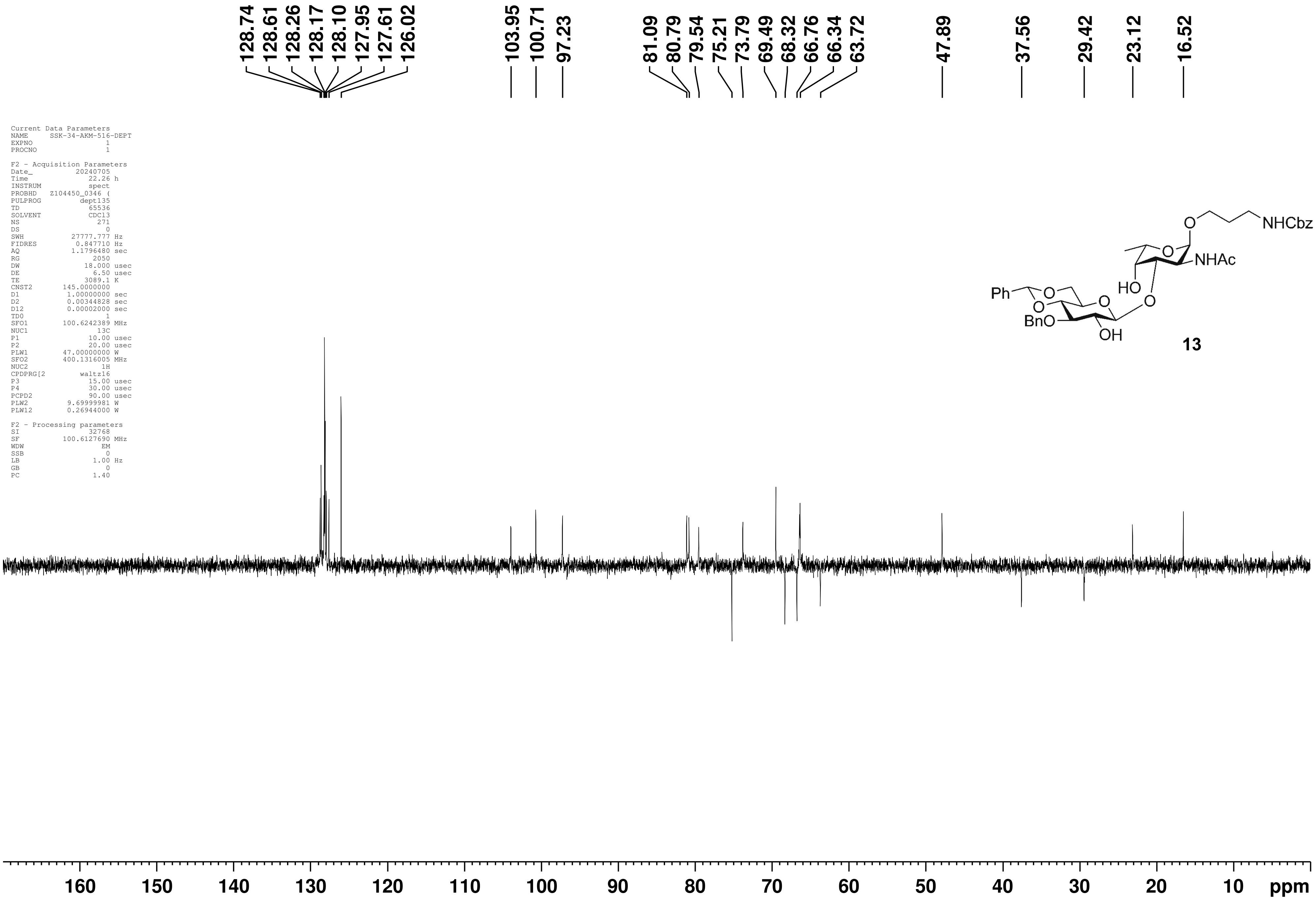

13

SSK-34-AKM-516-COSY

```
Current Data Parameters
NAME      SSK-34-AKM-516-COSY
EXPNO                     2
PROCNO                    1

F2 - Acquisition Parameters
Date_              20240705
Time                  22.33 h
INSTRUM               spect
PROBHD    Z104450_0346 (
PULPROG            cosygpqf
TD                     2048
SOLVENT               CDCl3
NS                        2
DS                        0
SWH                2802.691 Hz
FIDRES             2.737003 Hz
AQ                0.3653632 sec
RG                      456
DW                  178.400 usec
DE                     6.50 usec
TE                   3080.4 K
D0               0.00000300 sec
D1               1.00000000 sec
D13              0.00000400 sec
D16              0.00020000 sec
IN0              0.00035680 sec
TDav                      1
SFO1            400.1313950 MHz
NUC1                     1H
P0                    15.00 usec
P1                    15.00 usec
PLW1             9.69999981 W
GPNAM[1]           SINE.100
GPZ1                  10.00 %
P16                 1000.00 usec

F1 - Acquisition parameters
TD                      103
SFO1               400.1314 MHz
FIDRES            54.421177 Hz
SW                    7.004 ppm
FnMODE                   QF

F2 - Processing parameters
SI                     1024
SF              400.1300000 MHz
WDW                    SINE
SSB                       0
LB                        0 Hz
GB                        0
PC                     1.40

F1 - Processing parameters
SI                     1024
MC2                      QF
SF              400.1300000 MHz
WDW                    SINE
SSB                       0
LB                        0 Hz
GB                        0
```

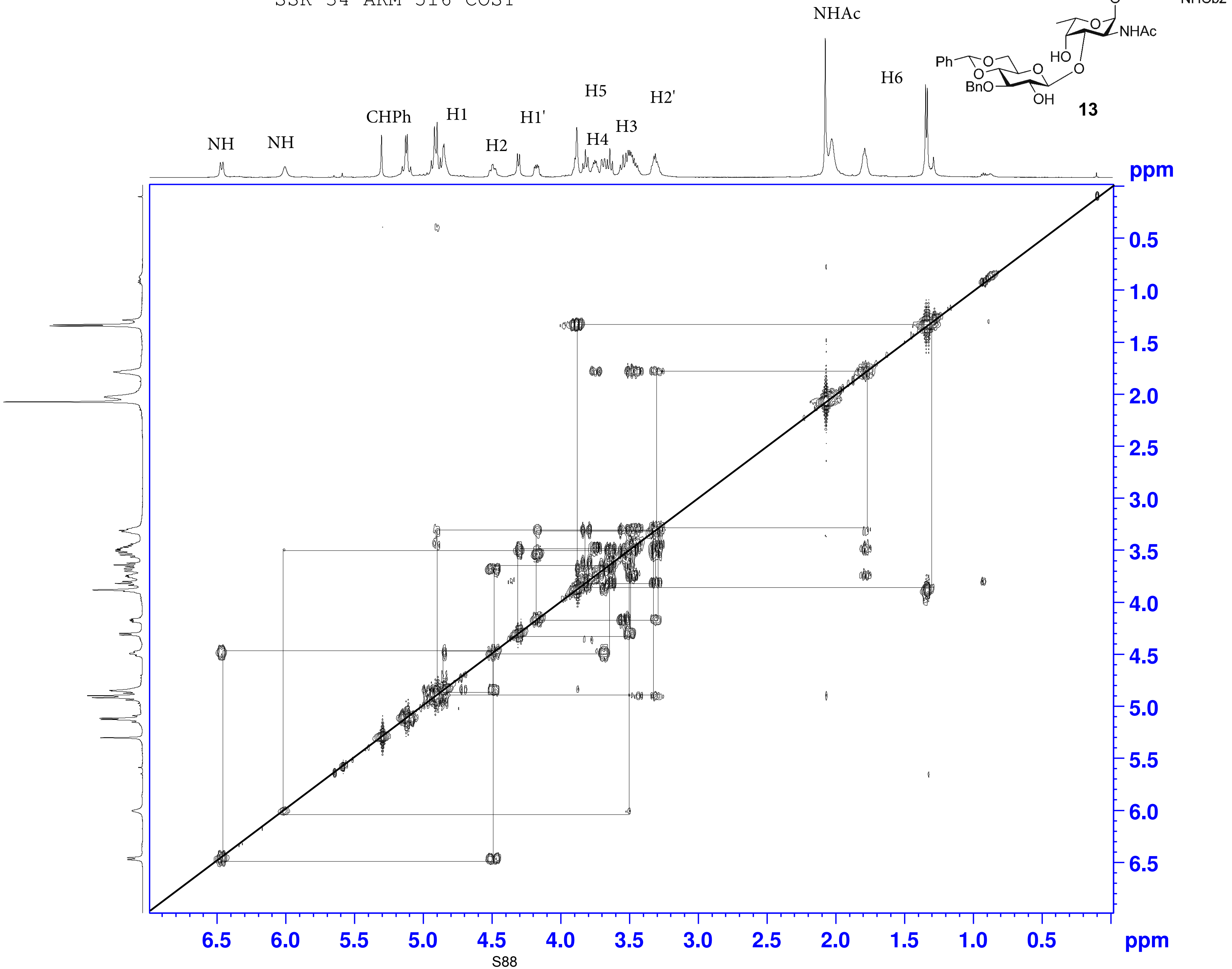

SSK-34-AKM-516-HSQC

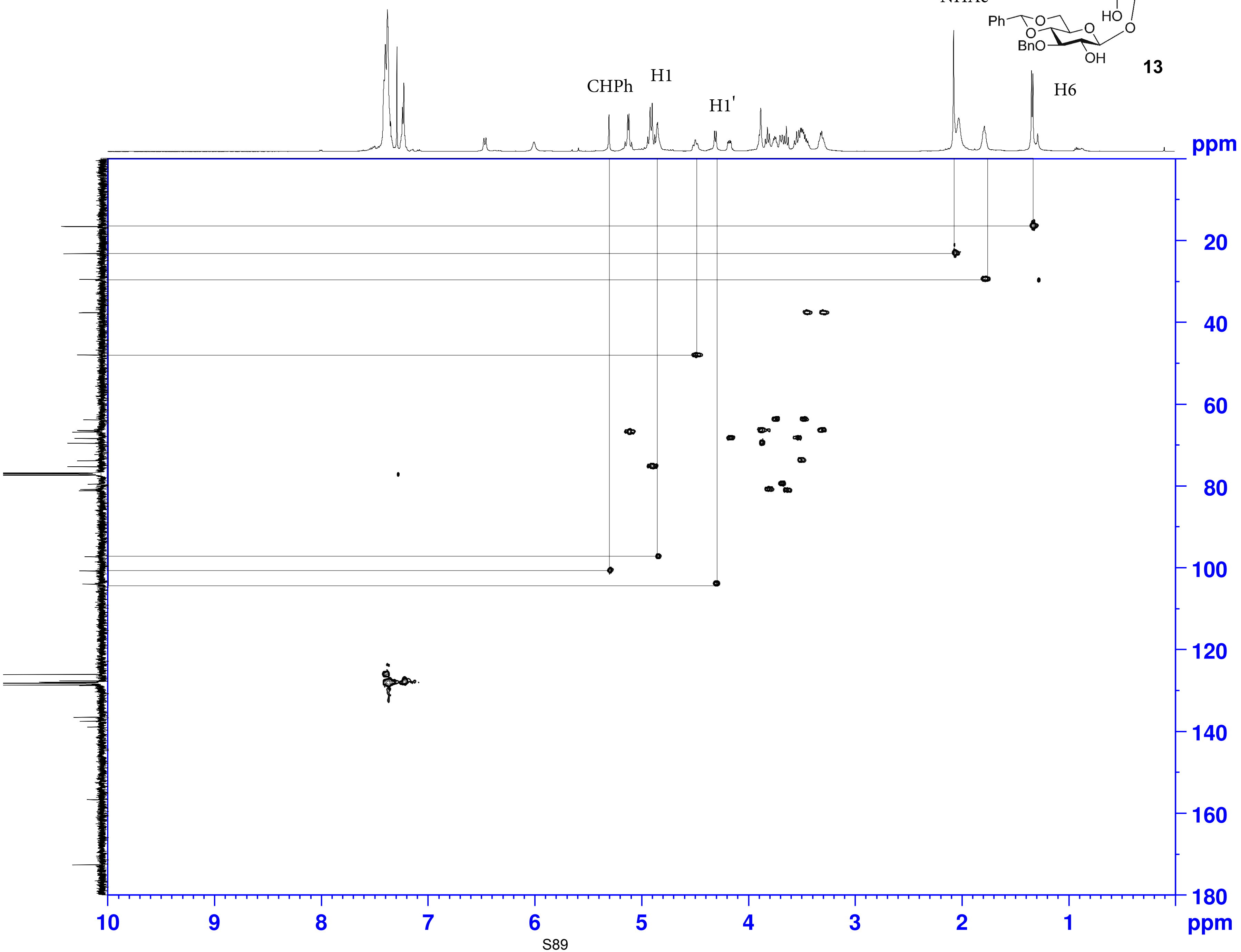

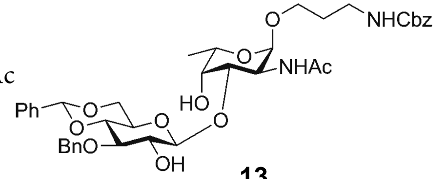

13

```
Current Data Parameters
NAME     SSK-34-AKM-516-HSQC
EXPNO                      3
PROCNO                     1

F2 - Acquisition Parameters
Date_               20240705
Time                   22.46 h
INSTRUM                spect
PROBHD      Z104450_0346 (
PULPROG             hsqcetgp
TD                      2048
SOLVENT                CDCl3
NS                         2
DS                         0
SWH                 4032.258 Hz
FIDRES              3.937752 Hz
AQ                 0.2539520 sec
RG                      2050
DW                   124.000 usec
DE                      6.50 usec
TE                    3110.8 K
CNST2            145.0000000
D0                0.00000300 sec
D1                1.00000000 sec
D4                0.00172414 sec
D11               0.03000000 sec
D16               0.00020000 sec
IN0               0.00002700 sec
TDav                       1
ZGOPTNS
SFO1             400.1319959 MHz
NUC1                      1H
P1                     15.00 usec
P2                     30.00 usec
PLW1              9.69999981 W
SFO2             100.6219346 MHz
NUC2                     13C
CPDPRG[2                garp
P3                     10.00 usec
P4                     20.00 usec
PCPD2                  80.00 usec
PLW2             47.00000000 W
PLW12             0.73438001 W
GPNAM[1]            SINE.100
GPZ1                   80.00 %
GPNAM[2]            SINE.100
GPZ2                   20.10 %
P16                  1000.00 usec

F1 - Acquisition parameters
TD                       256
SFO1                100.6219 MHz
FIDRES            144.675919 Hz
SW                   184.041 ppm
FnMODE        Echo-Antiecho

F2 - Processing parameters
SI                      2048
SF               400.1300000 MHz
WDW                    QSINE
SSB                        2
LB                         0 Hz
GB                         0
PC                      1.40

F1 - Processing parameters
SI                      1024
MC2           echo-antiecho
SF               100.6127690 MHz
WDW                    QSINE
SSB                        2
LB                         0 Hz
GB                         0
```

SSK-34-AKM-G4

Current Data Parameters
NAME SSK-34-AKM-G4
EXPNO 1
PROCNO 1

F2 - Acquisition Parameters
Date_ 20251126
Time 13.25
INSTRUM Avance Neo 600
PROBHD Z154705_0128 (
PULPROG zg
TD 28846
SOLVENT D2O
NS 50
DS 0
SWH 9615.385
FIDRES 0.666670
AQ 1.4999920
RG 36
DW 52.000
DE 6.63 usec
TE 299.2 K

F2 - Processing parameters
SI 65536
SF 600.3700000 MHz
WDW EM
SSB 0
LB 0.30 Hz
GB 0
PC 1.00

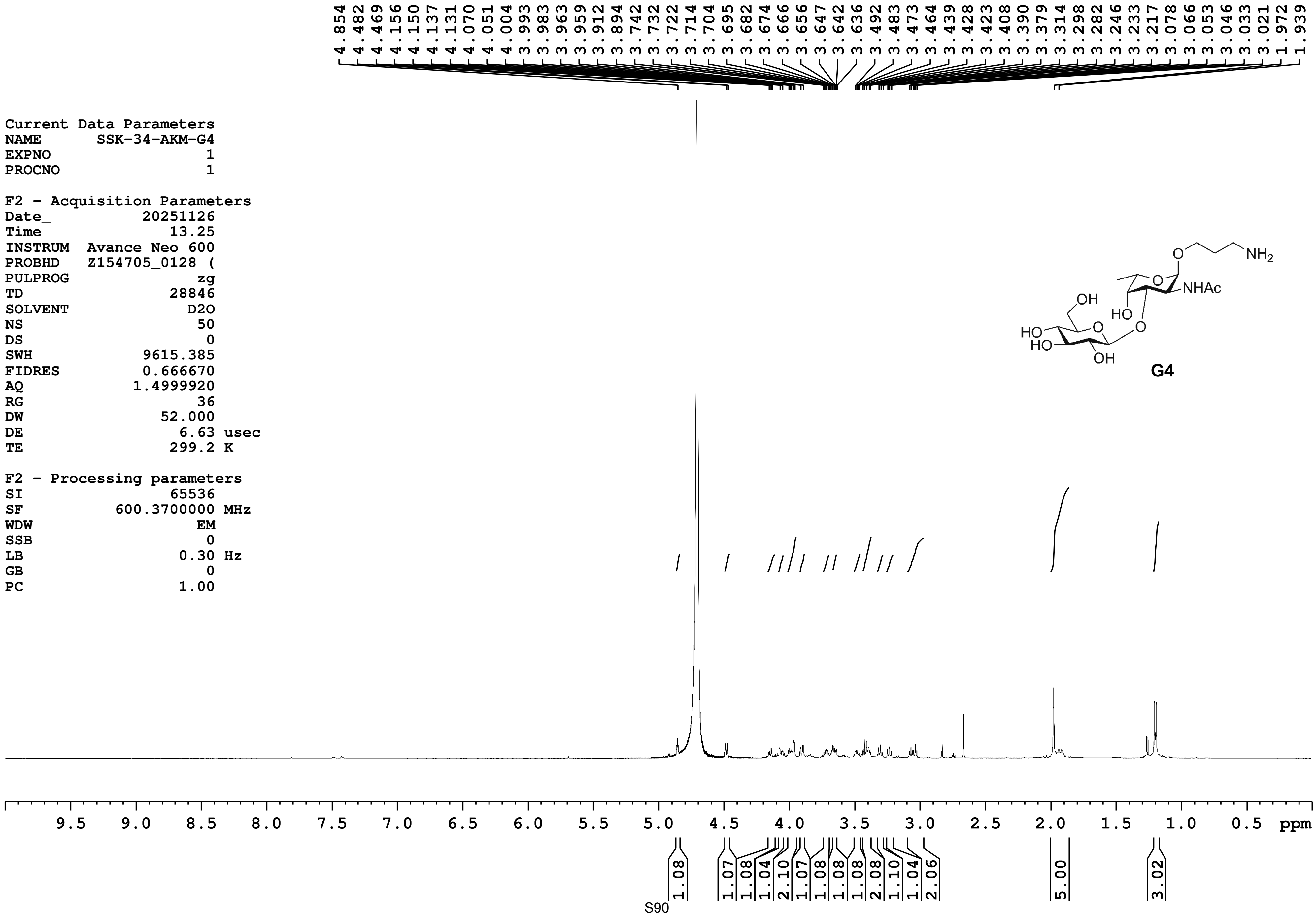

G4

SSK-34-AKM-517-8-13C

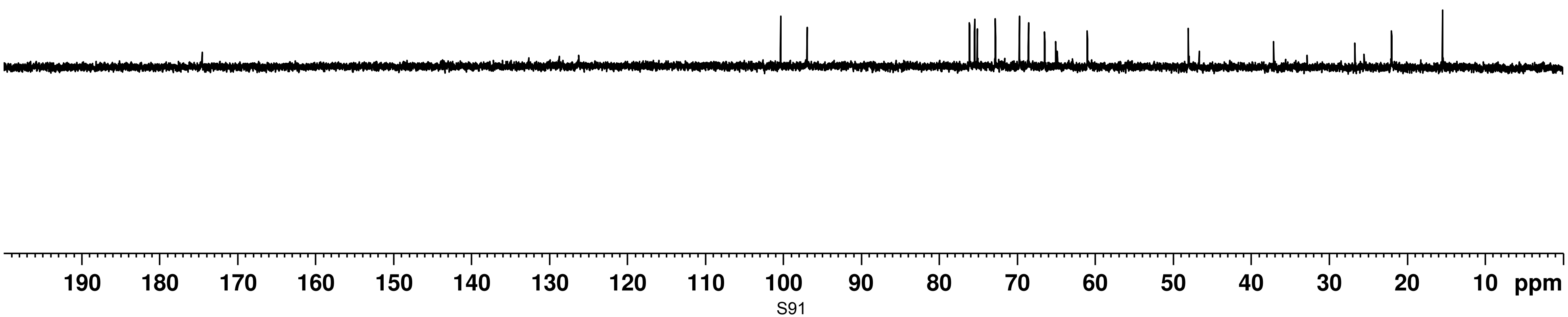

Current Data Parameters
NAME SSK-34-AKM-517-8-13C
EXPNO 3
PROCNO 1

F2 - Acquisition Parameters
Date_ 20240714
Time 22.20 h
INSTRUM spect
PROBHD Z119470_0087 (
PULPROG zgpg30
TD 65536
SOLVENT D2O
NS 15387
DS 0
SWH 34722.223 Hz
FIDRES 1.059638 Hz
AQ 0.9437184 sec
RG 197.27
DW 14.400 usec
DE 6.50 usec
TE 297.1 K
D1 1.00000000 sec
D11 0.03000000 sec
TD0 1
SFO1 125.7721254 MHz
NUC1 13C
P0 2.97 usec
P1 8.90 usec
PLW1 103.00000000 W
SFO2 500.1320005 MHz
NUC2 1H
CPDPRG[2 waltz16
PCPD2 80.00 usec
PLW2 16.00000000 W
PLW12 0.44556001 W
PLW13 0.22411001 W

F2 - Processing parameters
SI 32768
SF 125.7577890 MHz
WDW EM
SSB 0
LB 1.00 Hz
GB 0
PC 0.30

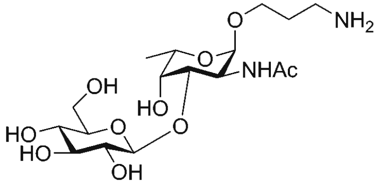

G4

SSK-34-AKM-517-8-DEPT

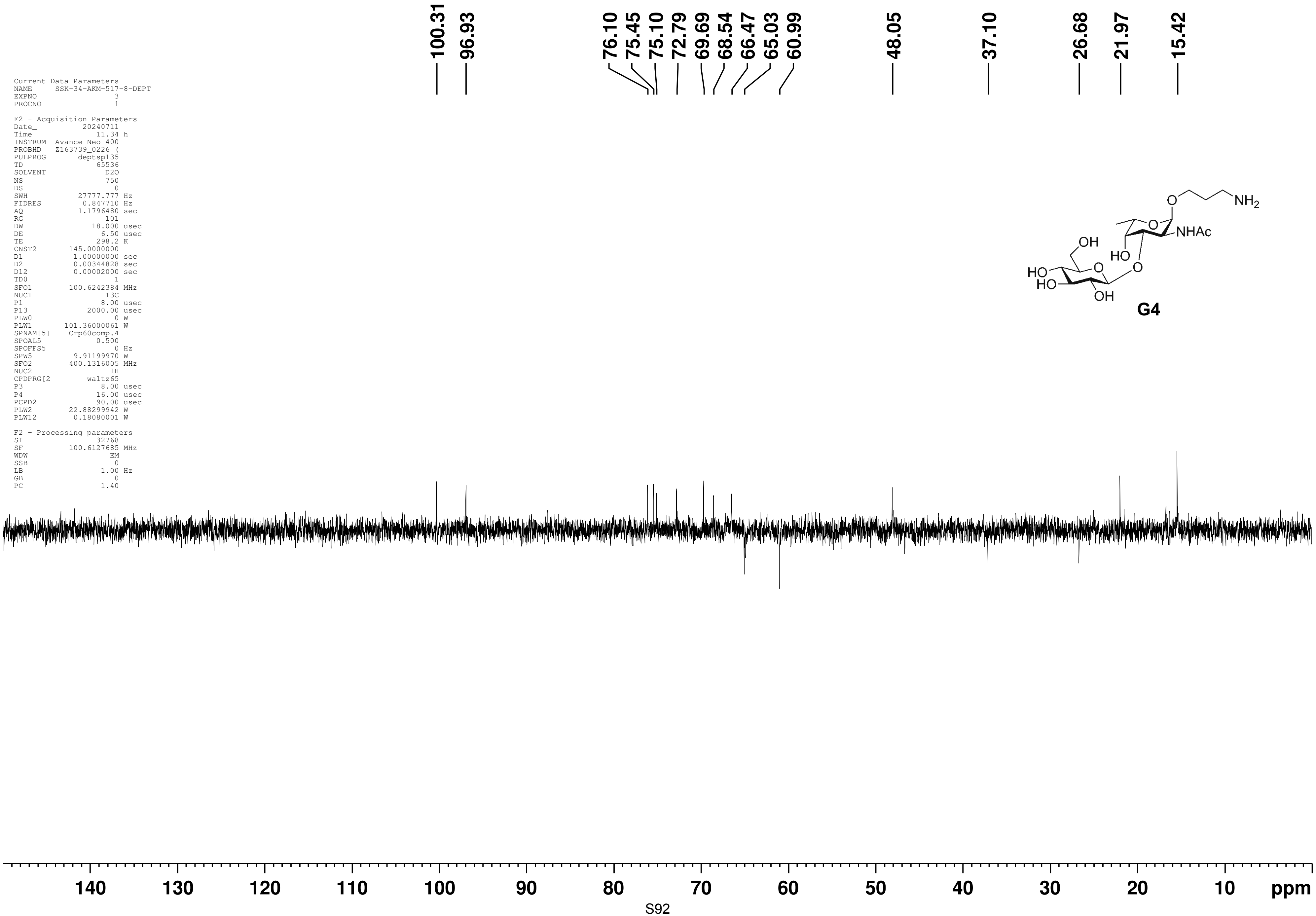

SSK-34-AKM-517-8-GL-HSQC

```
Current Data Parameters
NAME     SSK-34-AKM-517-8-GL-HSQC
EXPNO                   3
PROCNO                  1

F2 - Acquisition Parameters
Date_          20240710
Time              21.39 h
INSTRUM          Avance
PROBHD   Z163739_0237 (
PULPROG  hsqcedetgpsisp2.3
TD                 2048
SOLVENT             D2O
NS                    4
DS                    0
SWH            8196.721 Hz
FIDRES         8.004611 Hz
AQ            0.1249280 sec
RG                  101
DW               61.000 usec
DE                 6.50 usec
TE                297.2 K
CNST2       145.0000000
CNST17       -0.5000000
D0           0.00000300 sec
D1           1.00000000 sec
D4           0.00172414 sec
D11          0.03000000 sec
D16          0.00020000 sec
D21          0.00344800 sec
D24          0.00086200 sec
IN0          0.00001799 sec
TDav                  1
ZGOPTNS
SFO1        400.3024719 MHz
NUC1                 1H
P1                 8.00 usec
P2                16.00 usec
PLW1        21.00099945 W
SFO2        100.6669898 MHz
NUC2                13C
CPDPRG[2          garp4
P3                 8.00 usec
P14              500.00 usec
P24             2000.00 usec
P31             2119.00 usec
PCPD2             80.00 usec
PLW0                  0 W
PLW2        97.90799713 W
PLW12        0.97908002 W
SPNAM[3] Crp60,0.5,20.1
SPOAL3            0.500
SPOFFS3               0 Hz
SPW3         9.57390022 W
SPNAM[7]    Crp60comp.4
SPOAL7            0.500
SPOFFS7               0 Hz
SPW7         9.57390022 W
SPNAM[18  Crp60_xfilt.2
SPOAL18           0.500
SPOFFS18              0 Hz
SPW18        1.84440005 W
GPNAM[1]     SMSQ10.100
GPZ1              80.00 %
GPNAM[2]     SMSQ10.100
GPZ2              20.10 %
GPNAM[3]     SMSQ10.100
GPZ3              11.00 %
GPNAM[4]     SMSQ10.100
GPZ4              -5.00 %
P16             1000.00 usec
P19              600.00 usec

======== F1 INDIRECT DIMENSION ========
td1      1024
sw_F1    276.000000

F1 - Acquisition parameters
TD                  480
SFO1            100.667 MHz
FIDRES       115.767036 Hz
SW              276.000 ppm
FnMODE    Echo-Antiecho

F2 - Processing parameters
SI                 1024
SF          400.3000000 MHz
WDW               QSINE
SSB                   2
LB                    0 Hz
GB                    0
PC                 1.40

F1 - Processing parameters
SI                 1024
MC2        echo-antiecho
SF          100.6555151 MHz
WDW               QSINE
SSB                   2
LB                    0 Hz
GB                    0
```

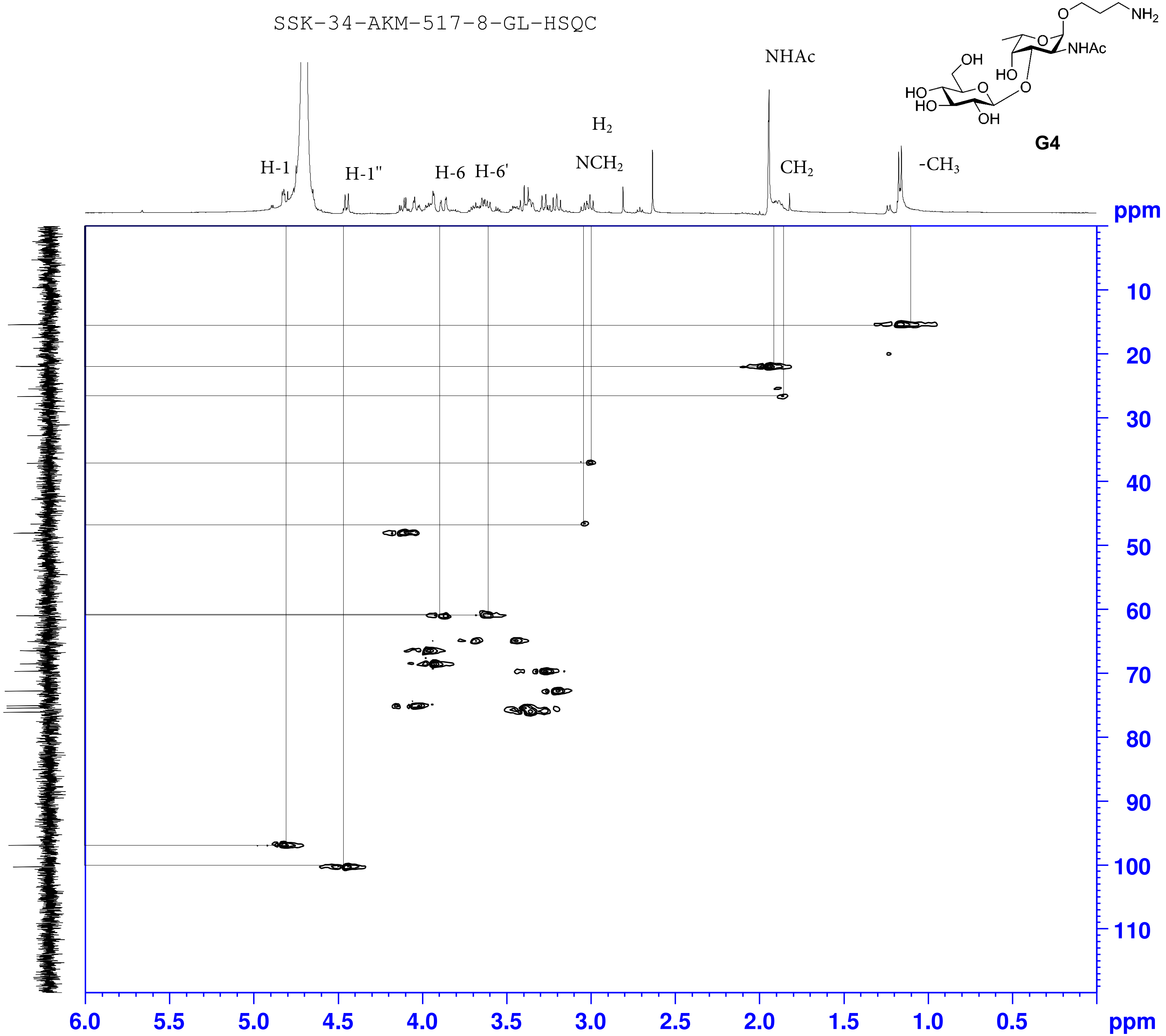

SSK-34-AKM-480-DS-1H

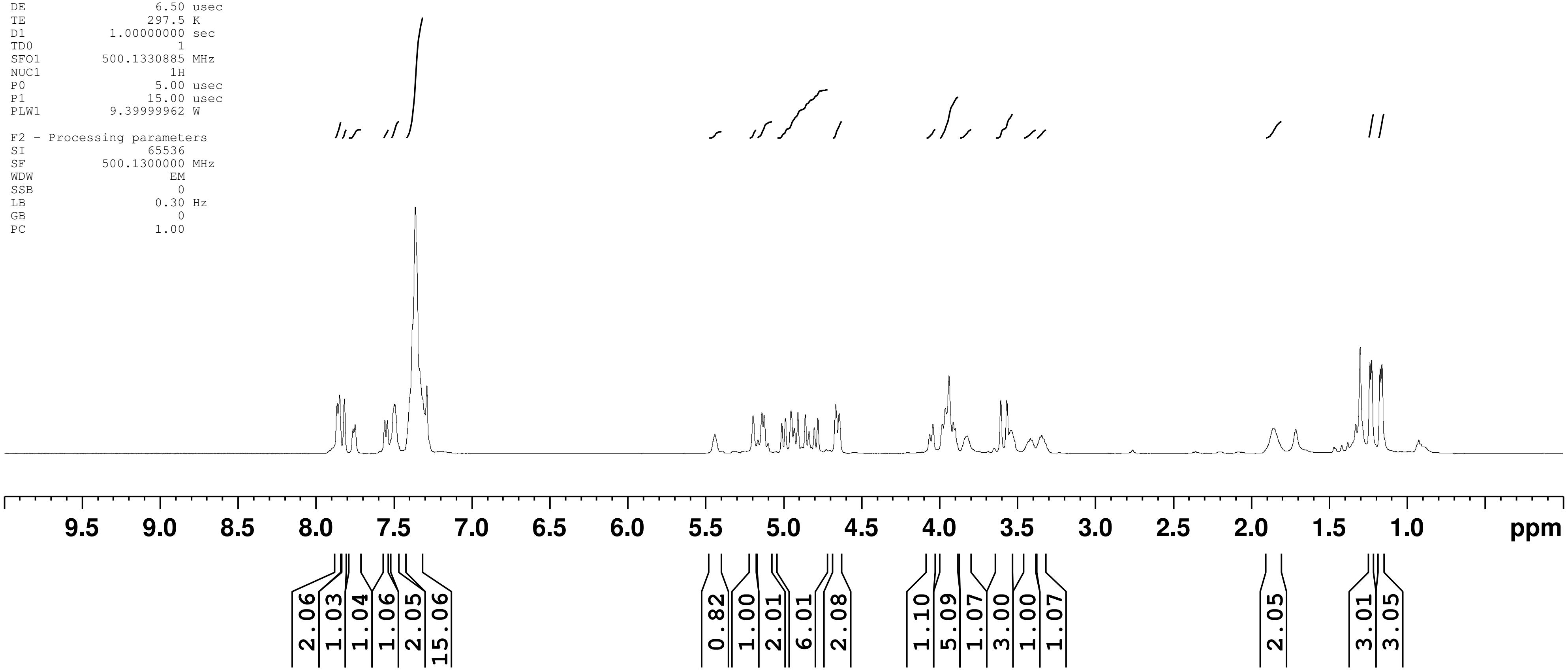

Current Data Parameters
NAME SSK-34-AKM-480-DS-1H
EXPNO 1
PROCNO 1

F2 - Acquisition Parameters
Date_ 20240420
Time 15.44 h
INSTRUM spect
PROBHD Z124627_0008 (
PULPROG zg30
TD 65536
SOLVENT CDCl3
NS 16
DS 0
SWH 10000.000 Hz
FIDRES 0.305176 Hz
AQ 3.2767999 sec
RG 106.54
DW 50.000 usec
DE 6.50 usec
TE 297.5 K
D1 1.00000000 sec
TD0 1
SFO1 500.1330885 MHz
NUC1 1H
P0 5.00 usec
P1 15.00 usec
PLW1 9.39999962 W

F2 - Processing parameters
SI 65536
SF 500.1300000 MHz
WDW EM
SSB 0
LB 0.30 Hz
GB 0
PC 1.00

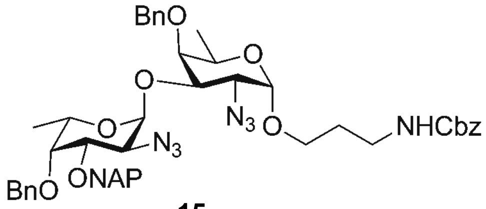

15

SSK-34-AKM-480-DS-13C

```
Current Data Parameters
NAME     SSK-34-AKM-480-DS-13C
EXPNO                    2
PROCNO                   1

F2 - Acquisition Parameters
Date_           20240420
Time               15.53 h
INSTRUM            spect
PROBHD   Z124627_0008 (
PULPROG          zgpg30
TD                65536
SOLVENT           CDCl3
NS                  254
DS                    0
SWH           34722.223 Hz
FIDRES         1.059638 Hz
AQ            0.9437184 sec
RG               197.27
DW               14.400 usec
DE                 6.50 usec
TE                297.6 K
D1           1.00000000 sec
D11          0.03000000 sec
TD0                   1
SFO1        125.7721254 MHz
NUC1                13C
P0                 3.33 usec
P1                10.00 usec
PLW1        50.00000000 W
SFO2        500.1320005 MHz
NUC2                 1H
CPDPRG[2        waltz16
PCPD2             80.00 usec
PLW2         9.39999962 W
PLW12        0.33047000 W
PLW13        0.16621999 W

F2 - Processing parameters
SI                32768
SF          125.7577890 MHz
WDW                  EM
SSB                   0
LB                 1.00 Hz
GB                    0
PC                 1.40
```

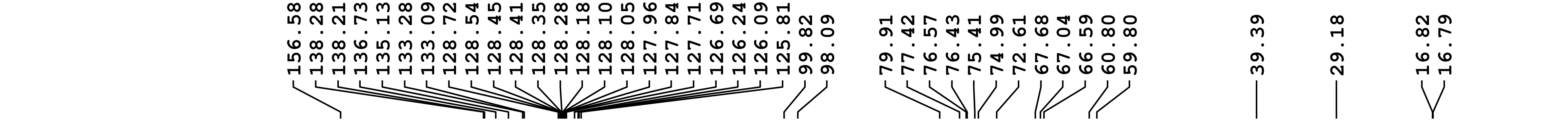

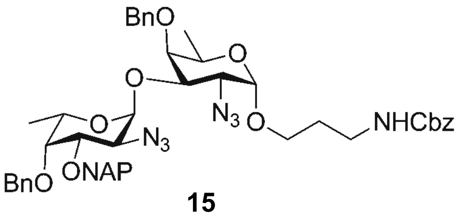

15

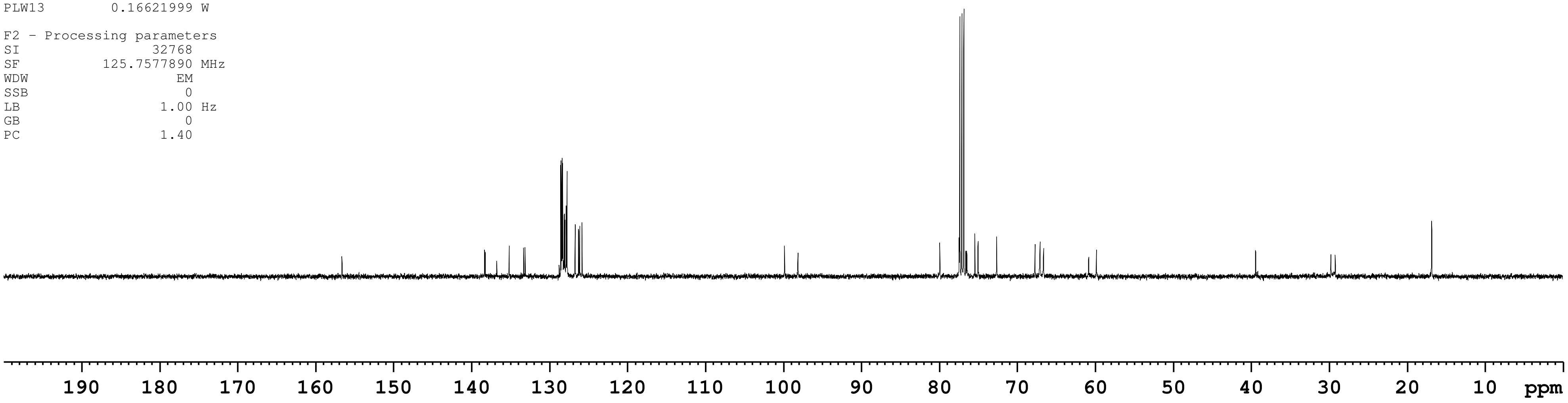

SSK-34-AKM-480-DEPT

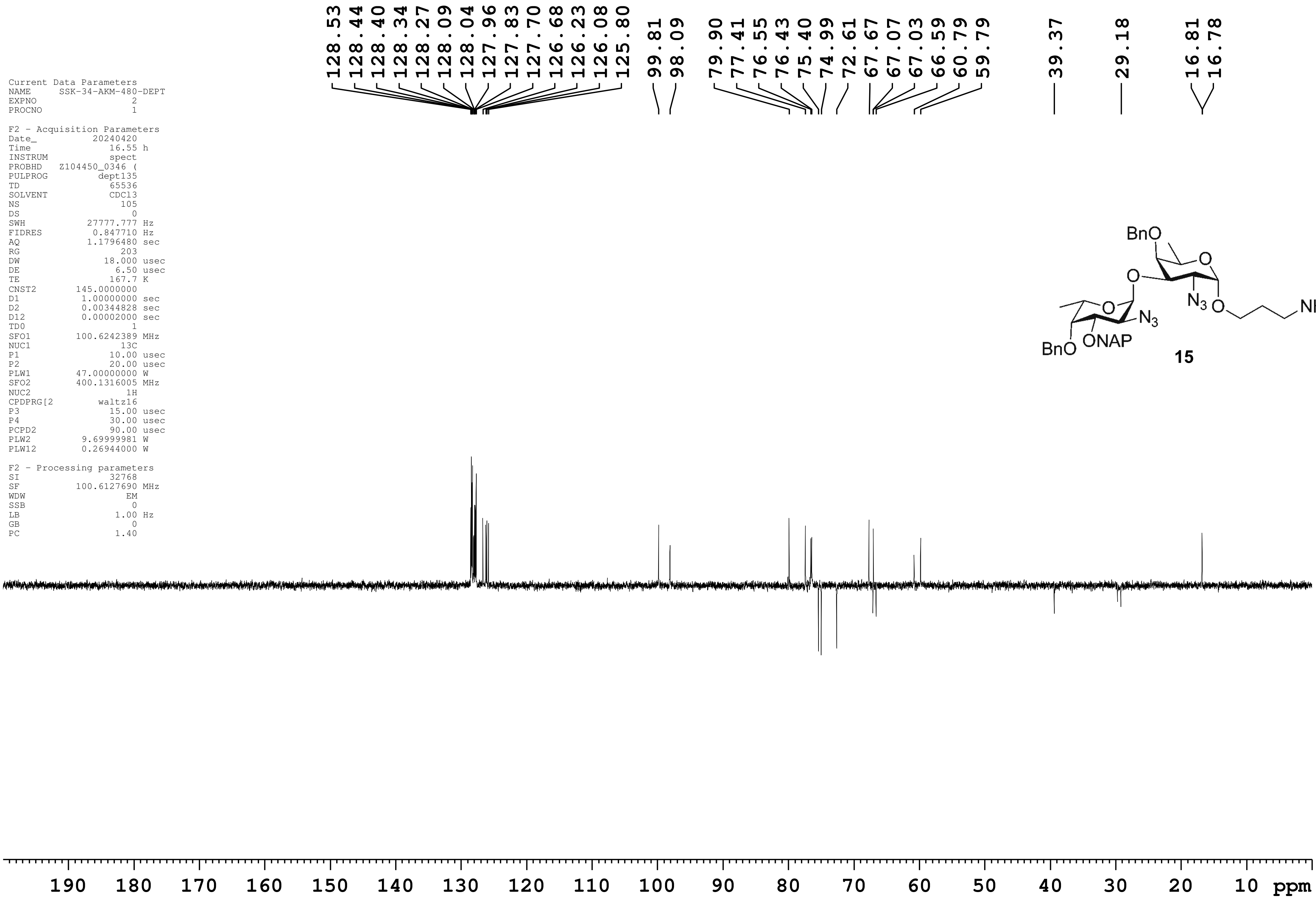

15

SSK-34-AKM-480-DS-COSY

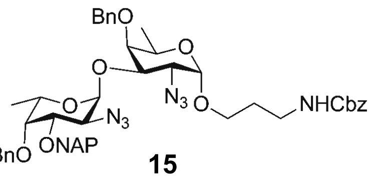

15

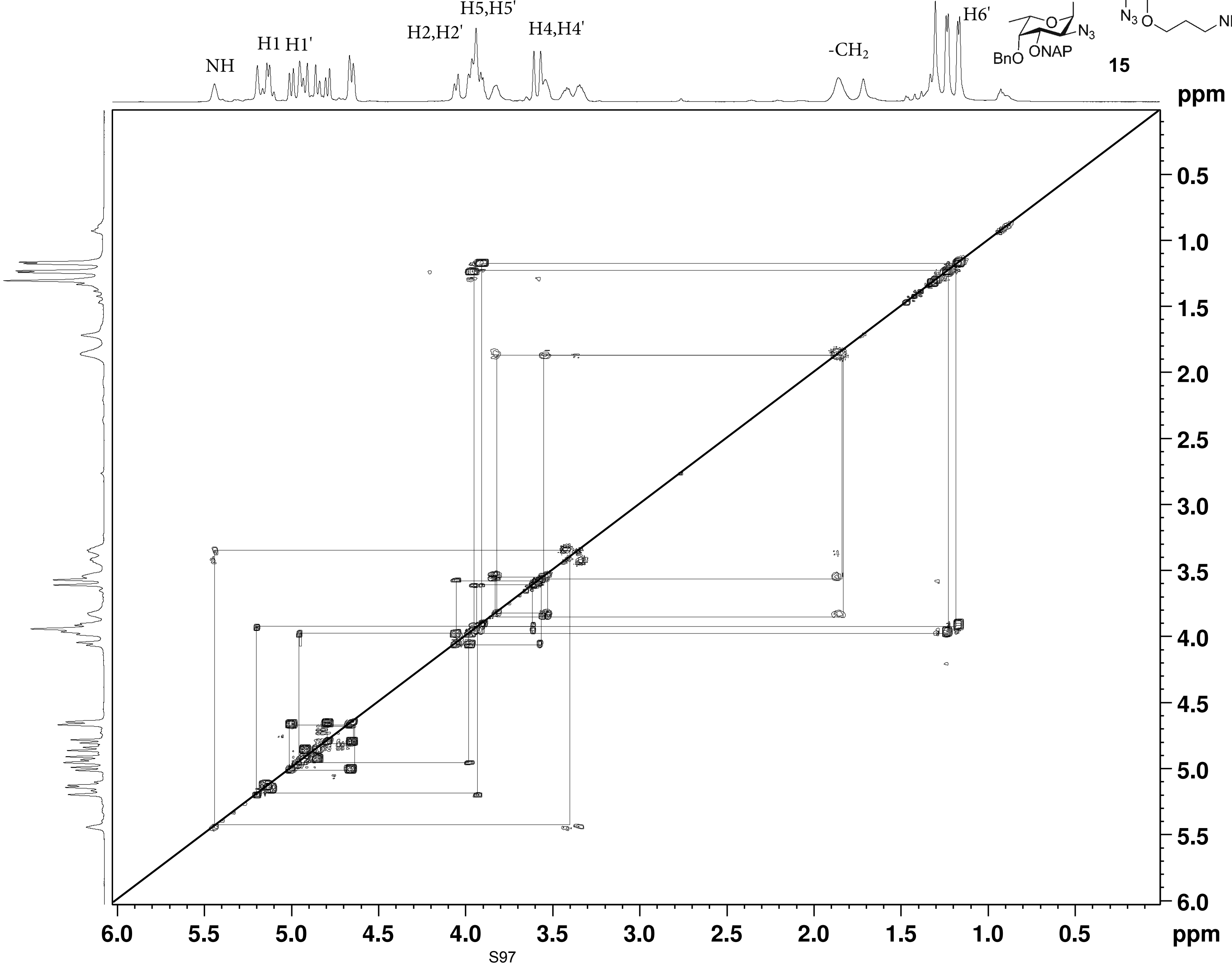


```
Current Data Parameters
NAME      SSK-34-AKM-480-DS-COSY
EXPNO                4
PROCNO               1

F2 - Acquisition Parameters
Date_          20240420
Time              16.16 h
INSTRUM           spect
PROBHD   Z124627_0008 (
PULPROG       cosygpppqf
TD                 2048
SOLVENT           CDCl3
NS                    4
DS                    0
SWH            3009.631 Hz
FIDRES         2.939093 Hz
AQ            0.3402411 sec
RG               197.27
DW              166.133 usec
DE                 6.50 usec
TE                297.7 K
D0           0.00000300 sec
D1           1.00000000 sec
D11          0.03000000 sec
D12          0.00002000 sec
D13          0.00000400 sec
D16          0.00020000 sec
IN0          0.00033220 sec
TDav                  1
SFO1        500.1315110 MHz
NUC1                 1H
P0                15.00 usec
P1                15.00 usec
P17             2500.00 usec
PLW1         9.39999962 W
PLW10        2.34999990 W
GPNAM[1]      SMSQ10.100
GPZ1              10.00 %
P16             1000.00 usec

F1 - Acquisition parameters
TD                  128
SFO1           500.1315 MHz
FIDRES        47.034920 Hz
SW                6.019 ppm
FnMODE               QF

F2 - Processing parameters
SI                 1024
SF          500.1300000 MHz
WDW               QSINE
SSB                   0
LB                    0 Hz
GB                    0
PC                 1.40

F1 - Processing parameters
SI                 1024
MC2                  QF
SF          500.1300000 MHz
WDW               QSINE
SSB                   0
LB                    0 Hz
GB                    0
```

SSK-34-AKM-480-DS-HSQC

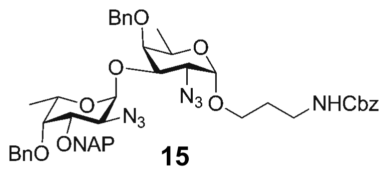

15

```
Current Data Parameters
NAME      SSK-34-AKM-480-DS-HSQC
EXPNO                      5
PROCNO                     1

F2 - Acquisition Parameters
Date_            20240420
Time                16.23 h
INSTRUM             spect
PROBHD   Z124627_0008 (
PULPROG  hsqcedetgpsisp2.2
TD                   1024
SOLVENT             CDCl3
NS                      2
DS                      0
SWH              4295.533 Hz
FIDRES           8.389712 Hz
AQ              0.1191936 sec
RG                 197.27
DW                116.400 usec
DE                   6.50 usec
TE                  297.5 K
CNST2         145.0000000
CNST17         -0.5000000
D0             0.00000300 sec
D1             1.00000000 sec
D4             0.00172414 sec
D11            0.03000000 sec
D16            0.00020000 sec
D21            0.00360000 sec
D24            0.00089000 sec
IN0            0.00002450 sec
TDav                    1
ZGOPTNS
SFO1          500.1323629 MHz
NUC1                   1H
P1                  15.00 usec
P2                  30.00 usec
PLW1           9.39999962 W
SFO2          125.7690478 MHz
NUC2                  13C
CPDPRG[2 bi_p5m4sp_4sp.2
P3                  10.00 usec
P14                500.00 usec
P24               2000.00 usec
P63               1500.00 usec
PLW0                    0 W
PLW2          50.00000000 W
PLW12          1.02040005 W
SPNAM[3] Crp60,0.5,20.1
SPOAL3              0.500
SPOFFS3                 0 Hz
SPW3           7.63940001 W
SPNAM[7]    Crp60comp.4
SPOAL7              0.500
SPOFFS7                 0 Hz
SPW7           7.63940001 W
SPNAM[14 Crp32,1.5,20.2
SPOAL14             0.500
SPOFFS14                0 Hz
SPW14          3.25950003 W
SPNAM[31 Crp32,1.5,20.2
SPOAL31             0.500
SPOFFS31                0 Hz
SPW31          0.81487000 W
GPNAM[1]     SMSQ10.100
GPZ1                80.00 %
GPNAM[2]     SMSQ10.100
GPZ2                20.10 %
GPNAM[3]     SMSQ10.100
GPZ3                11.00 %
GPNAM[4]     SMSQ10.100
GPZ4                -5.00 %
P16               1000.00 usec
P19                600.00 usec

F1 - Acquisition parameters
TD                    177
SFO1              125.769 MHz
FIDRES         230.600708 Hz
SW                162.267 ppm
FnMODE    Echo-Antiecho

F2 - Processing parameters
SI                   1024
SF            500.1300000 MHz
WDW                 QSINE
SSB                     2
LB                      0 Hz
GB                      0
PC                   1.40

F1 - Processing parameters
SI                   1024
MC2         echo-antiecho
SF            125.7577890 MHz
WDW                 QSINE
SSB                     2
LB                      0 Hz
GB                      0
```

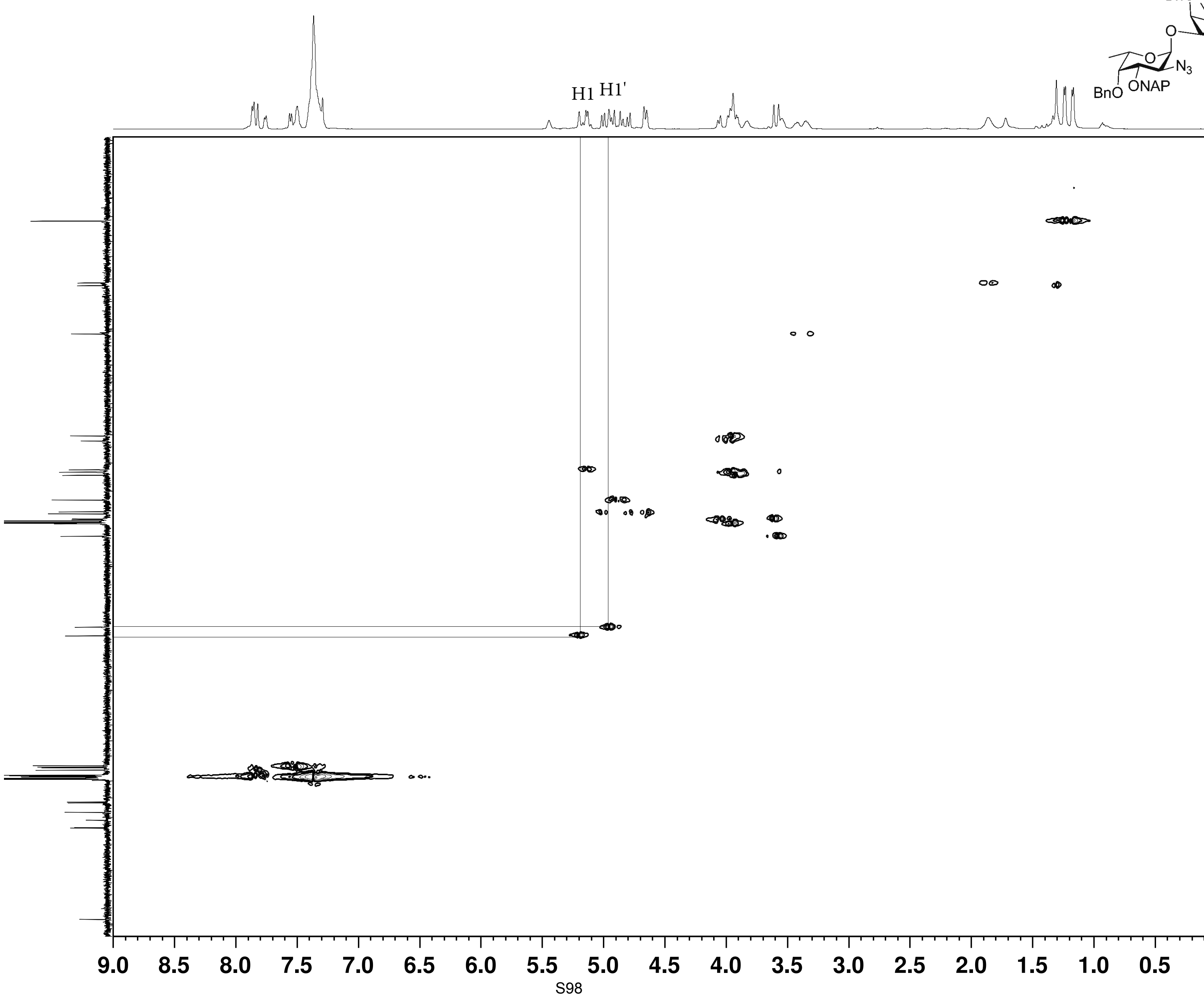

SSK-34-AKM-501-DSA-1

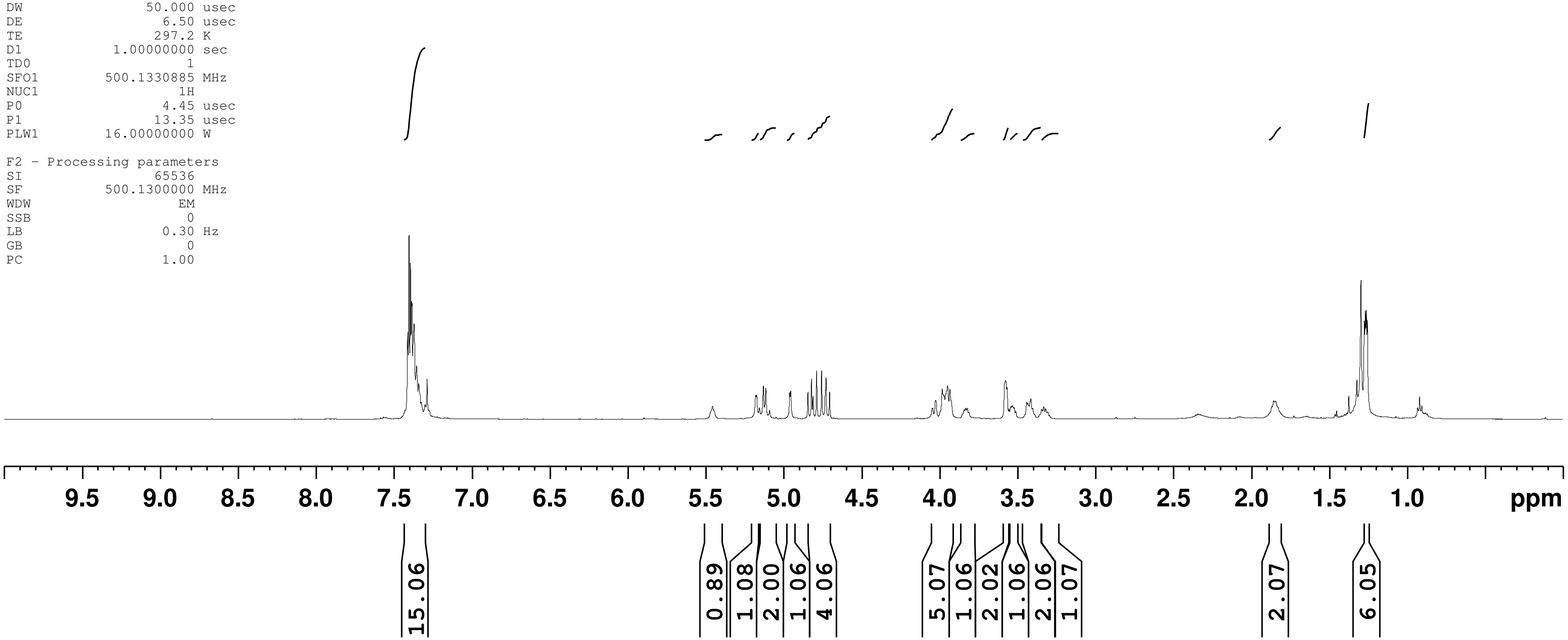


```
Current Data Parameters
NAME     SSK-34-AKM-501-DSA-1
EXPNO                  1
PROCNO                 1

F2 - Acquisition Parameters
Date_           20240605
Time               10.53 h
INSTRUM            spect
PROBHD   Z119470_0087 (
PULPROG             zg30
TD                 65536
SOLVENT            CDCl3
NS                    16
DS                     0
SWH            10000.000 Hz
FIDRES          0.305176 Hz
AQ             3.2767999 sec
RG                 30.72
DW                50.000 usec
DE                  6.50 usec
TE                 297.2 K
D1            1.00000000 sec
TD0                    1
SFO1         500.1330885 MHz
NUC1                  1H
P0                  4.45 usec
P1                 13.35 usec
PLW1         16.00000000 W

F2 - Processing parameters
SI                 65536
SF           500.1300000 MHz
WDW                   EM
SSB                    0
LB                  0.30 Hz
GB                     0
PC                  1.00
```

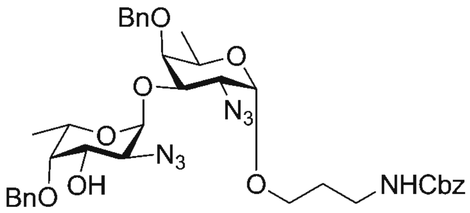

16

SSK-34-AKM-501-DSA-13C

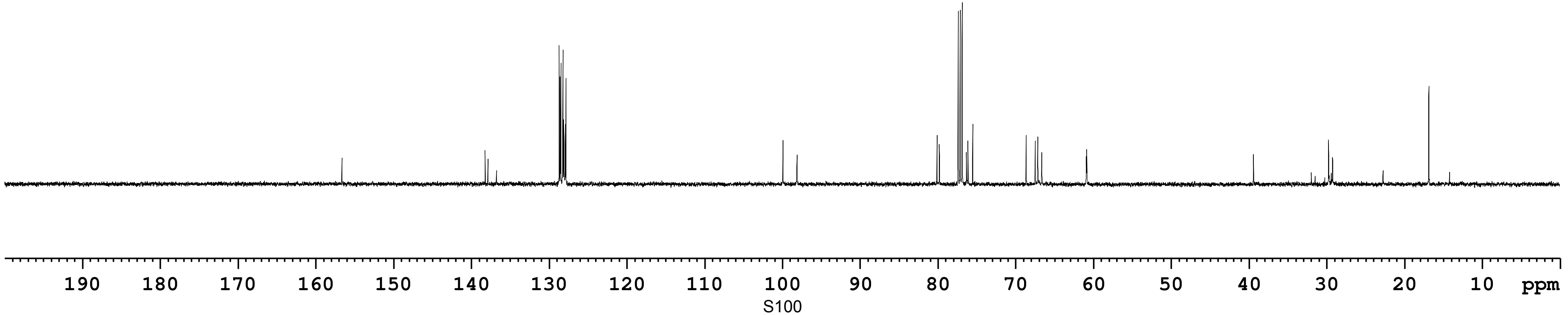


```
Current Data Parameters
NAME     SSK-34-AKM-501-DSA-13C
EXPNO                 2
PROCNO                1

F2 - Acquisition Parameters
Date_          20240605
Time              10.56 h
INSTRUM           spect
PROBHD   Z119470_0087 (
PULPROG          zgpg30
TD                65536
SOLVENT           CDCl3
NS                  105
DS                    0
SWH           34722.223 Hz
FIDRES         1.059638 Hz
AQ            0.9437184 sec
RG               197.27
DW               14.400 usec
DE                 6.50 usec
TE                297.6 K
D1           1.00000000 sec
D11          0.03000000 sec
TD0                   1
SFO1        125.7721254 MHz
NUC1                13C
P0                 2.97 usec
P1                 8.90 usec
PLW1       103.00000000 W
SFO2        500.1320005 MHz
NUC2                 1H
CPDPRG[2        waltz16
PCPD2             80.00 usec
PLW2        16.00000000 W
PLW12        0.44556001 W
PLW13        0.22411001 W

F2 - Processing parameters
SI                32768
SF          125.7577890 MHz
WDW                  EM
SSB                   0
LB                 1.00 Hz
GB                    0
PC                 1.40
```

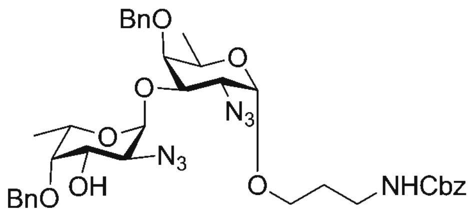

16

SSK-34-AKM-501-DSA-DEPT

```
Current Data Parameters
NAME     SSK-34-AKM-501-DSA-DEPT
EXPNO                  3
PROCNO                 1

F2 - Acquisition Parameters
Date_           20240605
Time               10.58 h
INSTRUM            spect
PROBHD   Z119470_0087 (
PULPROG       deptsp135
TD                 65536
SOLVENT            CDCl3
NS                    28
DS                     0
SWH            20161.291 Hz
FIDRES          0.615274 Hz
AQ             1.6252928 sec
RG                197.27
DW                24.800 usec
DE                  6.50 usec
TE                 297.7 K
CNST2        145.0000000
D1            1.00000000 sec
D2            0.00344828 sec
D12           0.00002000 sec
TD0                    1
SFO1         125.7678486 MHz
NUC1                 13C
P1                  8.90 usec
P13              2000.00 usec
PLW0                   0 W
PLW1        103.00000000 W
SPNAM[5]     Crp60comp.4
SPOAL5             0.500
SPOFFS5                0 Hz
SPW5         12.46500015 W
SFO2         500.1315995 MHz
NUC2                  1H
CPDPRG[2         waltz16
P3                 13.35 usec
P4                 26.70 usec
PCPD2              80.00 usec
PLW2         16.00000000 W
PLW12         0.44556001 W

F2 - Processing parameters
SI                 32768
SF           125.7577890 MHz
WDW                   EM
SSB                    0
LB                  1.00 Hz
GB                     0
PC                  1.40
```

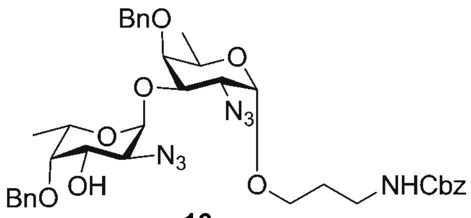

16

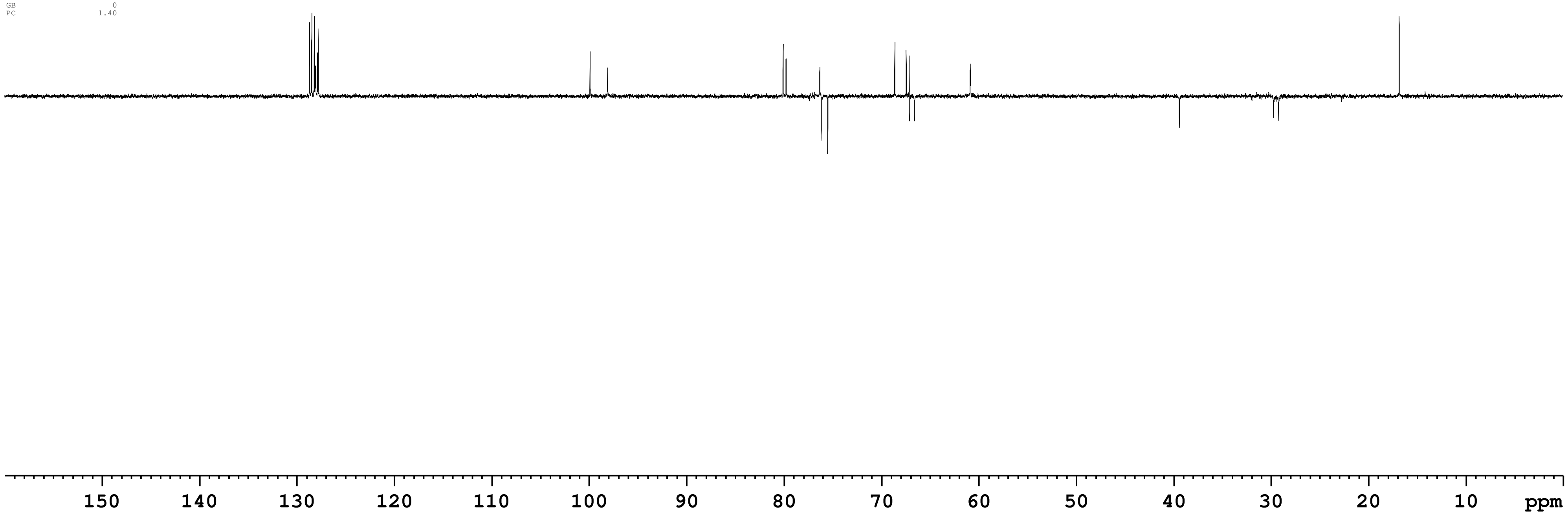

SSK-34-AKM-501-DSA-COSY

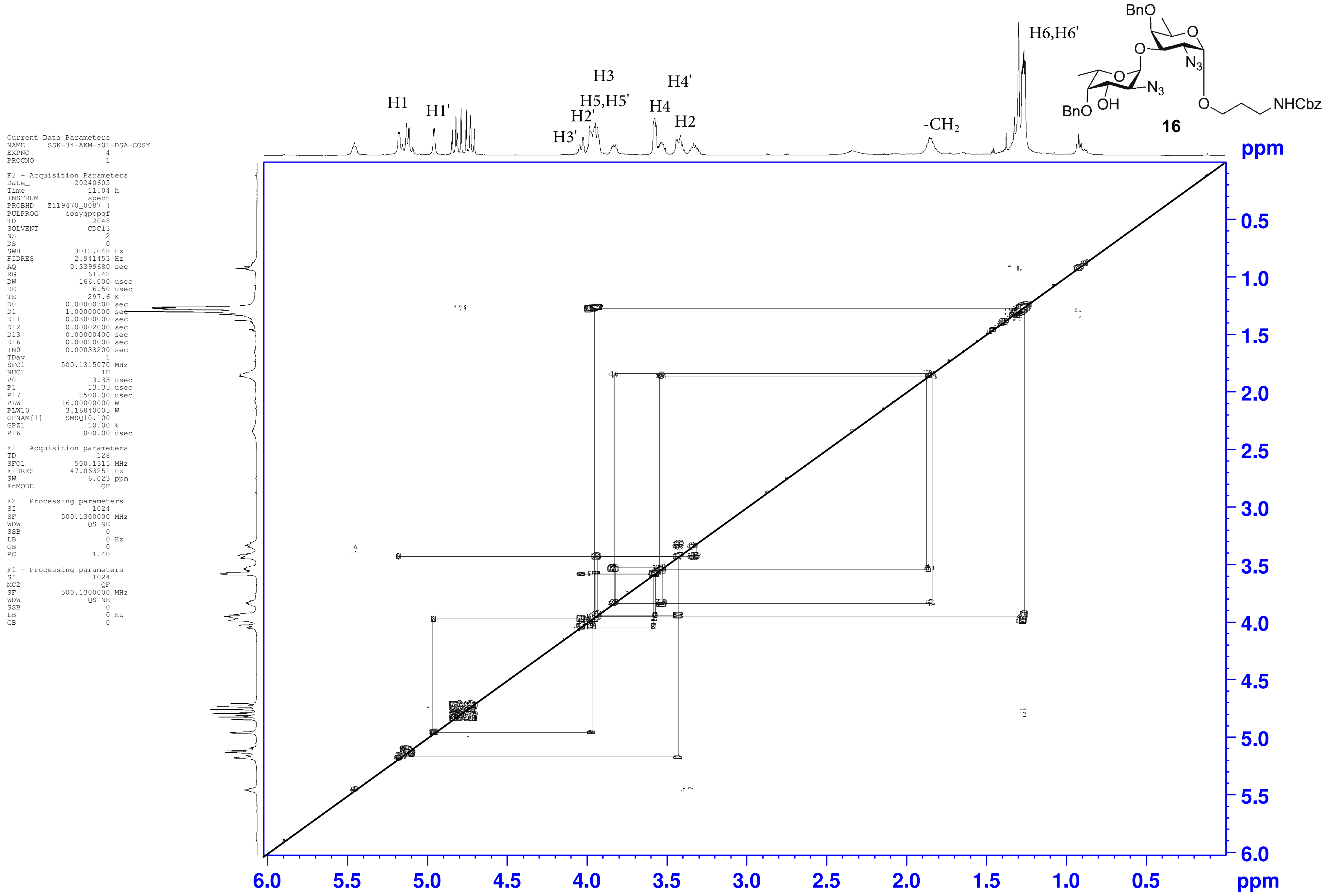

SSK-34-AKM-501-DSA-HSQC

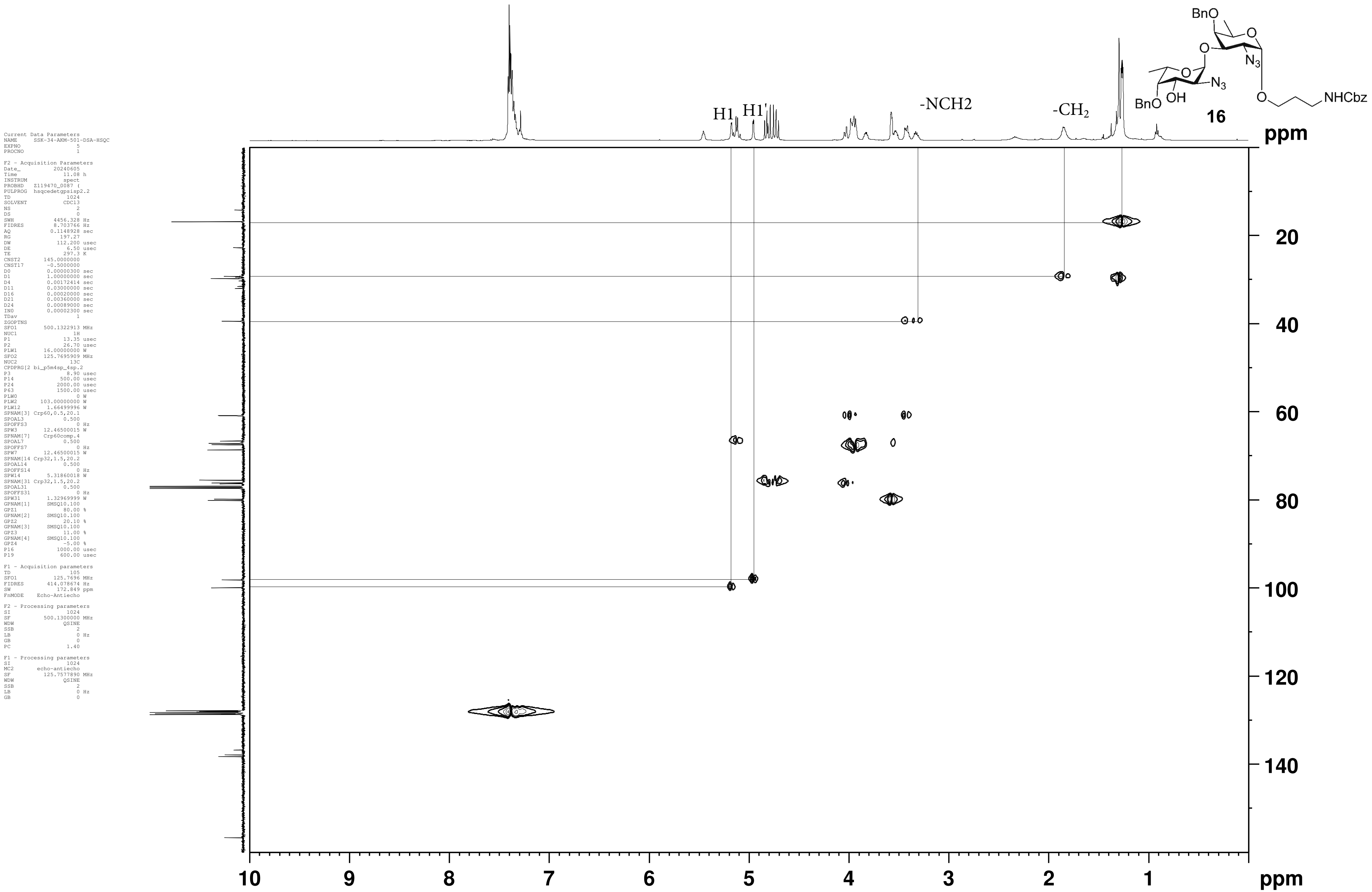

SSK-34-AKM-CP8-DI-13-1H

```
Current Data Parameters
NAME     SSK-34-AKM-CP8-DI-13-1H
EXPNO                 1
PROCNO                1

F2 - Acquisition Parameters
Date_          20240902
Time              19.22 h
INSTRUM           spect
PROBHD   Z119470_0087 (
PULPROG            zg30
TD                65536
SOLVENT             D2O
NS                   34
DS                    2
SWH           10000.000 Hz
FIDRES         0.305176 Hz
AQ            3.2767999 sec
RG                30.72
DW               50.000 usec
DE                 6.50 usec
TE                298.1 K
D1           1.00000000 sec
TD0                   1
SFO1        500.1330885 MHz
NUC1                 1H
P0                 4.45 usec
P1                13.35 usec
PLW1        16.00000000 W

F2 - Processing parameters
SI                65536
SF          500.1300000 MHz
WDW                  EM
SSB                   0
LB                 0.30 Hz
GB                    0
PC                 1.00
```

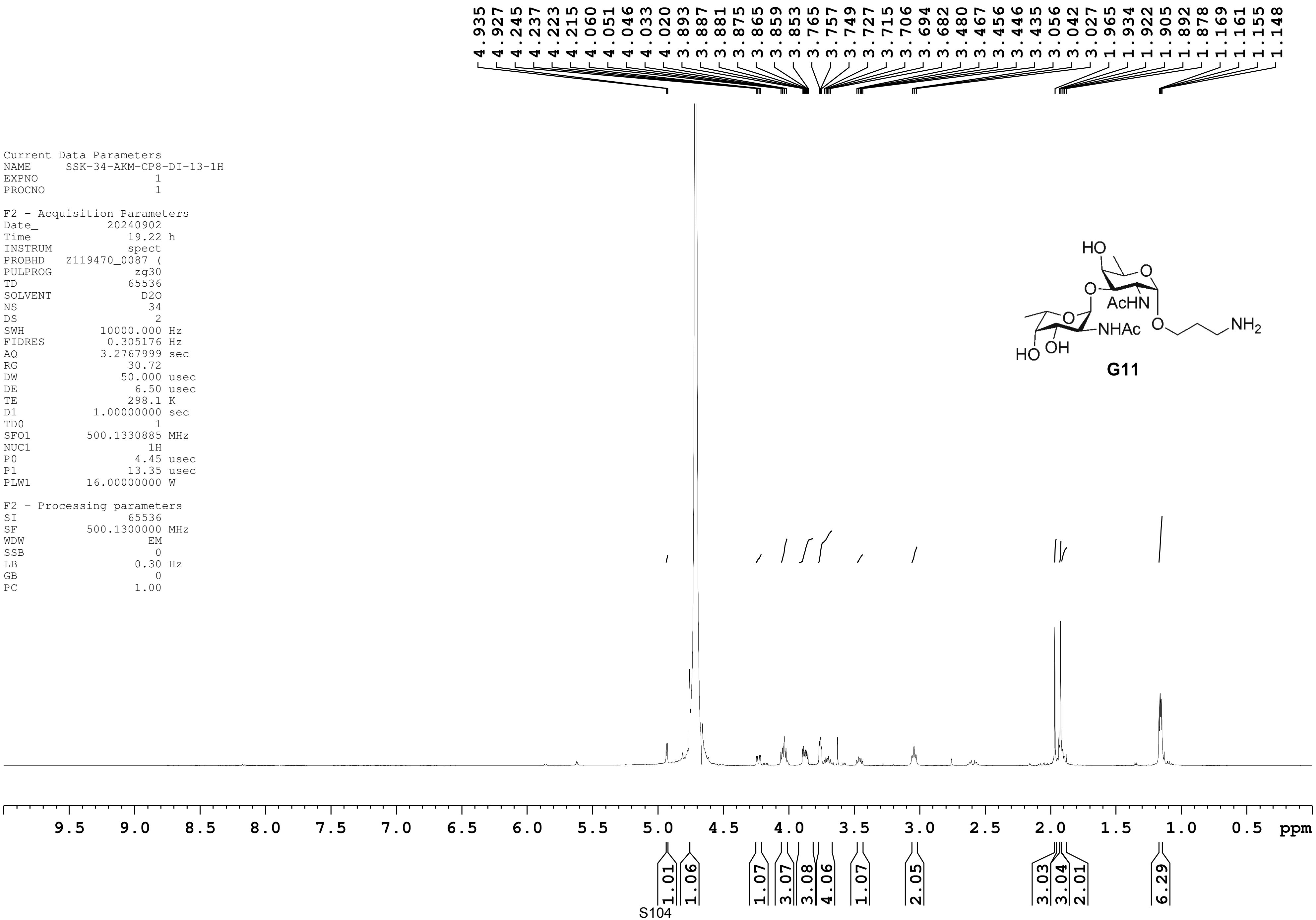

G11

SSK-34-AKM-CP8-DI-13-13C

```
Current Data Parameters
NAME      SSK-34-AKM-CP8-DI-13-13C
EXPNO                    2
PROCNO                   1

F2 - Acquisition Parameters
Date_          20240903
Time              12.16 h
INSTRUM           spect
PROBHD   Z119470_0087 (
PULPROG         zgpg30
TD               65536
SOLVENT            D2O
NS                3999
DS                   0
SWH          34722.223 Hz
FIDRES        1.059638 Hz
AQ           0.9437184 sec
RG              197.27
DW              14.400 usec
DE                6.50 usec
TE               298.4 K
D1          1.00000000 sec
D11         0.03000000 sec
TD0                  1
SFO1       125.7721254 MHz
NUC1               13C
P0                2.97 usec
P1                8.90 usec
PLW1      103.00000000 W
SFO2       500.1320005 MHz
NUC2                1H
CPDPRG[2        waltz16
PCPD2            80.00 usec
PLW2       16.00000000 W
PLW12       0.44556001 W
PLW13       0.22411001 W

F2 - Processing parameters
SI               32768
SF         125.7577890 MHz
WDW                 EM
SSB                  0
LB                1.00 Hz
GB                   0
PC                1.40
```

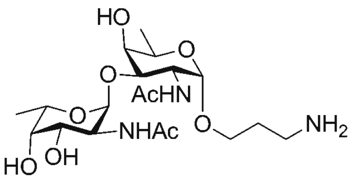

G11

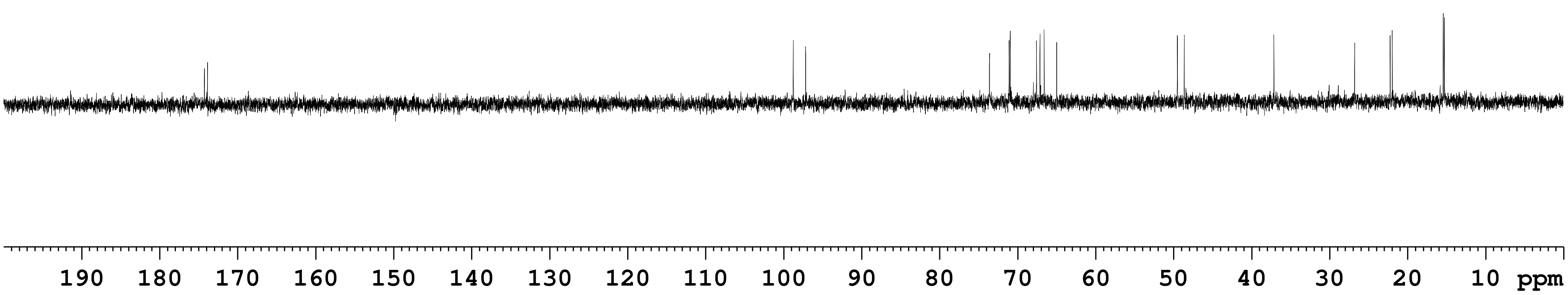

SSK-34-AKM-DS-FINAL-DEPT

```
Current Data Parameters
NAME     SSK-34-AKM-CP8-DI-13-DEPT
EXPNO                    3
PROCNO                   1

F2 - Acquisition Parameters
Date_           20240913
Time               18.14 h
INSTRUM            spect
PROBHD   Z119470_0087 (
PULPROG       deptsp135
TD                 65536
SOLVENT              D2O
NS                   250
DS                     0
SWH            20161.291 Hz
FIDRES          0.615274 Hz
AQ             1.6252928 sec
RG                197.27
DW                24.800 usec
DE                  6.50 usec
TE                 297.4 K
CNST2        145.0000000
D1            1.00000000 sec
D2            0.00344828 sec
D12           0.00002000 sec
TD0                    1
SFO1         125.7678486 MHz
NUC1                 13C
P1                  8.90 usec
P13              2000.00 usec
PLW0                   0 W
PLW1        103.00000000 W
SPNAM[5]    Crp60comp.4
SPOAL5             0.500
SPOFFS5                0 Hz
SPW5         12.46500015 W
SFO2         500.1315995 MHz
NUC2                  1H
CPDPRG[2         waltz16
P3                 13.35 usec
P4                 26.70 usec
PCPD2              80.00 usec
PLW2         16.00000000 W
PLW12         0.44556001 W

F2 - Processing parameters
SI                 32768
SF           125.7577890 MHz
WDW                   EM
SSB                    0
LB                  1.00 Hz
GB                     0
PC                  1.40
```

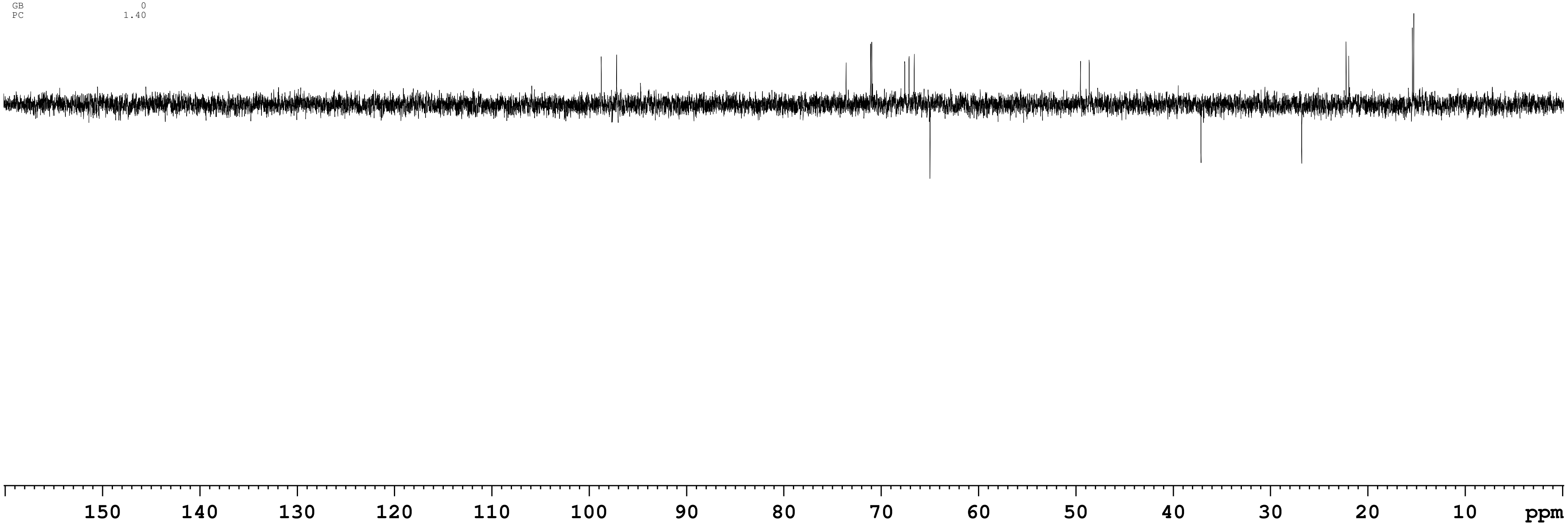

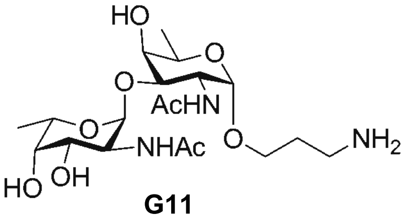

G11

SSK-34-AKM-CP8-DI-FINAL-HSQC

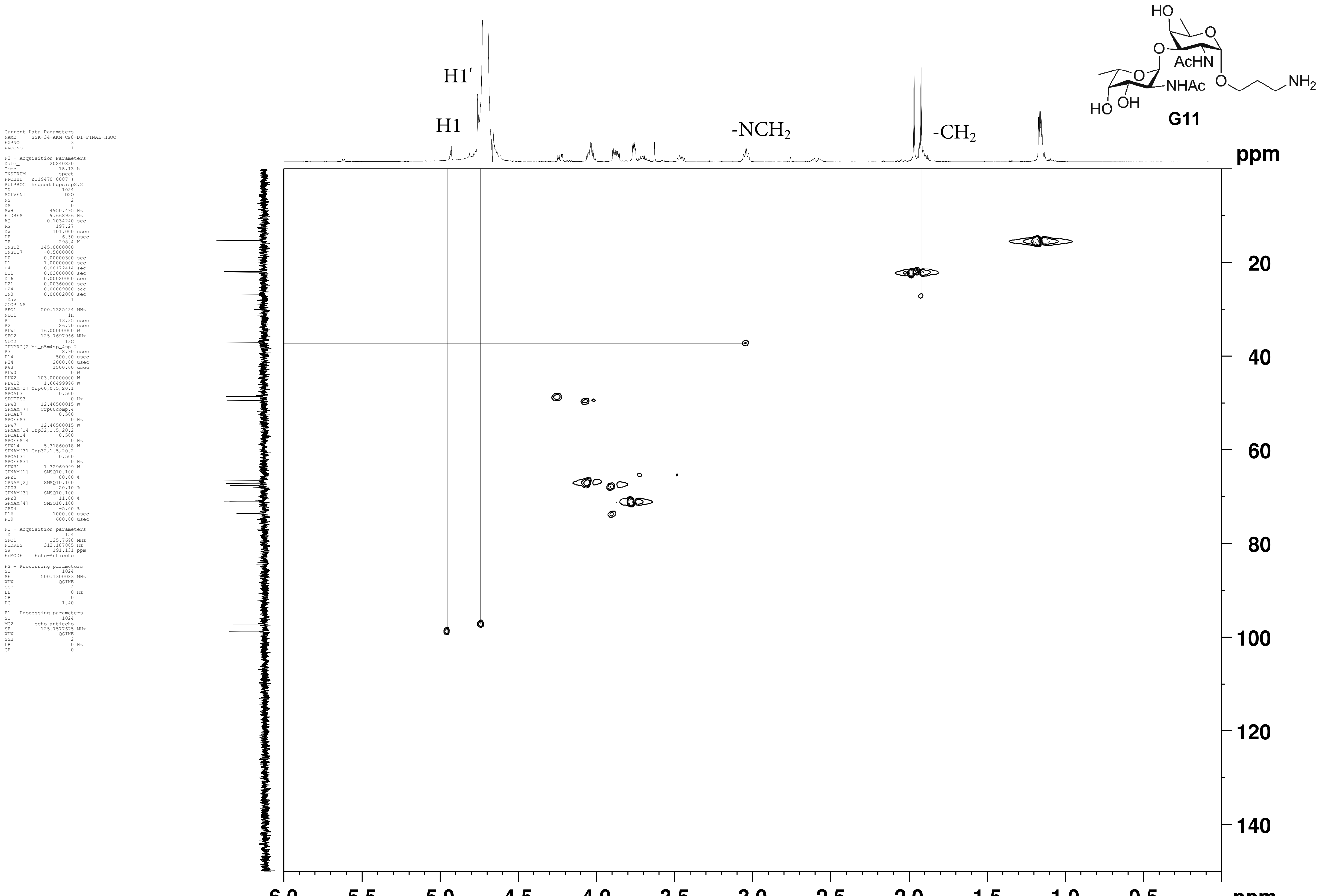

SSK-34-AKM-453-1H

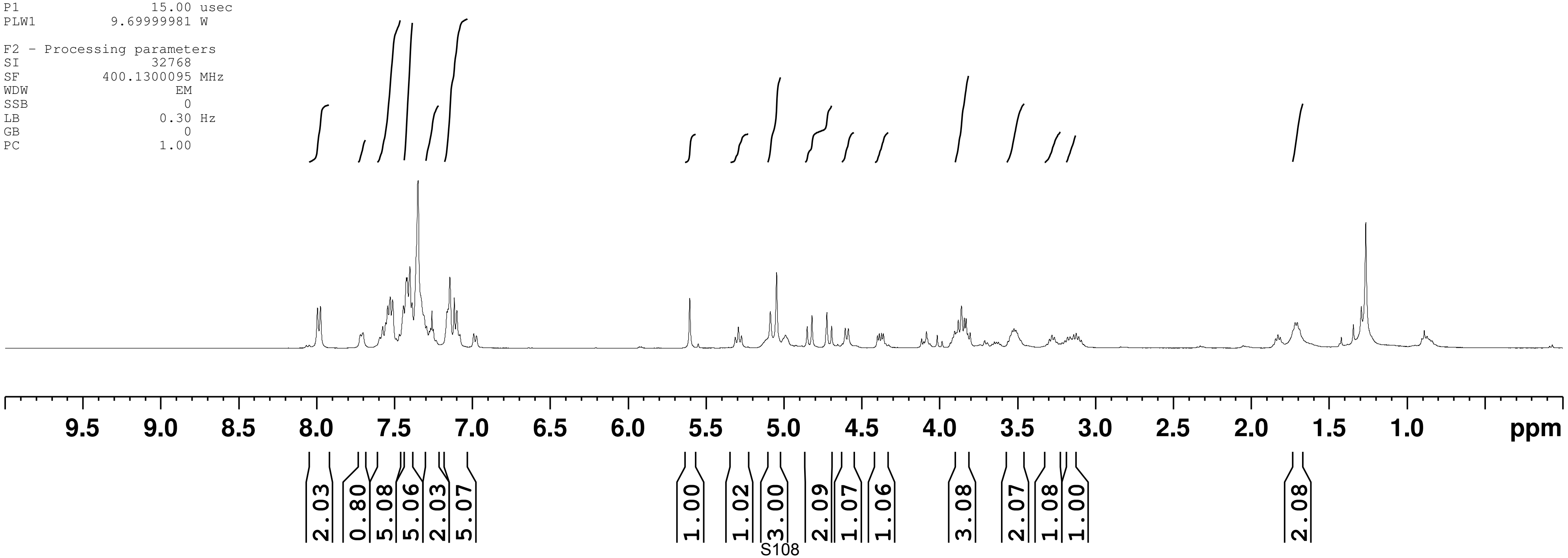

Current Data Parameters
NAME SSK-34-AKM-453-1H
EXPNO 1
PROCNO 1

F2 - Acquisition Parameters
Date_ 20240306
Time 12.01 h
INSTRUM spect
PROBHD Z104450_0346 (
PULPROG zg30
TD 54274
SOLVENT CDCl3
NS 8
DS 0
SWH 8223.685 Hz
FIDRES 0.303043 Hz
AQ 3.2998593 sec
RG 80.6
DW 60.800 usec
DE 6.50 usec
TE 294.9 K
D1 1.00000000 sec
TD0 1
SFO1 400.1324710 MHz
NUC1 1H
P0 5.00 usec
P1 15.00 usec
PLW1 9.69999981 W

F2 - Processing parameters
SI 32768
SF 400.1300095 MHz
WDW EM
SSB 0
LB 0.30 Hz
GB 0
PC 1.00

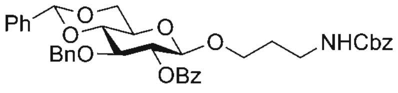

17

SSK-34-AKM-453-13C

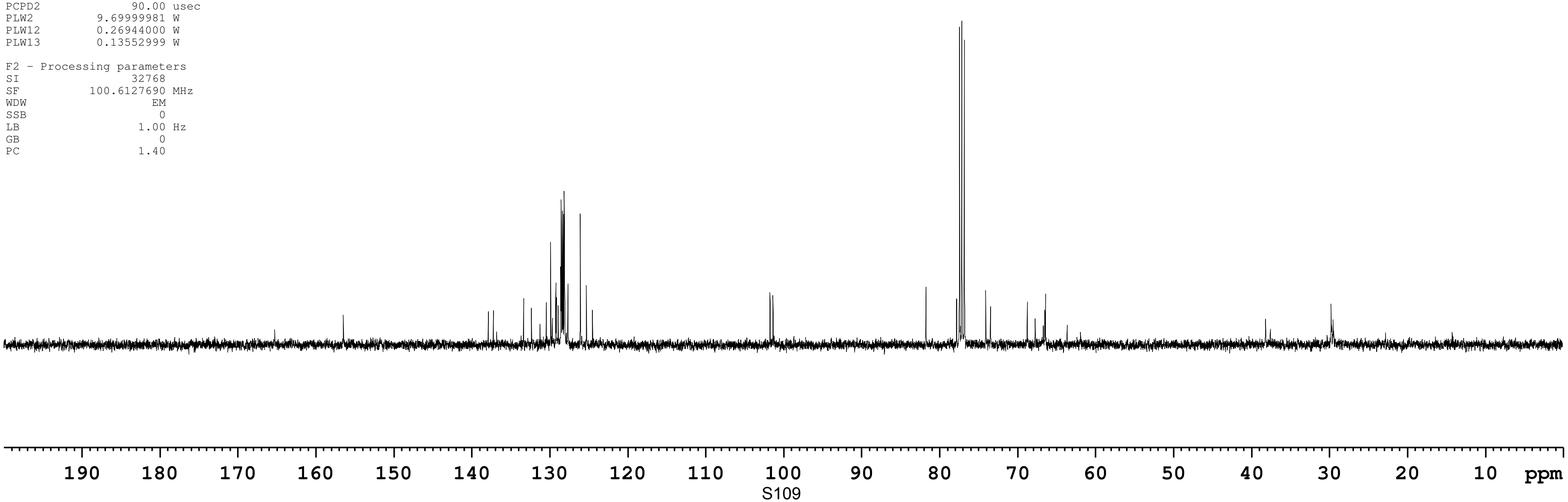


```
Current Data Parameters
NAME     SSK-34-AKM-453-13C
EXPNO                  2
PROCNO                 1

F2 - Acquisition Parameters
Date_           20240306
Time               12.09 h
INSTRUM            spect
PROBHD   Z104450_0346 (
PULPROG           zgpg30
TD                 65536
SOLVENT            CDCl3
NS                   186
DS                     0
SWH            26041.666 Hz
FIDRES          0.794729 Hz
AQ             1.2582912 sec
RG                  1030
DW                19.200 usec
DE                  6.50 usec
TE                 295.1 K
D1            1.00000000 sec
D11           0.03000000 sec
TD0                    1
SFO1         100.6238364 MHz
NUC1                 13C
P0                  3.33 usec
P1                 10.00 usec
PLW1         47.00000000 W
SFO2         400.1316005 MHz
NUC2                  1H
CPDPRG[2         waltz16
PCPD2              90.00 usec
PLW2          9.69999981 W
PLW12         0.26944000 W
PLW13         0.13552999 W

F2 - Processing parameters
SI                 32768
SF           100.6127690 MHz
WDW                   EM
SSB                    0
LB                  1.00 Hz
GB                     0
PC                  1.40
```

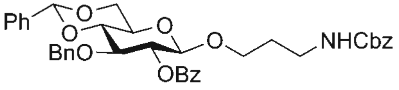

17

SSK-34-AKM-453-DEPT

Current Data Parameters
NAME SSK-34-AKM-453-DEPT
EXPNO 3
PROCNO 1

F2 - Acquisition Parameters
Date_ 20240306
Time 12.25 h
INSTRUM spect
PROBHD Z104450_0346 (
PULPROG dept135
TD 65536
SOLVENT CDCl3
NS 426
DS 0
SWH 27777.777 Hz
FIDRES 0.847710 Hz
AQ 1.1796480 sec
RG 203
DW 18.000 usec
DE 6.50 usec
TE 295.4 K
CNST2 145.0000000
D1 1.00000000 sec
D2 0.00344828 sec
D12 0.00002000 sec
TD0 1
SFO1 100.6242389 MHz
NUC1 13C
P1 10.00 usec
P2 20.00 usec
PLW1 47.00000000 W
SFO2 400.1316005 MHz
NUC2 1H
CPDPRG[2 waltz16
P3 15.00 usec
P4 30.00 usec
PCPD2 90.00 usec
PLW2 9.69999981 W
PLW12 0.26944000 W

F2 - Processing parameters
SI 32768
SF 100.6127690 MHz
WDW EM
SSB 0
LB 1.00 Hz
GB 0
PC 1.40

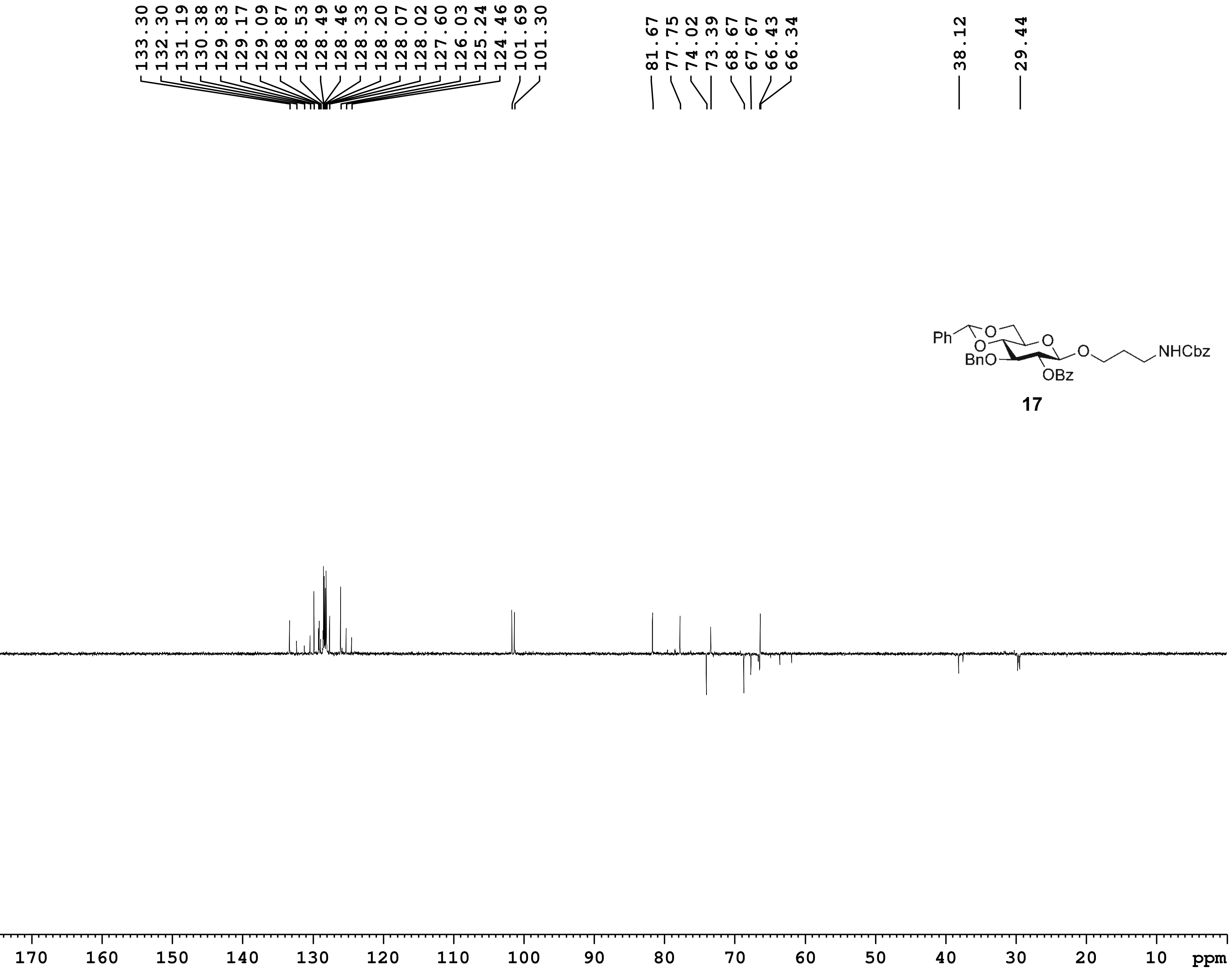

17

SSK-34-AKM-455-1H

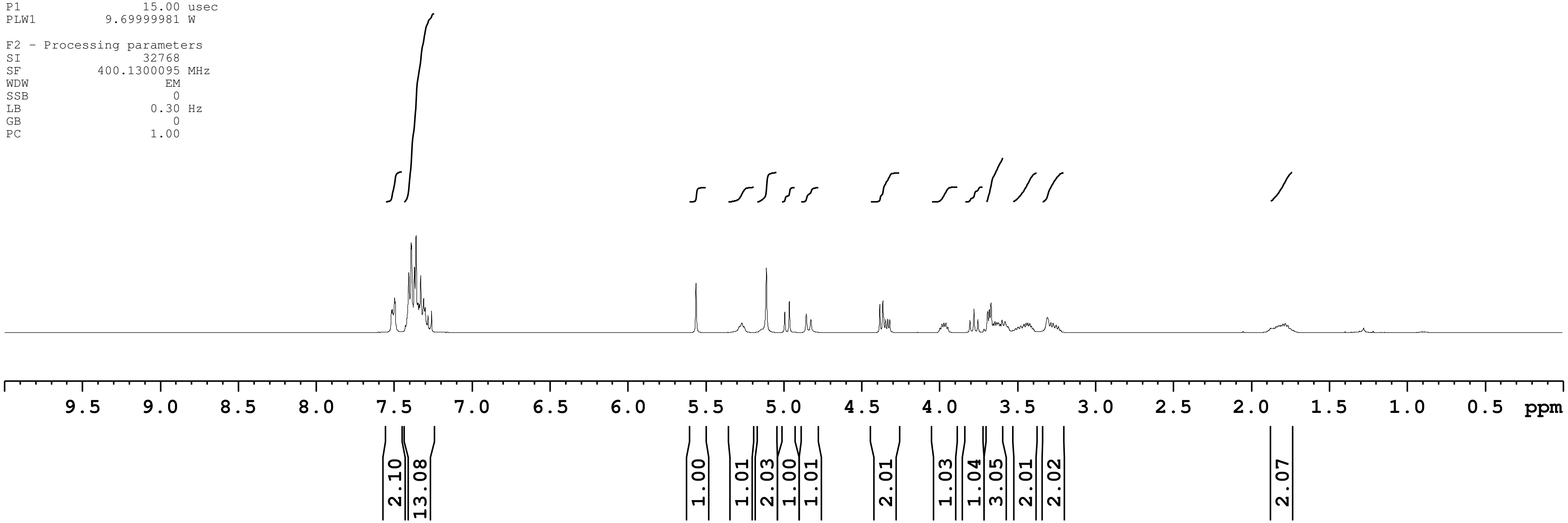

Current Data Parameters
NAME SSK-34-AKM-455-1H
EXPNO 1
PROCNO 1

F2 - Acquisition Parameters
Date_ 20240308
Time 0.11 h
INSTRUM spect
PROBHD Z104450_0346 (
PULPROG zg30
TD 54274
SOLVENT CDC13
NS 11
DS 0
SWH 8223.685 Hz
FIDRES 0.303043 Hz
AQ 3.2998593 sec
RG 64
DW 60.800 usec
DE 6.50 usec
TE 295.6 K
D1 1.00000000 sec
TD0 1
SFO1 400.1324710 MHz
NUC1 1H
P0 5.00 usec
P1 15.00 usec
PLW1 9.69999981 W

F2 - Processing parameters
SI 32768
SF 400.1300095 MHz
WDW EM
SSB 0
LB 0.30 Hz
GB 0
PC 1.00

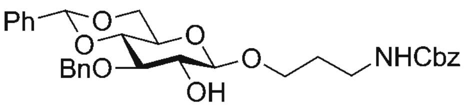

18

SSK-34-AKM-455-13C

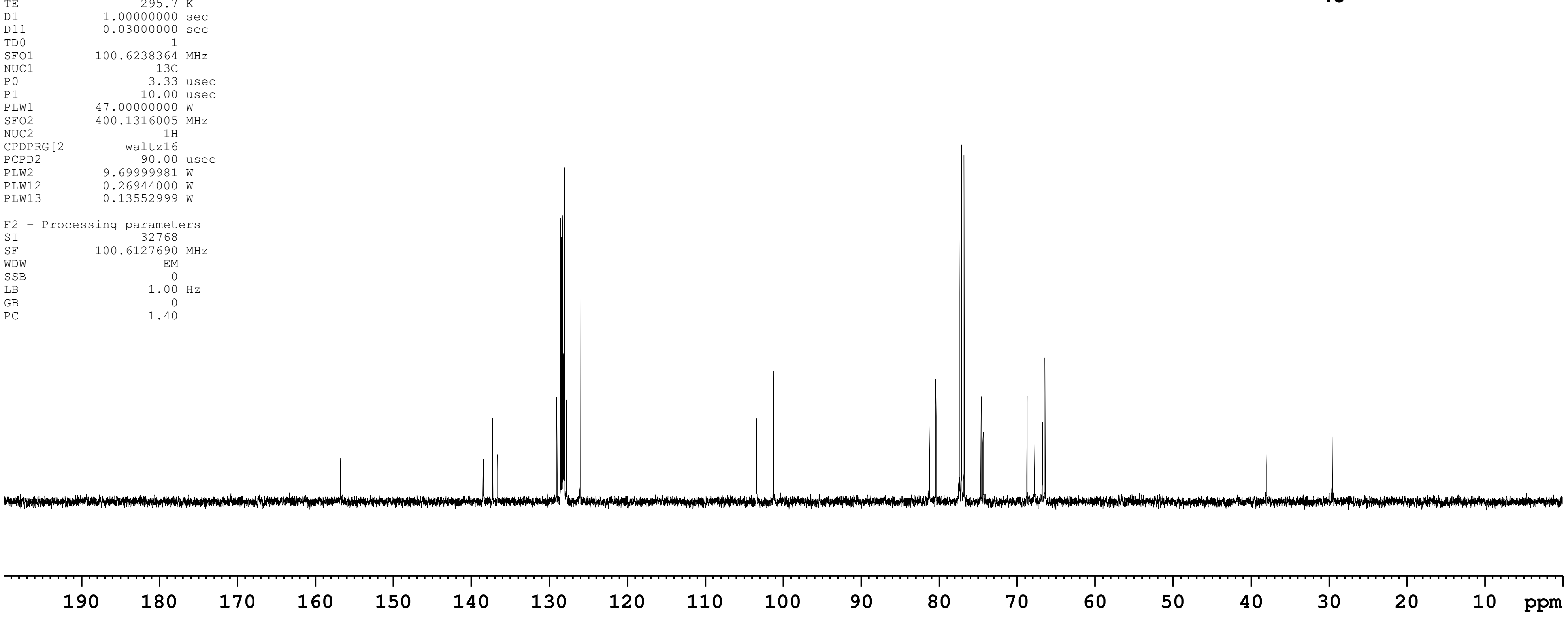

Current Data Parameters

NAME	SSK-34-AKM-455-13C
EXPNO	2
PROCNO	1

F2 - Acquisition Parameters

Date_	20240308
Time	0.15 h
INSTRUM	spect
PROBHD	Z104450_0346 (
PULPROG	zgpg30
TD	65536
SOLVENT	CDCl3
NS	113
DS	0
SWH	26041.666 Hz
FIDRES	0.794729 Hz
AQ	1.2582912 sec
RG	1030
DW	19.200 usec
DE	6.50 usec
TE	295.7 K
D1	1.00000000 sec
D11	0.03000000 sec
TD0	1
SFO1	100.6238364 MHz
NUC1	13C
P0	3.33 usec
P1	10.00 usec
PLW1	47.00000000 W
SFO2	400.1316005 MHz
NUC2	1H
CPDPRG[2	waltz16
PCPD2	90.00 usec
PLW2	9.69999981 W
PLW12	0.26944000 W
PLW13	0.13552999 W

F2 - Processing parameters

SI	32768
SF	100.6127690 MHz
WDW	EM
SSB	0
LB	1.00 Hz
GB	0
PC	1.40

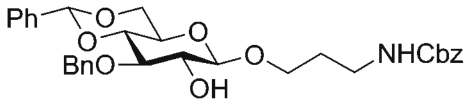

18

SSK-34-AKM-455-DEPT

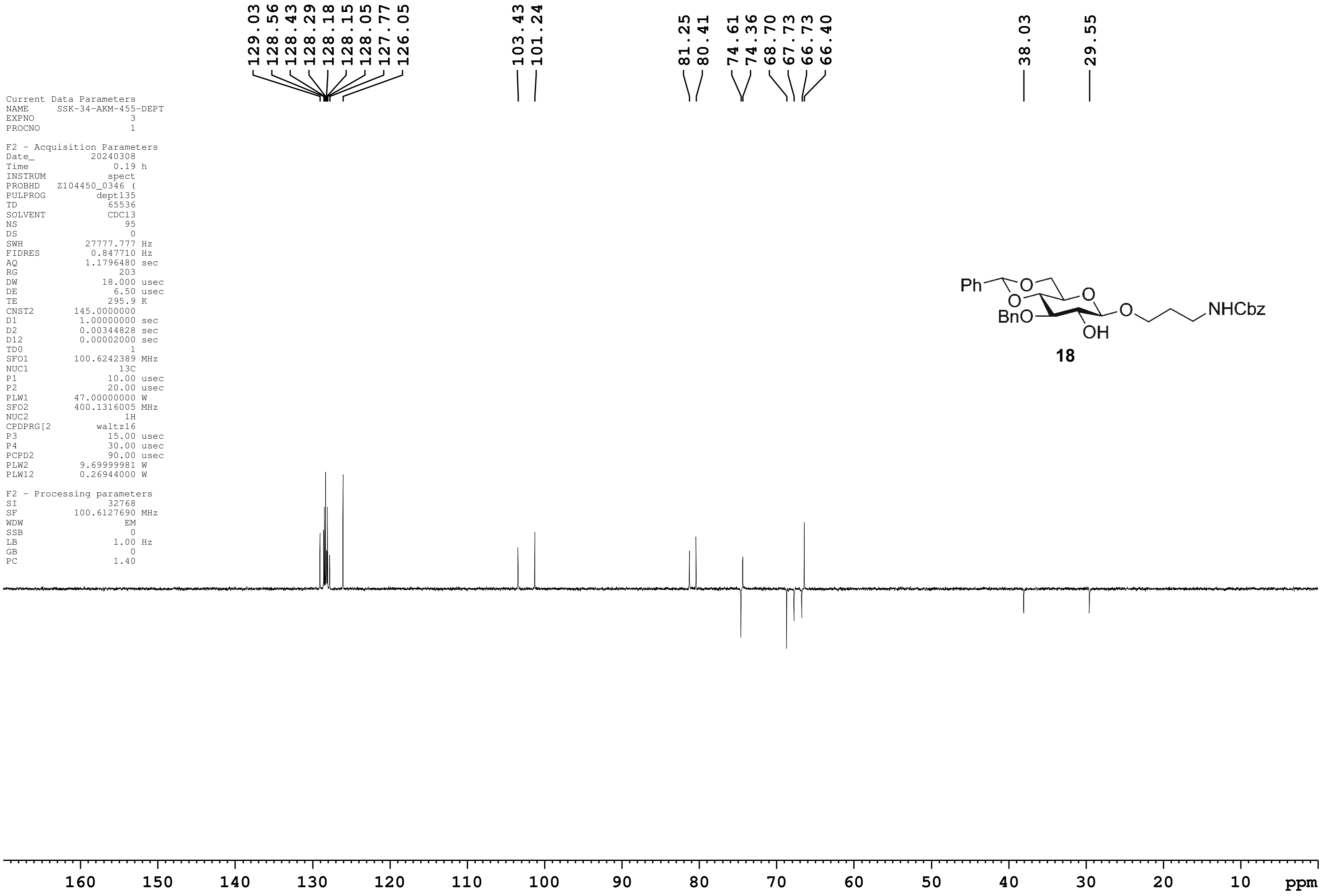

Current Data Parameters
NAME SSK-34-AKM-455-DEPT
EXPNO 3
PROCNO 1

F2 - Acquisition Parameters
Date_ 20240308
Time 0.19 h
INSTRUM spect
PROBHD Z104450_0346 (
PULPROG dept135
TD 65536
SOLVENT CDCl3
NS 95
DS 0
SWH 27777.777 Hz
FIDRES 0.847710 Hz
AQ 1.1796480 sec
RG 203
DW 18.000 usec
DE 6.50 usec
TE 295.9 K
CNST2 145.0000000
D1 1.00000000 sec
D2 0.00344828 sec
D12 0.00002000 sec
TD0 1
SFO1 100.6242389 MHz
NUC1 13C
P1 10.00 usec
P2 20.00 usec
PLW1 47.00000000 W
SFO2 400.1316005 MHz
NUC2 1H
CPDPRG[2 waltz16
P3 15.00 usec
P4 30.00 usec
PCPD2 90.00 usec
PLW2 9.69999981 W
PLW12 0.26944000 W

F2 - Processing parameters
SI 32768
SF 100.6127690 MHz
WDW EM
SSB 0
LB 1.00 Hz
GB 0
PC 1.40

18

SSK-34-AKM-455-COSY

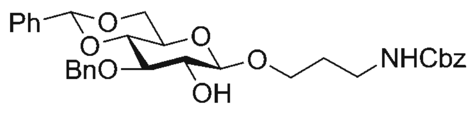

18

```
Current Data Parameters
NAME      SSK-34-AKM-455-COSY
EXPNO                    4
PROCNO                   1

F2 - Acquisition Parameters
Date_             20240308
Time                0.25 h
INSTRUM              spect
PROBHD   Z104450_0346 (
PULPROG           cosygpqf
TD                    2048
SOLVENT              CDCl3
NS                       2
DS                       0
SWH               2429.543 Hz
FIDRES            2.372601 Hz
AQ               0.4214784 sec
RG                      64
DW                 205.800 usec
DE                    6.50 usec
TE                   295.8 K
D0              0.00000300 sec
D1              1.00000000 sec
D13             0.00000400 sec
D16             0.00020000 sec
IN0             0.00041160 sec
TDav                     1
SFO1           400.1312093 MHz
NUC1                    1H
P0                   15.00 usec
P1                   15.00 usec
PLW1            9.69999981 W
GPNAM[1]          SINE.100
GPZ1                 10.00 %
P16                1000.00 usec

F1 - Acquisition parameters
TD                     128
SFO1              400.1312 MHz
FIDRES           37.961613 Hz
SW                   6.072 ppm
FnMODE                  QF

F2 - Processing parameters
SI                    1024
SF             400.1300000 MHz
WDW                   SINE
SSB                      0
LB                       0 Hz
GB                       0
PC                    1.40

F1 - Processing parameters
SI                    1024
MC2                     QF
SF             400.1300000 MHz
WDW                   SINE
SSB                      0
LB                       0 Hz
GB                       0
```

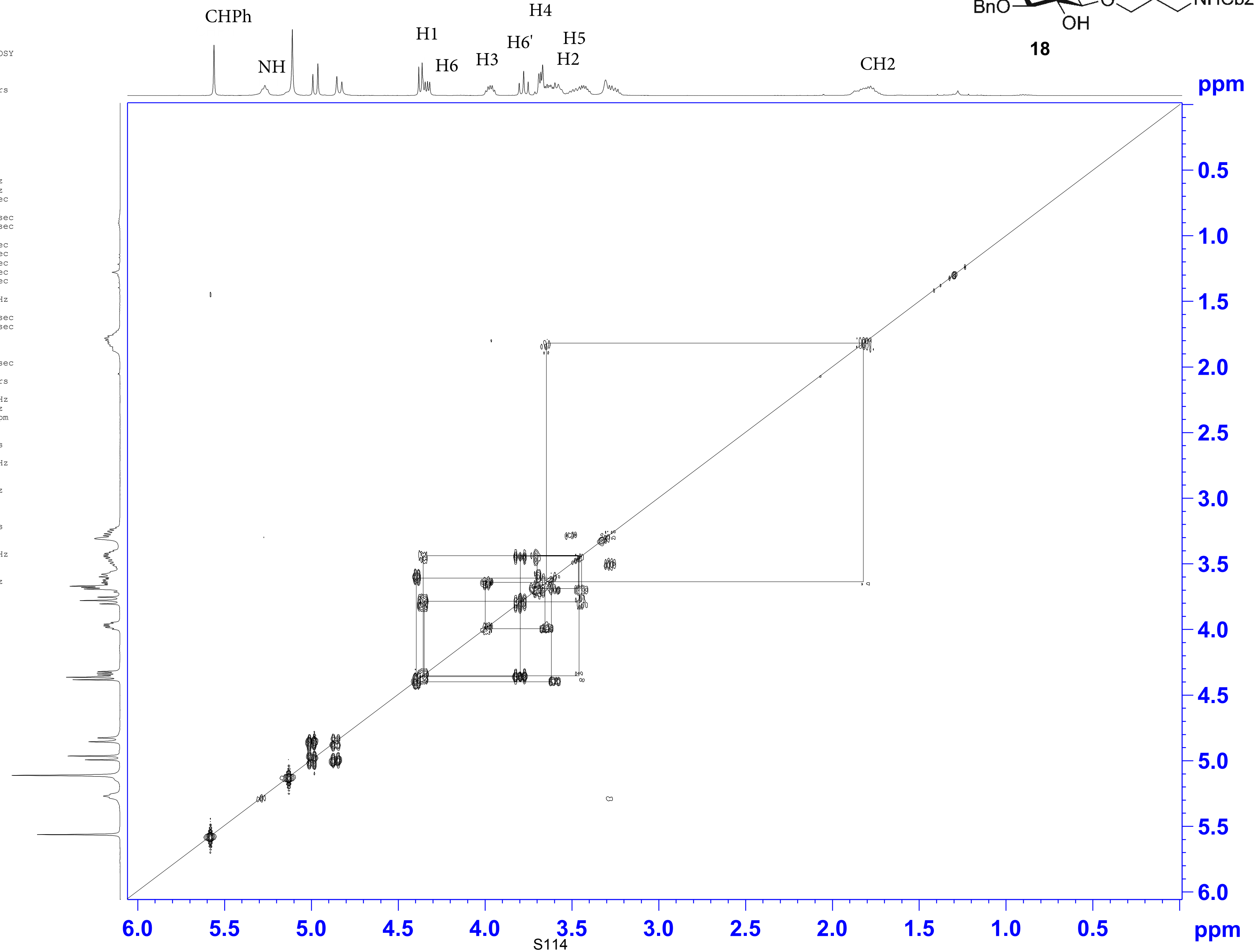

SSK-34-AKM-455-HSQC

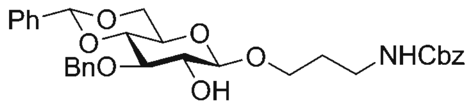

18

```
Current Data Parameters
NAME      SSK-34-AKM-455-HSQC
EXPNO                    5
PROCNO                   1

F2 - Acquisition Parameters
Date_             20240308
Time                0.33 h
INSTRUM              spect
PROBHD   Z104450_0346 (
PULPROG          hsqcetgp
TD                    2048
SOLVENT              CDCl3
NS                       2
DS                       0
SWH               4006.410 Hz
FIDRES            3.912510 Hz
AQ               0.2555904 sec
RG                    2050
DW                 124.800 usec
DE                    6.50 usec
TE                   295.4 K
CNST2          145.0000000
D0              0.00000300 sec
D1              1.00000000 sec
D4              0.00172414 sec
D11             0.03000000 sec
D16             0.00020000 sec
IN0             0.00002760 sec
TDav                     1
ZGOPTNS
SFO1          400.1320038 MHz
NUC1                    1H
P1                   15.00 usec
P2                   30.00 usec
PLW1            9.69999981 W
SFO2          100.6218251 MHz
NUC2                   13C
CPDPRG[2              garp
P3                   10.00 usec
P4                   20.00 usec
PCPD2                80.00 usec
PLW2           47.00000000 W
PLW12           0.73438001 W
GPNAM[1]          SINE.100
GPZ1                 80.00 %
GPNAM[2]          SINE.100
GPZ2                 20.10 %
P16                1000.00 usec

F1 - Acquisition parameters
TD                     187
SFO1              100.6218 MHz
FIDRES          193.753387 Hz
SW                 180.040 ppm
FnMODE     Echo-Antiecho

F2 - Processing parameters
SI                    2048
SF             400.1300000 MHz
WDW                  QSINE
SSB                      2
LB                       0 Hz
GB                       0
PC                    1.40

F1 - Processing parameters
SI                    1024
MC2          echo-antiecho
SF             100.6127690 MHz
WDW                  QSINE
SSB                      2
LB                       0 Hz
GB                       0
```

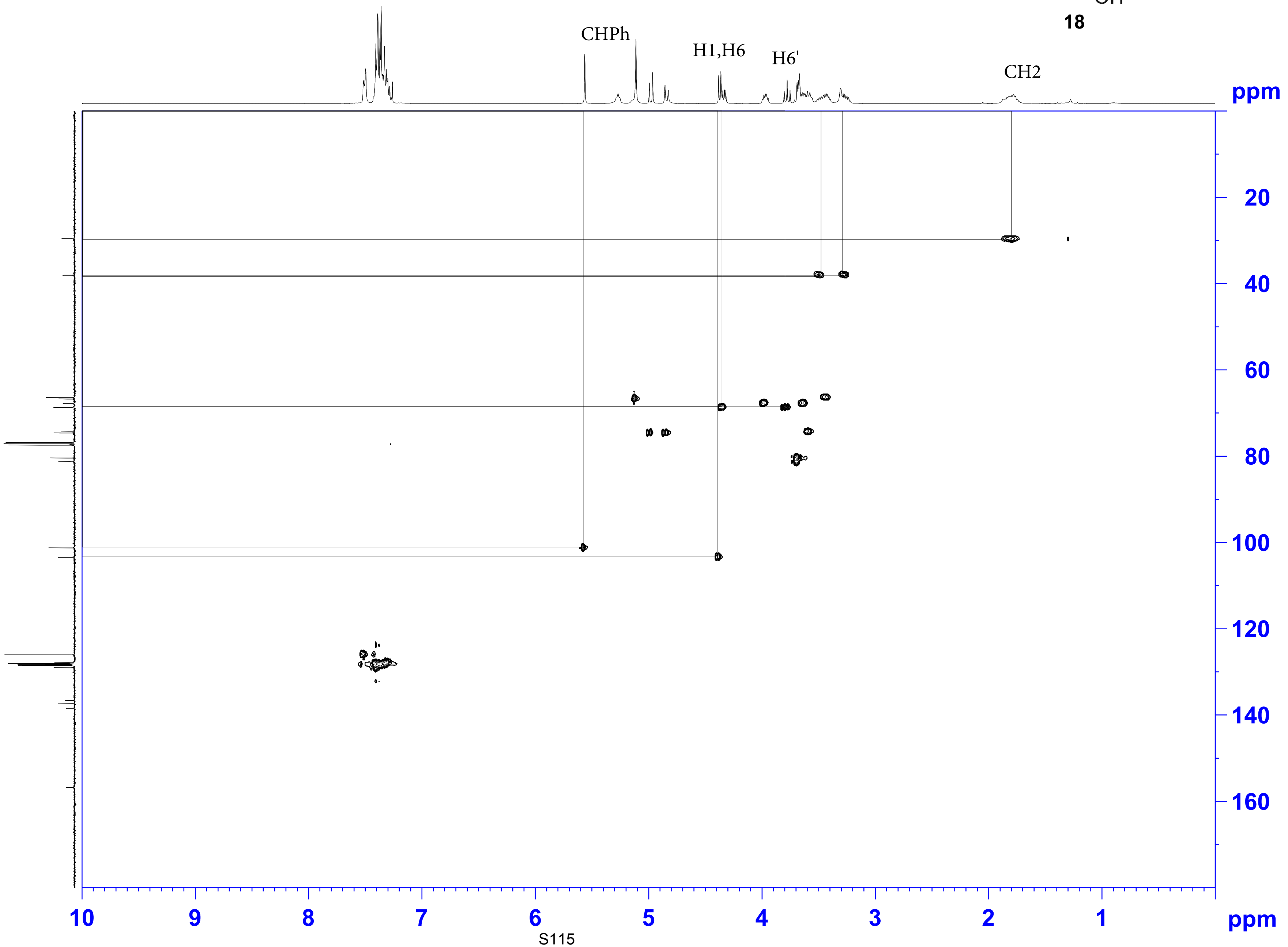

SSK-34-AKM-456-1H

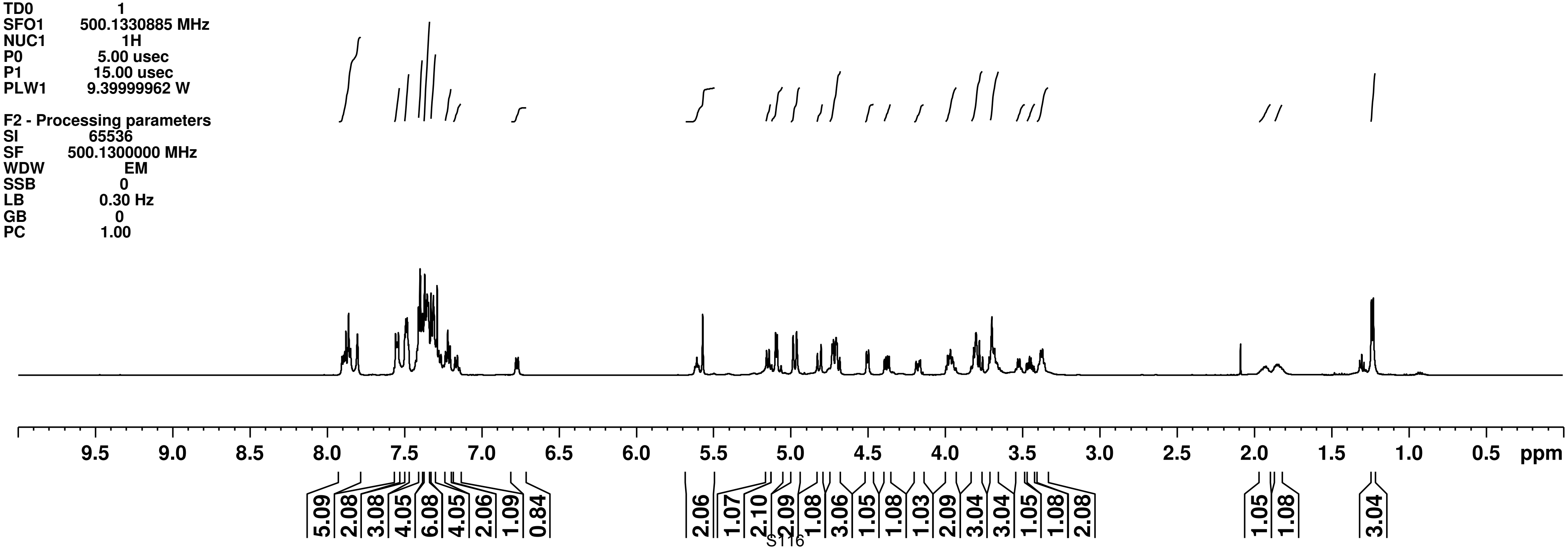

Current Data Parameters
NAME SSK-34-AKM-456-1H
EXPNO 1
PROCNO 1

F2 - Acquisition Parameters
Date_ 20240312
Time 12.03 h
INSTRUM spect
PROBHD Z124627_0008 (
PULPROG zg30
TD 65536
SOLVENT CDCl3
NS 12
DS 0
SWH 10000.000 Hz
FIDRES 0.305176 Hz
AQ 3.2767999 sec
RG 80.35
DW 50.000 usec
DE 6.50 usec
TE 295.6 K
D1 1.00000000 sec
TD0 1
SFO1 500.1330885 MHz
NUC1 1H
P0 5.00 usec
P1 15.00 usec
PLW1 9.39999962 W

F2 - Processing parameters
SI 65536
SF 500.1300000 MHz
WDW EM
SSB 0
LB 0.30 Hz
GB 0
PC 1.00

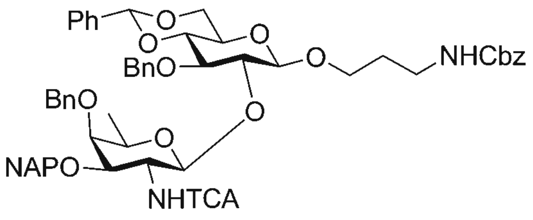

20

SSK-34-AKM-456-13C

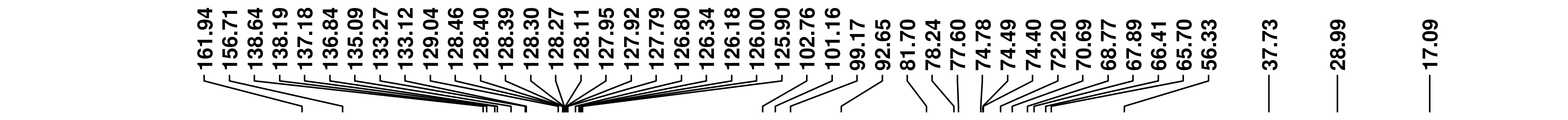

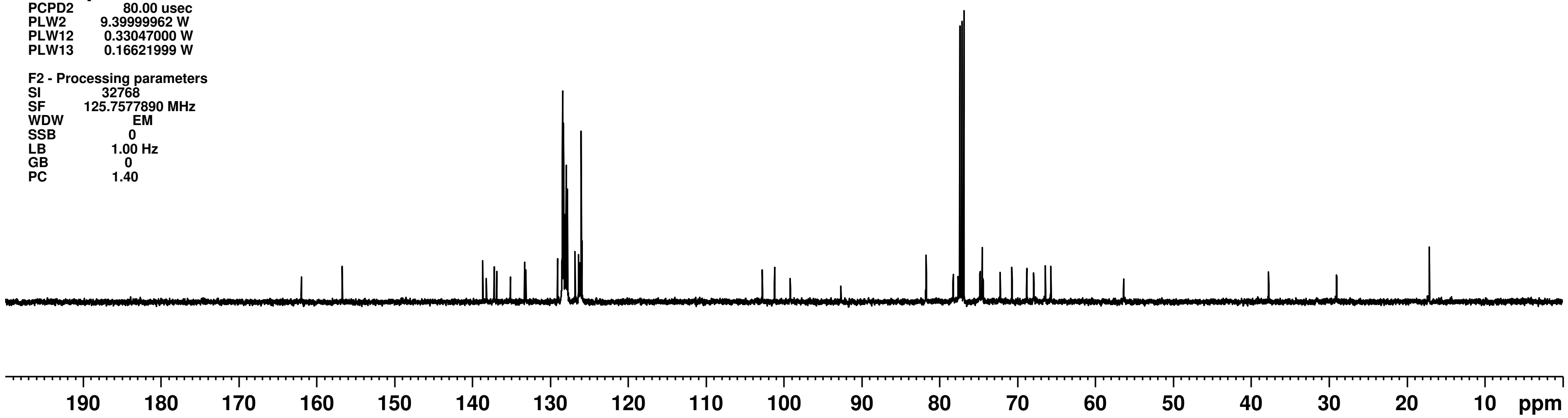

Current Data Parameters
NAME SSK-34-AKM-456-13C
EXPNO 2
PROCNO 1

F2 - Acquisition Parameters
Date_ 20240312
Time 12.09 h
INSTRUM spect
PROBHD Z124627_0008 (
PULPROG zgpg30
TD 65536
SOLVENT CDCl3
NS 156
DS 0
SWH 34722.223 Hz
FIDRES 1.059638 Hz
AQ 0.9437184 sec
RG 197.27
DW 14.400 usec
DE 6.50 usec
TE 295.8 K
D1 1.00000000 sec
D11 0.03000000 sec
TD0 1
SFO1 125.7721254 MHz
NUC1 13C
P0 3.33 usec
P1 10.00 usec
PLW1 50.00000000 W
SFO2 500.1320005 MHz
NUC2 1H
CPDPRG[2 waltz16
PCPD2 80.00 usec
PLW2 9.39999962 W
PLW12 0.33047000 W
PLW13 0.16621999 W

F2 - Processing parameters
SI 32768
SF 125.7577890 MHz
WDW EM
SSB 0
LB 1.00 Hz
GB 0
PC 1.40

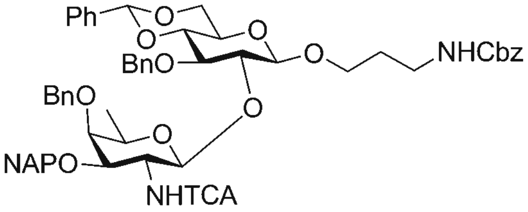

20

SSK-34-AKM-456-DEPT

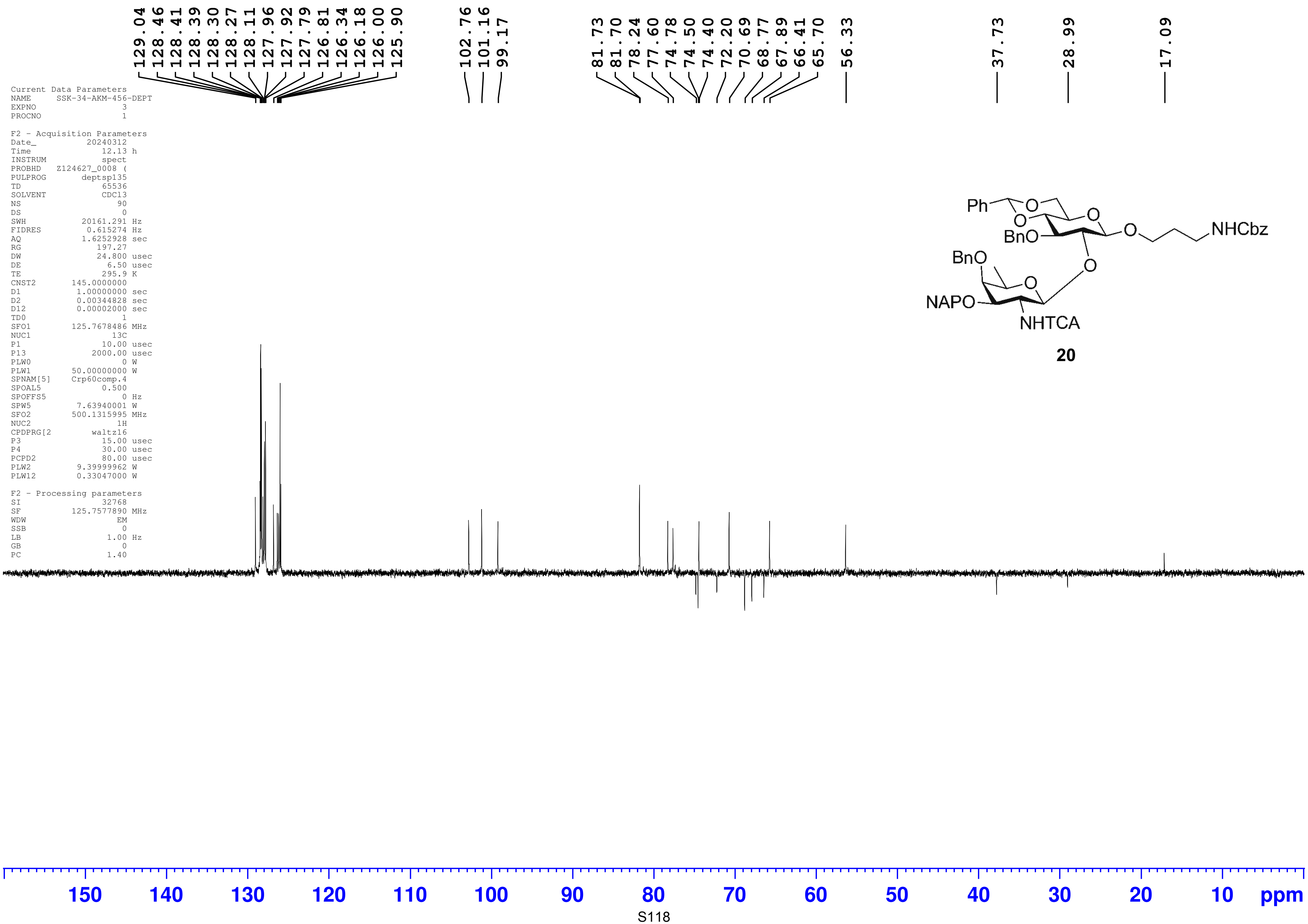


```
Current Data Parameters
NAME     SSK-34-AKM-456-DEPT
EXPNO                 3
PROCNO                1

F2 - Acquisition Parameters
Date_          20240312
Time              12.13 h
INSTRUM           spect
PROBHD   Z124627_0008 (
PULPROG       deptsp135
TD                65536
SOLVENT           CDCl3
NS                   90
DS                    0
SWH           20161.291 Hz
FIDRES         0.615274 Hz
AQ            1.6252928 sec
RG               197.27
DW               24.800 usec
DE                 6.50 usec
TE                295.9 K
CNST2       145.0000000
D1           1.00000000 sec
D2           0.00344828 sec
D12          0.00002000 sec
TD0                   1
SFO1        125.7678486 MHz
NUC1                13C
P1                10.00 usec
P13             2000.00 usec
PLW0                  0 W
PLW1        50.00000000 W
SPNAM[5]    Crp60comp.4
SPOAL5            0.500
SPOFFS5               0 Hz
SPW5         7.63940001 W
SFO2        500.1315995 MHz
NUC2                 1H
CPDPRG[2        waltz16
P3                15.00 usec
P4                30.00 usec
PCPD2             80.00 usec
PLW2         9.39999962 W
PLW12        0.33047000 W

F2 - Processing parameters
SI                32768
SF          125.7577890 MHz
WDW                  EM
SSB                   0
LB                 1.00 Hz
GB                    0
PC                 1.40
```

20

Current Data Parameters
NAME SSK-34-AKM-456-COSY
EXPNO 4
PROCNO 1

F2 - Acquisition Parameters
Date_ 20240312
Time 12.19 h
INSTRUM spect
PROBHD Z124627_0008 (
PULPROG cosygpppqf
TD 2048
SOLVENT CDCl3
NS 2
DS 0
SWH 3516.174 Hz
FIDRES 3.433764 Hz
AQ 0.2912256 sec
RG 61.42
DW 142.200 usec
DE 6.50 usec
TE 295.9 K
D0 0.00000300 sec
D1 1.00000000 sec
D11 0.03000000 sec
D12 0.00002000 sec
D13 0.00000400 sec
D16 0.00020000 sec
IN0 0.00028440 sec
TDav 1
SFO1 500.1317453 MHz
NUC1 1H
P0 15.00 usec
P1 15.00 usec
P17 2500.00 usec
PLW1 9.39999962 W
PLW10 2.34999990 W
GPNAM[1] SMSQ10.100
GPZ1 10.00 %
P16 1000.00 usec

F1 - Acquisition parameters
TD 128
SFO1 500.1317 MHz
FIDRES 54.940224 Hz
SW 7.030 ppm
FnMODE QF

F2 - Processing parameters
SI 1024
SF 500.1300000 MHz
WDW QSINE
SSB 0
LB 0 Hz
GB 0
PC 1.40

F1 - Processing parameters
SI 1024
MC2 QF
SF 500.1300000 MHz
WDW QSINE
SSB 0
LB 0 Hz
GB 0

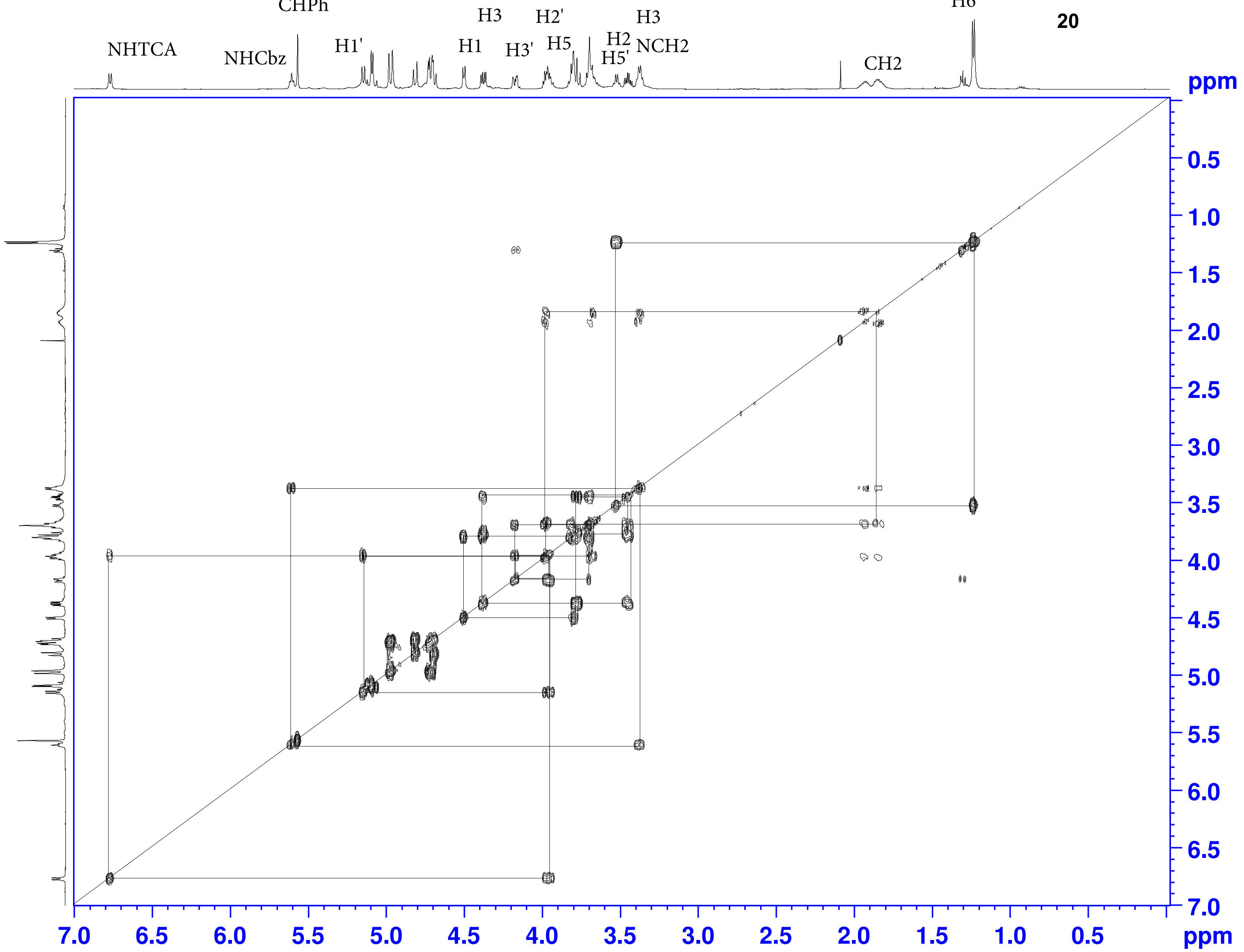

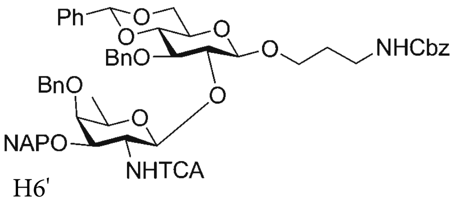

20

SSK-34-AKM-456-HSQC

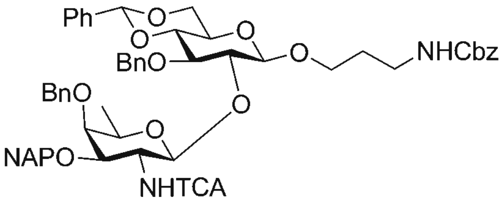

20

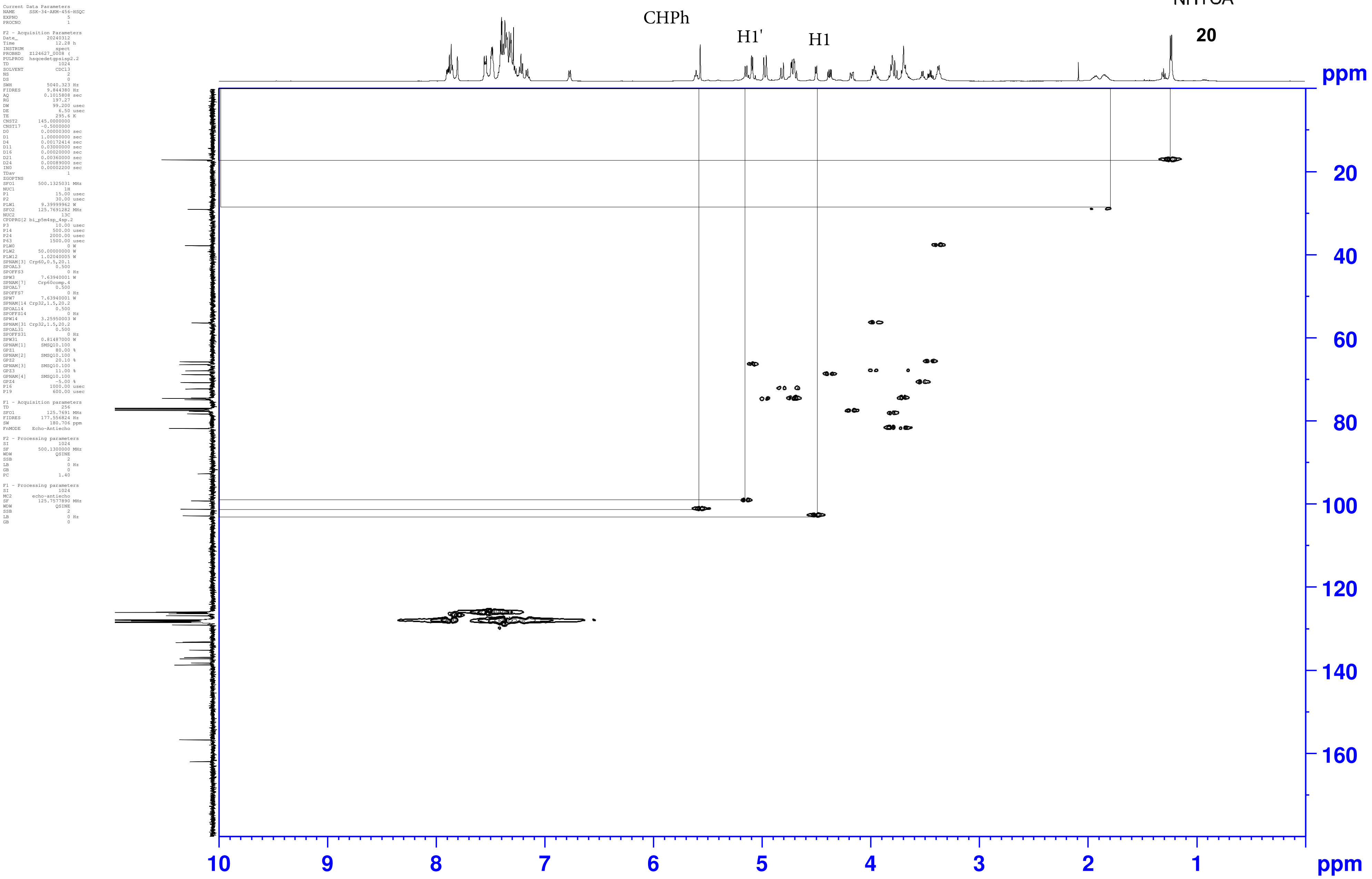

SSK-34-AKM-458-1H

Current Data Parameters
NAME SSK-34-AKM-458-1H
EXPNO 1
PROCNO 1

F2 - Acquisition Parameters
Date_ 20240313
Time 22.49 h
INSTRUM spect
PROBHD Z104450_0346 (
PULPROG zg30
TD 54274
SOLVENT CDCl3
NS 21
DS 0
SWH 8223.685 Hz
FIDRES 0.303043 Hz
AQ 3.2998593 sec
RG 71.8
DW 60.800 usec
DE 6.50 usec
TE 161.0 K
D1 1.00000000 sec
TD0 1
SFO1 400.1324710 MHz
NUC1 1H
P0 5.00 usec
P1 15.00 usec
PLW1 9.69999981 W

F2 - Processing parameters
SI 32768
SF 400.1300095 MHz
WDW EM
SSB 0
LB 0.30 Hz
GB 0
PC 1.00

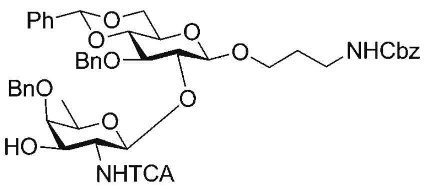

21

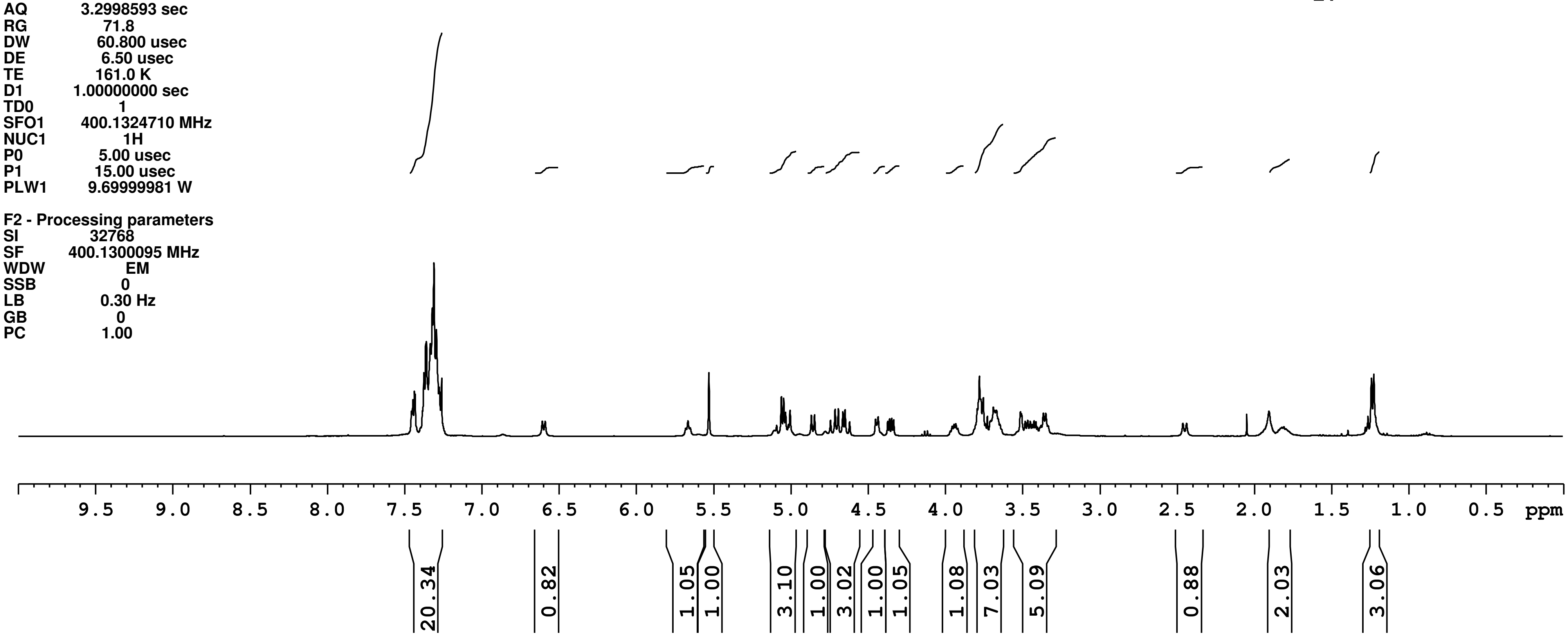

SSK-34-AKM-458-13C

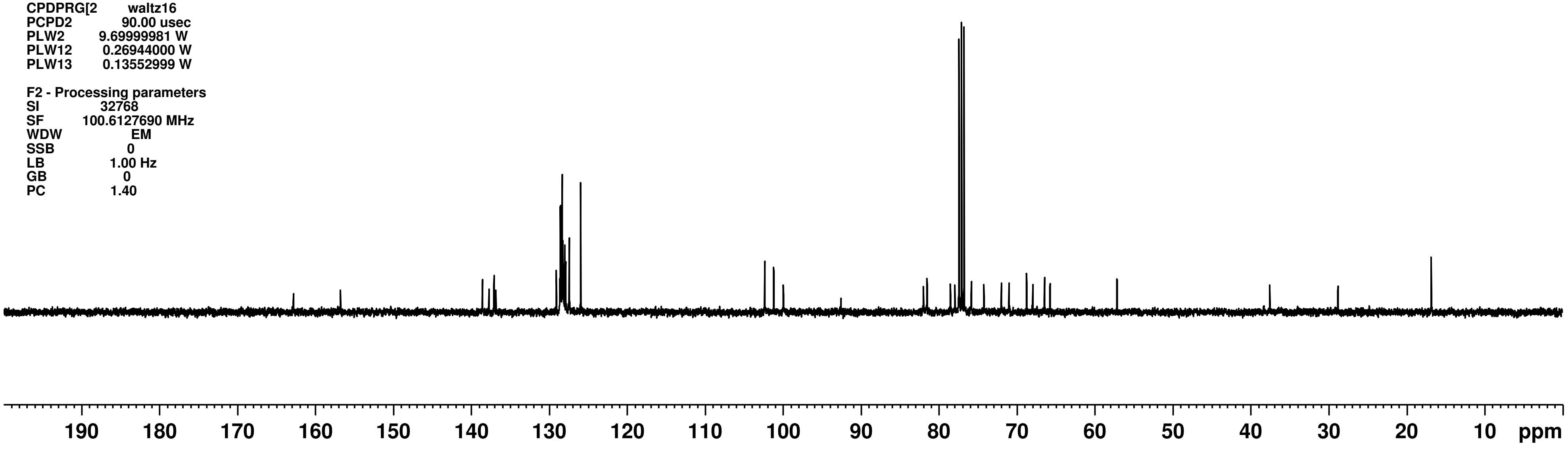

Current Data Parameters
NAME SSK-34-AKM-458-13C
EXPNO 2
PROCNO 1

F2 - Acquisition Parameters
Date_ 20240313
Time 22.55 h
INSTRUM spect
PROBHD Z104450_0346 (
PULPROG zgpg30
TD 65536
SOLVENT CDCl3
NS 150
DS 0
SWH 26041.666 Hz
FIDRES 0.794729 Hz
AQ 1.2582912 sec
RG 1030
DW 19.200 usec
DE 6.50 usec
TE 160.9 K
D1 1.00000000 sec
D11 0.03000000 sec
TD0 1
SFO1 100.6238364 MHz
NUC1 13C
P0 3.33 usec
P1 10.00 usec
PLW1 47.00000000 W
SFO2 400.1316005 MHz
NUC2 1H
CPDPRG[2 waltz16
PCPD2 90.00 usec
PLW2 9.69999981 W
PLW12 0.26944000 W
PLW13 0.13552999 W

F2 - Processing parameters
SI 32768
SF 100.6127690 MHz
WDW EM
SSB 0
LB 1.00 Hz
GB 0
PC 1.40

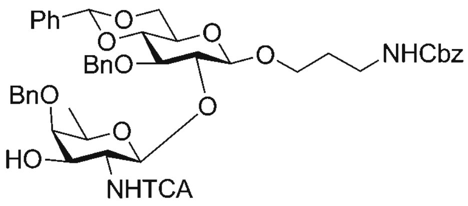

21

SSK-34-AKM-458-DEPT

Current Data Parameters
NAME SSK-34-AKM-458-DEPT
EXPNO 3
PROCNO 1

F2 - Acquisition Parameters
Date_ 20240313
Time 23.00 h
INSTRUM spect
PROBHD Z104450_0346 (
PULPROG dept135
TD 65536
SOLVENT CDCl3
NS 121
DS 0
SWH 27777.777 Hz
FIDRES 0.847710 Hz
AQ 1.1796480 sec
RG 203
DW 18.000 usec
DE 6.50 usec
TE 160.7 K
CNST2 145.0000000
D1 1.00000000 sec
D2 0.00344828 sec
D12 0.00002000 sec
TD0 1
SFO1 100.6242389 MHz
NUC1 13C
P1 10.00 usec
P2 20.00 usec
PLW1 47.00000000 W
SFO2 400.1316005 MHz
NUC2 1H
CPDPRG[2 waltz16
P3 15.00 usec
P4 30.00 usec
PCPD2 90.00 usec
PLW2 9.69999981 W
PLW12 0.26944000 W

F2 - Processing parameters
SI 32768
SF 100.6127690 MHz
WDW EM
SSB 0
LB 1.00 Hz
GB 0
PC 1.40

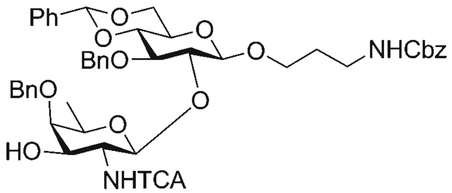

21

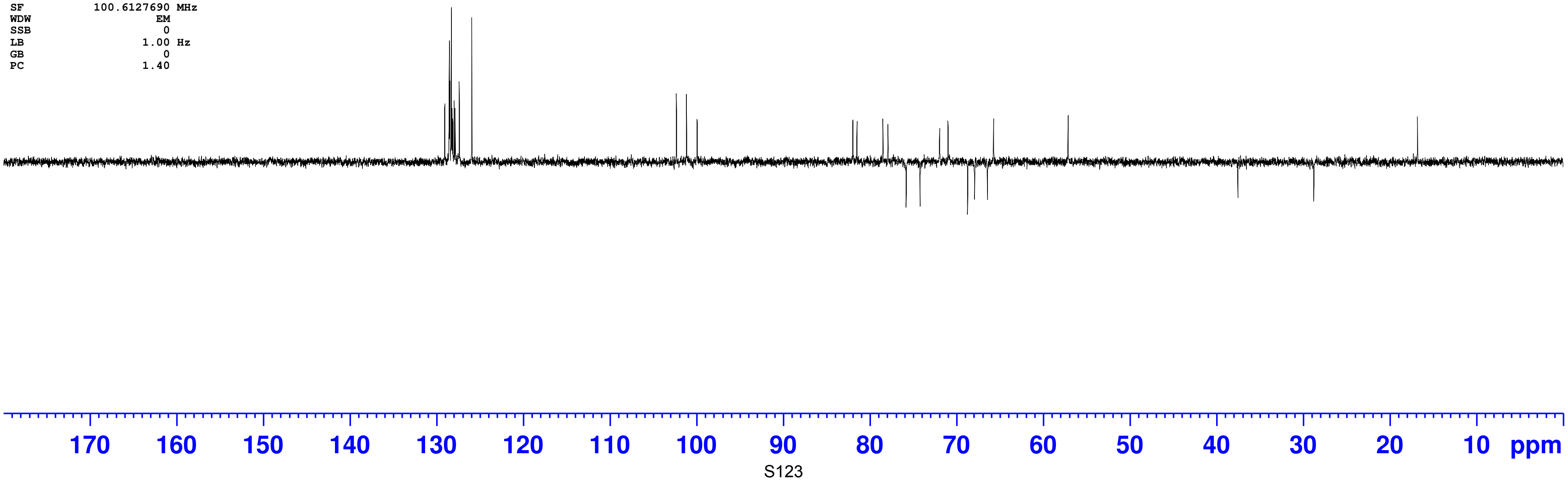

SSK-34-AKM-458-COSY

Current Data Parameters
NAME SSK-34-AKM-458-COSY
EXPNO 4
PROCNO 1

F2 - Acquisition Parameters
Date_ 20240313
Time 23.06 h
INSTRUM spect
PROBHD Z104450_0346 (
PULPROG cosygpqf
TD 2048
SOLVENT CDCl3
NS 2
DS 0
SWH 2808.989 Hz
FIDRES 2.743153 Hz
AQ 0.3645440 sec
RG 64
DW 178.000 usec
DE 6.50 usec
TE 160.6 K
D0 0.00000300 sec
D1 1.00000000 sec
D13 0.00000400 sec
D16 0.00020000 sec
IN0 0.00035600 sec
TDav 1
SFO1 400.1314008 MHz
NUC1 1H
P0 15.00 usec
P1 15.00 usec
PLW1 9.69999981 W
GPNAM[1] SINE.100
GPZ1 10.00 %
P16 1000.00 usec

F1 - Acquisition parameters
TD 128
SFO1 400.1314 MHz
FIDRES 43.890450 Hz
SW 7.020 ppm
FnMODE QF

F2 - Processing parameters
SI 1024
SF 400.1300000 MHz
WDW SINE
SSB 0
LB 0 Hz
GB 0
PC 1.40

F1 - Processing parameters
SI 1024
MC2 QF
SF 400.1300000 MHz
WDW SINE
SSB 0
LB 0 Hz
GB 0

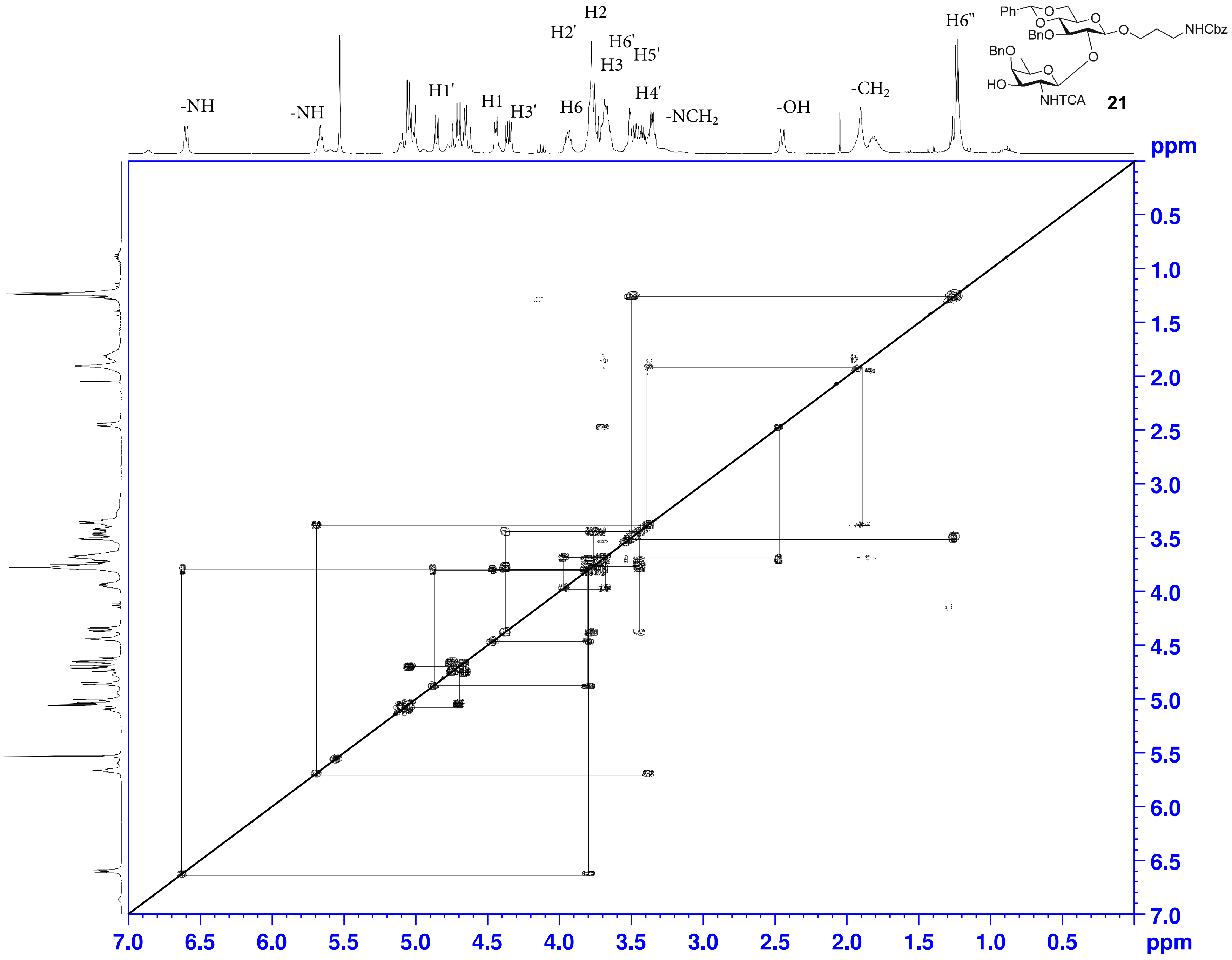

SSK-34-AKM-458-HSQC

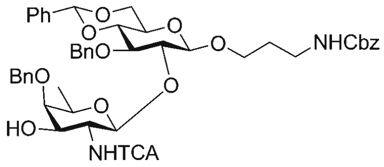

21

```
Current Data Parameters
NAME      SSK-34-AKM-458-HSQC
EXPNO                   5
PROCNO                  1

F2 - Acquisition Parameters
Date_           20240313
Time               23.12 h
INSTRUM            spect
PROBHD   Z104450_0346 (
PULPROG         hsqcetgp
TD                  2048
SOLVENT            CDCl3
NS                     2
DS                     0
SWH             4045.307 Hz
FIDRES          3.950495 Hz
AQ             0.2531328 sec
RG                  2050
DW               123.600 usec
DE                  6.50 usec
TE                 160.7 K
CNST2        145.0000000
D0            0.00000300 sec
D1            1.00000000 sec
D4            0.00172414 sec
D11           0.03000000 sec
D16           0.00020000 sec
IN0           0.00002760 sec
TDav                   1
ZGOPTNS
SFO1         400.1320038 MHz
NUC1                  1H
P1                 15.00 usec
P2                 30.00 usec
PLW1          9.69999981 W
SFO2         100.6218141 MHz
NUC2                 13C
CPDPRG[2            garp
P3                 10.00 usec
P4                 20.00 usec
PCPD2              80.00 usec
PLW2         47.00000000 W
PLW12         0.73438001 W
GPNAM[1]       SINE.100
GPZ1               80.00 %
GPNAM[2]       SINE.100
GPZ2               20.10 %
P16              1000.00 usec

F1 - Acquisition parameters
TD                   136
SFO1            100.6218 MHz
FIDRES        266.410919 Hz
SW               180.040 ppm
FnMODE     Echo-Antiecho

F2 - Processing parameters
SI                  2048
SF           400.1300000 MHz
WDW                QSINE
SSB                    2
LB                     0 Hz
GB                     0
PC                  1.40

F1 - Processing parameters
SI                  1024
MC2        echo-antiecho
SF           100.6127690 MHz
WDW                QSINE
SSB                    2
LB                     0 Hz
GB                     0
```

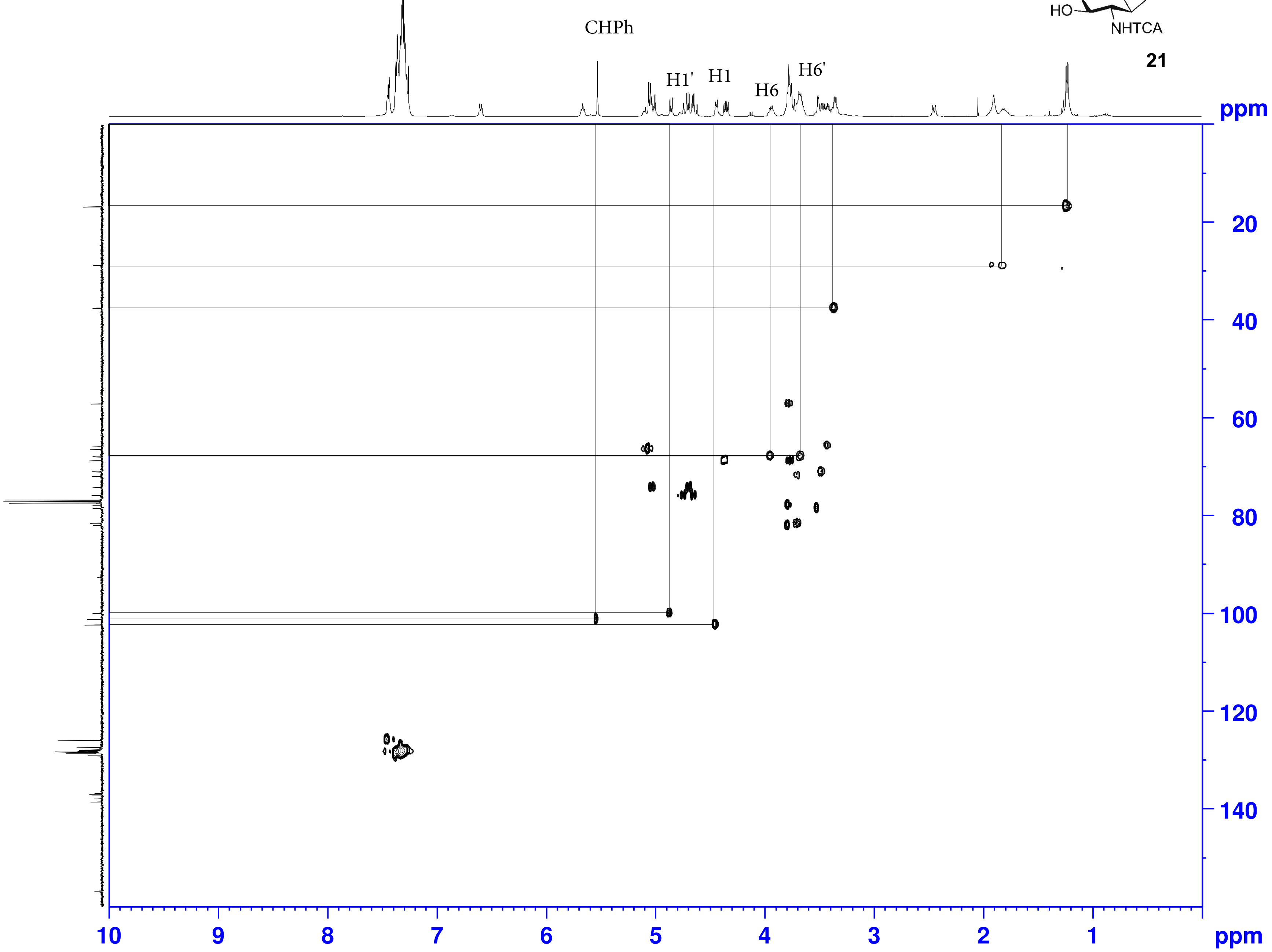

SSK-34-AKM-459-TRI-1H

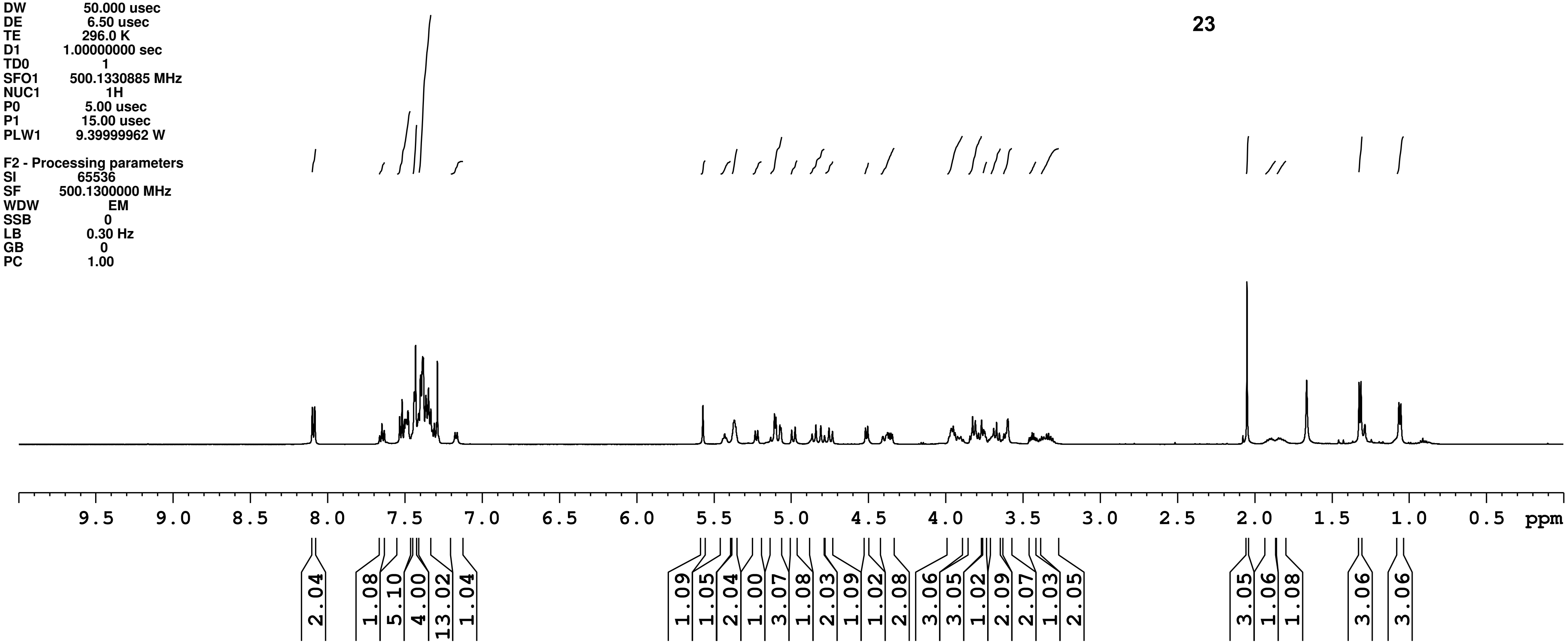

Current Data Parameters
NAME SSK-34-AKM-459-TRI-1H
EXPNO 1
PROCNO 1

F2 - Acquisition Parameters
Date_ 20240315
Time 20.08 h
INSTRUM spect
PROBHD Z124627_0008 (
PULPROG zg30
TD 65536
SOLVENT CDCl3
NS 16
DS 0
SWH 10000.000 Hz
FIDRES 0.305176 Hz
AQ 3.2767999 sec
RG 197.27
DW 50.000 usec
DE 6.50 usec
TE 296.0 K
D1 1.00000000 sec
TD0 1
SFO1 500.1330885 MHz
NUC1 1H
P0 5.00 usec
P1 15.00 usec
PLW1 9.39999962 W

F2 - Processing parameters
SI 65536
SF 500.1300000 MHz
WDW EM
SSB 0
LB 0.30 Hz
GB 0
PC 1.00

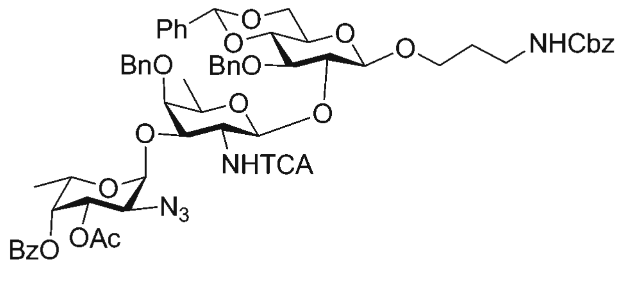

23

SSK-34-AKM-459-TRI-13C

Current Data Parameters
NAME SSK-34-AKM-459-TRI-13C
EXPNO 2
PROCNO 1

F2 - Acquisition Parameters
Date_ 20240315
Time 21.02 h
INSTRUM spect
PROBHD Z124627_0008 (
PULPROG zgpg30
TD 65536
SOLVENT CDCl3
NS 1024
DS 0
SWH 34722.223 Hz
FIDRES 1.059638 Hz
AQ 0.9437184 sec
RG 197.27
DW 14.400 usec
DE 6.50 usec
TE 295.8 K
D1 1.00000000 sec
D11 0.03000000 sec
TD0 1
SFO1 125.7721254 MHz
NUC1 13C
P0 3.33 usec
P1 10.00 usec
PLW1 50.00000000 W
SFO2 500.1320005 MHz
NUC2 1H
CPDPRG[2 waltz16
PCPD2 80.00 usec
PLW2 9.39999962 W
PLW12 0.33047000 W
PLW13 0.16621999 W

F2 - Processing parameters
SI 32768
SF 125.7577890 MHz
WDW EM
SSB 0
LB 1.00 Hz
GB 0
PC 1.40

23

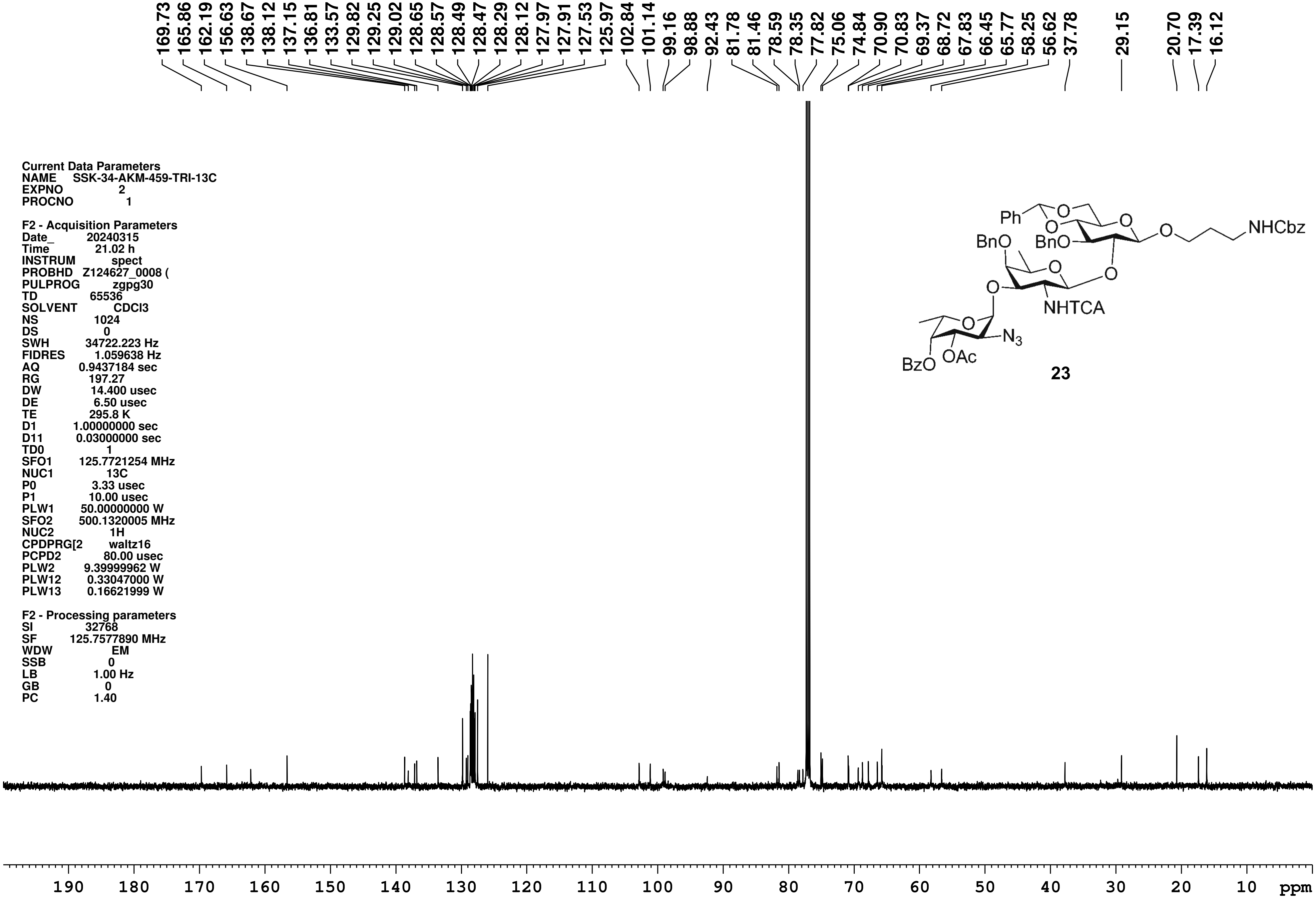

SSK-34-AKM-459-TRI-CH-DI

Current Data Parameters
NAME SSK-34-AKM-459-TRI-CH-DEPT
EXPNO 7
PROCNO 1

F2 - Acquisition Parameters
Date_ 20240316
Time 10.35 h
INSTRUM spect
PROBHD Z124627_0008 (
PULPROG deptsp135
TD 65536
SOLVENT CDCl3
NS 500
DS 0
SWH 20161.291 Hz
FIDRES 0.615274 Hz
AQ 1.6252928 sec
RG 197.27
DW 24.800 usec
DE 6.50 usec
TE 295.7 K
CNST2 145.0000000
D1 1.00000000 sec
D2 0.00344828 sec
D12 0.00002000 sec
TD0 1
SFO1 125.7678486 MHz
NUC1 13C
P1 10.00 usec
P13 2000.00 usec
PLW0 0 W
PLW1 50.00000000 W
SPNAM[5] Crp60comp.4
SPOAL5 0.500
SPOFFS5 0 Hz
SPW5 7.63940001 W
SFO2 500.1315995 MHz
NUC2 1H
CPDPRG[2 waltz16
P3 15.00 usec
P4 30.00 usec
PCPD2 80.00 usec
PLW2 9.39999962 W
PLW12 0.33047000 W

F2 - Processing parameters
SI 32768
SF 125.7577890 MHz
WDW EM
SSB 0
LB 1.00 Hz
GB 0
PC 1.40

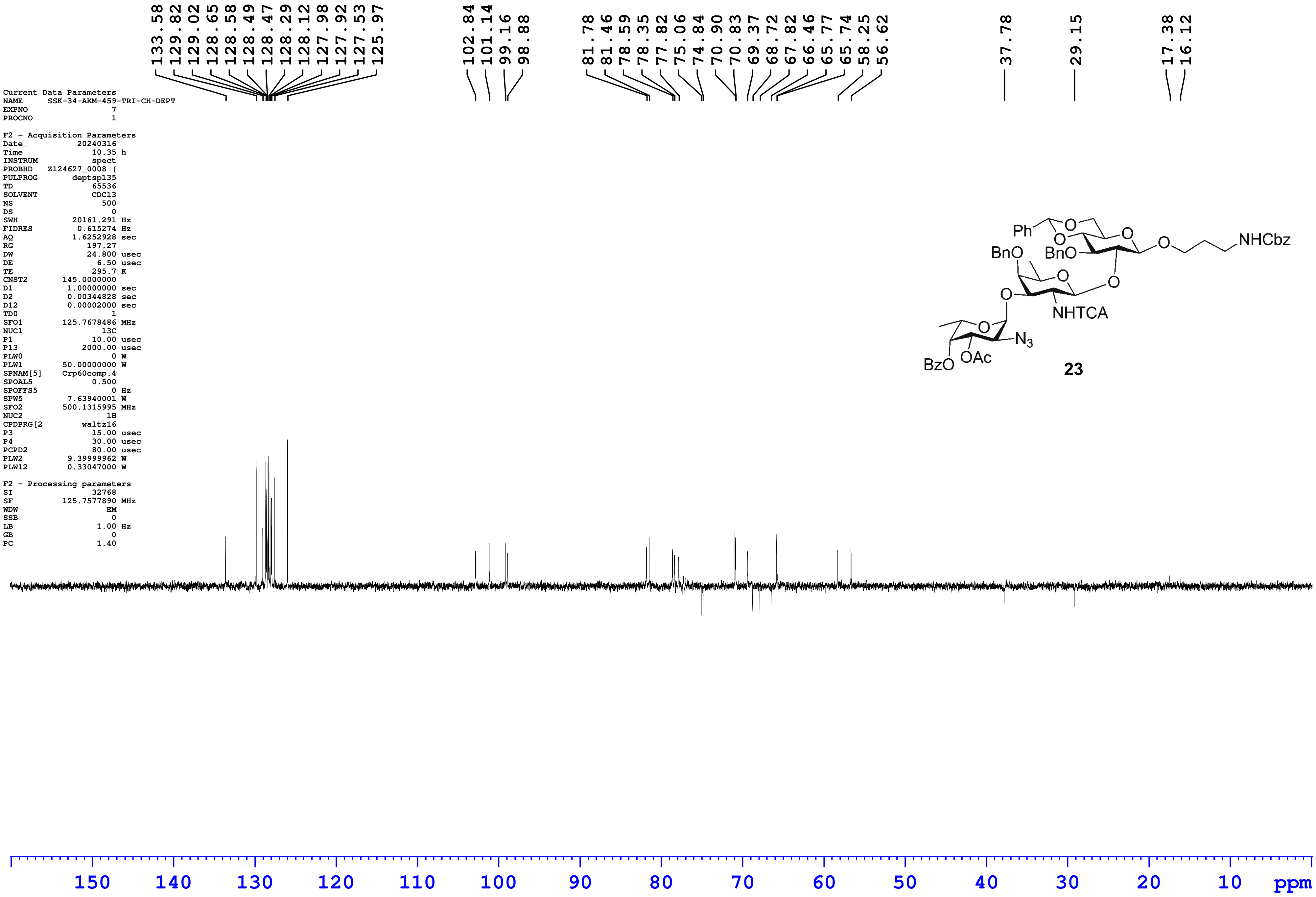

SSK-34-AKM-459-TRI-COSY-1

```
Current Data Parameters
NAME     SSK-34-AKM-459-TRI-COSY-1
EXPNO                     6
PROCNO                    1

F2 - Acquisition Parameters
Date_              20240316
Time                  10.13 h
INSTRUM               spect
PROBHD   Z124627_0008 (
PULPROG          cosygpppqf
TD                     2048
SOLVENT               CDCl3
NS                        4
DS                        0
SWH                4528.985 Hz
FIDRES             4.422837 Hz
AQ                0.2260992 sec
RG                    61.42
DW                  110.400 usec
DE                     6.50 usec
TE                    295.5 K
D0               0.00000300 sec
D1               1.00000000 sec
D11              0.03000000 sec
D12              0.00002000 sec
D13              0.00000400 sec
D16              0.00020000 sec
IN0              0.00022080 sec
TDav                      1
SFO1            500.1322571 MHz
NUC1                     1H
P0                    15.00 usec
P1                    15.00 usec
P17                 2500.00 usec
PLW1             9.39999962 W
PLW10            2.34999990 W
GPNAM[1]         SMSQ10.100
GPZ1                  10.00 %
P16                 1000.00 usec

F1 - Acquisition parameters
TD                      128
SFO1               500.1323 MHz
FIDRES            70.765396 Hz
SW                    9.056 ppm
FnMODE                   QF

F2 - Processing parameters
SI                     1024
SF              500.1300000 MHz
WDW                   QSINE
SSB                       0
LB                        0 Hz
GB                        0
PC                     1.40

F1 - Processing parameters
SI                     1024
MC2                      QF
SF              500.1300000 MHz
WDW                   QSINE
SSB                       0
LB                        0 Hz
GB                        0
```

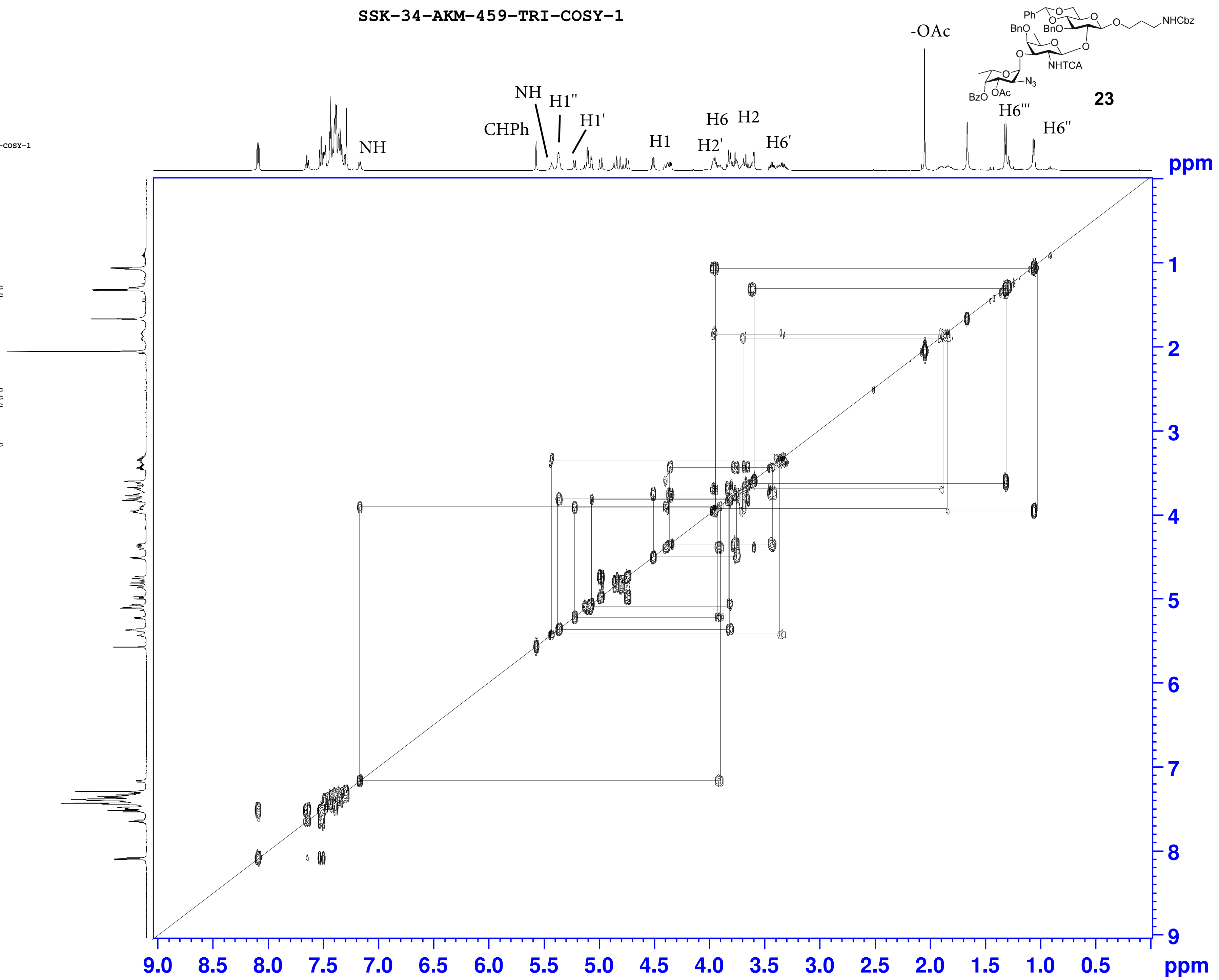

SSK-34-AKM-459-TRI-HSQC

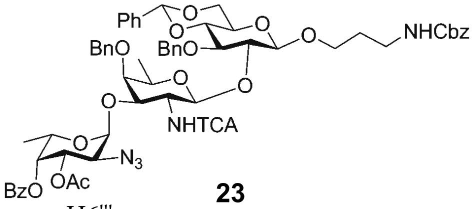

23

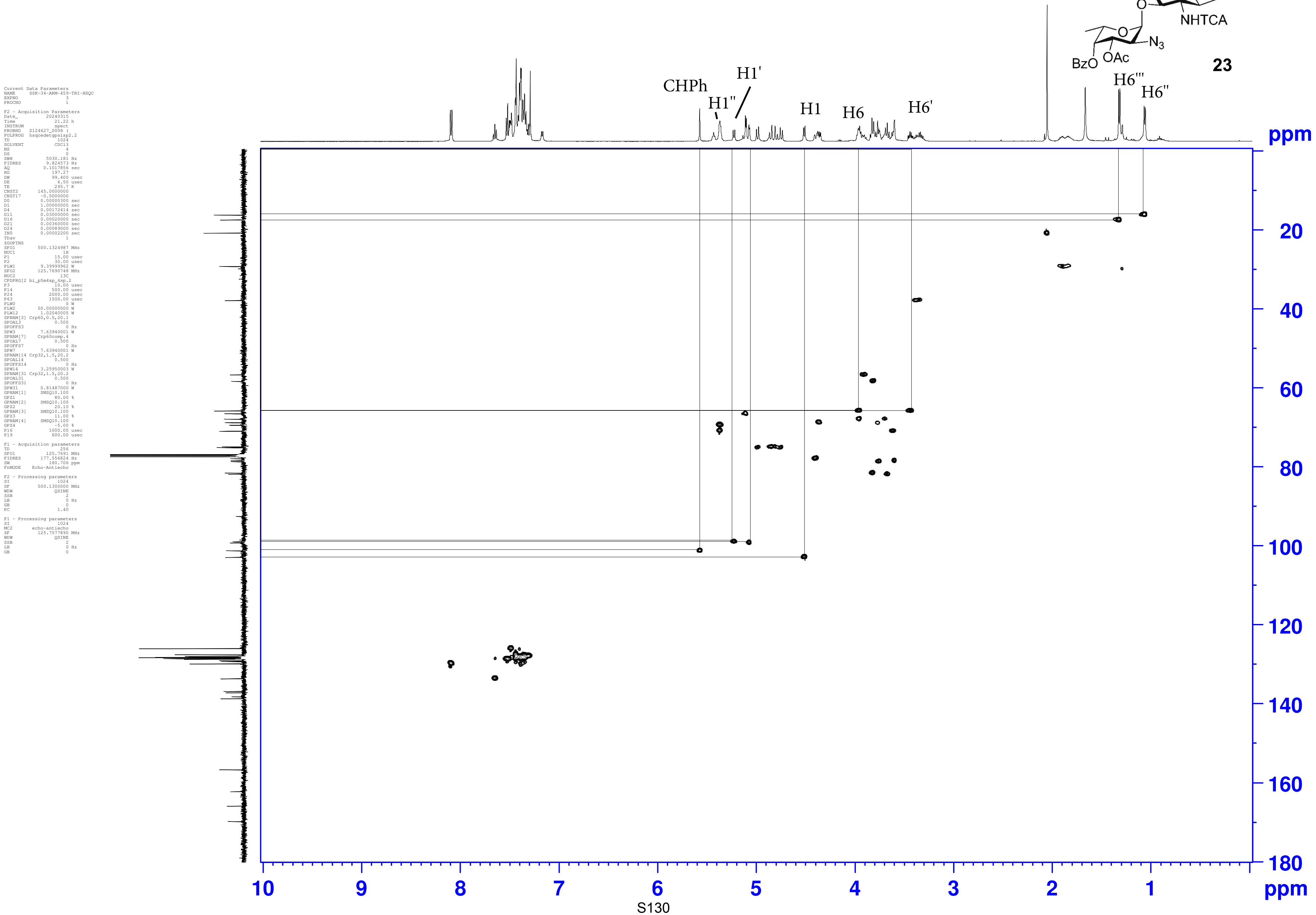

SSK-34-AKM-459-TRI-CH-CH-COUPLING

```
Current Data Parameters
NAME      SSK-34-AKM-459-TRI-CH-CH-COUPLING
EXPNO                    8
PROCNO                   1

F2 - Acquisition Parameters
Date_            20240317
Time                20.03 h
INSTRUM             spect
PROBHD   Z124627_0008 (
PULPROG           zggd30
TD                 65536
SOLVENT            CDCl3
NS                  7493
DS                     0
SWH            32894.738 Hz
FIDRES          1.003868 Hz
AQ             0.9961472 sec
RG                197.27
DW                15.200 usec
DE                  6.50 usec
TE                 295.6 K
D1            1.00000000 sec
D11           0.03000000 sec
TD0                    1
SFO1         125.7721254 MHz
NUC1                 13C
P0                  3.33 usec
P1                 10.00 usec
PLW1         50.00000000 W
SFO2         500.1320005 MHz
NUC2                  1H
CPDPRG[2         waltz16
PCPD2              80.00 usec
PLW2          9.39999962 W
PLW12         0.33047000 W

F2 - Processing parameters
SI                 32768
SF           125.7577681 MHz
WDW                   EM
SSB                    0
LB                  1.00 Hz
GB                     0
PC                  1.40
```

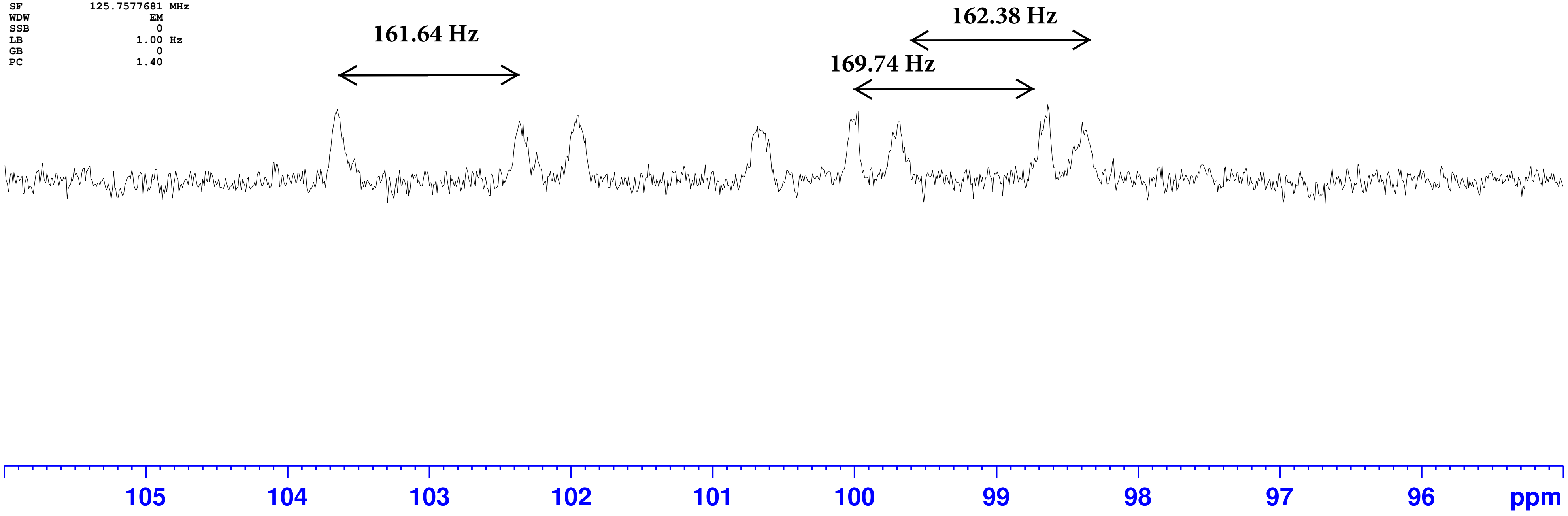

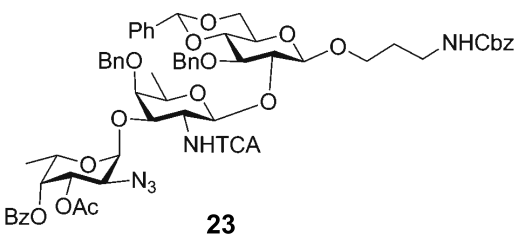

23

SSK-34-AKM-3rd-Tri-14-1H

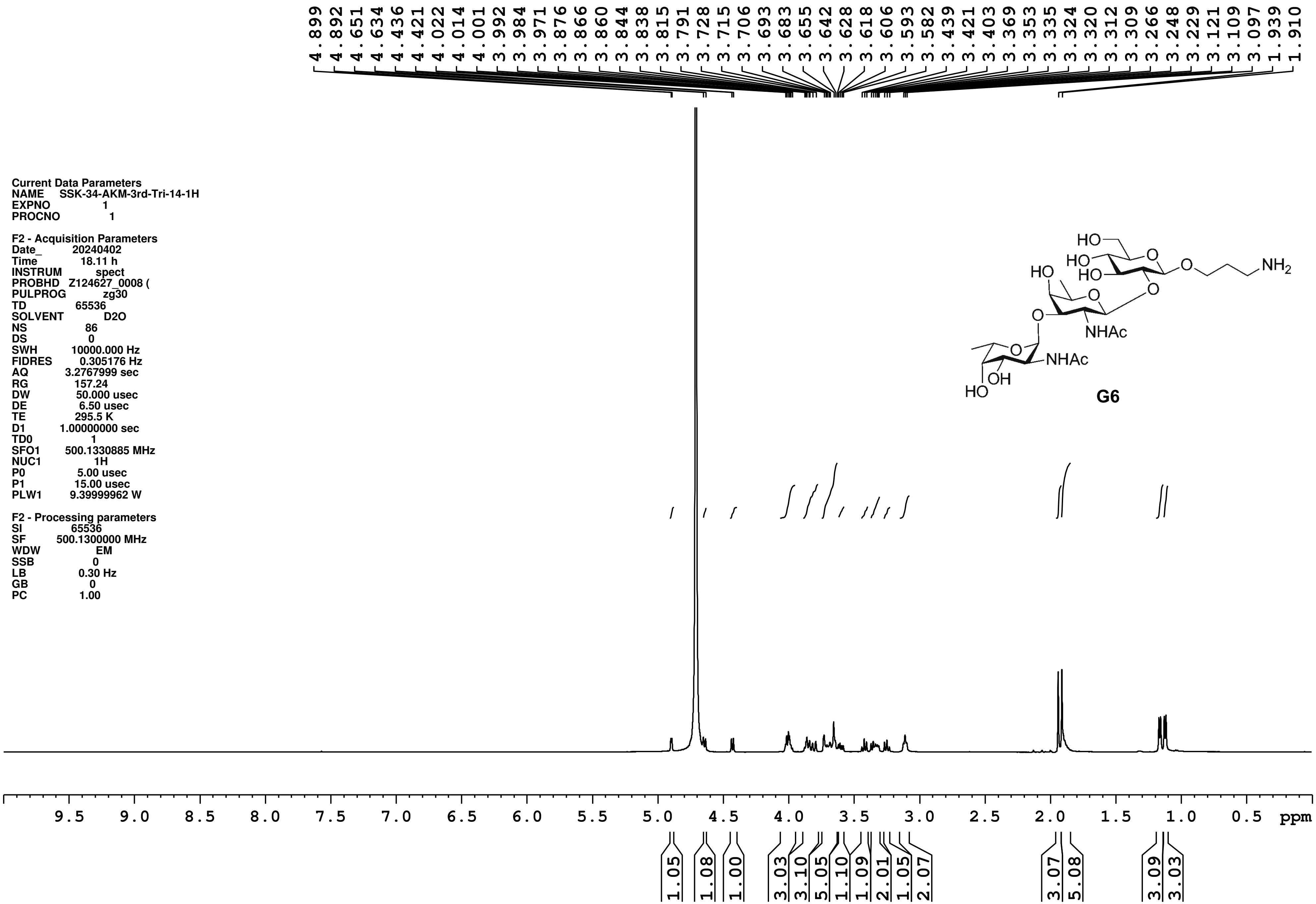

Current Data Parameters
NAME SSK-34-AKM-3rd-Tri-14-1H
EXPNO 1
PROCNO 1

F2 - Acquisition Parameters
Date_ 20240402
Time 18.11 h
INSTRUM spect
PROBHD Z124627_0008 (
PULPROG zg30
TD 65536
SOLVENT D2O
NS 86
DS 0
SWH 10000.000 Hz
FIDRES 0.305176 Hz
AQ 3.2767999 sec
RG 157.24
DW 50.000 usec
DE 6.50 usec
TE 295.5 K
D1 1.00000000 sec
TD0 1
SFO1 500.1330885 MHz
NUC1 1H
P0 5.00 usec
P1 15.00 usec
PLW1 9.39999962 W

F2 - Processing parameters
SI 65536
SF 500.1300000 MHz
WDW EM
SSB 0
LB 0.30 Hz
GB 0
PC 1.00

G6

SSK-34-AKM-3rd-Tri-14-13C-1

Current Data Parameters
NAME SSK-34-AKM-3rd-Tri-14-13C-1
EXPNO 3
PROCNO 1

F2 - Acquisition Parameters
Date_ 20240402
Time 19.08 h
INSTRUM spect
PROBHD Z124627_0008 (
PULPROG zgpg30
TD 65536
SOLVENT D2O
NS 1500
DS 0
SWH 34722.223 Hz
FIDRES 1.059638 Hz
AQ 0.9437184 sec
RG 197.27
DW 14.400 usec
DE 6.50 usec
TE 295.8 K
D1 1.00000000 sec
D11 0.03000000 sec
TD0 1
SFO1 125.7721254 MHz
NUC1 13C
P0 3.33 usec
P1 10.00 usec
PLW1 50.00000000 W
SFO2 500.1320005 MHz
NUC2 1H
CPDPRG[2 waltz16
PCPD2 80.00 usec
PLW2 9.39999962 W
PLW12 0.33047000 W
PLW13 0.16621999 W

F2 - Processing parameters
SI 32768
SF 125.7577890 MHz
WDW EM
SSB 0
LB 1.00 Hz
GB 0
PC 1.40

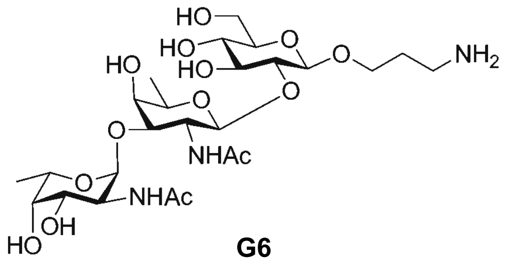

G6

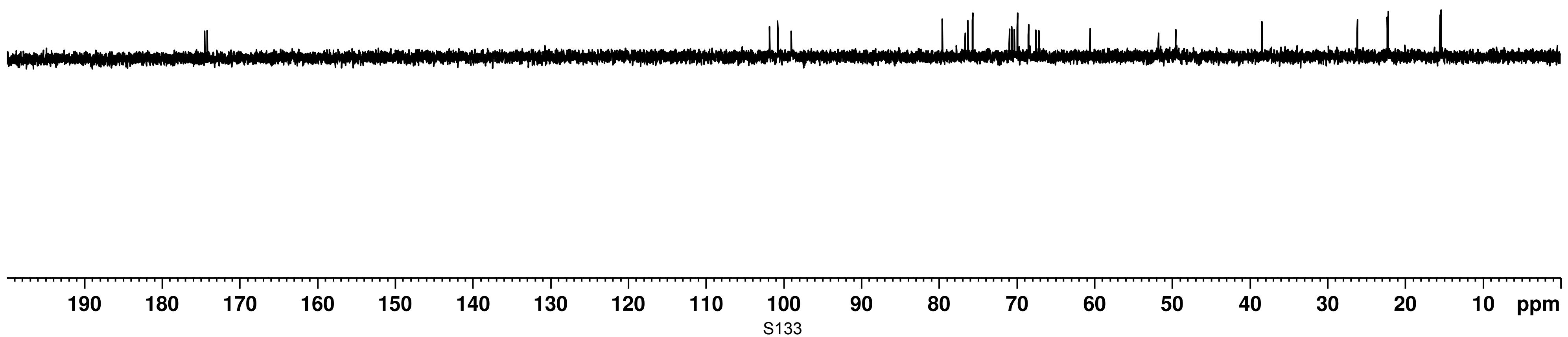

SSK-34-AKM-3rd-Tri-14-DEPT

101.78, 100.70, 98.97, 79.55, 76.57, 76.23, 75.62, 70.88, 70.57, 70.27, 69.85, 68.42, 67.48, 67.07, 60.51, 51.70, 49.48, 38.39, 26.10, 22.24, 22.12, 15.45, 15.34

```
Current Data Parameters
NAME     SSK-34-AKM-3rd-Tri-14-DEPT
EXPNO                 2
PROCNO                1

F2 - Acquisition Parameters
Date_          20240402
Time              23.44 h
INSTRUM           spect
PROBHD   Z104450_0346 (
PULPROG         dept135
TD                65536
SOLVENT             D2O
NS                 1024
DS                    0
SWH           27777.777 Hz
FIDRES         0.847710 Hz
AQ            1.1796480 sec
RG                 2050
DW               18.000 usec
DE                 6.50 usec
TE                686.4 K
CNST2       145.0000000
D1           1.00000000 sec
D2           0.00344828 sec
D12          0.00002000 sec
TD0                   1
SFO1        100.6242389 MHz
NUC1                13C
P1                10.00 usec
P2                20.00 usec
PLW1        47.00000000 W
SFO2        400.1316005 MHz
NUC2                 1H
CPDPRG[2        waltz16
P3                15.00 usec
P4                30.00 usec
PCPD2             90.00 usec
PLW2         9.69999981 W
PLW12        0.26944000 W

F2 - Processing parameters
SI                32768
SF          100.6127690 MHz
WDW                  EM
SSB                   0
LB                 1.00 Hz
GB                    0
PC                 1.40
```

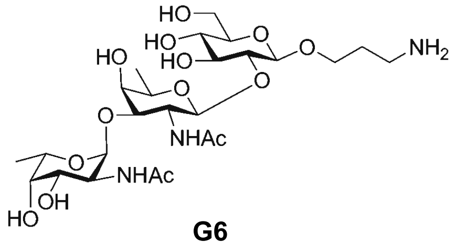

G6

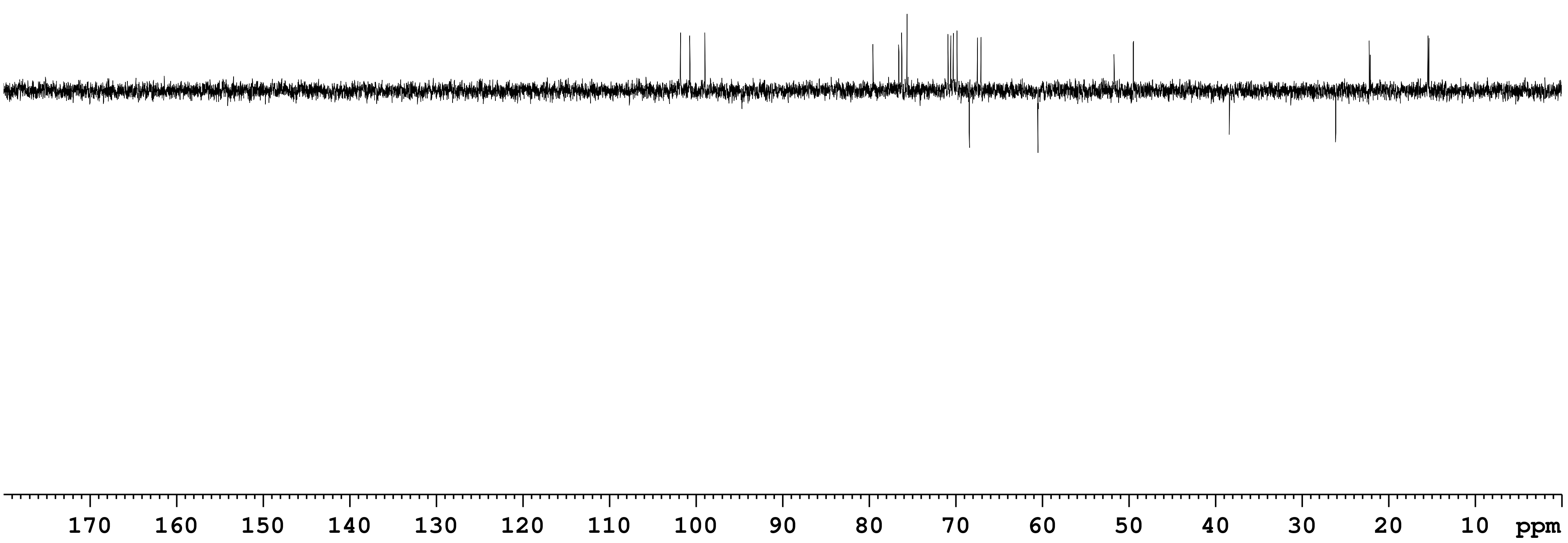

SSK-34-AKM-3rd-Tri-14-HSQC

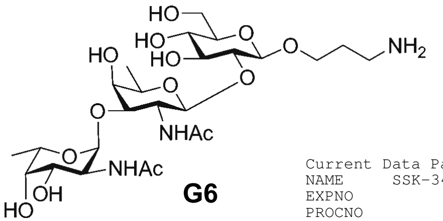

G6

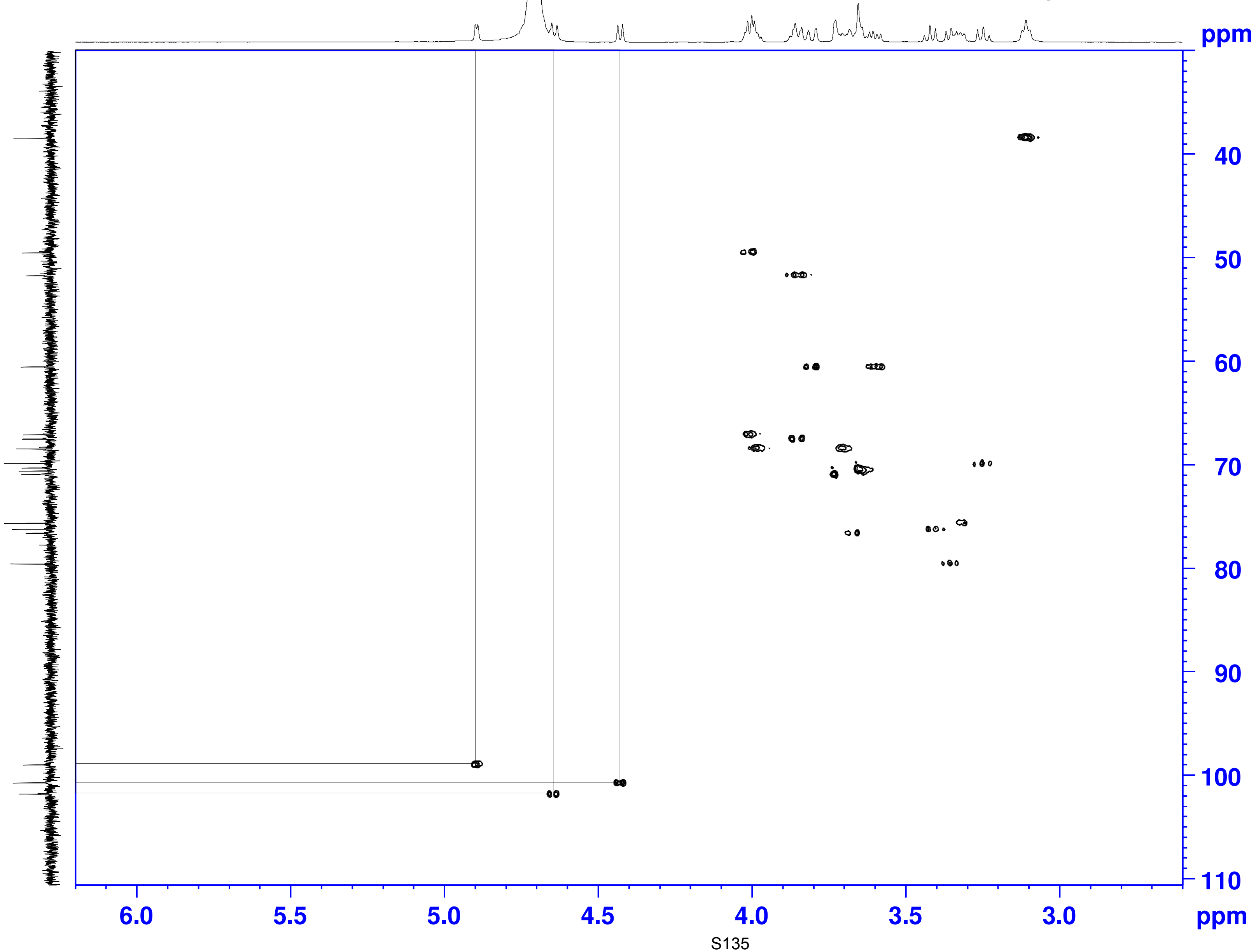

Current Data Parameters
NAME SSK-34-AKM-3rd-Tri-14-HSQC
EXPNO 1
PROCNO 1

F2 - Acquisition Parameters
Date_ 20240403
Time 2.48 h
INSTRUM spect
PROBHD Z104450_0346 (
PULPROG hsqcetgp
TD 2048
SOLVENT D2O
NS 8
DS 0
SWH 2857.143 Hz
FIDRES 2.790179 Hz
AQ 0.3584000 sec
RG 2050
DW 175.000 usec
DE 6.50 usec
TE 679.2 K
CNST2 145.0000000
D0 0.00000300 sec
D1 1.00000000 sec
D4 0.00172414 sec
D11 0.03000000 sec
D16 0.00020000 sec
IN0 0.00004500 sec
TDav 1
ZGOPTNS
SFO1 400.1324708 MHz
NUC1 1H
P1 15.00 usec
P2 30.00 usec
PLW1 9.69999981 W
SFO2 100.6183419 MHz
NUC2 13C
CPDPRG[2 garp
P3 10.00 usec
P4 20.00 usec
PCPD2 80.00 usec
PLW2 47.00000000 W
PLW12 0.73438001 W
GPNAM[1] SINE.100
GPZ1 80.00 %
GPNAM[2] SINE.100
GPZ2 20.10 %
P16 1000.00 usec

F1 - Acquisition parameters
TD 256
SFO1 100.6183 MHz
FIDRES 86.805557 Hz
SW 110.428 ppm
FnMODE Echo-Antiecho

F2 - Processing parameters
SI 2048
SF 400.1300000 MHz
WDW QSINE
SSB 2
LB 0 Hz
GB 0
PC 1.40

F1 - Processing parameters
SI 1024
MC2 echo-antiecho
SF 100.6127690 MHz
WDW QSINE
SSB 2
LB 0 Hz
GB 0

SSK-34-AKM-442-DS-ACCEPTOR-1H

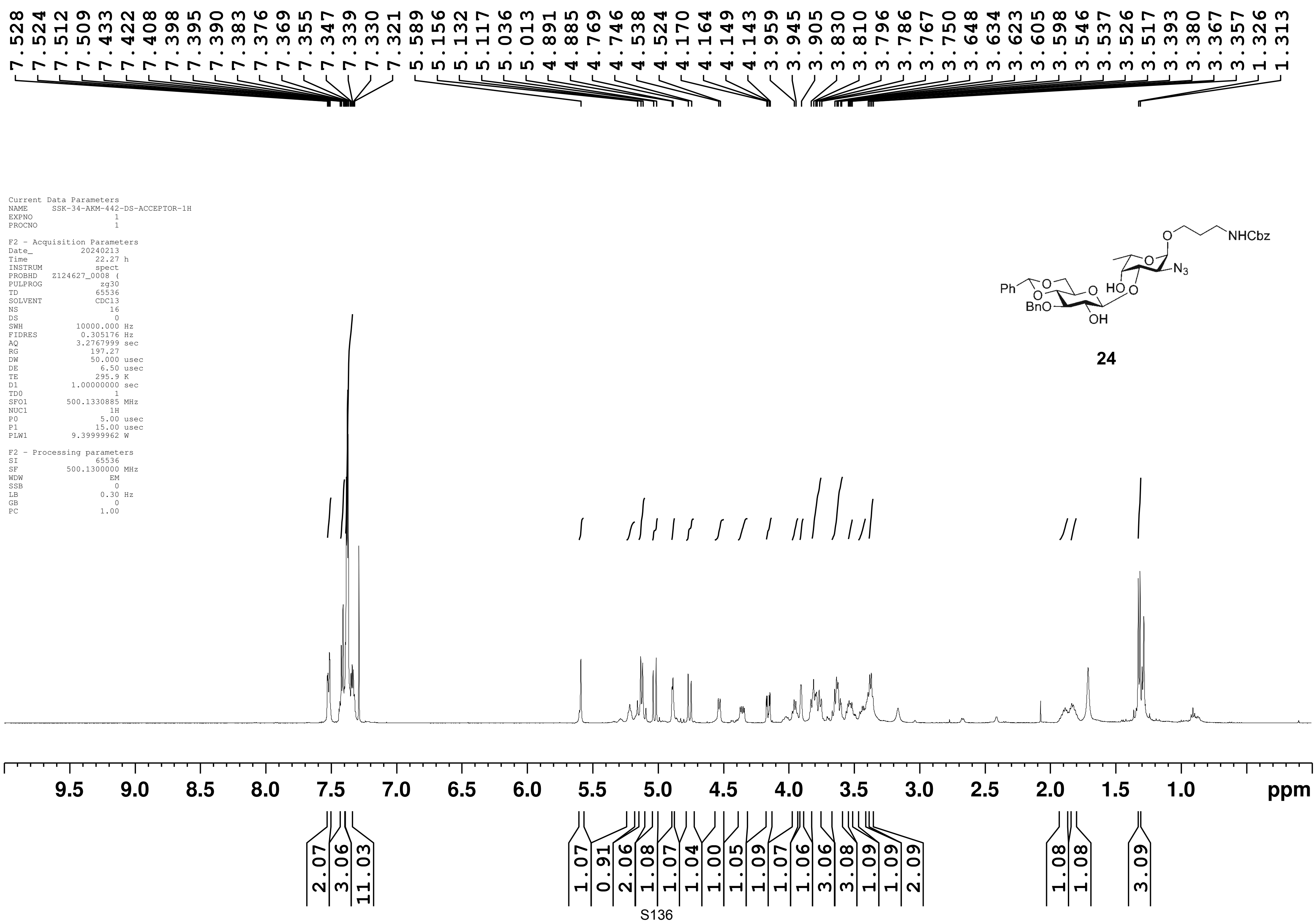


```
Current Data Parameters
NAME     SSK-34-AKM-442-DS-ACCEPTOR-1H
EXPNO                 1
PROCNO                1

F2 - Acquisition Parameters
Date_          20240213
Time              22.27 h
INSTRUM           spect
PROBHD   Z124627_0008 (
PULPROG            zg30
TD                65536
SOLVENT           CDC13
NS                   16
DS                    0
SWH           10000.000 Hz
FIDRES         0.305176 Hz
AQ            3.2767999 sec
RG               197.27
DW               50.000 usec
DE                 6.50 usec
TE                295.9 K
D1           1.00000000 sec
TD0                   1
SFO1        500.1330885 MHz
NUC1                 1H
P0                 5.00 usec
P1                15.00 usec
PLW1         9.39999962 W

F2 - Processing parameters
SI                65536
SF          500.1300000 MHz
WDW                  EM
SSB                   0
LB                 0.30 Hz
GB                    0
PC                 1.00
```


24

SSK-34-AKM-442-DS-ACCEPTOR-13C

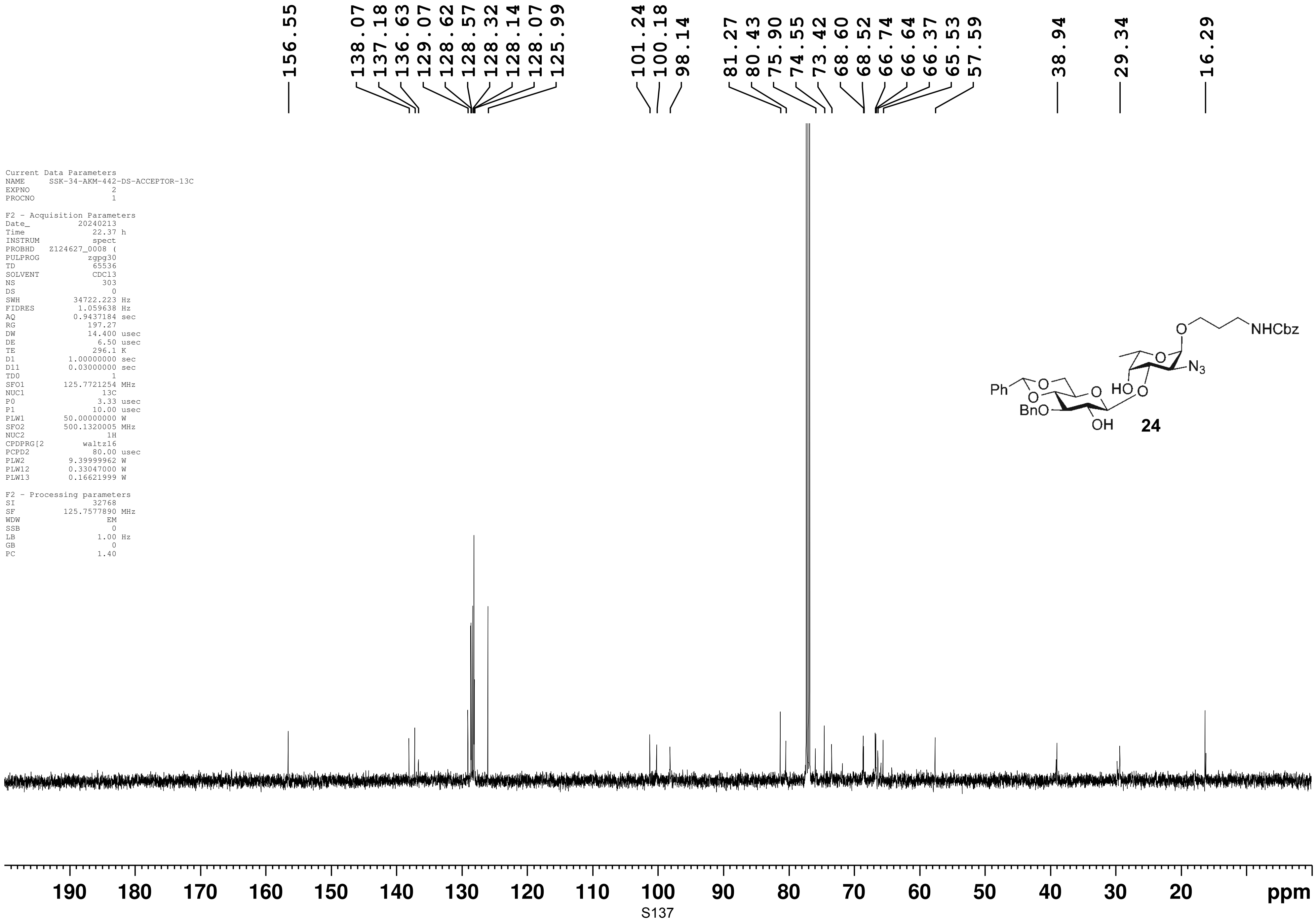

24

SSK-34-AKM-442-DEPT

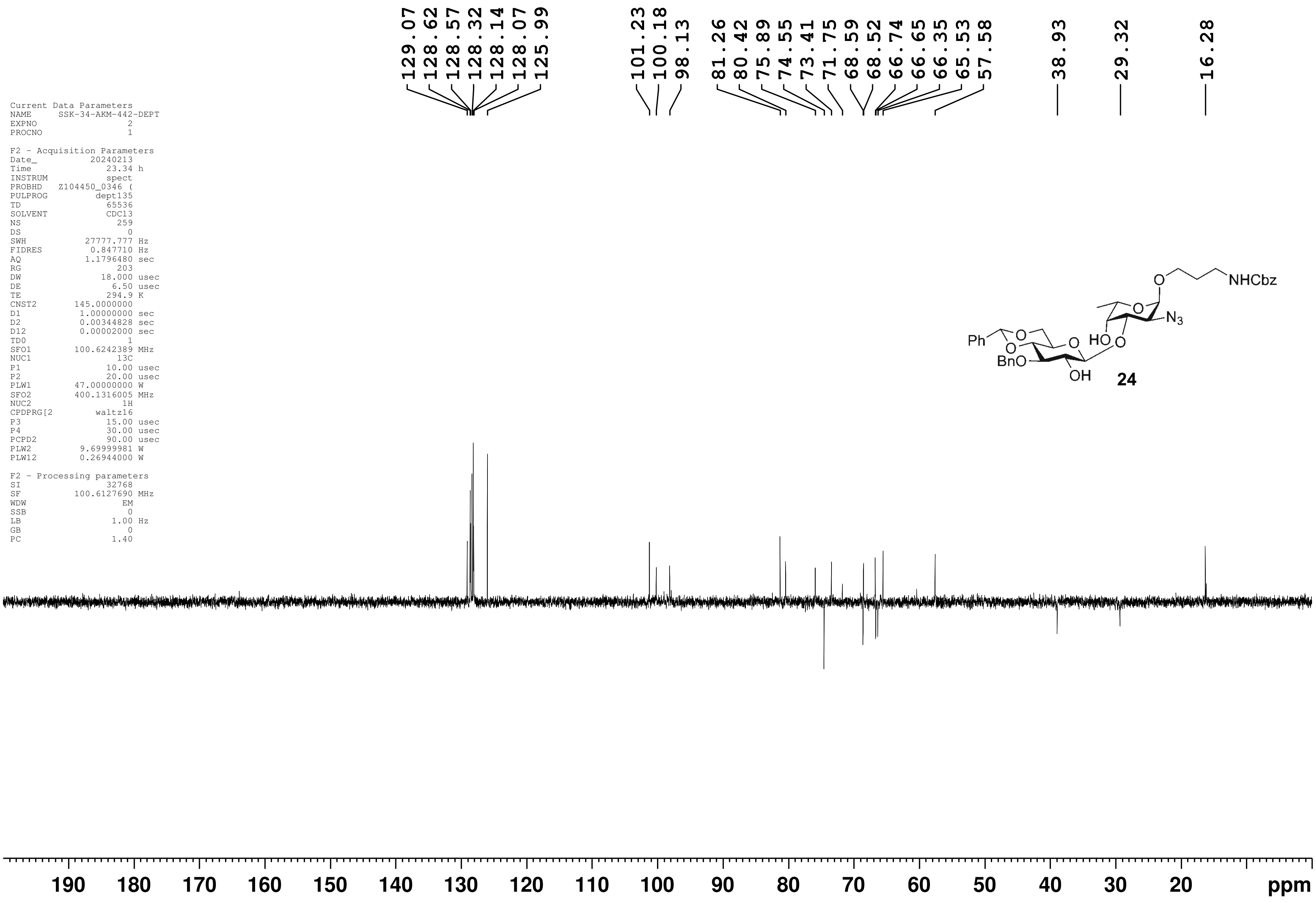

24

SSK-34-AKM-442-DS-ACCEPTOR-COSY

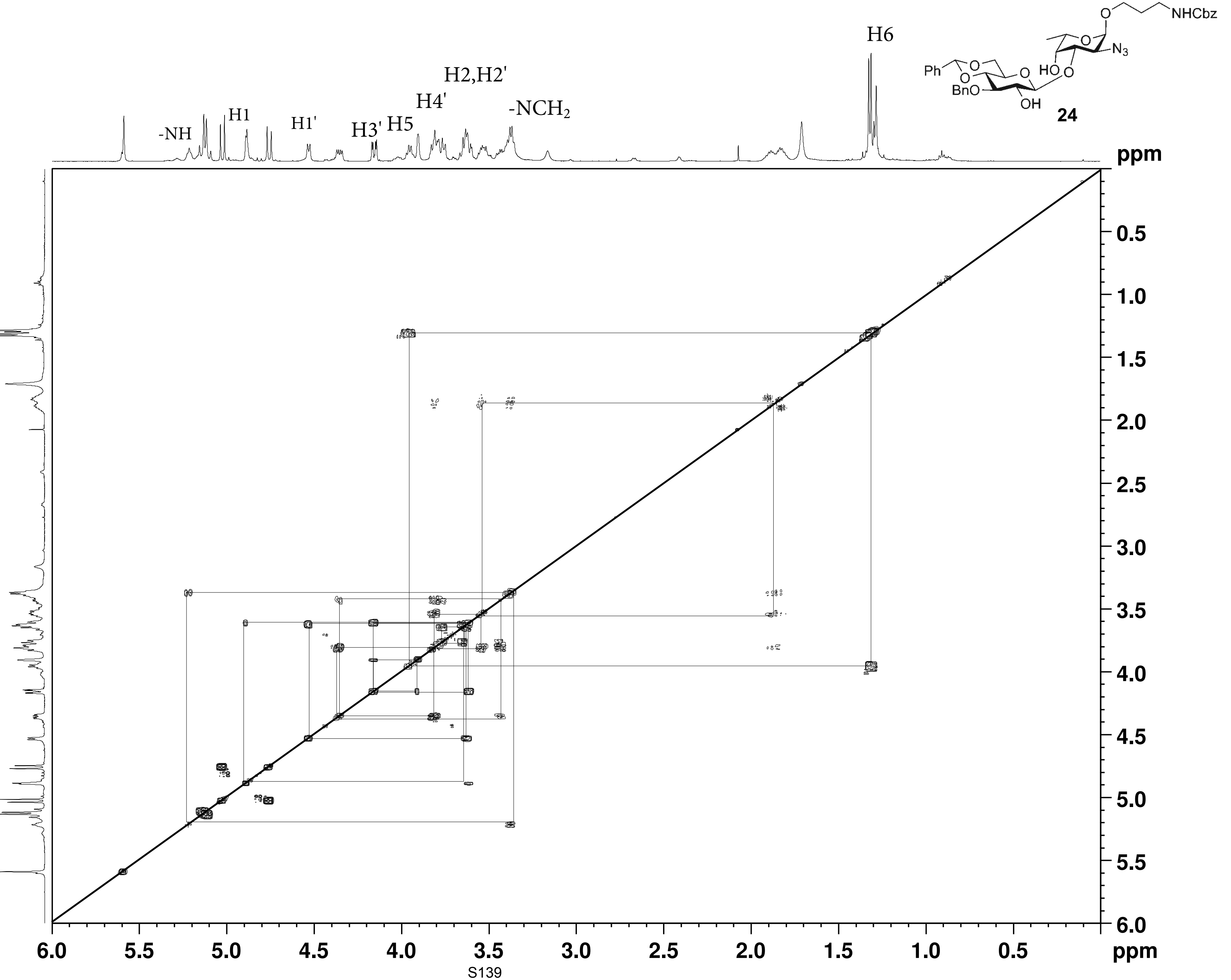


```
Current Data Parameters
NAME      SSK-34-AKM-442-DS-ACCEPTOR-COSY
EXPNO                 4
PROCNO                1

F2 - Acquisition Parameters
Date_          20240213
Time              22.50 h
INSTRUM           spect
PROBHD   Z124627_0008 (
PULPROG      cosygpppqf
TD                 2048
SOLVENT           CDCl3
NS                    2
DS                    0
SWH            3033.980 Hz
FIDRES         2.962872 Hz
AQ            0.3375104 sec
RG                61.42
DW              164.800 usec
DE                 6.50 usec
TE                296.0 K
D0           0.00000300 sec
D1           1.00000000 sec
D11          0.03000000 sec
D12          0.00002000 sec
D13          0.00000400 sec
D16          0.00020000 sec
IN0          0.00032960 sec
TDav                  1
SFO1        500.1314984 MHz
NUC1                 1H
P0                15.00 usec
P1                15.00 usec
P17             2500.00 usec
PLW1         9.39999962 W
PLW10        2.34999990 W
GPNAM[1]     SMSQ10.100
GPZ1              10.00 %
P16             1000.00 usec

F1 - Acquisition parameters
TD                  128
SFO1           500.1315 MHz
FIDRES        47.405945 Hz
SW                6.066 ppm
FnMODE               QF

F2 - Processing parameters
SI                 1024
SF          500.1300000 MHz
WDW               QSINE
SSB                   0
LB                    0 Hz
GB                    0
PC                 1.40

F1 - Processing parameters
SI                 1024
MC2                  QF
SF          500.1300000 MHz
WDW               QSINE
SSB                   0
LB                    0 Hz
GB                    0
```

SSK-34-AKM-442-DS-ACCEPTOR-HSQC

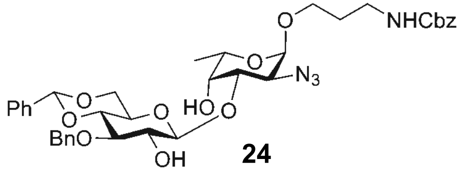

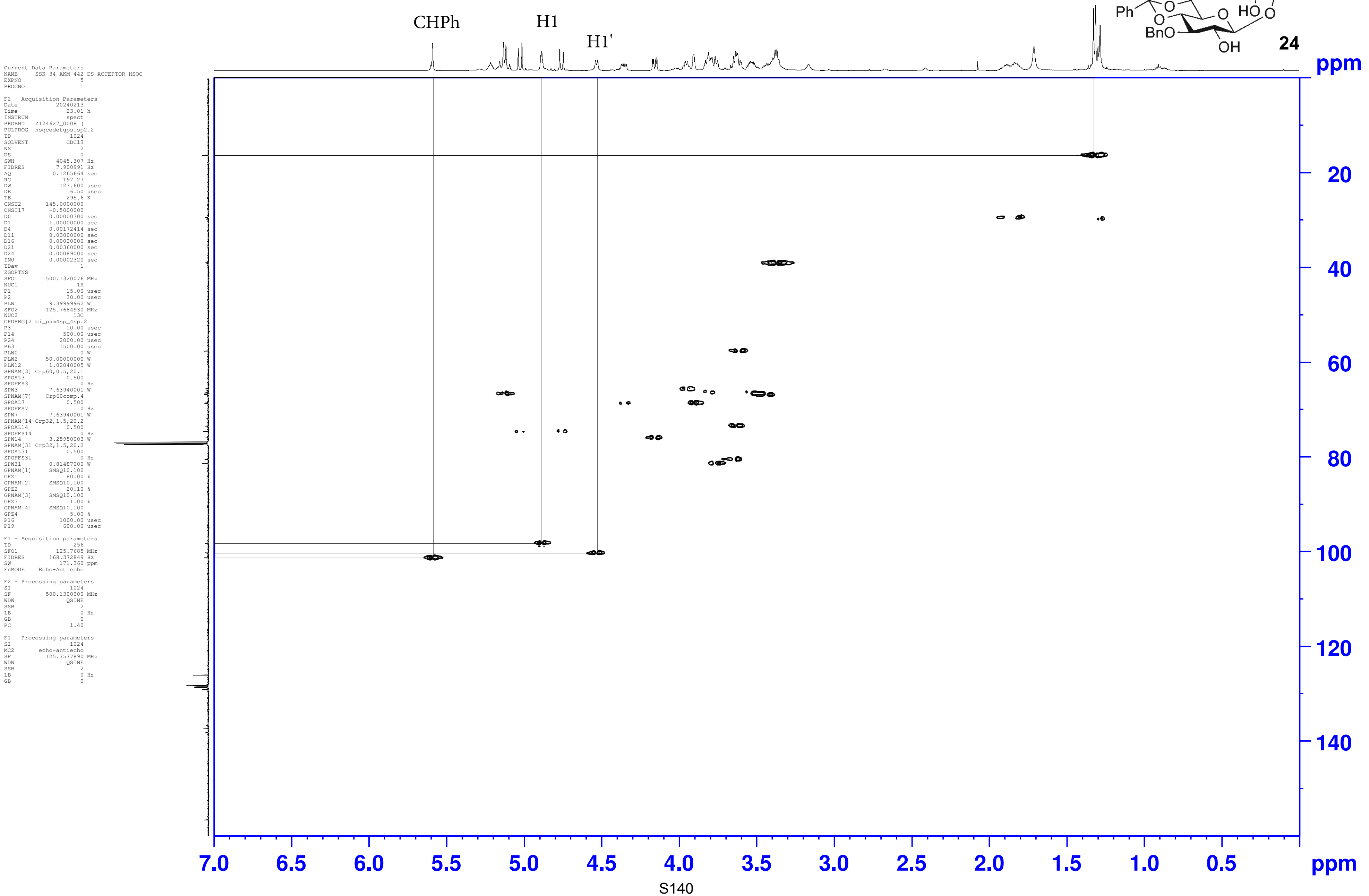

SSK-34-AKM-443-1H

```
Current Data Parameters
NAME      SSK-34-AKM-443-1H
EXPNO                     4
PROCNO                    1

F2 - Acquisition Parameters
Date_              20240215
Time                  12.11 h
INSTRUM               spect
PROBHD   Z104450_0346 (
PULPROG                zg30
TD                    54274
SOLVENT               CDCl3
NS                       25
DS                        0
SWH                8223.685 Hz
FIDRES             0.303043 Hz
AQ                3.2998593 sec
RG                      161
DW                   60.800 usec
DE                     6.50 usec
TE                    295.0 K
D1               1.00000000 sec
TD0                       1
SFO1            400.1324710 MHz
NUC1                     1H
P0                     5.00 usec
P1                    15.00 usec
PLW1             9.69999981 W

F2 - Processing parameters
SI                    32768
SF              400.1300095 MHz
WDW                      EM
SSB                       0
LB                     0.30 Hz
GB                        0
PC                     1.00
```

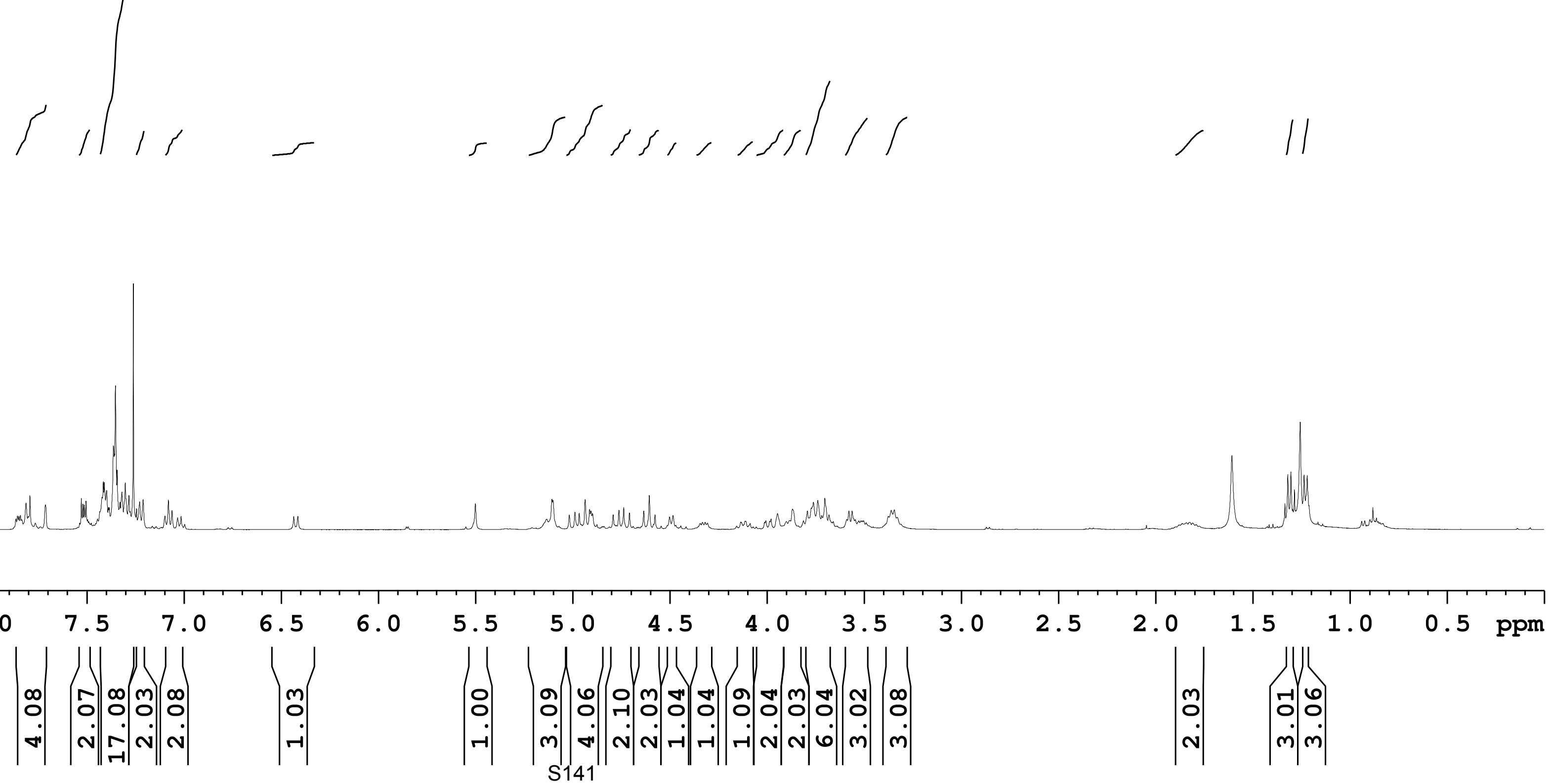

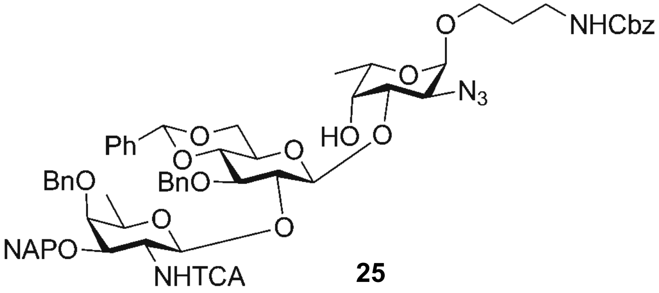

25

SSK-34-AKM-443-13C

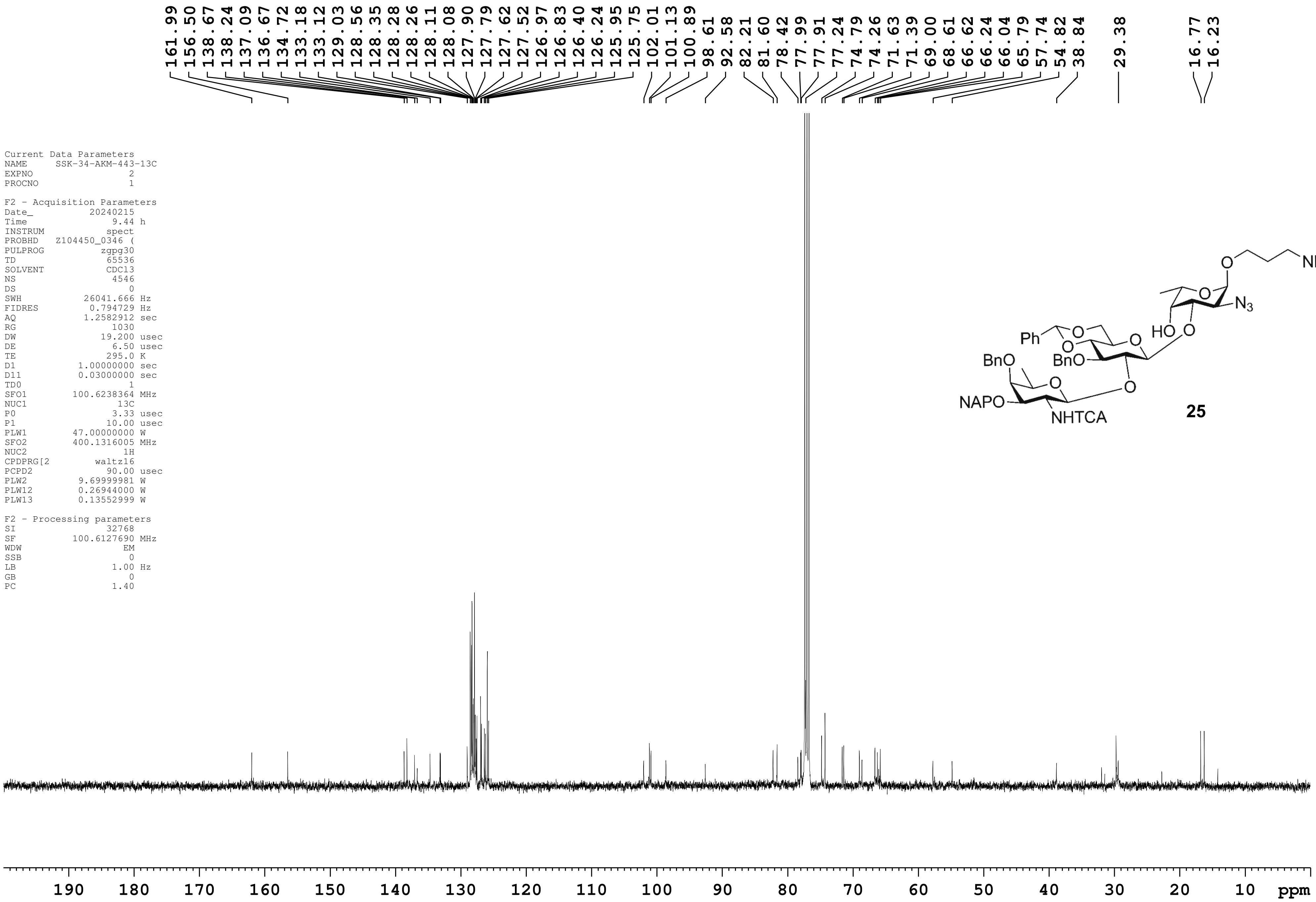


```
Current Data Parameters
NAME      SSK-34-AKM-443-13C
EXPNO                    2
PROCNO                   1

F2 - Acquisition Parameters
Date_             20240215
Time                  9.44 h
INSTRUM              spect
PROBHD   Z104450_0346 (
PULPROG            zgpg30
TD                  65536
SOLVENT             CDCl3
NS                   4546
DS                      0
SWH             26041.666 Hz
FIDRES           0.794729 Hz
AQ              1.2582912 sec
RG                   1030
DW                 19.200 usec
DE                   6.50 usec
TE                  295.0 K
D1             1.00000000 sec
D11            0.03000000 sec
TD0                     1
SFO1          100.6238364 MHz
NUC1                  13C
P0                   3.33 usec
P1                  10.00 usec
PLW1          47.00000000 W
SFO2          400.1316005 MHz
NUC2                   1H
CPDPRG[2          waltz16
PCPD2               90.00 usec
PLW2           9.69999981 W
PLW12          0.26944000 W
PLW13          0.13552999 W

F2 - Processing parameters
SI                  32768
SF            100.6127690 MHz
WDW                    EM
SSB                     0
LB                   1.00 Hz
GB                      0
PC                   1.40
```


25

SSK-34-AKM-443-DEPT

Current Data Parameters
NAME SSK-34-AKM-443-DEPT
EXPNO 5
PROCNO 1

F2 - Acquisition Parameters
Date_ 20240215
Time 12.40 h
INSTRUM spect
PROBHD Z104450_0346 (
PULPROG dept135
TD 65536
SOLVENT CDCl3
NS 780
DS 0
SWH 27777.777 Hz
FIDRES 0.847710 Hz
AQ 1.1796480 sec
RG 203
DW 18.000 usec
DE 6.50 usec
TE 295.3 K
CNST2 145.0000000
D1 1.00000000 sec
D2 0.00344828 sec
D12 0.00002000 sec
TD0 1
SFO1 100.6242389 MHz
NUC1 13C
P1 10.00 usec
P2 20.00 usec
PLW1 47.00000000 W
SFO2 400.1316005 MHz
NUC2 1H
CPDPRG[2 waltz16
P3 15.00 usec
P4 30.00 usec
PCPD2 90.00 usec
PLW2 9.69999981 W
PLW12 0.26944000 W

F2 - Processing parameters
SI 32768
SF 100.6127690 MHz
WDW EM
SSB 0
LB 1.00 Hz
GB 0
PC 1.40

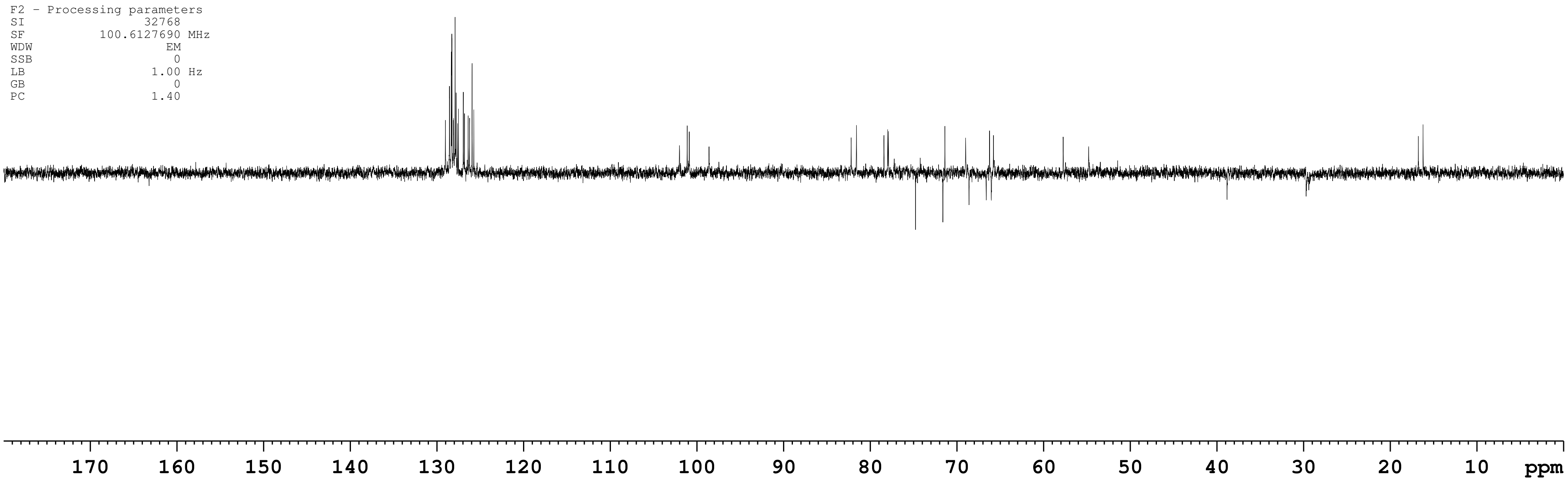

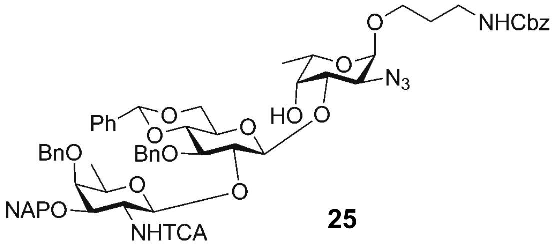

25

SSK-34-AKM-443-COSY

```
Current Data Parameters
NAME      SSK-34-AKM-443-COSY
EXPNO                    5
PROCNO                   1

F2 - Acquisition Parameters
Date_             20240215
Time                 14.36 h
INSTRUM              spect
PROBHD   Z104450_0346 (
PULPROG           cosygpqf
TD                    2048
SOLVENT              CDCl3
NS                       4
DS                       0
SWH               2615.063 Hz
FIDRES            2.553772 Hz
AQ               0.3915776 sec
RG                     575
DW                 191.200 usec
DE                    6.50 usec
TE                   295.1 K
D0             0.00000300 sec
D1             1.00000000 sec
D13            0.00000400 sec
D16            0.00020000 sec
IN0            0.00038240 sec
TDav                     1
SFO1           400.1313008 MHz
NUC1                    1H
P0                   15.00 usec
P1                   15.00 usec
PLW1            9.69999981 W
GPNAM[1]          SINE.100
GPZ1                 10.00 %
P16                1000.00 usec

F1 - Acquisition parameters
TD                     128
SFO1              400.1313 MHz
FIDRES           40.860355 Hz
SW                   6.536 ppm
FnMODE                  QF

F2 - Processing parameters
SI                    1024
SF             400.1300000 MHz
WDW                   SINE
SSB       0
LB        0 Hz
GB        0
PC                    1.40

F1 - Processing parameters
SI                    1024
MC2                     QF
SF             400.1300000 MHz
WDW                   SINE
SSB       0
LB        0 Hz
GB        0
```

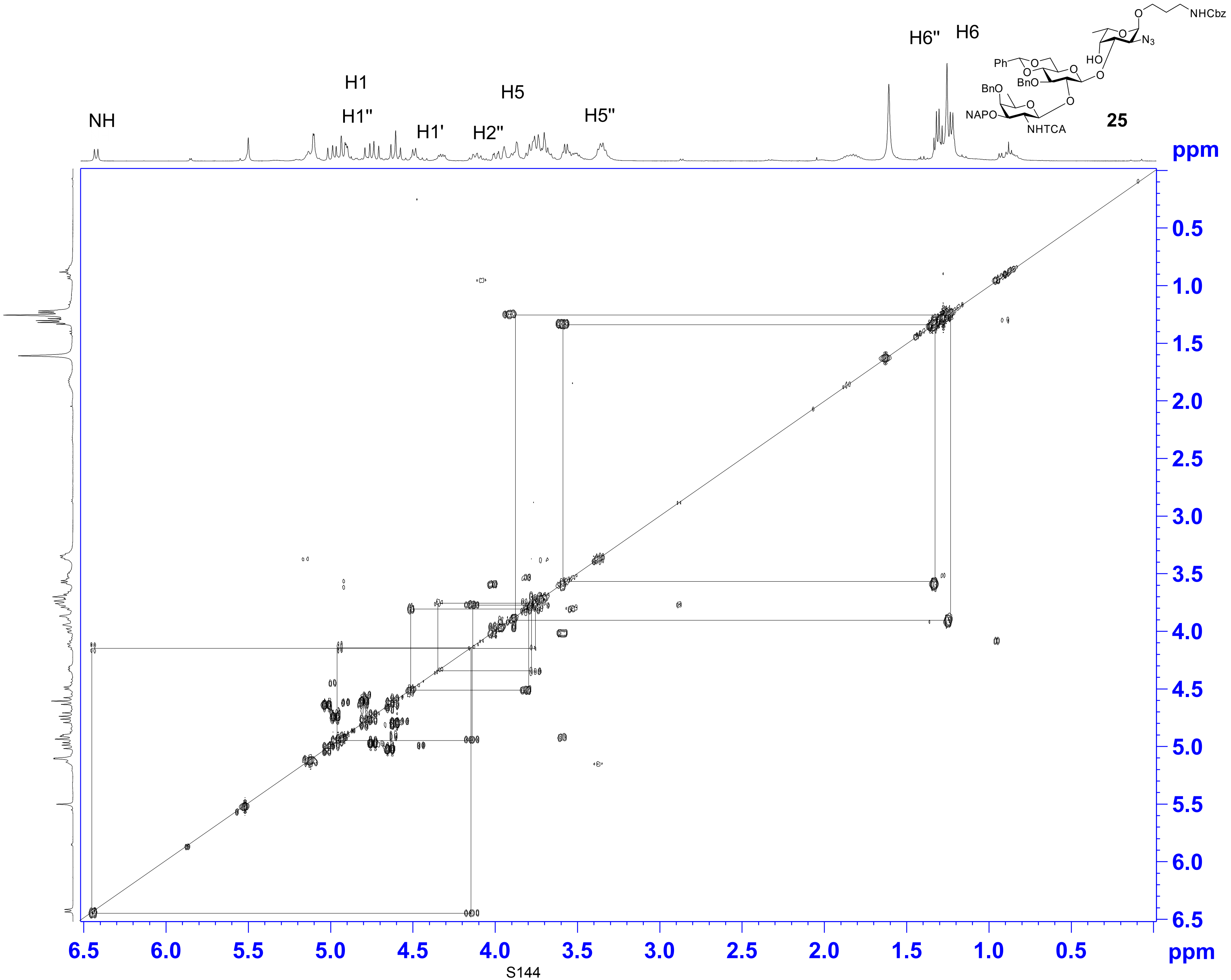

SSK-34-AKM-443-HSQC

```
Current Data Parameters
NAME      SSK-34-AKM-443-HSQC
EXPNO                    4
PROCNO                   1

F2 - Acquisition Parameters
Date_             20240215
Time                 12.56 h
INSTRUM              spect
PROBHD    Z104450_0346 (
PULPROG           hsqcetgp
TD                    2048
SOLVENT              CDCl3
NS                       4
DS                       0
SWH               4025.765 Hz
FIDRES            3.931411 Hz
AQ               0.2543616 sec
RG                    2050
DW                 124.200 usec
DE                    6.50 usec
TE                   295.3 K
CNST2          145.0000000
D0              0.00000300 sec
D1              1.00000000 sec
D4              0.00172414 sec
D11             0.03000000 sec
D16             0.00020000 sec
IN0             0.00002720 sec
TDav                     1
ZGOPTNS
SFO1           400.1320075 MHz
NUC1                    1H
P1                   15.00 usec
P2                   30.00 usec
PLW1            9.69999981 W
SFO2           100.6218336 MHz
NUC2                   13C
CPDPRG[2              garp
P3                   10.00 usec
P4                   20.00 usec
PCPD2                80.00 usec
PLW2           47.00000000 W
PLW12           0.73438001 W
GPNAM[1]          SINE.100
GPZ1                 80.00 %
GPNAM[2]          SINE.100
GPZ2                 20.10 %
P16                1000.00 usec

F1 - Acquisition parameters
TD                     191
SFO1              100.6218 MHz
FIDRES          192.485367 Hz
SW                 182.688 ppm
FnMODE        Echo-Antiecho

F2 - Processing parameters
SI                    2048
SF             400.1300000 MHz
WDW                  QSINE
SSB                      2
LB        0 Hz
GB        0
PC                    1.40

F1 - Processing parameters
SI                    1024
MC2           echo-antiecho
SF             100.6127690 MHz
WDW                  QSINE
SSB                      2
LB        0 Hz
GB        0
```

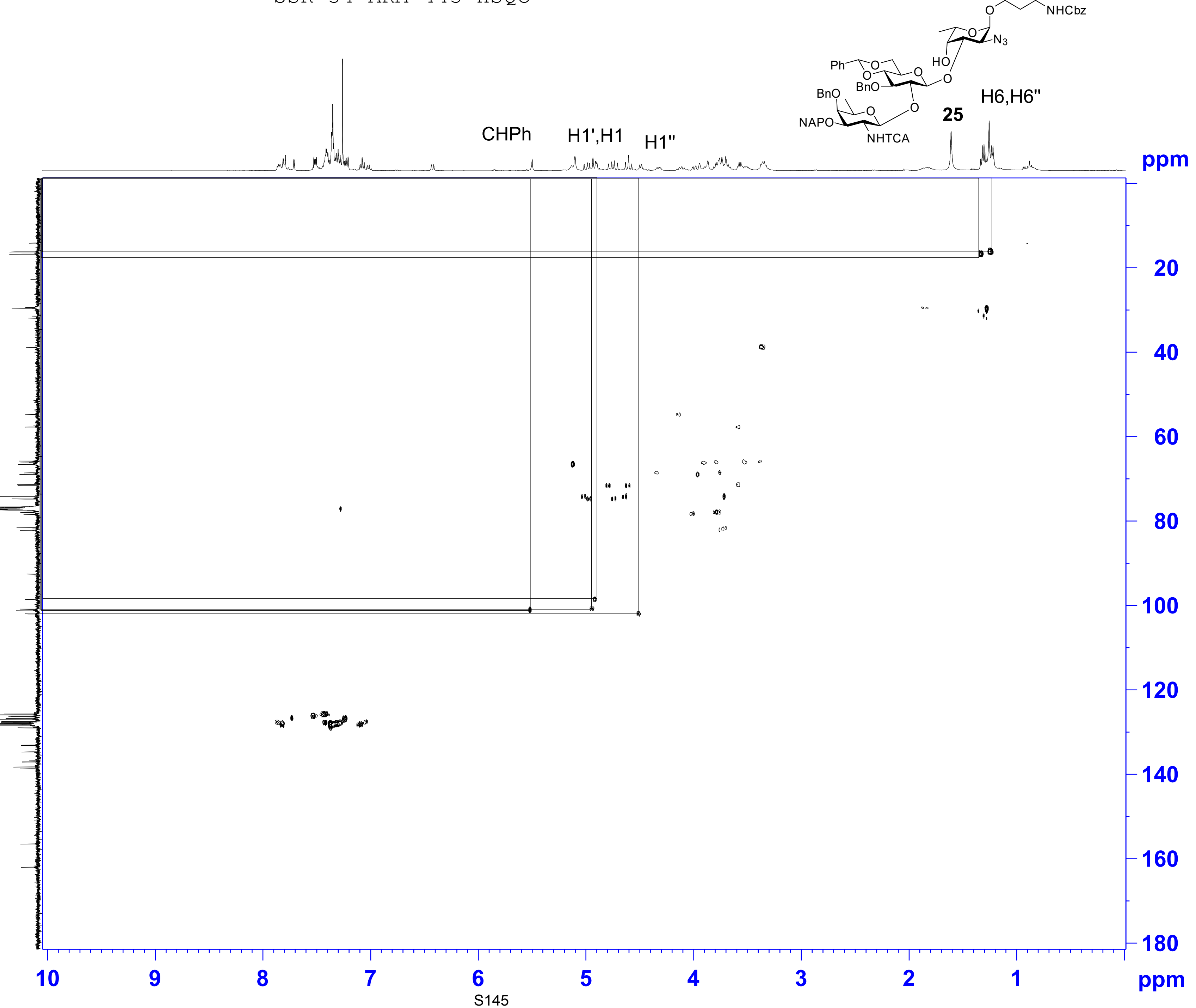

SSK-34-AKM-447-10-1H

Current Data Parameters
NAME SSK-34-AKM-447-10-1H
EXPNO 1
PROCNO 1

F2 - Acquisition Parameters
Date_ 20240301
Time 1.11 h
INSTRUM spect
PROBHD Z104450_0346 (
PULPROG zg30
TD 54274
SOLVENT D2O
NS 200
DS 0
SWH 8223.685 Hz
FIDRES 0.303043 Hz
AQ 3.2998593 sec
RG 161
DW 60.800 usec
DE 6.50 usec
TE 295.1 K
D1 1.00000000 sec
TD0 1
SFO1 400.1324710 MHz
NUC1 1H
P0 5.00 usec
P1 15.00 usec
PLW1 9.69999981 W

F2 - Processing parameters
SI 32768
SF 400.1300095 MHz
WDW EM
SSB 0
LB 0.30 Hz
GB 0
PC 1.00

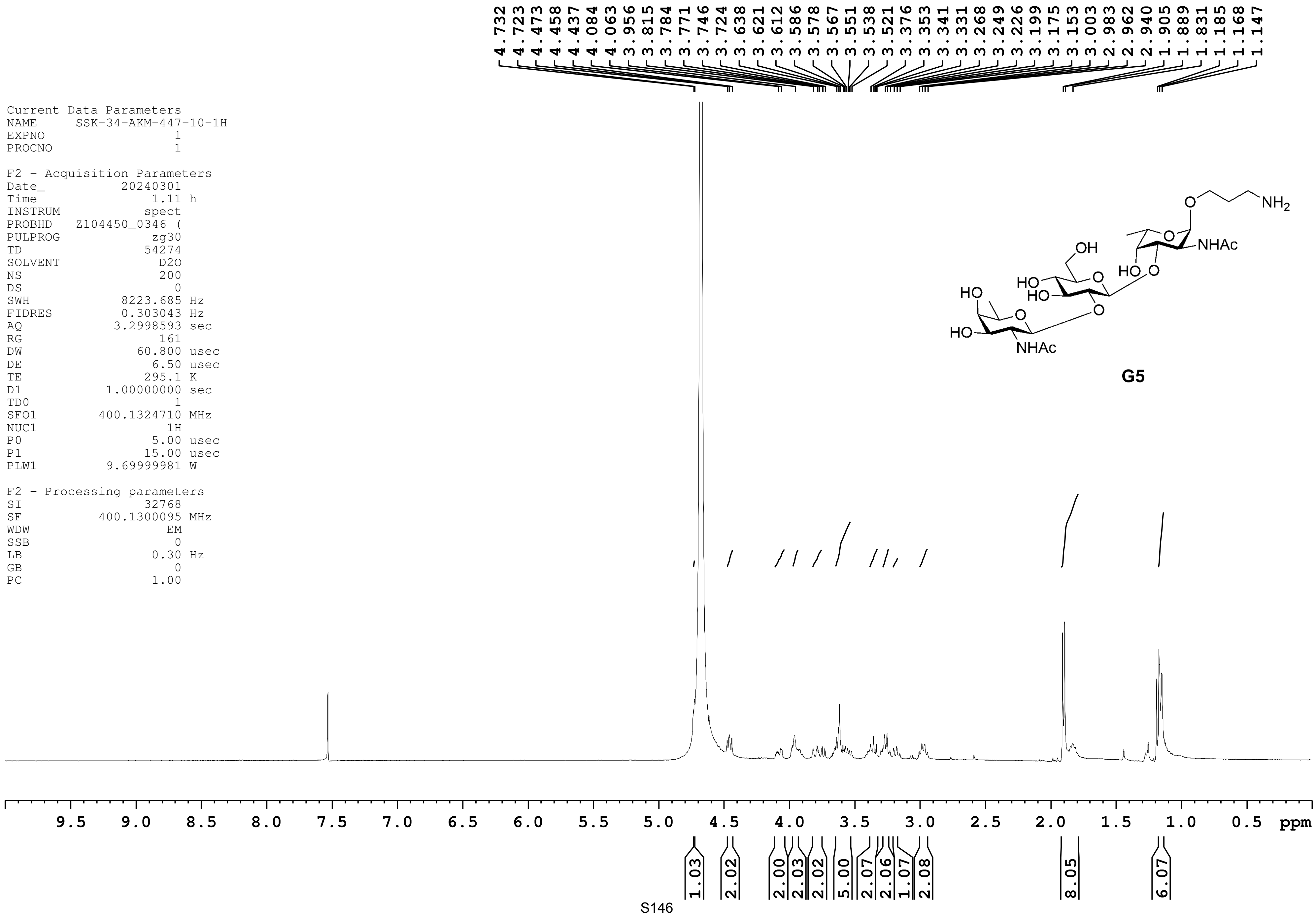

G5

SSK-34-AKM-447-13C

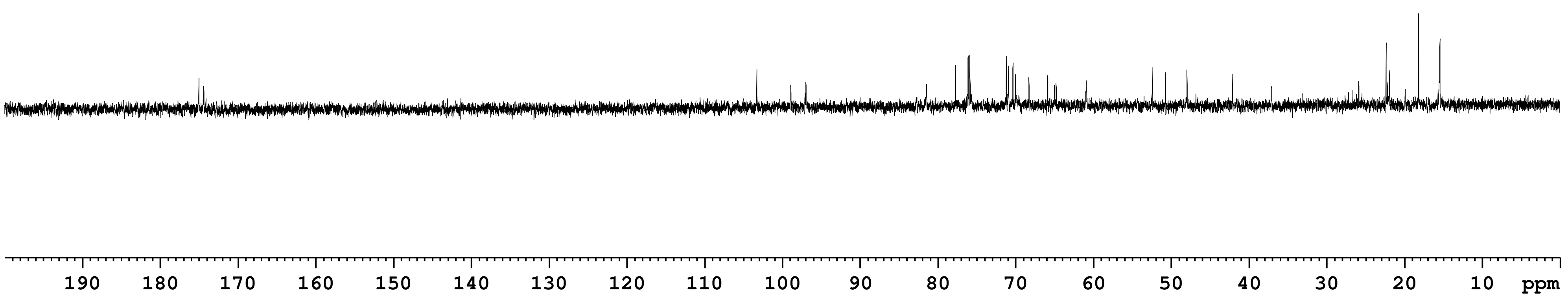


```
Current Data Parameters
NAME      SSK-34-AKM-447-13C
EXPNO                    3
PROCNO                   1

F2 - Acquisition Parameters
Date_           20240223
Time                1.56 h
INSTRUM            spect
PROBHD   Z104450_0346 (
PULPROG          zgpg30
TD                 65536
SOLVENT              D2O
NS                 14000
DS                     0
SWH           26041.666 Hz
FIDRES         0.794729 Hz
AQ            1.2582912 sec
RG                  2050
DW                19.200 usec
DE                  6.50 usec
TE                 295.1 K
D1            1.00000000 sec
D11           0.03000000 sec
TD0                    1
SFO1        100.6238364 MHz
NUC1                 13C
P0                  3.33 usec
P1                 10.00 usec
PLW1         47.00000000 W
SFO2        400.1316005 MHz
NUC2                  1H
CPDPRG[2         waltz16
PCPD2              90.00 usec
PLW2          9.69999981 W
PLW12         0.26944000 W
PLW13         0.13552999 W

F2 - Processing parameters
SI                 32768
SF          100.6127690 MHz
WDW                   EM
SSB                    0
LB                  1.00 Hz
GB                     0
PC                  1.20
```

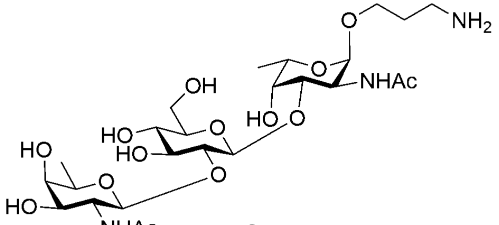

G5

SSK-34-AKM-447-DEPT

```
Current Data Parameters
NAME     SSK-34-AKM-447-DEPT
EXPNO                  4
PROCNO                 1

F2 - Acquisition Parameters
Date_          20240223
Time               8.07 h
INSTRUM           spect
PROBHD   Z104450_0346 (
PULPROG         dept135
TD                65536
SOLVENT             D2O
NS                10000
DS                    0
SWH           27777.777 Hz
FIDRES         0.847710 Hz
AQ            1.1796480 sec
RG                  203
DW               18.000 usec
DE                 6.50 usec
TE                294.8 K
CNST2       145.0000000
D1           1.00000000 sec
D2           0.00344828 sec
D12          0.00002000 sec
TD0                   1
SFO1        100.6242389 MHz
NUC1                13C
P1                10.00 usec
P2                20.00 usec
PLW1        47.00000000 W
SFO2        400.1316005 MHz
NUC2                 1H
CPDPRG[2        waltz16
P3                15.00 usec
P4                30.00 usec
PCPD2             90.00 usec
PLW2         9.69999981 W
PLW12        0.26944000 W

F2 - Processing parameters
SI                32768
SF          100.6127687 MHz
WDW                  EM
SSB                   0
LB                 1.00 Hz
GB                    0
PC                 1.40
```

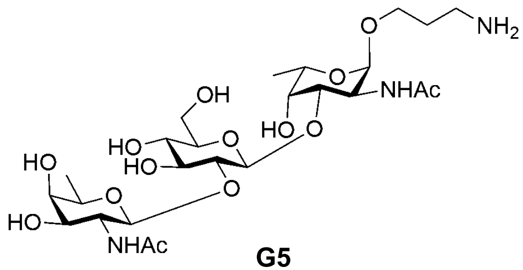

G5

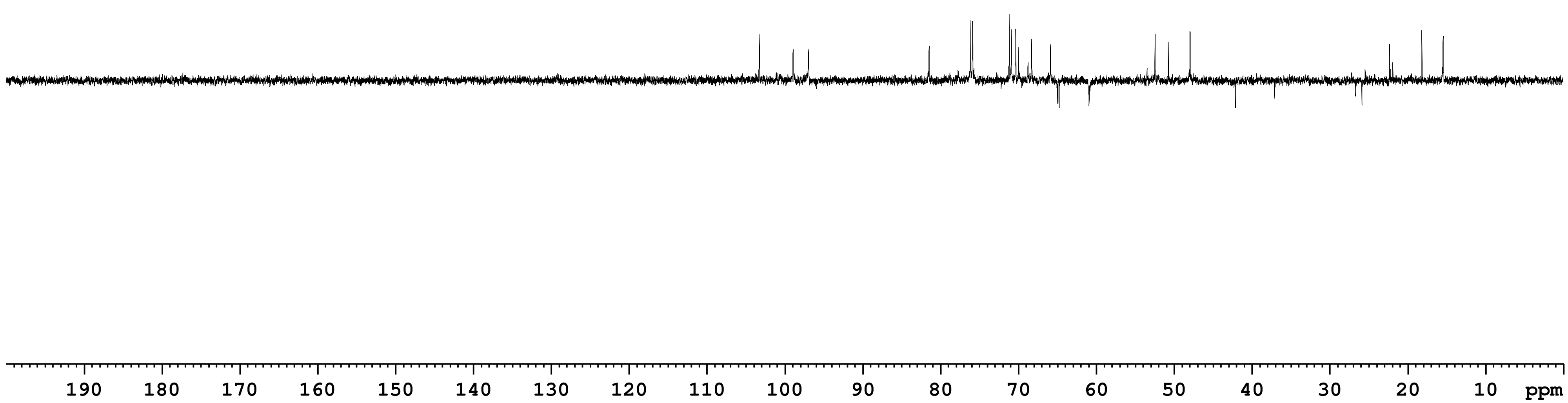

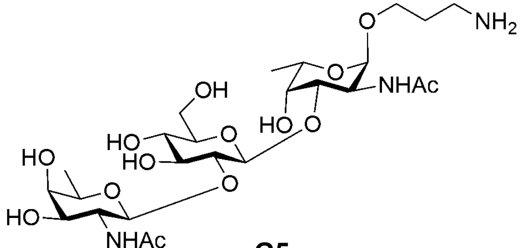

G5

COSYDFGPPH19 with water supp

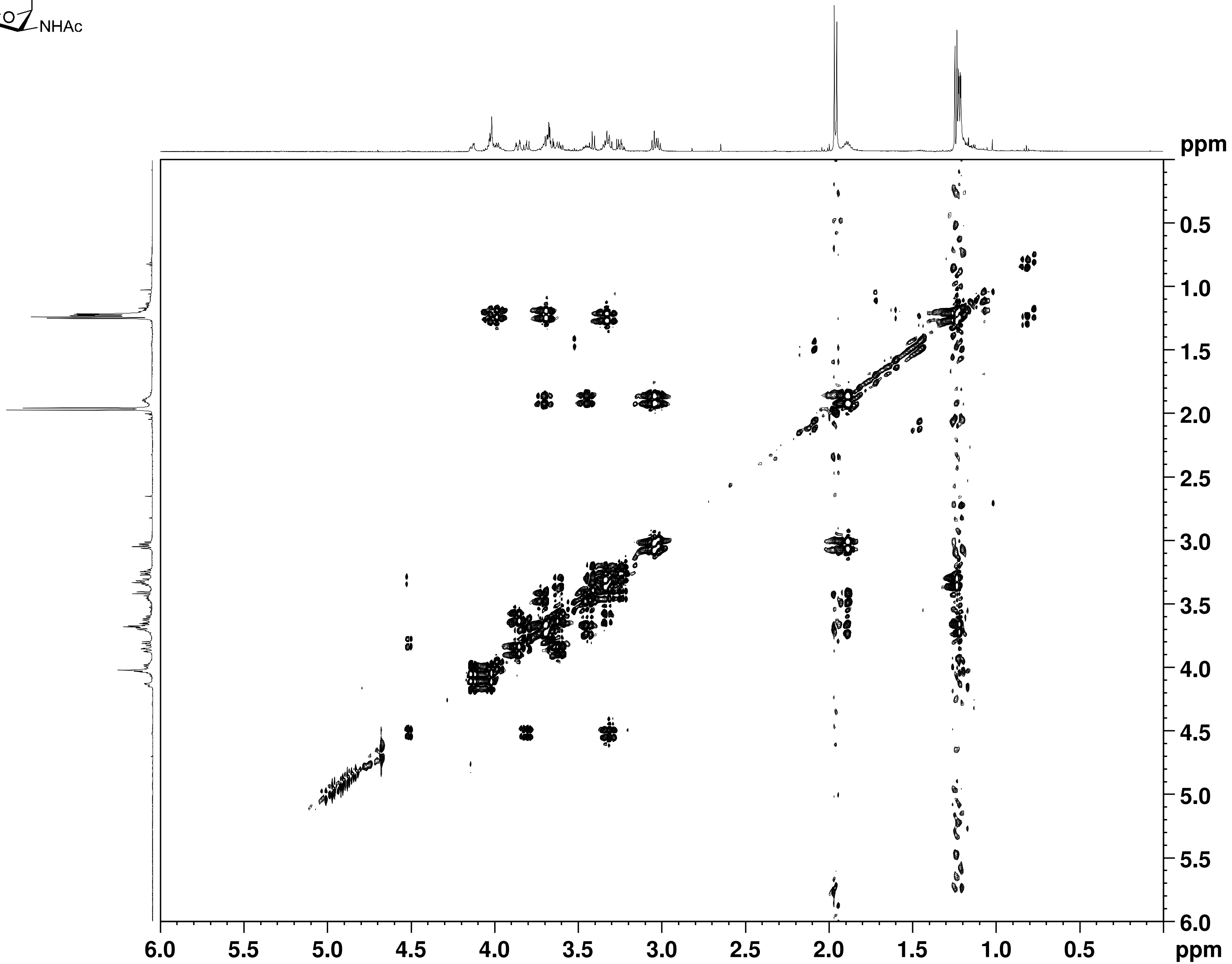


```
Current Data Parameters
NAME            SSK-AKM
EXPNO                13
PROCNO                1

F1 - Acquisition parameters
TD                  256
SFO1           600.3728 MHz
FIDRES        61.035156 Hz
SW               13.013 ppm
FnMODE       States-TPPI

F2 - Processing parameters
SI                 1024
SF          600.3700000 MHz
WDW               QSINE
SSB                   2
LB                    0 Hz
GB                    0
PC                 1.00

F1 - Processing parameters
SI                 1024
MC2          States-TPPI
SF          600.3700000 MHz
WDW               QSINE
SSB                   2
LB                    0 Hz
GB                    0
```

TOCSY
MLEVPHPR
Sample ID: SSK-34-AKM-FINAL
SolventD2O

Current Data Parameters
NAME SSK-AKM
EXPNO 10
PROCNO 1

F1 - Acquisition parameters
TD 256
SFO1 600.3728 MHz
FIDRES 48.828125 Hz
SW 10.410 ppm
FnMODE States-TPPI

F2 - Processing parameters
SI 2048
SF 600.3700000 MHz
WDW QSINE
SSB 2
LB 0 Hz
GB 0
PC 1.00

F1 - Processing parameters
SI 1024
MC2 States-TPPI
SF 600.3700000 MHz
WDW QSINE
SSB 2
LB 0 Hz
GB 0

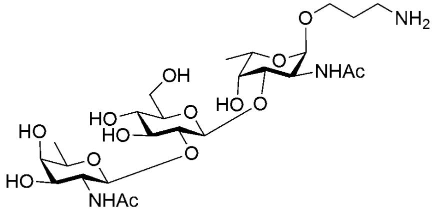

G5

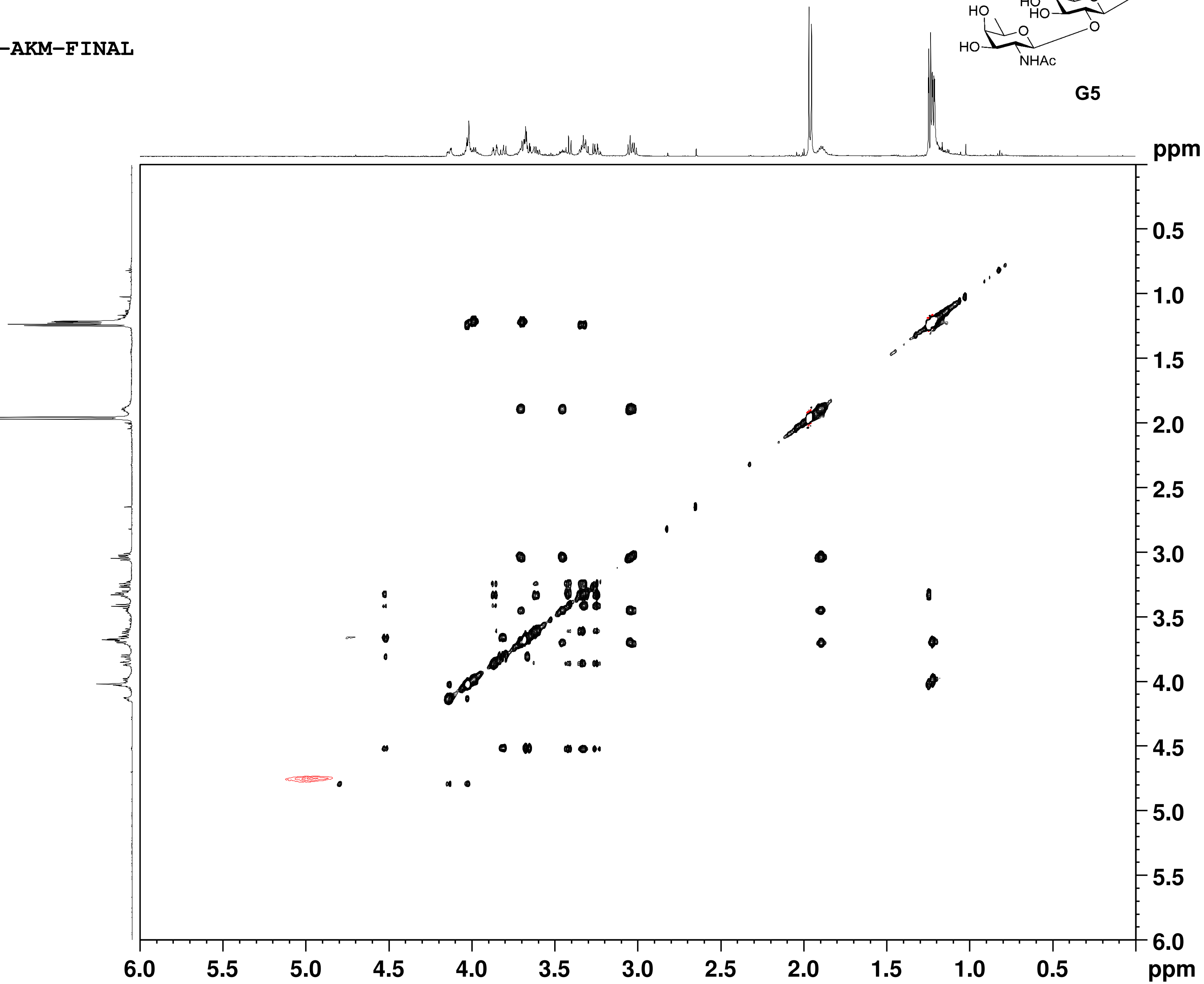

HSQCETGP

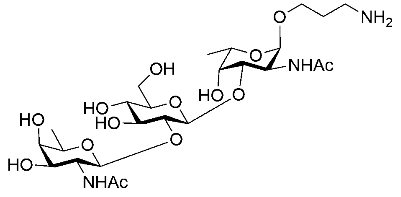

G5

Current Data Parameters
NAME SSK-AKM
EXPNO 12
PROCNO 1

F1 - Acquisition parameters
TD 256
SFO1 150.9745 MHz
FIDRES 194.615540 Hz
SW 165.000 ppm
FnMODE Echo-Antiecho

F2 - Processing parameters
SI 1024
SF 600.3700000 MHz
WDW QSINE
SSB 2
LB 0 Hz
GB 0
PC 1.00

F1 - Processing parameters
SI 1024
MC2 echo-antiecho
SF 150.9631566 MHz
WDW QSINE
SSB 2
LB 0 Hz
GB 0

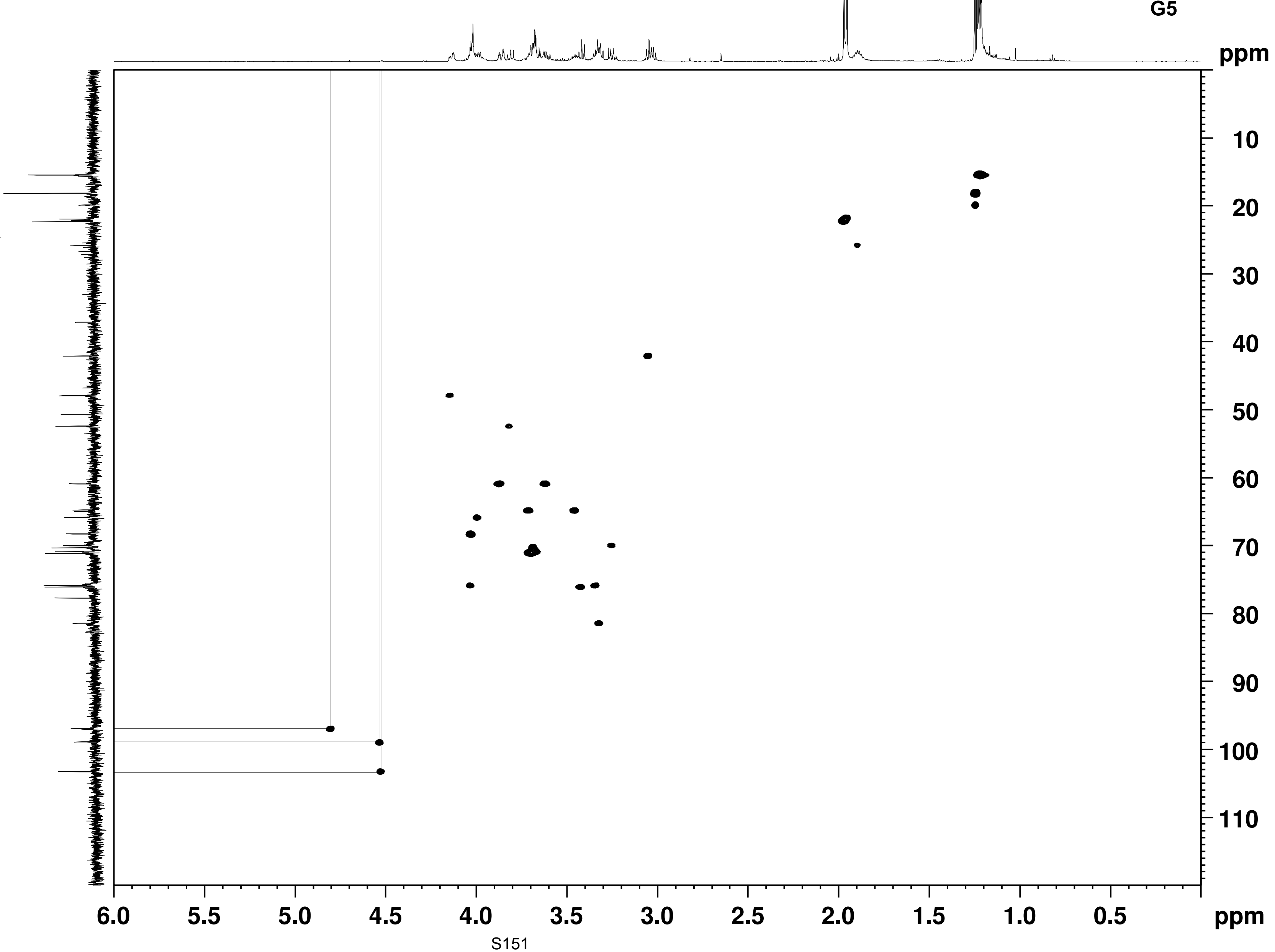

HMBCGP with water suppression

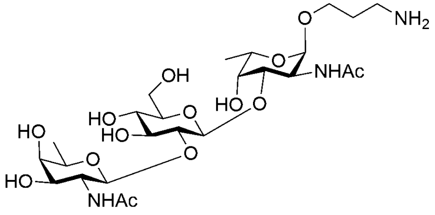

G5

```
Current Data Parameters
NAME            SSK-AKM
EXPNO                11
PROCNO                1

F1 - Acquisition parameters
TD                  128
SFO1         150.9783 MHz
FIDRES     518.987732 Hz
SW            220.000 ppm
FnMODE               QF

F2 - Processing parameters
SI                 2048
SF         600.3700000 MHz
WDW               QSINE
SSB                   0
LB                    0 Hz
GB                    0
PC                 1.00

F1 - Processing parameters
SI                 1024
MC2                  QF
SF         150.9631566 MHz
WDW               QSINE
SSB                   3
LB                    0 Hz
GB                    0
```

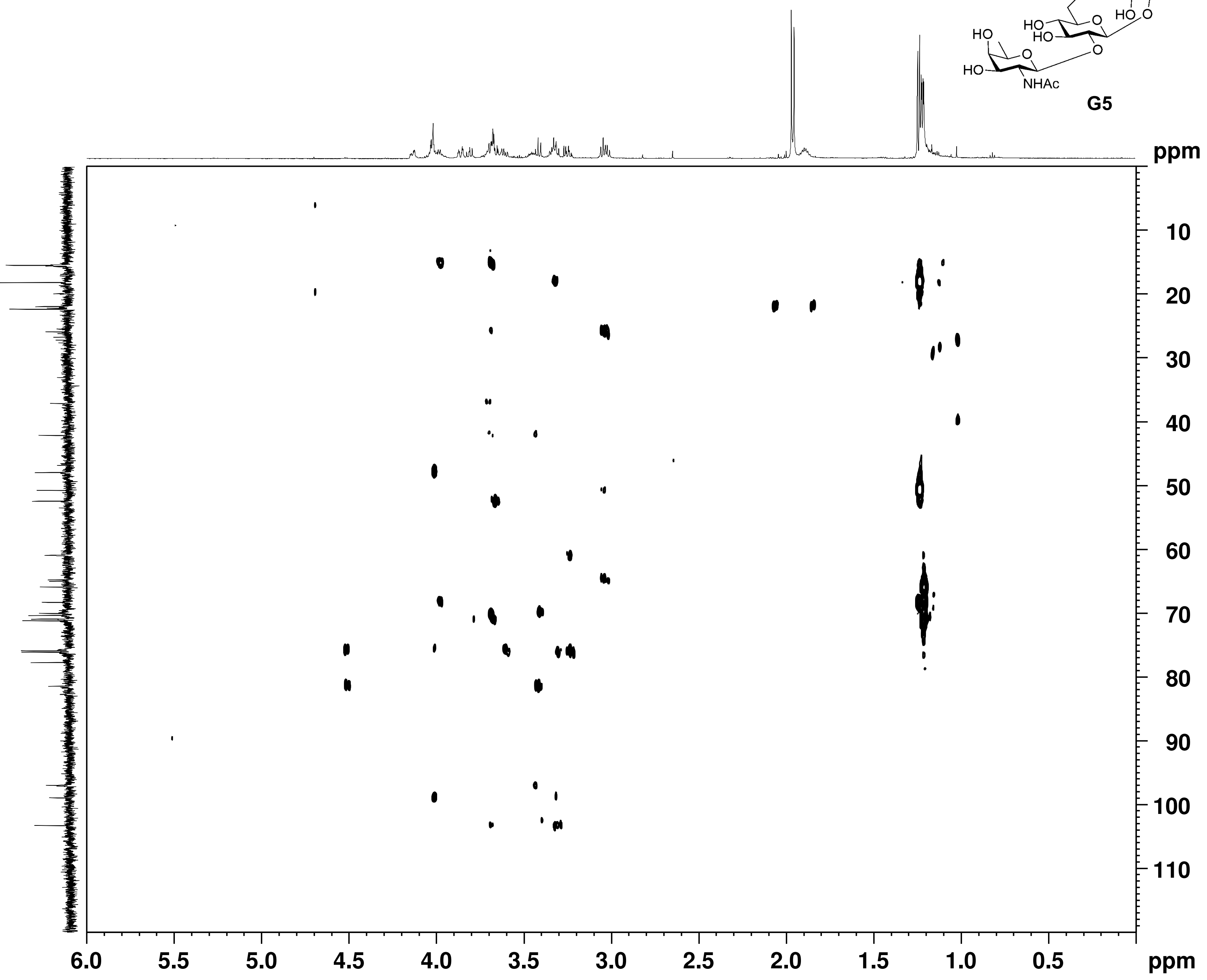

SSK-34-AKM-448-TETRA-1H

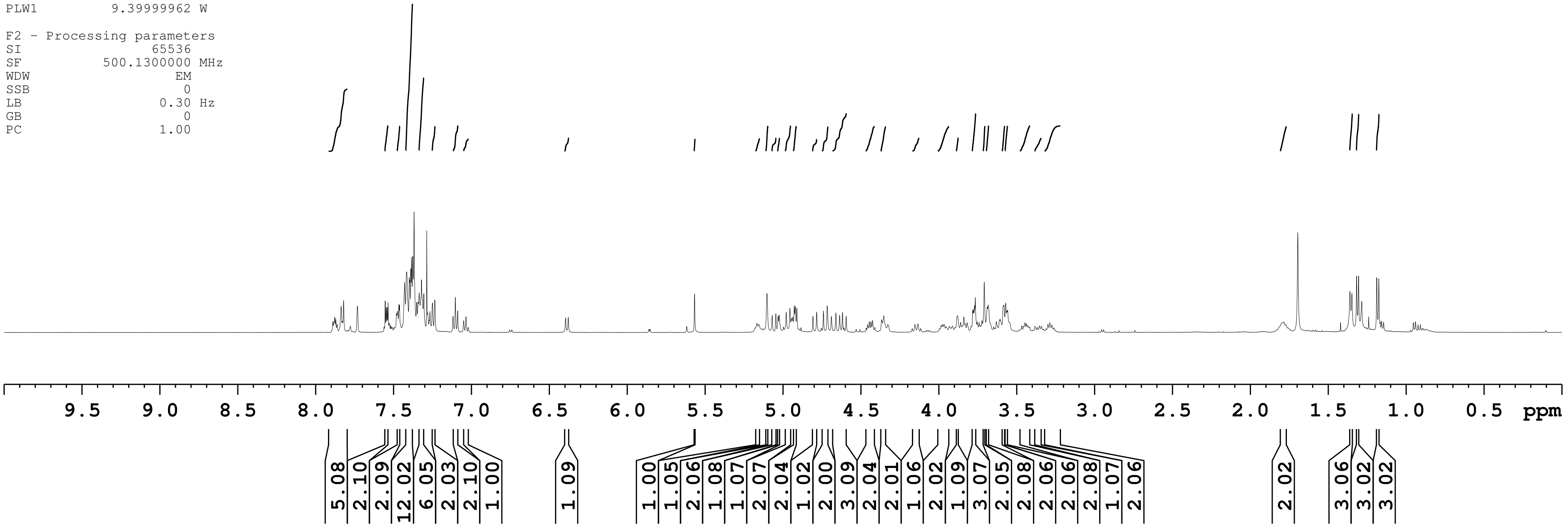

Current Data Parameters
NAME SSK-34-AKM-448-TETRA-1H
EXPNO 1
PROCNO 1

F2 - Acquisition Parameters
Date_ 20240222
Time 21.00 h
INSTRUM spect
PROBHD Z124627_0008 (
PULPROG zg30
TD 65536
SOLVENT CDCl3
NS 16
DS 0
SWH 10000.000 Hz
FIDRES 0.305176 Hz
AQ 3.2767999 sec
RG 197.27
DW 50.000 usec
DE 6.50 usec
TE 295.6 K
D1 1.00000000 sec
TD0 1
SFO1 500.1330885 MHz
NUC1 1H
P0 5.00 usec
P1 15.00 usec
PLW1 9.39999962 W

F2 - Processing parameters
SI 65536
SF 500.1300000 MHz
WDW EM
SSB 0
LB 0.30 Hz
GB 0
PC 1.00

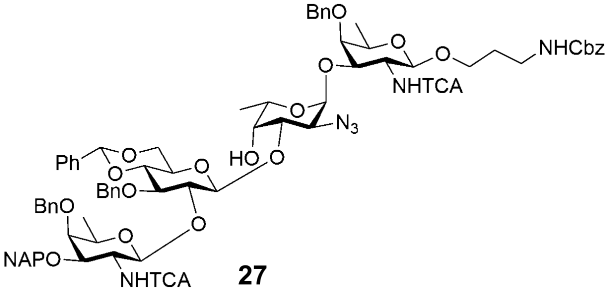

27

SSK-34-AKM-448-TETRA-13C

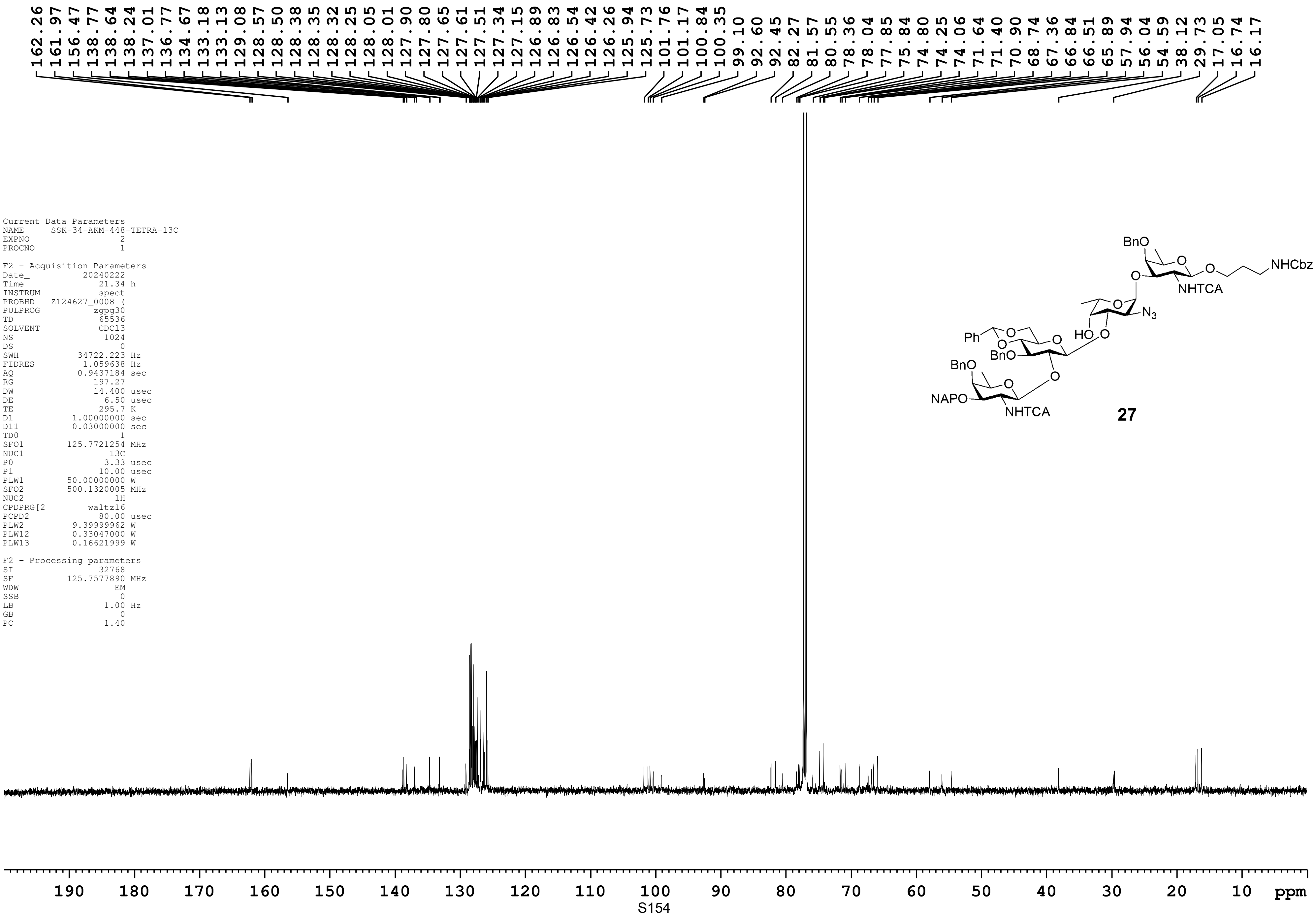


```
Current Data Parameters
NAME     SSK-34-AKM-448-TETRA-13C
EXPNO                     2
PROCNO                    1

F2 - Acquisition Parameters
Date_          20240222
Time              21.34 h
INSTRUM           spect
PROBHD   Z124627_0008 (
PULPROG          zgpg30
TD                65536
SOLVENT           CDCl3
NS                 1024
DS                    0
SWH           34722.223 Hz
FIDRES         1.059638 Hz
AQ            0.9437184 sec
RG               197.27
DW               14.400 usec
DE                 6.50 usec
TE                295.7 K
D1          1.00000000 sec
D11         0.03000000 sec
TD0                   1
SFO1        125.7721254 MHz
NUC1                13C
P0                 3.33 usec
P1                10.00 usec
PLW1        50.00000000 W
SFO2        500.1320005 MHz
NUC2                 1H
CPDPRG[2        waltz16
PCPD2             80.00 usec
PLW2         9.39999962 W
PLW12        0.33047000 W
PLW13        0.16621999 W

F2 - Processing parameters
SI                32768
SF          125.7577890 MHz
WDW                  EM
SSB                   0
LB                 1.00 Hz
GB                    0
PC                 1.40
```


27

SSK-34-AKM-448-DEPT

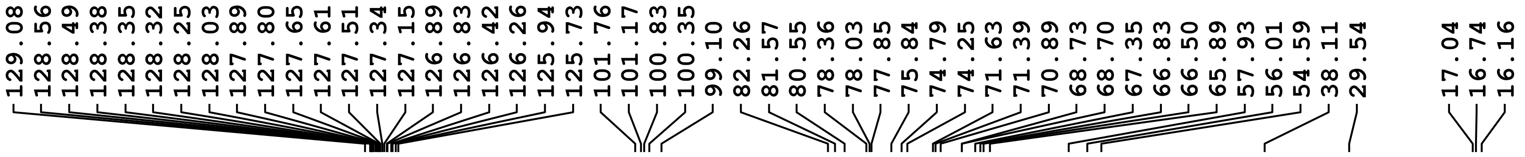


```
Current Data Parameters
NAME      SSK-34-AKM-448-DEPT
EXPNO                    2
PROCNO                   1

F2 - Acquisition Parameters
Date_           20240223
Time               13.56 h
INSTRUM            spect
PROBHD   Z104450_0346 (
PULPROG          dept135
TD                 65536
SOLVENT            CDCl3
NS                   400
DS                     0
SWH            27777.777 Hz
FIDRES          0.847710 Hz
AQ             1.1796480 sec
RG                   203
DW                18.000 usec
DE                  6.50 usec
TE                 295.2 K
CNST2        145.0000000
D1            1.00000000 sec
D2            0.00344828 sec
D12           0.00002000 sec
TD0                    1
SFO1         100.6242389 MHz
NUC1                 13C
P1                 10.00 usec
P2                 20.00 usec
PLW1         47.00000000 W
SFO2         400.1316005 MHz
NUC2                  1H
CPDPRG[2         waltz16
P3                 15.00 usec
P4                 30.00 usec
PCPD2              90.00 usec
PLW2          9.69999981 W
PLW12         0.26944000 W

F2 - Processing parameters
SI                 32768
SF           100.6127690 MHz
WDW                   EM
SSB                    0
LB                  1.00 Hz
GB                     0
PC                  1.40
```

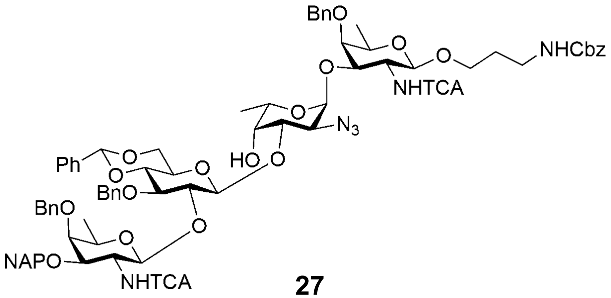

27

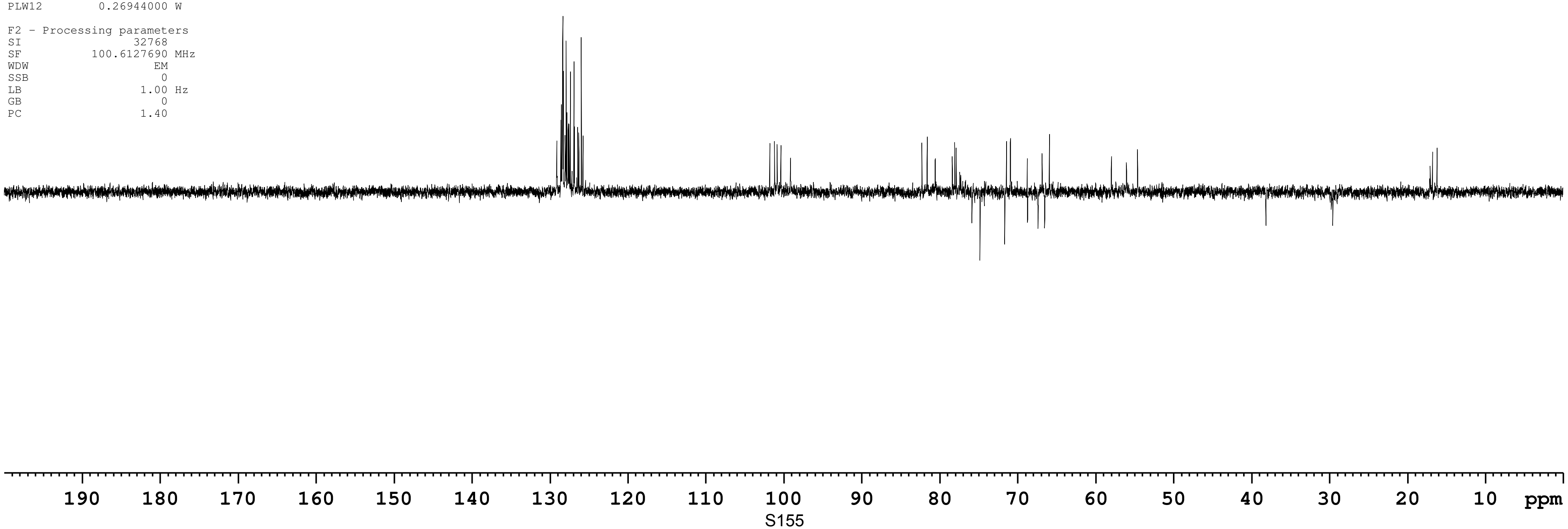

SSK-34-AKM-448-COSY

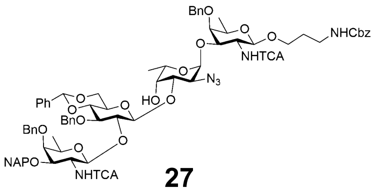

27

Current Data Parameter
NAME SSK-34-AKM-44
EXPNO
PROCNO

F2 - Acquisition Parame
Date_ 2024022
Time 14.3
INSTRUM spec
PROBHD Z104450_0346
PULPROG cosygpq
TD 204
SOLVENT CDCl
NS
DS
SWH 2626.05
FIDRES 2.56450
AQ 0.389939
RG 6
DW 190.40
DE 6.5
TE 295.
D0 0.0000030
D1 1.0000000
D13 0.0000040
D16 0.0002000
IN0 0.0003808
TDav
SFO1 400.131290
NUC1 1
P0 15.0
P1 15.0
PLW1 9.6999998

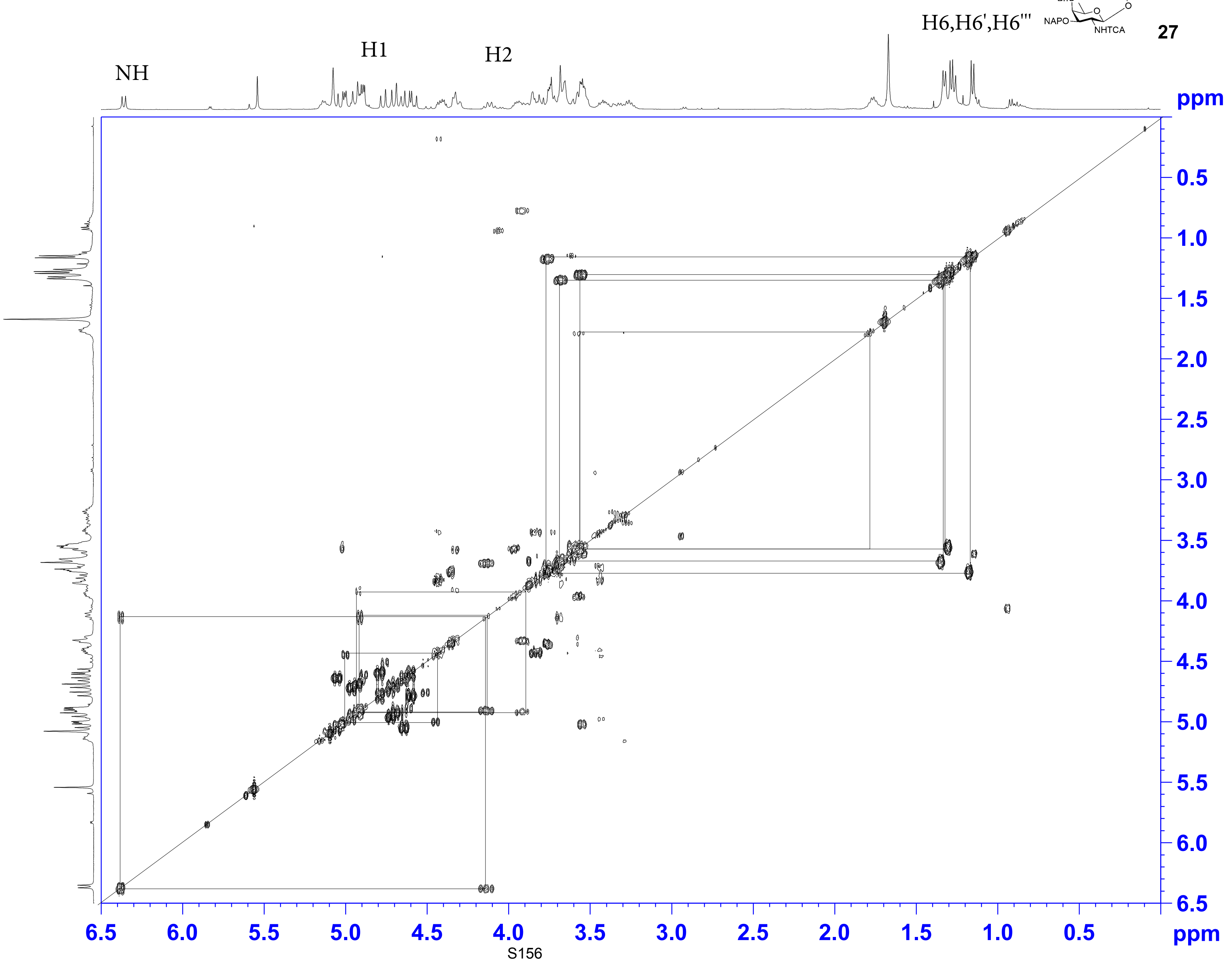

SSK-34-AKM-448-HSQC

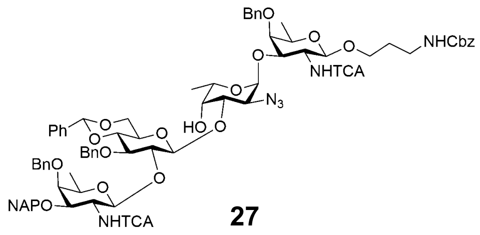

27

Current Data Parameters
NAME SSK-34-AKM-448-H[illegible]
EXPNO 3
PROCNO 1

F2 - Acquisition Parameters
Date_ 20240223
Time 14.20 h
INSTRUM spect
PROBHD Z104450_0346 (
PULPROG hsqcetgp
TD 2048
SOLVENT CDCl3
NS 4
DS 0
SWH 4032.258 Hz
FIDRES 3.937752 Hz
AQ 0.2539520 sec
RG 2050
DW 124.000 usec
DE 6.50 usec
TE 295.3 K
CNST2 145.0000000
D0 0.00000300 sec
D1 1.00000000 sec
D4 0.00172414 sec
D11 0.03000000 sec
D16 0.00020000 sec
IN0 0.00002760 sec
TDav 1
ZGOPTNS
[illegible]

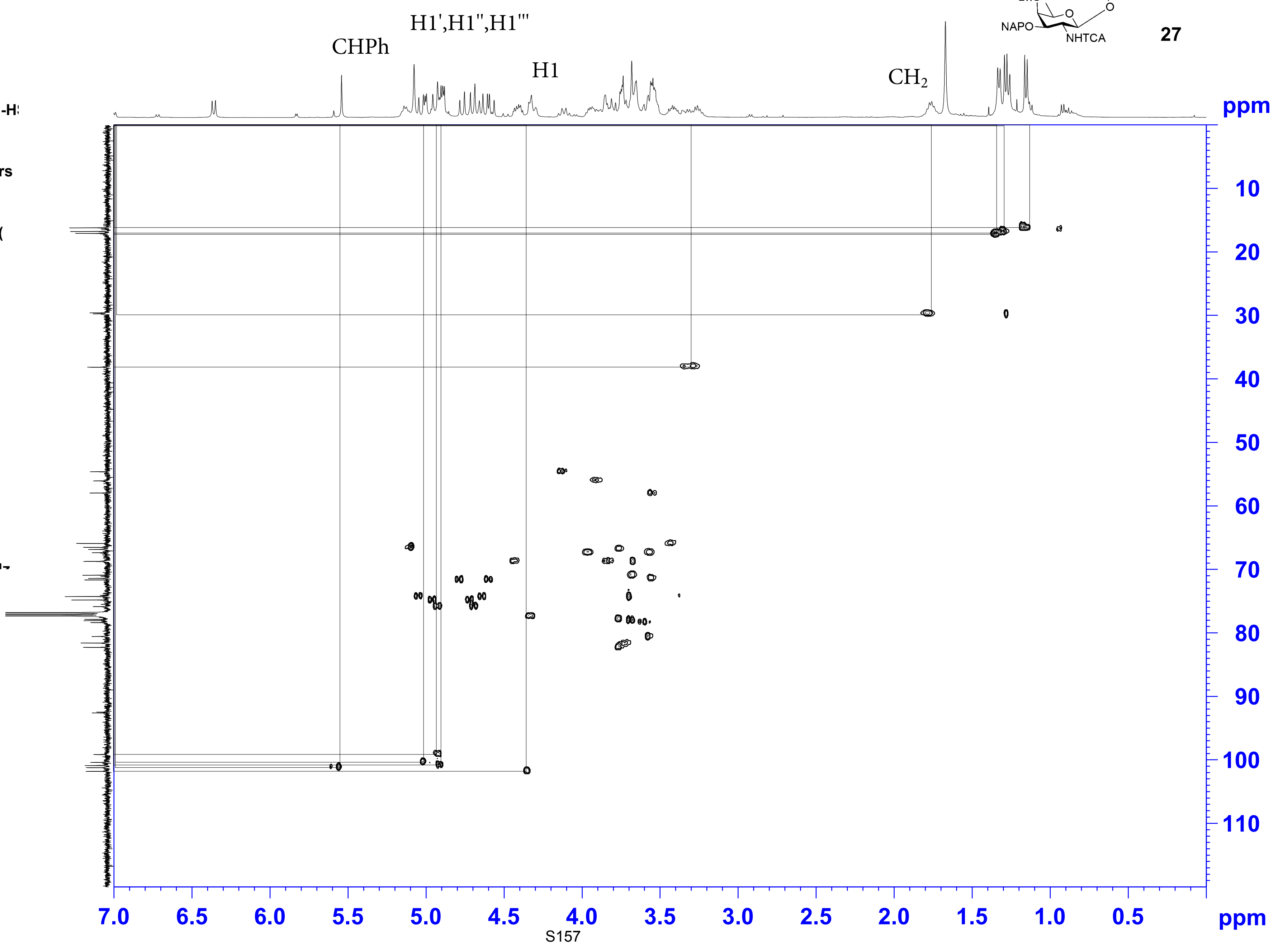

Sample ID: SSK-34-AKM-TETRA-FINAL
Solvent:D2O
Exp: 1D proton

Current Data Parameters
NAME 2-20251121T184453Z-1-001
EXPNO 2
PROCNO 1

F2 - Acquisition Parameters
Date_ 20250227
Time 17.
INSTRUM Avance
PROBHD Z154705_0128 (PI HR-BBO600S3-BBF/H/D-5.0
PULPROG
TD 24576
SOLVENT D2O
NS 64
DS 2
SWH 7812.500 Hz
FIDRES 0.635783 Hz
AQ 1.5728640 sec
RG 14.2
DW 64.000 usec
DE 6.50 usec
TE 298.1 K

F2 - Processing parameters
SI 16384
SF 600.3700000 M
WDW
SSB 0
LB 0.40 Hz
GB 0
PC 1.00

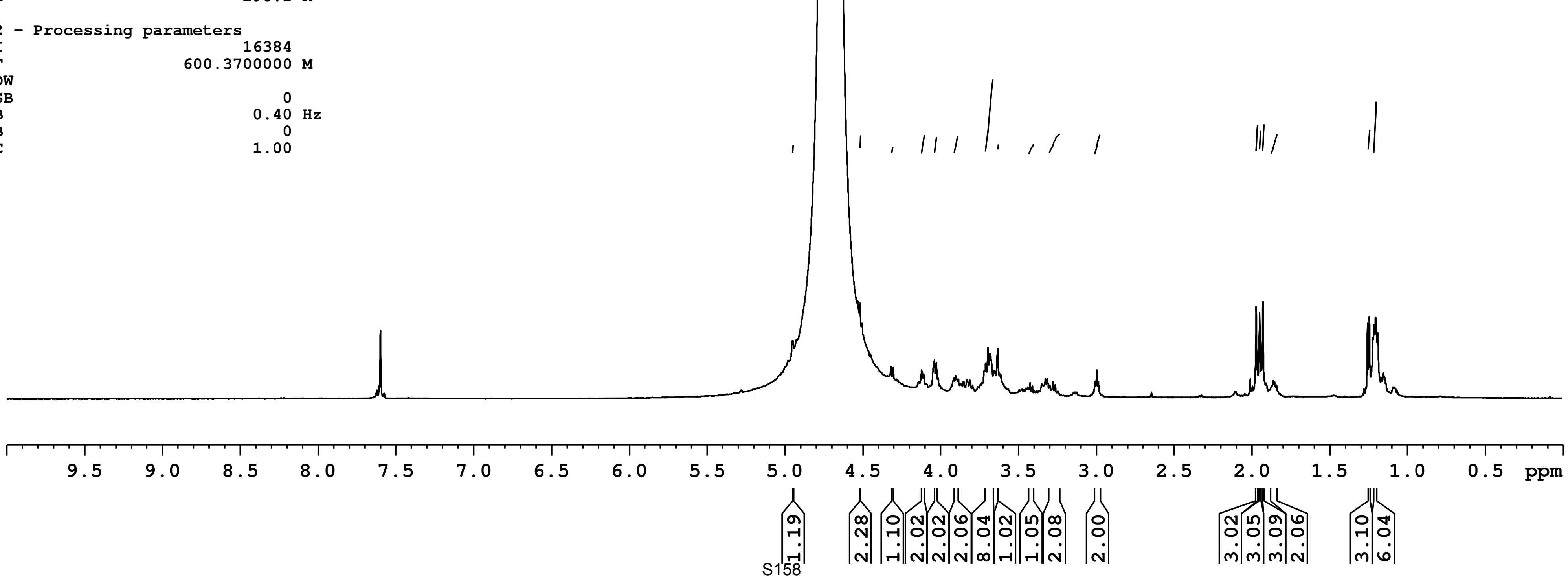

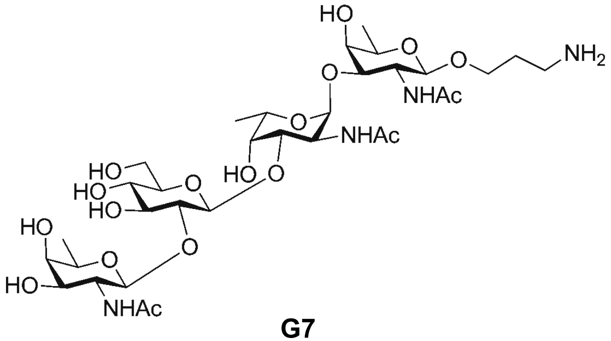

G7

SSK-34-AKM-454-TETRA-13C-2

175.07
174.37
174.17
103.26
101.60
98.99
98.81
81.55
76.62
76.12
75.85
75.47
71.18
70.96
70.69
70.38
69.98
68.16
67.94
66.32
60.86
52.49
51.37
48.88
47.86
37.67
26.69
22.37
22.20
15.45
15.33

Current Data Parameters
NAME SSK-34-AKM-454-TETRA-13C-2
EXPNO 3
PROCNO 1

F2 - Acquisition Parameters
Date_ 20240307
Time 9.28 h
INSTRUM Avance
PROBHD Z163739_0237 (
PULPROG zgpg30
TD 65536
SOLVENT D2O
NS 14180
DS 0
SWH 27777.778 Hz
FIDRES 0.847710 Hz
AQ 1.1796480 sec
RG 101
DW 18.000 usec
DE 6.50 usec
TE 295.7 K
D1 1.00000000 sec
D11 0.03000000 sec
TD0 1
SFO1 100.6669898 MHz
NUC1 13C
P0 2.67 usec
P1 8.00 usec
PLW1 97.90799713 W
SFO2 400.3016012 MHz
NUC2 1H
CPDPRG[2 waltz65
PCPD2 90.00 usec
PLW2 21.00099945 W
PLW12 0.16593000 W
PLW13 0.08346400 W

F2 - Processing parameters
SI 32768
SF 100.6555151 MHz
WDW EM
SSB 0
LB 1.00 Hz
GB 0
PC 1.40

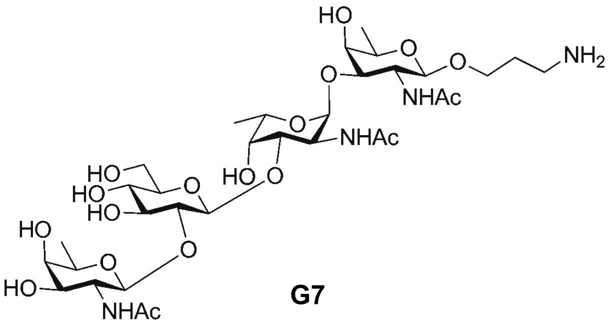

G7

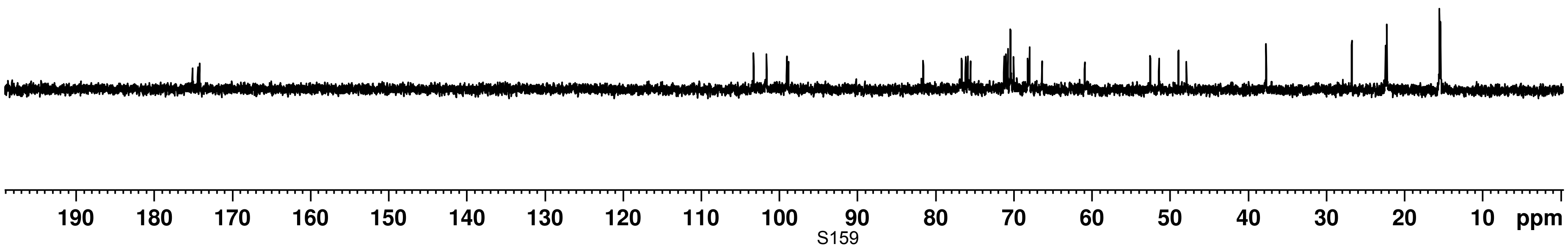

SSK-34-AKM-454-DEPT-1

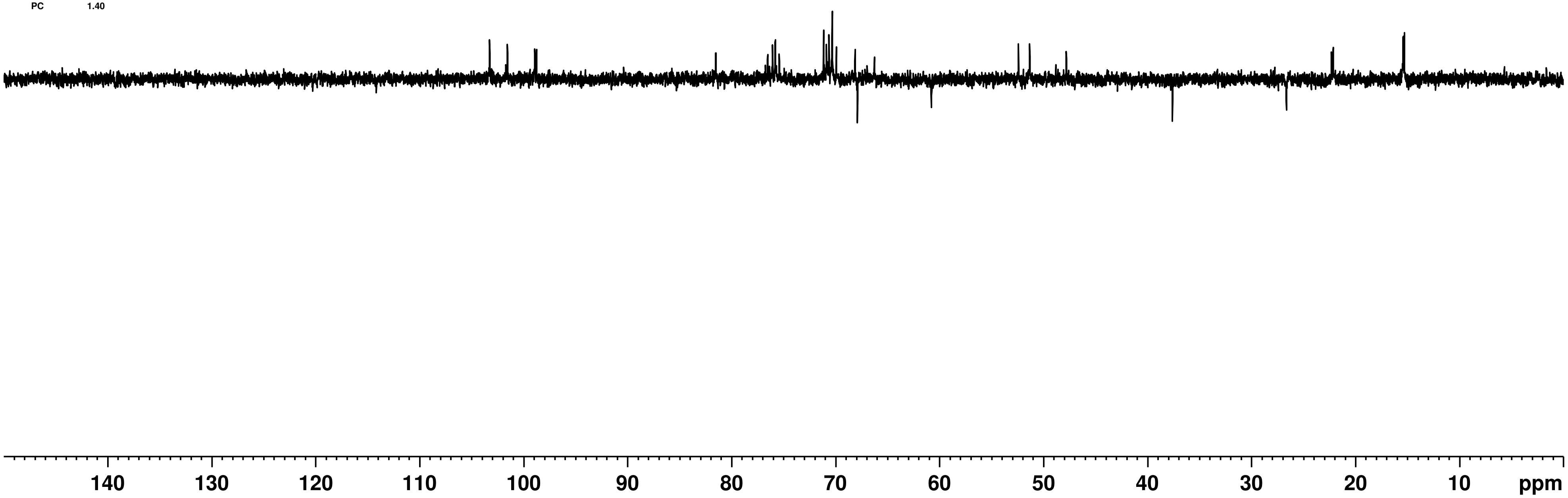

Current Data Parameters
NAME SSK-34-AKM-454-DEPT-1
EXPNO 1
PROCNO 1

F2 - Acquisition Parameters
Date_ 20240308
Time 3.10 h
INSTRUM spect
PROBHD Z104450_0346 (
PULPROG dept135
TD 65536
SOLVENT D2O
NS 4000
DS 0
SWH 27777.777 Hz
FIDRES 0.847710 Hz
AQ 1.1796480 sec
RG 2050
DW 18.000 usec
DE 6.50 usec
TE 295.5 K
CNST2 145.0000000
D1 1.00000000 sec
D2 0.00344828 sec
D12 0.00002000 sec
TD0 1
SFO1 100.6242389 MHz
NUC1 13C
P1 10.00 usec
P2 20.00 usec
PLW1 47.00000000 W
SFO2 400.1316005 MHz
NUC2 1H
CPDPRG[2 waltz16
P3 15.00 usec
P4 30.00 usec
PCPD2 90.00 usec
PLW2 9.69999981 W
PLW12 0.26944000 W

F2 - Processing parameters
SI 32768
SF 100.6127690 MHz
WDW EM
SSB 0
LB 1.00 Hz
GB 0
PC 1.40

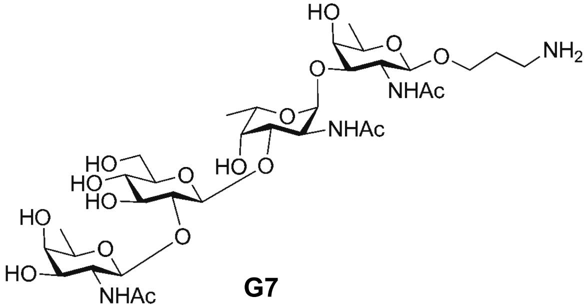

G7

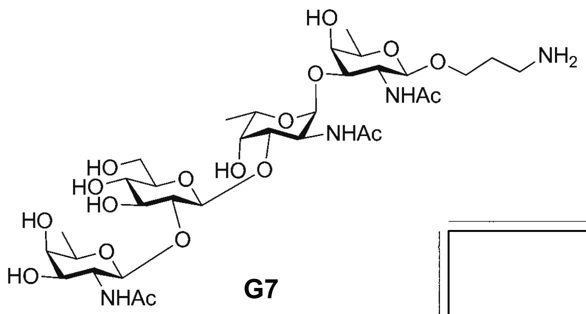

G7

COSYDFGPPH19

```
Current Data Parameters
NAME          SSK-AKM-1
EXPNO                10
PROCNO                1

F1 - Acquisition parameters
TD                  256
SFO1         600.3728 MHz
FIDRES       61.035156 Hz
SW             13.013 ppm
FnMODE       States-TPPI

F2 - Processing parameters
SI                 1024
SF          600.3700000 MHz
WDW               QSINE
SSB                   2
LB                    0 Hz
GB                    0
PC                 1.00

F1 - Processing parameters
SI                 1024
MC2          States-TPPI
SF          600.3700000 MHz
WDW               QSINE
SSB                   2
LB                    0 Hz
GB                    0
```

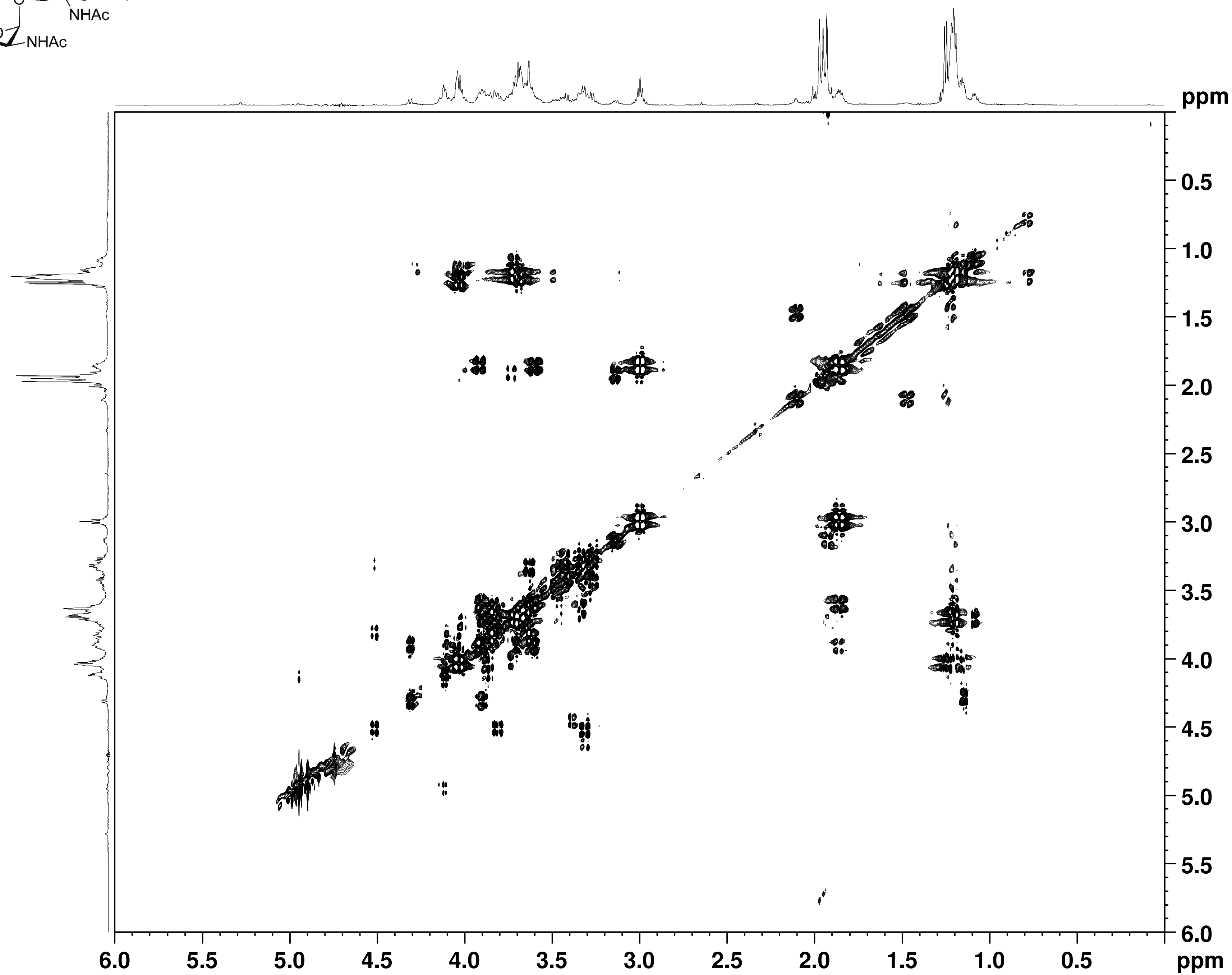

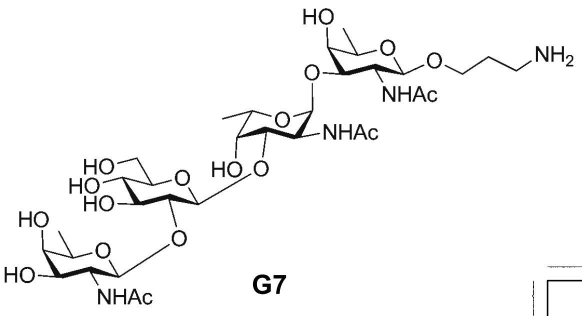

G7

TOCSY
MLEVPHPR
Sample ID: SSK-34-AKM-TETRA-FINAL
SolventD2O

```
Current Data Parameters
NAME            SSK-AKM
EXPNO                10
PROCNO                1

F1 - Acquisition parameters
TD                  256
SFO1        600.3728 MHz
FIDRES     48.828125 Hz
SW           10.410 ppm
FnMODE      States-TPPI

F2 - Processing parameters
SI                 2048
SF       600.3700000 MHz
WDW               QSINE
SSB                   2
LB                 0 Hz
GB                    0
PC                 1.00

F1 - Processing parameters
SI                 1024
MC2         States-TPPI
SF       600.3700000 MHz
WDW               QSINE
SSB                   2
LB                 0 Hz
GB                    0
```

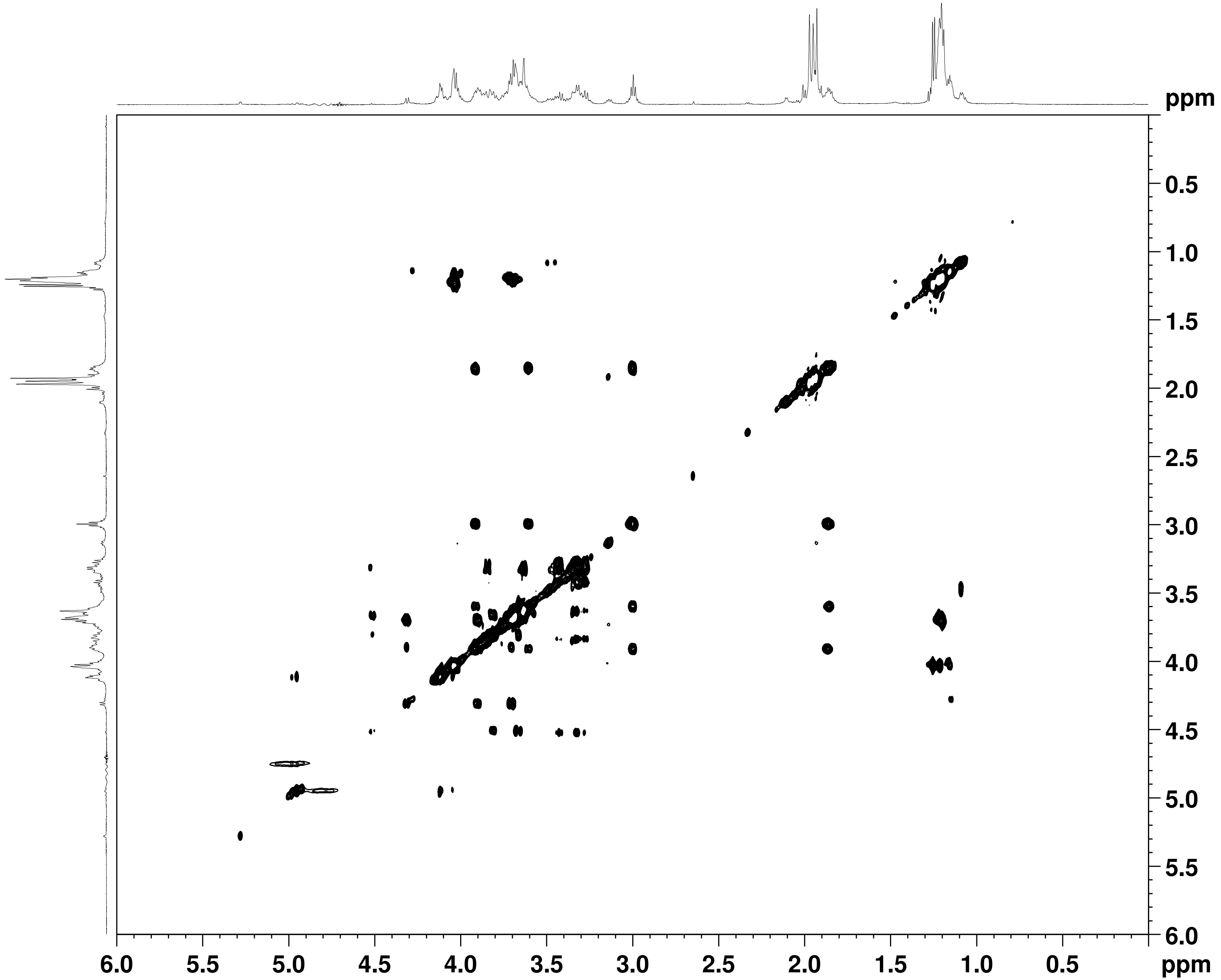

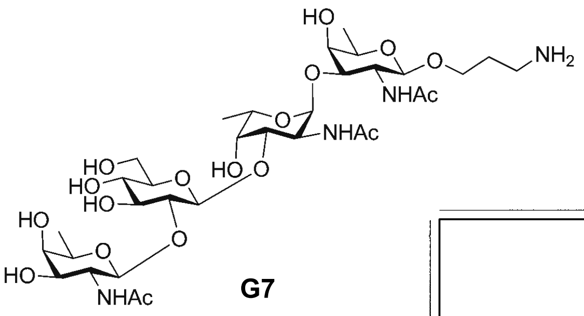

G7

NOESYPHPR

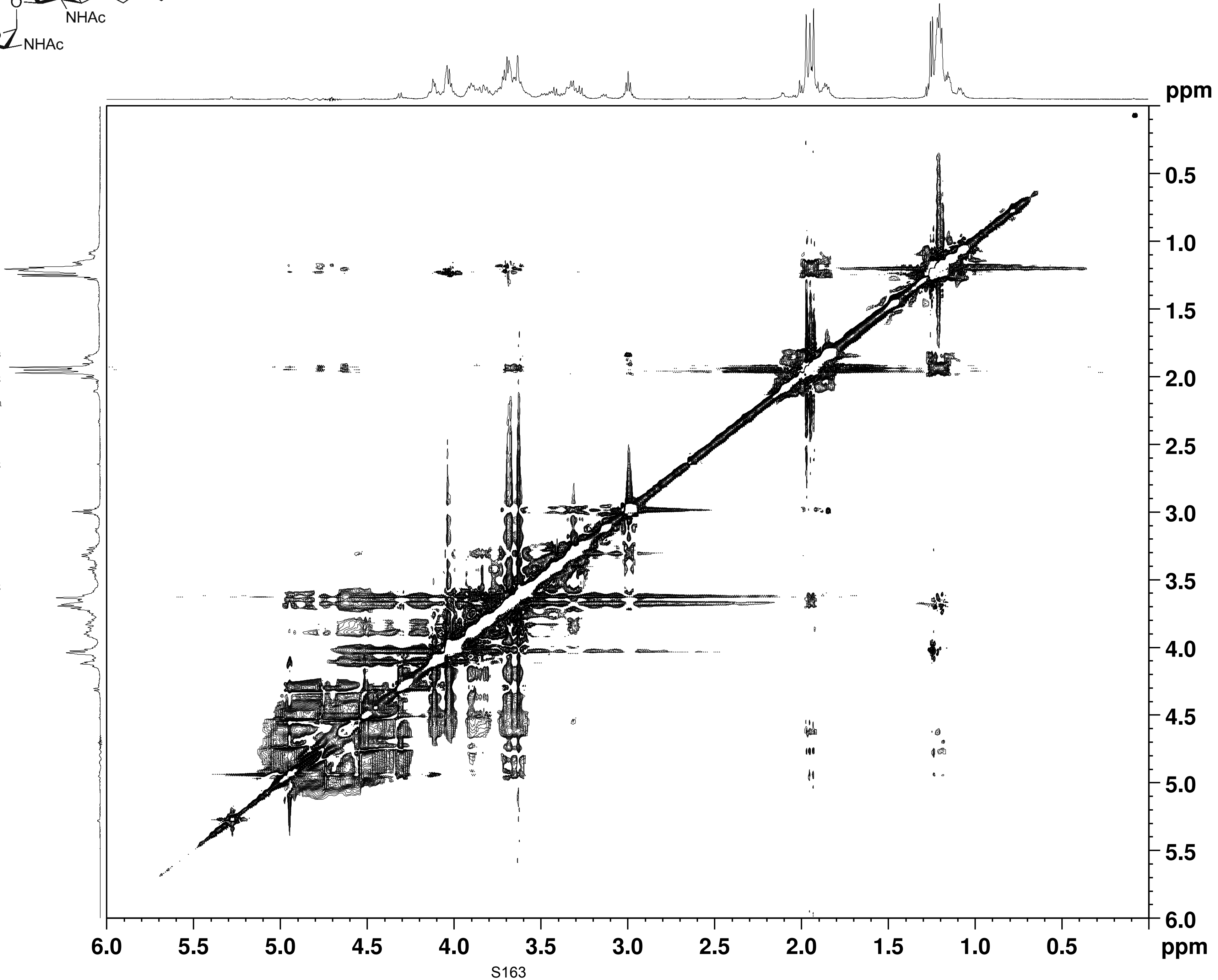


```
Current Data Parameters
NAME          SSK-AKM-1
EXPNO                12
PROCNO                1

F1 - Acquisition parameters
TD                  256
SFO1       600.3728 MHz
FIDRES    48.828125 Hz
SW           10.410 ppm
FnMODE      States-TPPI

F2 - Processing parameters
SI                 2048
SF      600.3700000 MHz
WDW               QSINE
SSB                   2
LB                 0 Hz
GB                    0
PC                 1.00

F1 - Processing parameters
SI                 1024
MC2         States-TPPI
SF      600.3700000 MHz
WDW               QSINE
SSB                   2
LB                 0 Hz
GB                    0
```

HSQCETGP

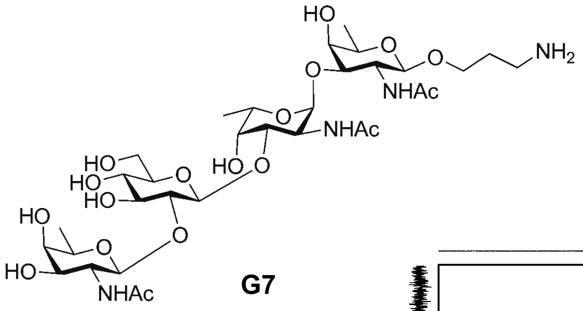

G7

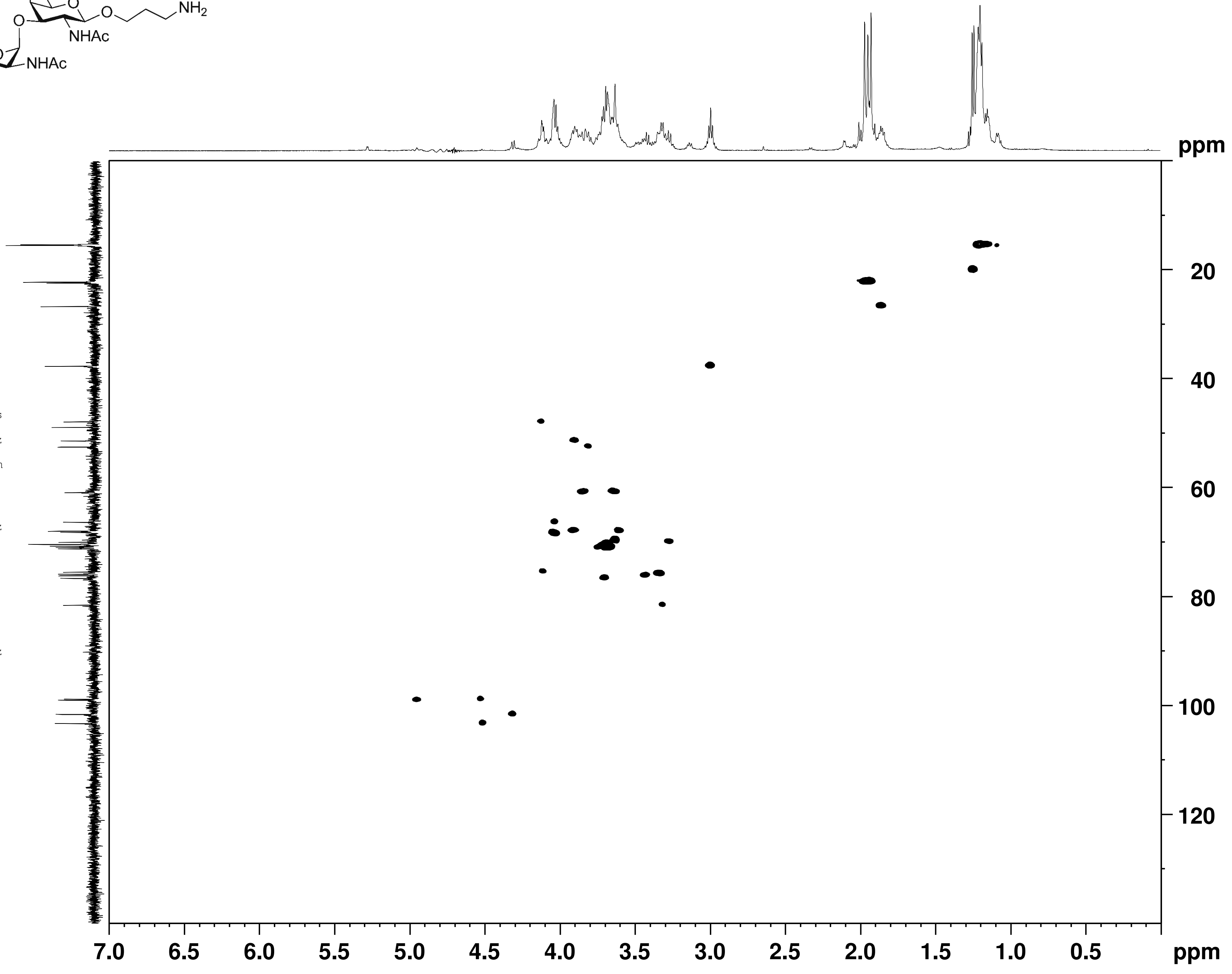


```
Current Data Parameters
NAME              SSK-AKM
EXPNO                  14
PROCNO                  1

F1 - Acquisition parameters
TD                    256
SFO1          150.9745 MHz
FIDRES      194.615540 Hz
SW             165.000 ppm
FnMODE    Echo-Antiecho

F2 - Processing parameters
SI                   1024
SF         600.3700000 MHz
WDW                 QSINE
SSB                     2
LB                      0 Hz
GB                      0
PC                   1.00

F1 - Processing parameters
SI                   1024
MC2         echo-antiecho
SF         150.9631566 MHz
WDW                 QSINE
SSB                     2
LB                      0 Hz
GB                      0
```

HMBCGP with water suppression

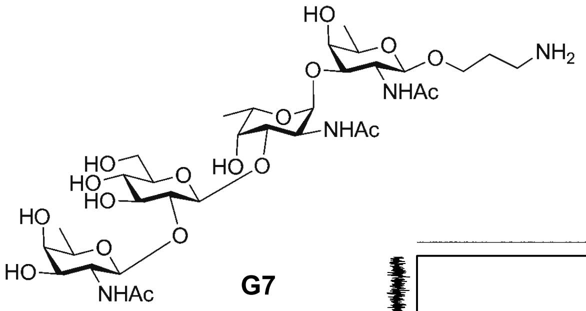

G7

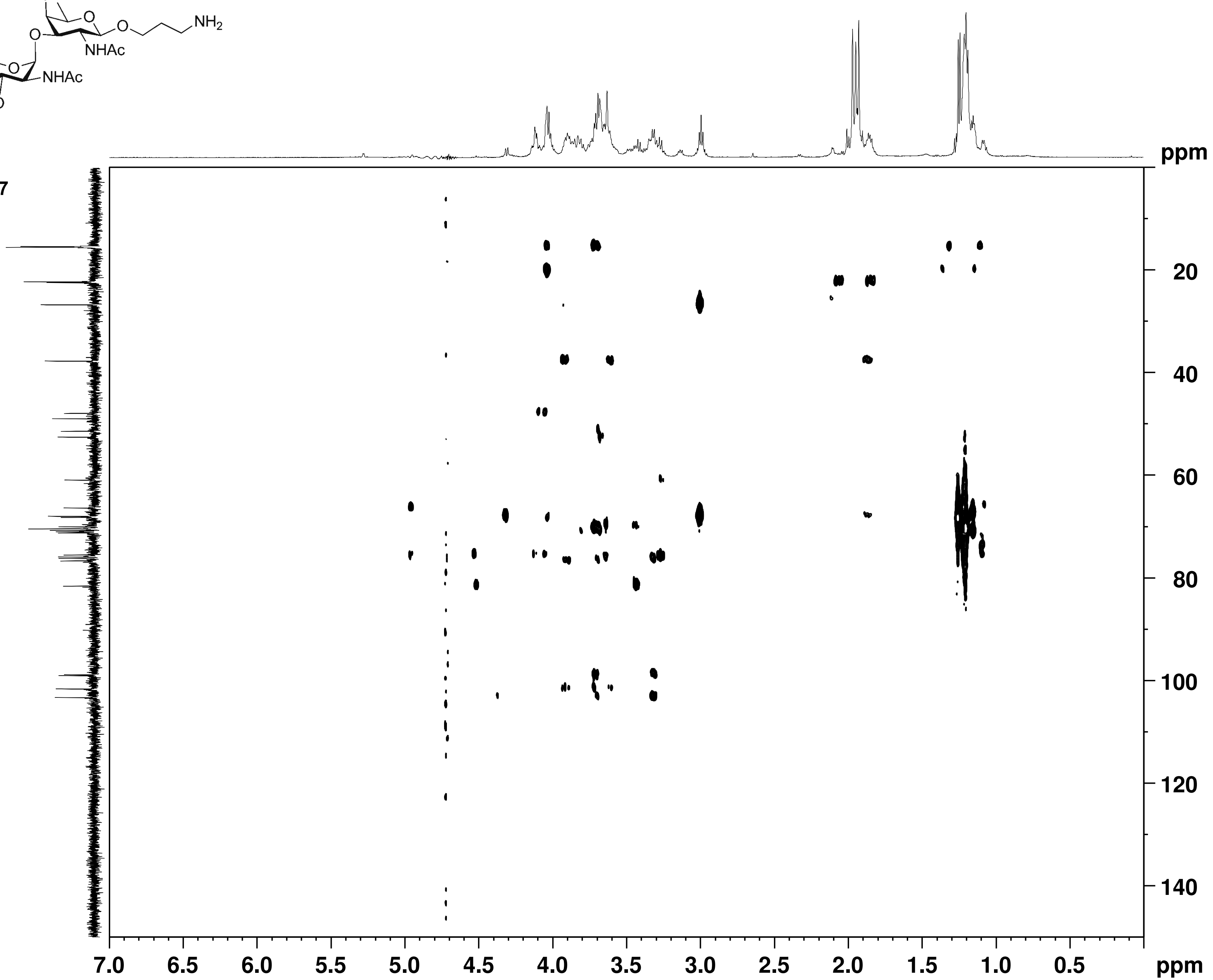


```
Current Data Parameters
NAME             SSK-AKM
EXPNO                 12
PROCNO                 1

F1 - Acquisition parameters
TD                   128
SFO1         150.9783 MHz
FIDRES     518.987732 Hz
SW            220.000 ppm
FnMODE                QF

F2 - Processing parameters
SI                  2048
SF          600.3700000 MHz
WDW                QSINE
SSB                    2
LB                     0 Hz
GB                     0
PC                  1.00

F1 - Processing parameters
SI                  1024
MC2                   QF
SF          150.9631566 MHz
WDW                QSINE
SSB                    2
LB                     0 Hz
GB                     0
```

HSQCETGP

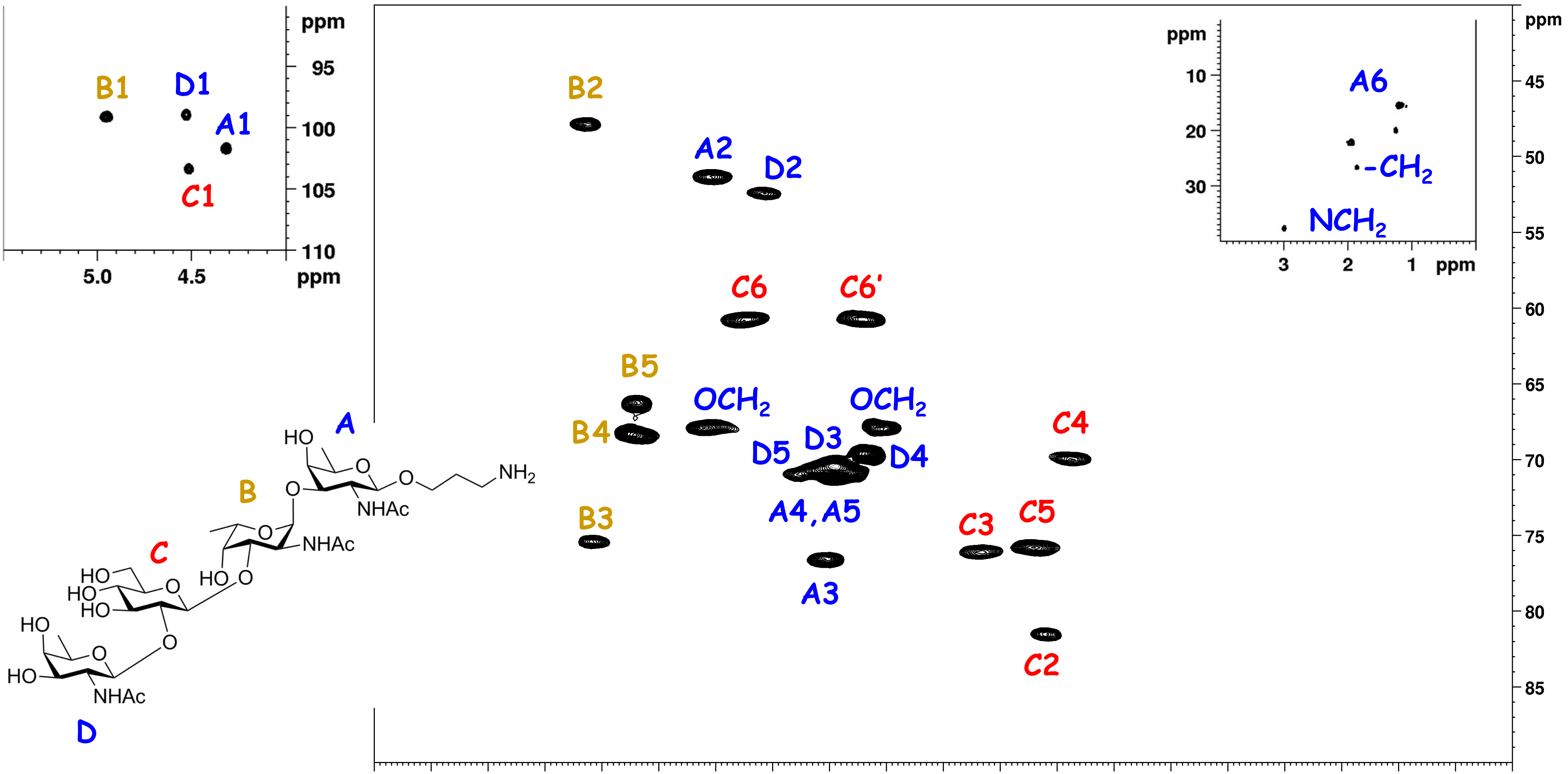
B1
D1
A1
C1
ppm
95
100
105
110
5.0
4.5
ppm
B2
A2
D2
C6
C6'
B5
B4
OCH2
OCH2
D3
D5
D4
C4
A4, A5
B3
C3
C5
A3
C2
A6
-CH2
NCH2
ppm
10
20
30
3
2
1
ppm
45
50
55
60
65
70
75
80
85
4.4
4.3
4.2
4.1
4.0
3.9
3.8
3.7
3.6
3.5
3.4
3.3
3.2
3.1
3.0
2.9
2.8
2.7
2.6
ppm
HO
A
O
O
NH2
B
O
NHAc
O
NHAc
C
HO
O
HO
O
HO
HO
HO
HO
O
O
HO
NHAc
D

20

SSK-34-AKM-508-TRI-1H

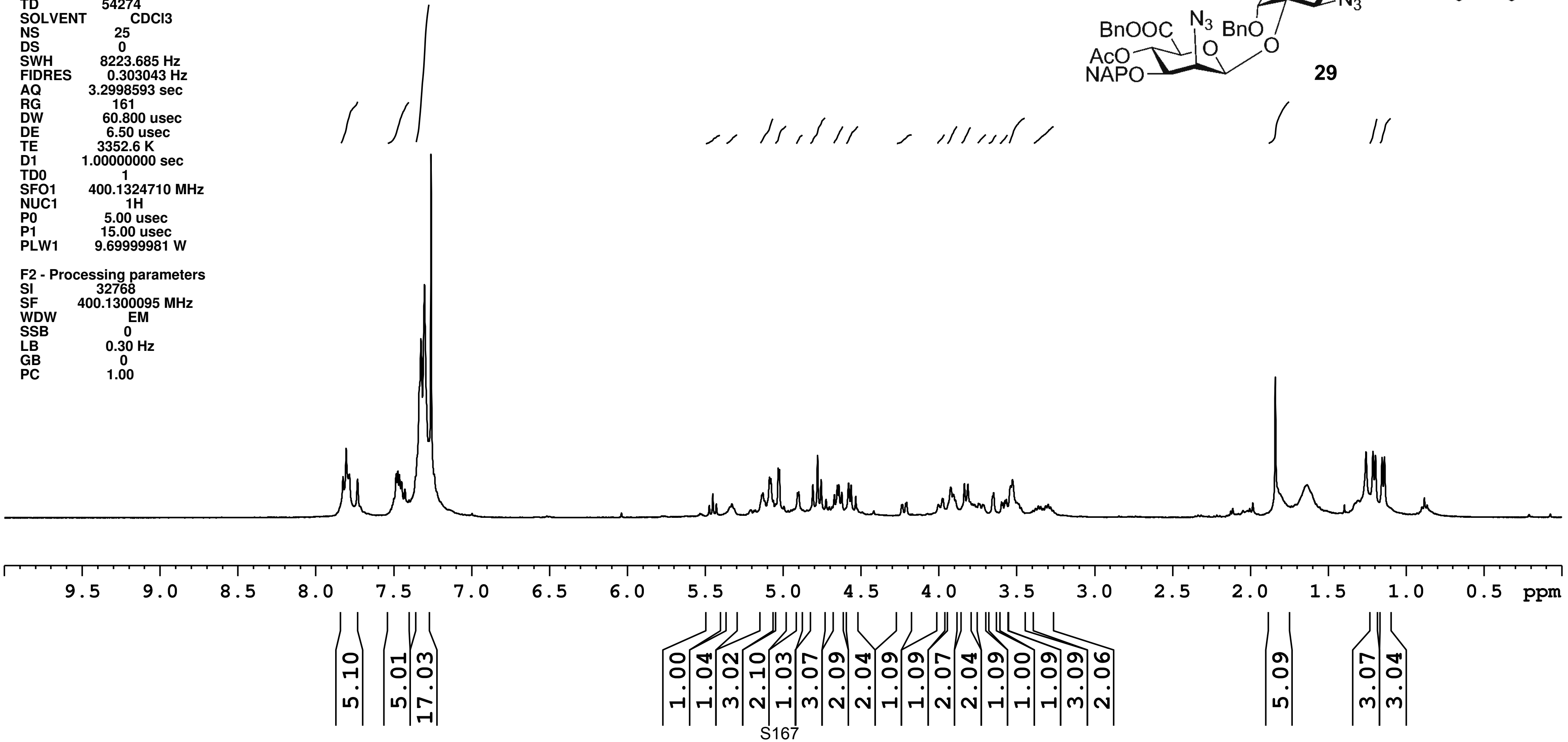

Current Data Parameters
NAME SSK-34-AKM-508-TRI-1H
EXPNO 1
PROCNO 1

F2 - Acquisition Parameters
Date_ 20240616
Time 15.26 h
INSTRUM spect
PROBHD Z104450_0346 (
PULPROG zg30
TD 54274
SOLVENT CDCl3
NS 25
DS 0
SWH 8223.685 Hz
FIDRES 0.303043 Hz
AQ 3.2998593 sec
RG 161
DW 60.800 usec
DE 6.50 usec
TE 3352.6 K
D1 1.00000000 sec
TD0 1
SFO1 400.1324710 MHz
NUC1 1H
P0 5.00 usec
P1 15.00 usec
PLW1 9.69999981 W

F2 - Processing parameters
SI 32768
SF 400.1300095 MHz
WDW EM
SSB 0
LB 0.30 Hz
GB 0
PC 1.00

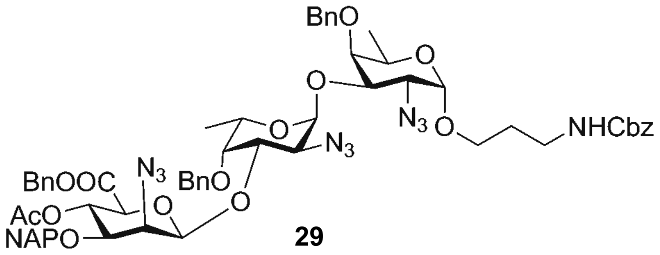

29

SSK-34-AKM-508-TRI-13C

Current Data Parameters
NAME SSK-34-AKM-508-TRI-13C
EXPNO 4
PROCNO 1

F2 - Acquisition Parameters
Date_ 20240617
Time 8.28 h
INSTRUM spect
PROBHD Z104450_0346 (
PULPROG zgpg30
TD 65536
SOLVENT CDCl3
NS 12377
DS 0
SWH 26041.666 Hz
FIDRES 0.794729 Hz
AQ 1.2582912 sec
RG 2050
DW 19.200 usec
DE 6.50 usec
TE 3308.9 K
D1 1.00000000 sec
D11 0.03000000 sec
TD0 1
SFO1 100.6238364 MHz
NUC1 13C
P0 3.33 usec
P1 10.00 usec
PLW1 47.00000000 W
SFO2 400.1316005 MHz
NUC2 1H
CPDPRG[2 waltz16
PCPD2 90.00 usec
PLW2 9.69999981 W
PLW12 0.26944000 W
PLW13 0.13552999 W

F2 - Processing parameters
SI 32768
SF 100.6127690 MHz
WDW EM
SSB 0
LB 1.00 Hz
GB 0
PC 1.40

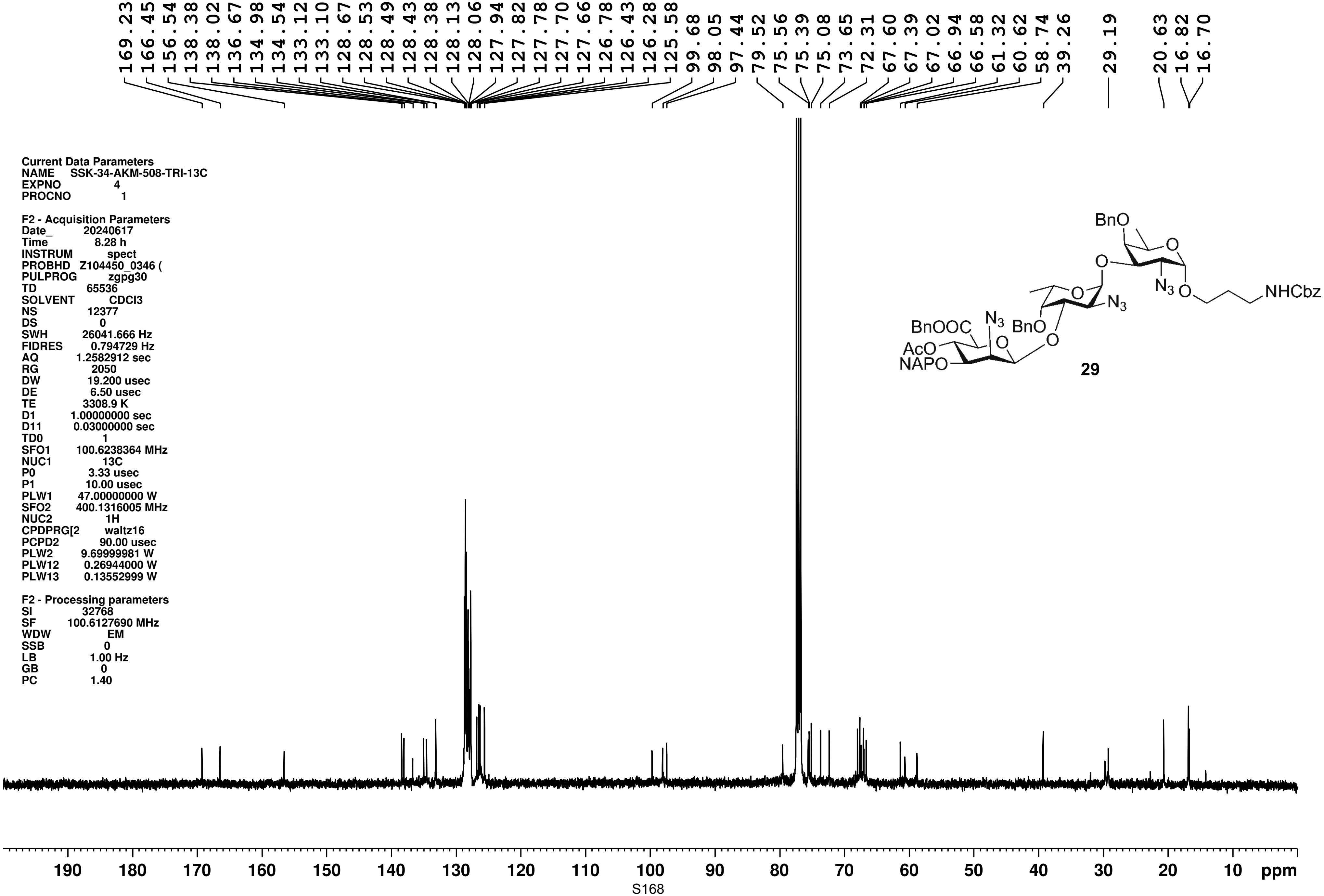

29

SSK-34-AKM-508-TRI-DEPT-

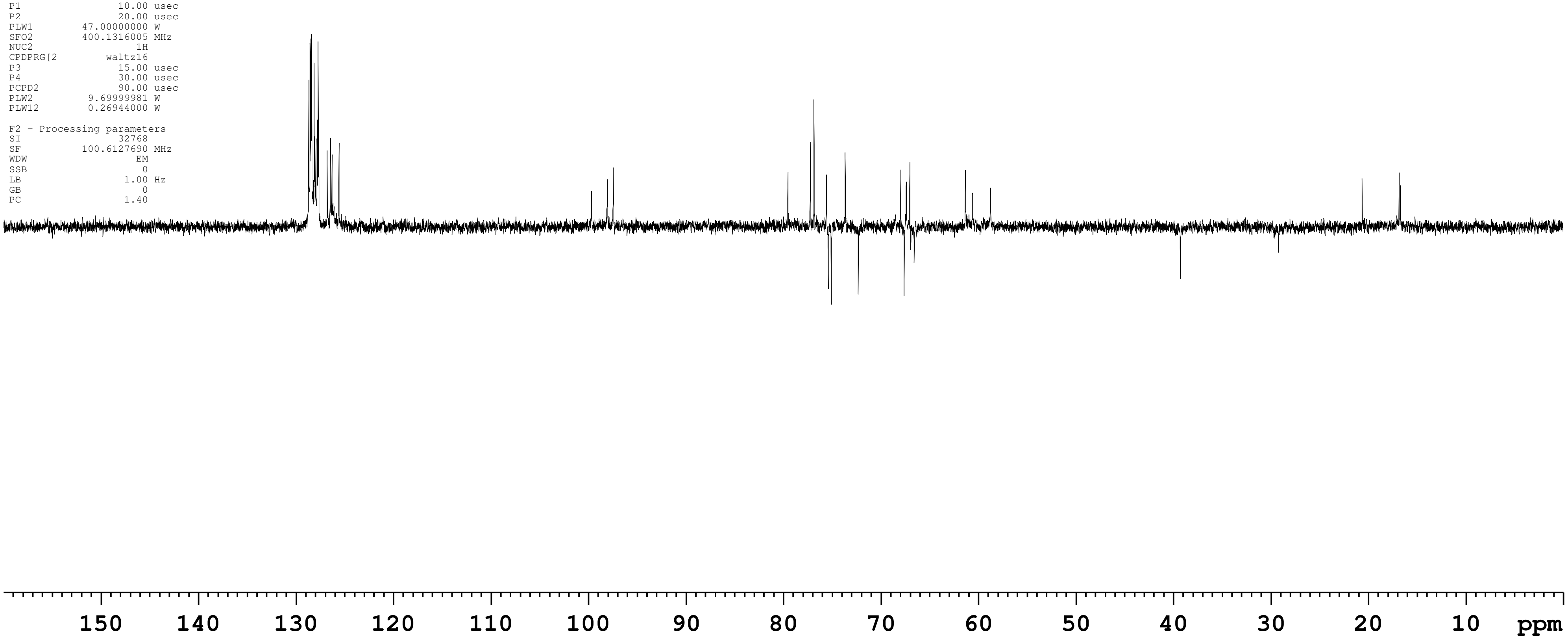


```
Current Data Parameters
NAME     SSK-34-AKM-508-TRI-DEPT-1
EXPNO                    3
PROCNO                   1

F2 - Acquisition Parameters
Date_          20240616
Time              17.49 h
INSTRUM           spect
PROBHD   Z104450_0346 (
PULPROG          dept135
TD                65536
SOLVENT           CDCl3
NS                 2465
DS                    0
SWH           27777.777 Hz
FIDRES         0.847710 Hz
AQ            1.1796480 sec
RG                 2050
DW               18.000 usec
DE                 6.50 usec
TE               3364.7 K
CNST2       145.0000000
D1           1.00000000 sec
D2           0.00344828 sec
D12          0.00002000 sec
TD0                   1
SFO1        100.6242389 MHz
NUC1                13C
P1                10.00 usec
P2                20.00 usec
PLW1        47.00000000 W
SFO2        400.1316005 MHz
NUC2                 1H
CPDPRG[2        waltz16
P3                15.00 usec
P4                30.00 usec
PCPD2             90.00 usec
PLW2         9.69999981 W
PLW12        0.26944000 W

F2 - Processing parameters
SI                32768
SF          100.6127690 MHz
WDW                  EM
SSB                   0
LB                 1.00 Hz
GB                    0
PC                 1.40
```

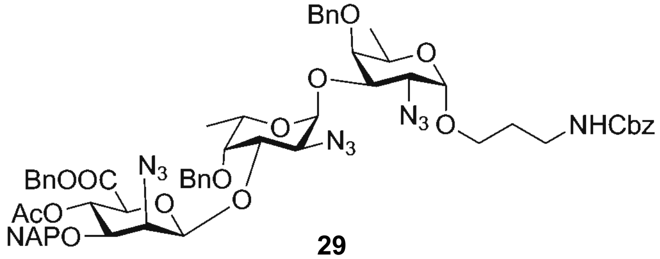

29

SSK-34-AKM-508-TRI-HSQC

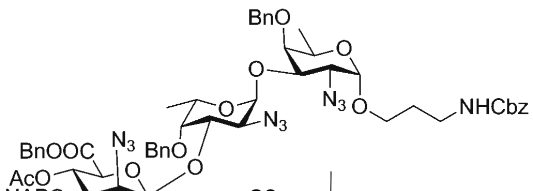

29

Current Data Parameters
NAME SSK-34-AKM-508-TRI-HSQC
EXPNO 2
PROCNO 1

F2 - Acquisition Parameters
Date_ 20240616
Time 15.55 h
INSTRUM spect
PROBHD Z104450_0346 (
PULPROG hsqcetgp
TD 2048
SOLVENT CDCl3
NS 8
DS 0
SWH 3840.246 Hz
FIDRES 3.750240 Hz
AQ 0.2666496 sec
RG 2050
DW 130.200 usec
DE 6.50 usec
TE 3391.3 K
CNST2 145.0000000
D0 0.00000300 sec
D1 1.00000000 sec
D4 0.00172414 sec
D11 0.03000000 sec
D16 0.00020000 sec
IN0 0.00003000 sec
TDav 1
ZGOPTNS
SFO1 400.1319625 MHz
NUC1 1H
P1 15.00 usec
P2 30.00 usec
PLW1 9.69999981 W
SFO2 100.6212419 MHz
NUC2 13C
CPDPRG[2 garp
P3 10.00 usec
P4 20.00 usec
PCPD2 80.00 usec
PLW2 47.00000000 W
PLW12 0.73438001 W
GPNAM[1] SINE.100
GPZ1 80.00 %
GPNAM[2] SINE.100
GPZ2 20.10 %
P16 1000.00 usec

F1 - Acquisition parameters
TD 152
SFO1 100.6212 MHz
FIDRES 219.298248 Hz
SW 165.638 ppm
FnMODE Echo-Antiecho

F2 - Processing parameters
SI 2048
SF 400.1300000 MHz
WDW QSINE
SSB 2
LB 0 Hz
GB 0
PC 1.40

F1 - Processing parameters
SI 1024
MC2 echo-antiecho
SF 100.6127690 MHz
WDW QSINE
SSB 2
LB 0 Hz
GB 0

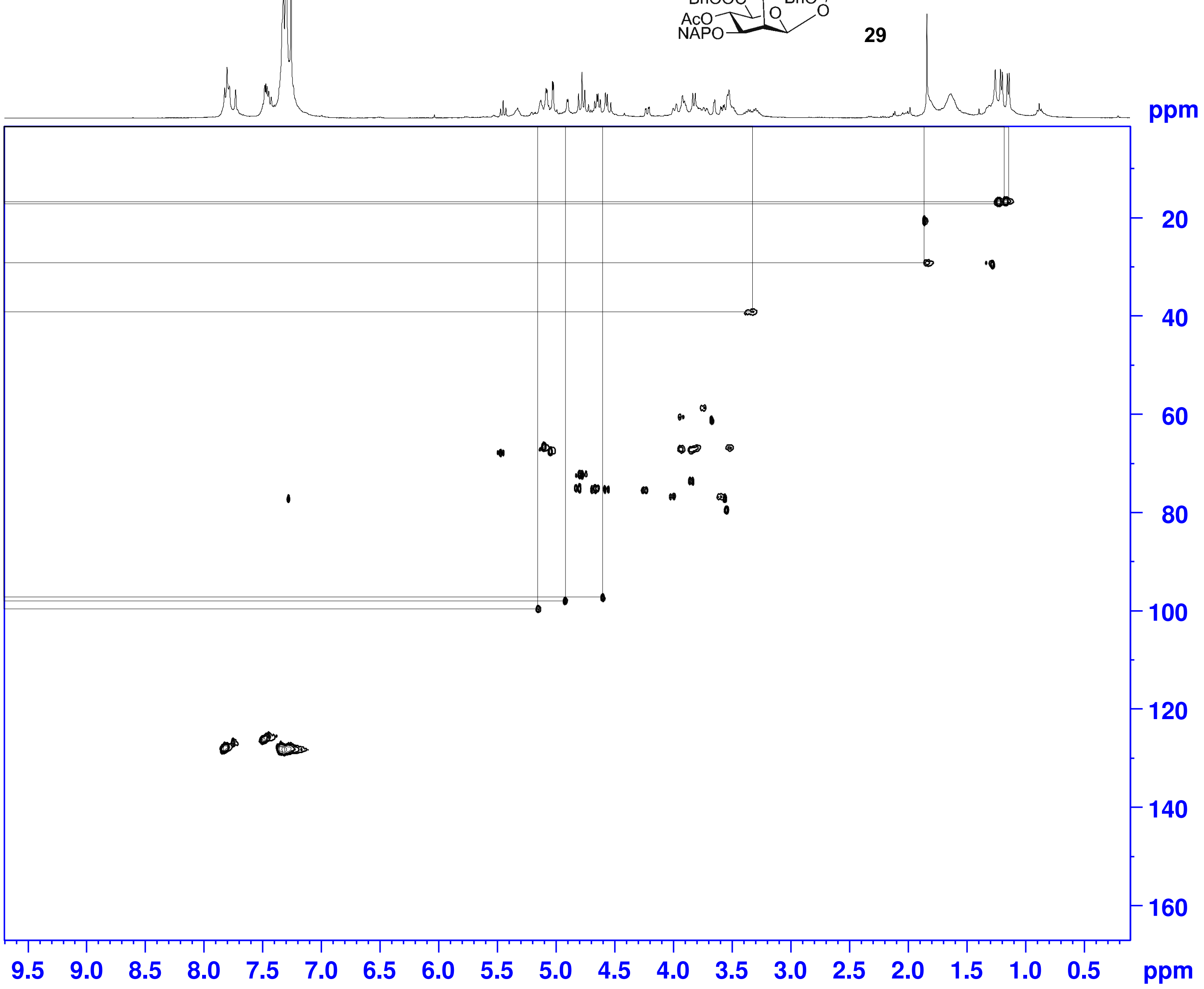

SSK-34-AKM-508-SA-TRI-CHCOUPLING

```
Current Data Parameters
NAME     SSK-34-AKM-508-SA-TRI-CHCOUPLING
EXPNO                   3
PROCNO                  1

F2 - Acquisition Parameters
Date_            20240619
Time                13.56 h
INSTRUM             spect
PROBHD   Z104450_0346 (
PULPROG            zggd30
TD                  65536
SOLVENT             CDCl3
NS                   4590
DS                      0
SWH             27777.777 Hz
FIDRES           0.847710 Hz
AQ              1.1796480 sec
RG                   2050
DW                 18.000 usec
DE                   6.50 usec
TE                -2487.8 K
D1             1.00000000 sec
D11            0.03000000 sec
TD0                     1
SFO1          100.6242389 MHz
NUC1                  13C
P0                   3.33 usec
P1                  10.00 usec
PLW1          47.00000000 W
SFO2          400.1316005 MHz
NUC2                   1H
CPDPRG[2          waltz16
PCPD2               90.00 usec
PLW2           9.69999981 W
PLW12          0.26944000 W

F2 - Processing parameters
SI                  32768
SF            100.6127690 MHz
WDW                    EM
SSB                     0
LB                   1.00 Hz
GB                      0
PC                   1.40
```

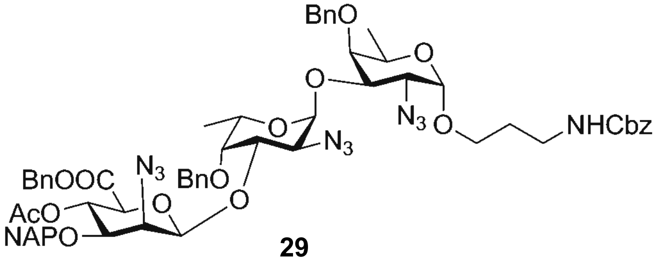

29

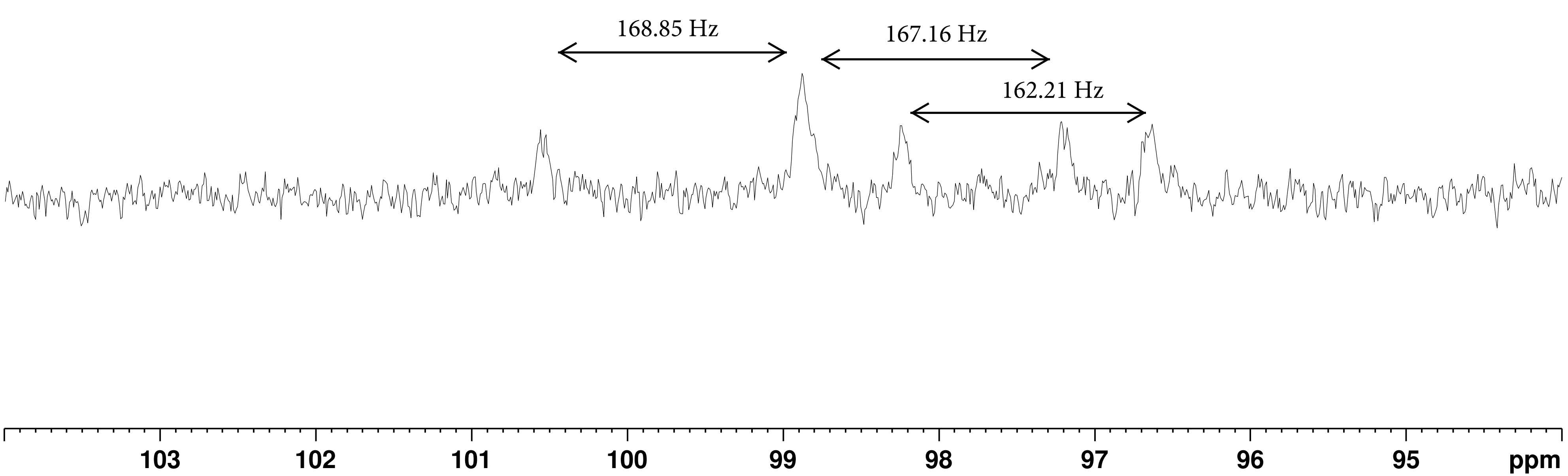

SSK-34-AKM-FINAL-CP8-1H

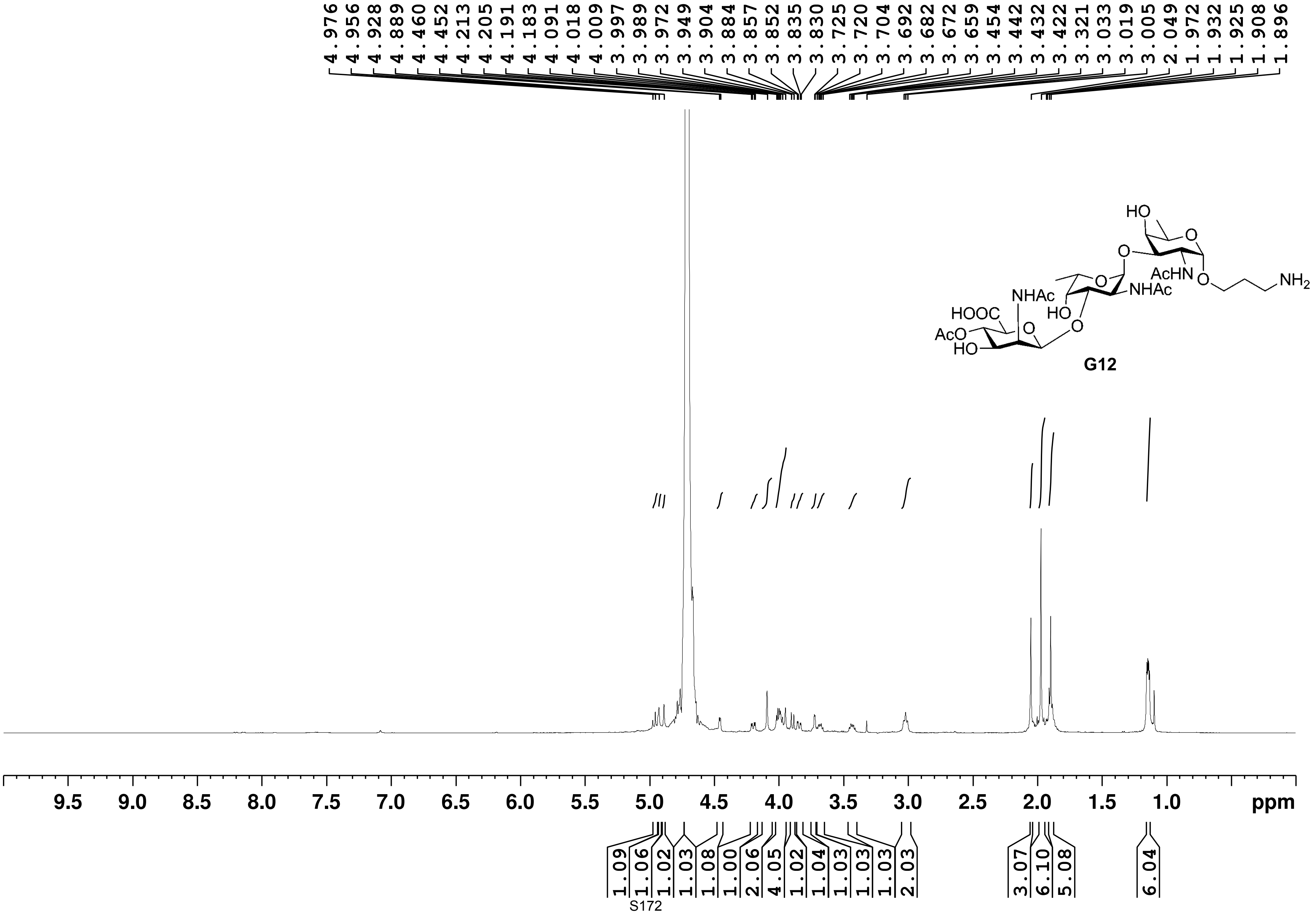

SSK-34-AKM-FINAL-CP8-13C

Current Data Parameters
NAME SSK-34-AKM-FINAL-CP8-13C
EXPNO 4
PROCNO 1

F2 - Acquisition Parameters
Date_ 20241213
Time 9.06 h
INSTRUM spect
PROBHD Z119470_0087 (
PULPROG zgpg30
TD 65536
SOLVENT D2O
NS 14609
DS 0
SWH 34722.223 Hz
FIDRES 1.059638 Hz
AQ 0.9437184 sec
RG 197.27
DW 14.400 usec
DE 6.50 usec
TE 297.3 K
D1 1.00000000 sec
D11 0.03000000 sec
TD0 1
SFO1 125.7721254 MHz
NUC1 13C
P0 2.97 usec
P1 8.90 usec
PLW1 103.00000000 W
SFO2 500.1320005 MHz
NUC2 1H
CPDPRG[2 waltz16
PCPD2 80.00 usec
PLW2 16.00000000 W
PLW12 0.44556001 W
PLW13 0.22411001 W

F2 - Processing parameters
SI 32768
SF 125.7577890 MHz
WDW EM
SSB 0
LB 1.00 Hz
GB 0
PC 1.40

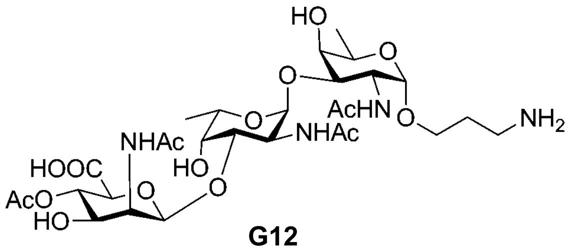

G12

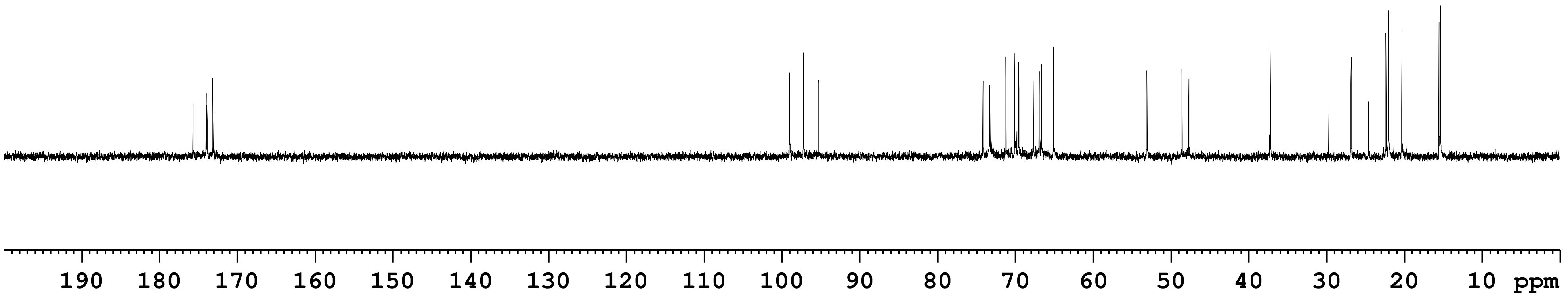

SSK-34-AKM-FINAL-CP8-DEPT

```
Current Data Parameters
NAME     SSK-34-AKM-FINAL-CP8-DEPT
EXPNO                     3
PROCNO                    1

F2 - Acquisition Parameters
Date_            20241212
Time                13.04 h
INSTRUM             spect
PROBHD   Z104450_0346 (
PULPROG          dept135
TD                  65536
SOLVENT               D2O
NS                    610
DS                      0
SWH             27777.777 Hz
FIDRES           0.847710 Hz
AQ              1.1796480 sec
RG                    203
DW                 18.000 usec
DE                   6.50 usec
TE                  177.9 K
CNST2         145.0000000
D1             1.00000000 sec
D2             0.00344828 sec
D12            0.00002000 sec
TD0                     1
SFO1          100.6242389 MHz
NUC1                  13C
P1                  10.00 usec
P2                  20.00 usec
PLW1          47.00000000 W
SFO2          400.1316005 MHz
NUC2                   1H
CPDPRG[2          waltz16
P3                  15.00 usec
P4                  30.00 usec
PCPD2               90.00 usec
PLW2           9.69999981 W
PLW12          0.26944000 W

F2 - Processing parameters
SI                  32768
SF            100.6127690 MHz
WDW                    EM
SSB                     0
LB                   1.00 Hz
GB                      0
PC                   1.40
```

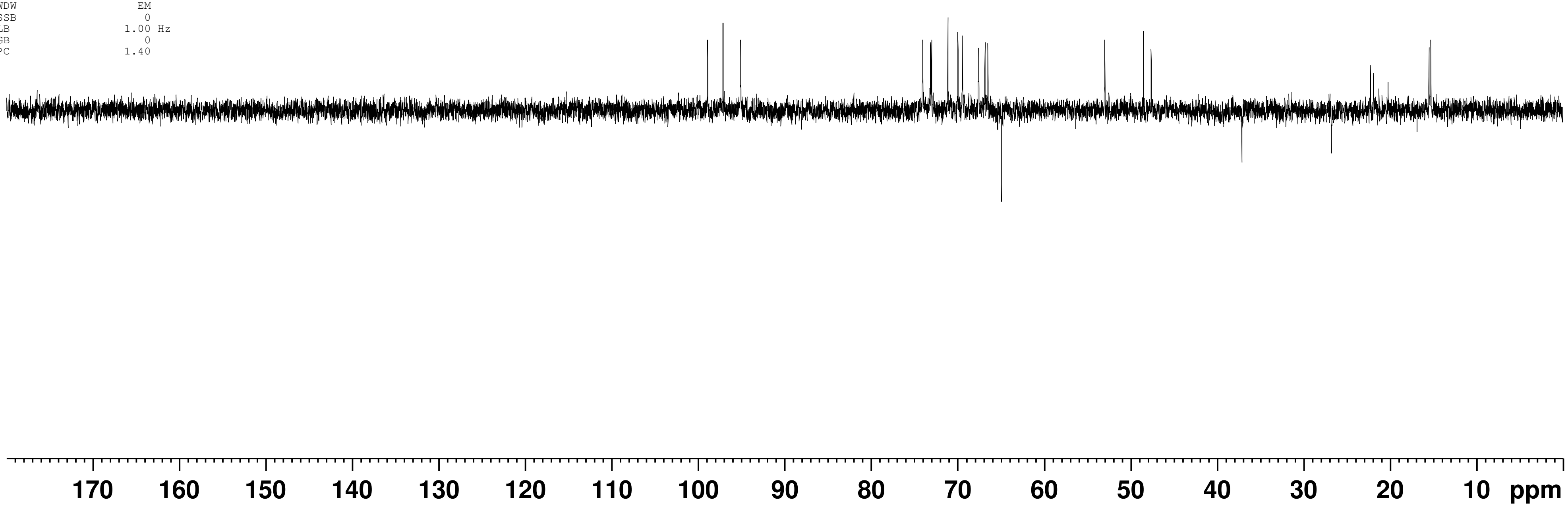

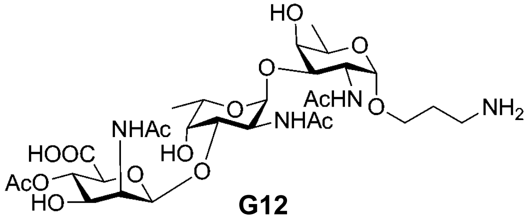

G12

SSK-34-AKM-CP8-HSQC

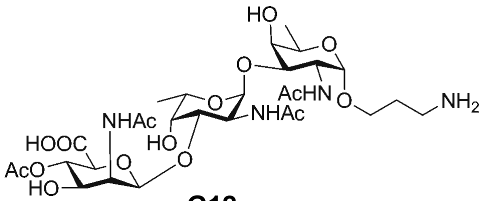

G12

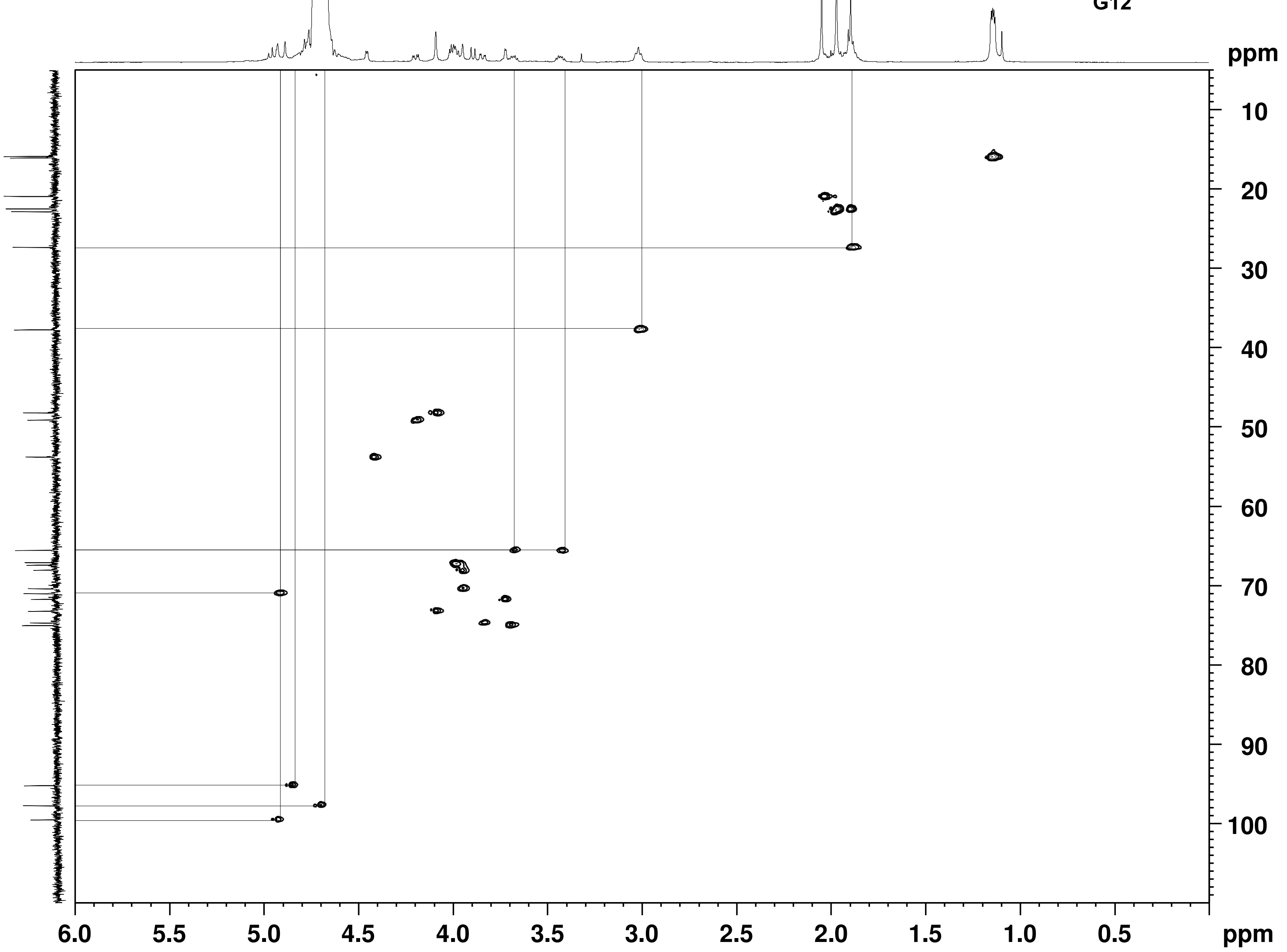


```
Current Data Parameters
NAME     SSK-34-AKM-CP8-M1-HSQC
EXPNO              2
PROCNO             1

F2 - Acquisition Parameters
Date_          20241225
Time              12.59 h
INSTRUM           spect
PROBHD   Z119470_0087 (
PULPROG  hsqcedetgpsisp2.2
TD                 1024
SOLVENT             D2O
NS                    4
DS                    0
SWH            5030.181 Hz
FIDRES         9.824573 Hz
AQ            0.1017856 sec
RG               197.27
DW               99.400 usec
DE                 6.50 usec
TE                296.2 K
CNST2       145.0000000
CNST17       -0.5000000
D0           0.00000300 sec
D1           1.00000000 sec
D4           0.00172414 sec
D11          0.03000000 sec
D16          0.00020000 sec
D21          0.00360000 sec
D24          0.00089000 sec
IN0          0.00003310 sec
TDav                  1
ZGOPTNS
SFO1        500.1324968 MHz
NUC1                 1H
P1                13.35 usec
P2                26.70 usec
PLW1        16.00000000 W
SFO2        125.7653265 MHz
NUC2                13C
CPDPRG[2 bi_p5m4sp_4sp.2
P3                 8.90 usec
P14              500.00 usec
P24             2000.00 usec
P63             1500.00 usec
PLW0                  0 W
PLW2       103.00000000 W
PLW12        1.66499996 W
SPNAM[3] Crp60,0.5,20.1
SPOAL3            0.500
SPOFFS3               0 Hz
SPW3        12.46500015 W
SPNAM[7]    Crp60comp.4
SPOAL7            0.500
SPOFFS7               0 Hz
SPW7        12.46500015 W
SPNAM[14 Crp32,1.5,20.2
SPOAL14           0.500
SPOFFS14              0 Hz
SPW14        5.31860018 W
SPNAM[31 Crp32,1.5,20.2
SPOAL31           0.500
SPOFFS31              0 Hz
SPW31        1.32969999 W
GPNAM[1]     SMSQ10.100
GPZ1              80.00 %
GPNAM[2]     SMSQ10.100
GPZ2              20.10 %
GPNAM[3]     SMSQ10.100
GPZ3              11.00 %
GPNAM[4]     SMSQ10.100
GPZ4              -5.00 %
P16             1000.00 usec
P19              600.00 usec

F1 - Acquisition parameters
TD                  237
SFO1           125.7653 MHz
FIDRES       127.474602 Hz
SW              120.111 ppm
FnMODE    Echo-Antiecho

F2 - Processing parameters
SI                 1024
SF          500.1300000 MHz
WDW               QSINE
SSB                   2
LB                    0 Hz
GB                    0
PC                 1.40

F1 - Processing parameters
SI                 1024
MC2      echo-antiecho
SF          125.7576987 MHz
WDW               QSINE
SSB                   2
LB                    0 Hz
GB                    0
```
